# Supplementary material for: The impact of Cochrane Reviews that apply network meta-analysis in clinical guidelines: A systematic review
Source: PLoS One. 2024 Dec 26;19(12):e0315563. doi: 10.1371/journal.pone.0315563 (PMC11671017; doi:10.1371/journal.pone.0315563)
Supplement: S1 File — (PDF) [file pone.0315563.s003.pdf]

File S1: Search strategy for Cochrane Database of Systematic Reviews

	**Search term**
#1	("network meta-analysis"):ti,ab,kw
#2	("mixed treatment comparison meta-analysis"):ti,ab,kw
#3	("multiple treatment comparison meta-analysis"):ti,ab,kw
#4	{OR #1-#3}
	Filter by Review Type Intervention
