# Supplementary material for: The impact of Cochrane Reviews that apply network meta-analysis in clinical guidelines: A systematic review
Source: PLoS One. 2024 Dec 26;19(12):e0315563. doi: 10.1371/journal.pone.0315563 (PMC11671017; doi:10.1371/journal.pone.0315563)
Supplement: S2 File — (PDF) [file pone.0315563.s004.pdf]

File S2: Eligibility assessment results

Review ID (reference)	Eligibility	Reason for exclusion
CD011383.pub2[1]	Excluded	Network meta-analysis not applied
CD013630.pub2[2]	Excluded	Network meta-analysis not applied
CD013649.pub2[3]	Excluded	Network meta-analysis not applied
CD014570.pub2[4]	Excluded	Network meta-analysis not applied
CD011837.pub2[5]	Excluded	Network meta-analysis not applied
CD007102.pub4[6]	Excluded	Network meta-analysis not applied
CD011567.pub2[7]	Excluded	Network meta-analysis not applied
CD012055.pub2[8]	Excluded	Network meta-analysis not applied
CD011343.pub2[9]	Excluded	Network meta-analysis not applied
CD011640.pub2[10]	Excluded	Network meta-analysis not applied
CD011645.pub2[11]	Excluded	Network meta-analysis not applied
CD011646.pub2[12]	Excluded	Network meta-analysis not applied
CD011647.pub2[13]	Excluded	Network meta-analysis not applied
CD011648.pub2[14]	Excluded	Network meta-analysis not applied
CD011649.pub2[15]	Excluded	Network meta-analysis not applied
CD011650.pub2[16]	Excluded	Network meta-analysis not applied
CD011384.pub2[17]	Excluded	Network meta-analysis not applied
CD011520.pub2[18]	Excluded	Network meta-analysis not applied
CD012234.pub2[19]	Excluded	Network meta-analysis not applied
CD010676.pub2[20]	Excluded	Network meta-analysis not applied
CD011644.pub3[21]	Excluded	Network meta-analysis not applied
CD010871.pub2[22]	Excluded	Network meta-analysis not applied
CD012930.pub2[23]	Excluded	Network meta-analysis not applied
CD001808.pub3[24]	Excluded	Network meta-analysis not applied
CD013620.pub2[25]	Excluded	Network meta-analysis not applied
CD013621.pub2[26]	Excluded	Network meta-analysis not applied
CD013622.pub2[27]	Excluded	Network meta-analysis not applied
CD013623.pub2[28]	Excluded	Network meta-analysis not applied
CD013624.pub2[29]	Excluded	Network meta-analysis not applied
CD011556.pub2[30]	Excluded	Network meta-analysis not applied
CD013499.pub2[31]	Excluded	Network meta-analysis not applied
CD013719.pub2[32]	Excluded	Network meta-analysis not applied
CD011392.pub2[33]	Excluded	Network meta-analysis not applied
CD003188.pub4[34]	Excluded	Main summary of findings table not based on network meta-analysis
CD005647.pub3[35]	Excluded	Main summary of findings table not based on network meta-analysis
CD000197.pub4[36]	Excluded	Main summary of findings table not based on network meta-analysis
CD012754.pub2[37]	Excluded	Main summary of findings table not based on network meta-analysis

Review ID (reference)	Eligibility	Reason for exclusion
CD013306.pub2[38]	Excluded	Main summary of findings table not based on network meta-analysis
CD013650.pub2[39]	Excluded	Main summary of findings table not based on network meta-analysis
CD003949.pub4[40]	Excluded	No summary of findings table. Grade results reported with network meta-analysis results table but presented pairwise meta-analysis results first.
CD009052.pub2[41]	Excluded	Summary of findings table not based on network meta-analysis
CD008933.pub2[42]	Excluded	Summary of findings table not based on network meta-analysis
CD009259.pub2[43]	Excluded	Summary of findings table not based on network meta-analysis
CD009036.pub2[44]	Excluded	Summary of findings table not based on network meta-analysis
CD011841.pub2[45]	Excluded	Summary of findings table not based on network meta-analysis
CD010683.pub3[46]	Excluded	Summary of findings table not based on network meta-analysis
CD011123.pub2[47]	Excluded	Summary of findings table not based on network meta-analysis
CD006187.pub3[48]	Excluded	Summary of findings table not based on network meta-analysis
CD003006.pub4[49]	Excluded	Summary of findings table not based on network meta-analysis
CD013156.pub2[50]	Excluded	Summary of findings table not based on network meta-analysis
CD013039.pub2[51]	Excluded	Summary of findings table not based on network meta-analysis
CD013157.pub2[52]	Excluded	Summary of findings table not based on network meta-analysis
CD013307.pub3[53]	Excluded	Summary of findings table not based on network meta-analysis
CD010216.pub8[54]	Excluded	Summary of findings table not based on network meta-analysis
CD013761.pub2[55]	Excluded	Overview of reviews
CD013180.pub2[56]	Excluded	Overview of reviews
CD013229.pub2[57]	Excluded	Overview of reviews
CD009329.pub2[58]	Excluded	Overview of reviews
CD008794.pub2[59]	Excluded	Overview of reviews
CD012591[60]	Excluded	Overview of reviews
CD012657[61]	Excluded	Overview of reviews
CD012437[62]	Excluded	Overview of reviews
CD010227.pub2[63]	Excluded	Overview of reviews

Review ID (reference)	Eligibility	Reason for exclusion
CD012183[64]	Excluded	Overview of reviews
CD010471.pub2[65]	Excluded	Overview of reviews
CD010314.pub2[66]	Excluded	Overview of reviews
CD002898.pub5[67]	Excluded	Indirect comparison methods used
CD007419.pub7[68]	Included	NA
CD010529.pub3[69]	Included	NA
CD010590.pub3[70]	Included	NA
CD011381.pub3[71]	Included	NA
CD011535.pub6[72]	Included	NA
CD011947.pub2[73]	Included	NA
CD012186.pub2[74]	Included	NA
CD012191.pub2[75]	Included	NA
CD012633.pub2[76]	Included	NA
CD012692.pub2[77]	Included	NA
CD012729.pub3[78]	Included	NA
CD012775.pub2[79]	Included	NA
CD013020.pub2[80]	Included	NA
CD013120.pub2[81]	Included	NA
CD013123.pub2[82]	Included	NA
CD013155.pub2[83]	Included	NA
CD013210.pub2[84]	Included	NA
CD013252.pub2[85]	Included	NA
CD013261.pub2[86]	Included	NA
CD013325.pub2[87]	Included	NA
CD013361.pub2[88]	Included	NA
CD013404.pub2[89]	Included	NA
CD013405.pub2[90]	Included	NA
CD013579.pub2[91]	Included	NA
CD013656.pub2[92]	Included	NA
CD013700.pub2[93]	Included	NA
CD013730.pub2[94]	Included	NA
CD013797.pub2[95]	Included	NA
CD013798.pub2[96]	Included	NA
CD013799.pub2[97]	Included	NA
CD013846.pub2[98]	Included	NA
CD014682.pub2[99]	Included	NA
CD014758.pub2[100]	Included	NA
CD015226.pub2[101]	Included	NA
CD014978.pub2[102]	Included	NA
CD012602.pub2[103]	Included	NA
CD013198.pub2[104]	Included	NA
CD011639.pub2[105]	Included	NA
CD013122.pub2[106]	Included	NA

Review ID (reference)	Eligibility	Reason for exclusion
CD013121.pub2[107]	Included	NA
CD013487[108]	Included	NA
CD013203.pub2[109]	Included	NA
CD006768.pub3[110]	Included	NA
CD012859.pub2[111]	Included	NA
CD013103.pub2[112]	Included	NA
CD012583.pub2[113]	Included	NA
CD011749.pub2[114]	Included	NA
CD013206.pub2[115]	Included	NA
CD013792.pub2[116]	Included	NA
CD011004.pub2[117]	Included	NA
CD011412.pub4[118]	Included	NA
CD010844.pub2[119]	Included	NA
CD011867.pub2[120]	Included	NA
CD012620.pub2[121]	Included	NA
CD011689.pub3[122]	Included	NA
CD010813.pub2[123]	Included	NA
CD007868.pub3[124]	Included	NA
CD013125.pub2[125]	Included	NA
CD013674.pub2[126]	Included	NA
CD013856.pub2[127]	Included	NA

References

1. Gurusamy KS, Belgaumkar AP, Haswell A, Pereira SP, Davidson BR. Interventions for necrotising pancreatitis. *Cochrane Database Syst Rev* 2016(4) doi: 10.1002/14651858.CD011383.pub2
2. Fountain DM, Bryant A, Barone DG, et al. Intraoperative imaging technology to maximise extent of resection for glioma: a network meta-analysis. *Cochrane Database Syst Rev* 2021(1) doi: 10.1002/14651858.CD013630.pub2
3. Beverly A, Ong G, Kimber C, et al. Drugs to reduce bleeding and transfusion in major open vascular or endovascular surgery: a systematic review and network meta-analysis. *Cochrane Database Syst Rev* 2023(2) doi: 10.1002/14651858.CD013649.pub2
4. Lynggaard LS, Rank CU, Als-Nielsen B, et al. PEG-asparaginase treatment regimens for acute lymphoblastic leukaemia in children: a network meta-analysis. *Cochrane Database Syst Rev* 2023(5) doi: 10.1002/14651858.CD014570.pub2
5. Lawrie TA, Nordin A, Chakrabarti M, et al. Medical and surgical interventions for the treatment of usual-type vulval intraepithelial neoplasia. *Cochrane Database Syst Rev* 2016(1) doi: 10.1002/14651858.CD011837.pub2
6. Lawrie TA, Alazzam M, Tidy J, Hancock BW, Osborne R. First-line chemotherapy in low-risk gestational trophoblastic neoplasia. *Cochrane Database Syst Rev* 2016(6) doi: 10.1002/14651858.CD007102.pub4
7. Bighelli I, Trespidi C, Castellazzi M, et al. Antidepressants and benzodiazepines for panic disorder in adults. *Cochrane Database Syst Rev* 2016(9) doi: 10.1002/14651858.CD011567.pub2
8. Desborough MJR, Hadjinicolaou AV, Chaimani A, et al. Alternative agents to prophylactic platelet transfusion for preventing bleeding in people with thrombocytopenia due to chronic bone marrow failure: a meta-analysis and systematic review. *Cochrane Database Syst Rev* 2016(10) doi: 10.1002/14651858.CD012055.pub2

9. Saffioti F, Gurusamy KS, Hawkins N, et al. Pharmacological interventions for primary sclerosing cholangitis. *Cochrane Database Syst Rev* 2017(3) doi: 10.1002/14651858.CD011343.pub2
10. Lombardi R, Onali S, Thorburn D, et al. Pharmacological interventions for non-alcohol related fatty liver disease (NAFLD). *Cochrane Database Syst Rev* 2017(3) doi: 10.1002/14651858.CD011640.pub2
11. Mantzoukis K, Rodríguez-Perálvarez M, Buzzetti E, et al. Pharmacological interventions for acute hepatitis B infection. *Cochrane Database Syst Rev* 2017(3) doi: 10.1002/14651858.CD011645.pub2
12. Buzzetti E, Kalafateli M, Thorburn D, et al. Pharmacological interventions for alcoholic liver disease (alcohol-related liver disease). *Cochrane Database Syst Rev* 2017(3) doi: 10.1002/14651858.CD011646.pub2
13. Buzzetti E, Kalafateli M, Thorburn D, et al. Interventions for hereditary haemochromatosis. *Cochrane Database Syst Rev* 2017(3) doi: 10.1002/14651858.CD011647.pub2
14. Saffioti F, Gurusamy KS, Eusebi LH, et al. Pharmacological interventions for primary biliary cholangitis. *Cochrane Database Syst Rev* 2017(3) doi: 10.1002/14651858.CD011648.pub2
15. Roccarina D, Majumdar A, Thorburn D, et al. Management of people with intermediate-stage hepatocellular carcinoma. *Cochrane Database Syst Rev* 2017(3) doi: 10.1002/14651858.CD011649.pub2
16. Majumdar A, Roccarina D, Thorburn D, et al. Management of people with early- or very early-stage hepatocellular carcinoma. *Cochrane Database Syst Rev* 2017(3) doi: 10.1002/14651858.CD011650.pub2
17. Moggia E, Koti R, Belgaumkar AP, et al. Pharmacological interventions for acute pancreatitis. *Cochrane Database Syst Rev* 2017(4) doi: 10.1002/14651858.CD011384.pub2
18. Koesters M, Ostuzzi G, Guaiana G, Breilmann J, Barbui C. Vortioxetine for depression in adults. *Cochrane Database Syst Rev* 2017(7) doi: 10.1002/14651858.CD011520.pub2
19. Norman G, Atkinson RA, Smith TA, et al. Intracavity lavage and wound irrigation for prevention of surgical site infection. *Cochrane Database Syst Rev* 2017(10) doi: 10.1002/14651858.CD012234.pub2
20. Bighelli I, Castellazzi M, Cipriani A, et al. Antidepressants versus placebo for panic disorder in adults. *Cochrane Database Syst Rev* 2018(4) doi: 10.1002/14651858.CD010676.pub2
21. Kalafateli M, Buzzetti E, Thorburn D, et al. Pharmacological interventions for acute hepatitis C infection. *Cochrane Database Syst Rev* 2018(12) doi: 10.1002/14651858.CD011644.pub3
22. Páez-Canro C, Alzate JP, González LM, et al. Antibiotics for treating urogenital Chlamydia trachomatis infection in men and non-pregnant women. *Cochrane Database Syst Rev* 2019(1) doi: 10.1002/14651858.CD010871.pub2
23. Maqsood U, Ho TN, Palmer K, et al. Once daily long-acting beta2-agonists and long-acting muscarinic antagonists in a combined inhaler versus placebo for chronic obstructive pulmonary disease. *Cochrane Database Syst Rev* 2019(3) doi: 10.1002/14651858.CD012930.pub2
24. Salati JA, Leathersich SJ, Williams MJ, Cuthbert A, Tolosa JE. Prophylactic oxytocin for the third stage of labour to prevent postpartum haemorrhage. *Cochrane Database Syst Rev* 2019(4) doi: 10.1002/14651858.CD001808.pub3
25. Shi C, Dumville JC, Cullum N, et al. Alternating pressure (active) air surfaces for preventing pressure ulcers. *Cochrane Database Syst Rev* 2021(5) doi: 10.1002/14651858.CD013620.pub2
26. Shi C, Dumville JC, Cullum N, Rhodes S, McInnes E. Foam surfaces for preventing pressure ulcers. *Cochrane Database Syst Rev* 2021(5) doi: 10.1002/14651858.CD013621.pub2
27. Shi C, Dumville JC, Cullum N, et al. Reactive air surfaces for preventing pressure ulcers. *Cochrane Database Syst Rev* 2021(5) doi: 10.1002/14651858.CD013622.pub2

28. Shi C, Dumville JC, Cullum N, Rhodes S, McInnes E. Alternative reactive support surfaces (non-foam and non-air-filled) for preventing pressure ulcers. *Cochrane Database Syst Rev* 2021(5) doi: 10.1002/14651858.CD013623.pub2
29. Shi C, Dumville JC, Cullum N, et al. Beds, overlays and mattresses for treating pressure ulcers. *Cochrane Database Syst Rev* 2021(5) doi: 10.1002/14651858.CD013624.pub2
30. Lindson N, Pritchard G, Hong B, et al. Strategies to improve smoking cessation rates in primary care. *Cochrane Database Syst Rev* 2021(9) doi: 10.1002/14651858.CD011556.pub2
31. Gibbs VN, Geneen LJ, Champaneria R, et al. Pharmacological interventions for the prevention of bleeding in people undergoing definitive fixation or joint replacement for hip, pelvic and long bone fractures. *Cochrane Database Syst Rev* 2023(6) doi: 10.1002/14651858.CD013499.pub2
32. Mateos-Haro M, Novoa-Candia M, Sánchez Vanegas G, et al. Treatments for alopecia areata: a network meta-analysis. *Cochrane Database Syst Rev* 2023(10) doi: 10.1002/14651858.CD013719.pub2
33. Gurusamy KS, Pallari E, Hawkins N, Pereira SP, Davidson BR. Management strategies for pancreatic pseudocysts. *Cochrane Database Syst Rev* 2016(4) doi: 10.1002/14651858.CD011392.pub2
34. Mhaskar R, Kumar A, Miladinovic B, Djulbegovic B. Bisphosphonates in multiple myeloma: an updated network meta-analysis. *Cochrane Database Syst Rev* 2017(12) doi: 10.1002/14651858.CD003188.pub4
35. Tenforde MW, Shapiro AE, Rouse B, et al. Treatment for HIV-associated cryptococcal meningitis. *Cochrane Database Syst Rev* 2018(7) doi: 10.1002/14651858.CD005647.pub3
36. Langhorne P, Ramachandra S. Organised inpatient (stroke unit) care for stroke: network meta-analysis. *Cochrane Database Syst Rev* 2020(4) doi: 10.1002/14651858.CD000197.pub4
37. Parry Smith WR, Papadopoulou A, Thomas E, et al. Uterotonic agents for first-line treatment of postpartum haemorrhage: a network meta-analysis. *Cochrane Database Syst Rev* 2020(11) doi: 10.1002/14651858.CD012754.pub2
38. Battle CE, Abdul-Rahim AH, Shenkin SD, Hewitt J, Quinn TJ. Cholinesterase inhibitors for vascular dementia and other vascular cognitive impairments: a network meta-analysis. *Cochrane Database Syst Rev* 2021(2) doi: 10.1002/14651858.CD013306.pub2
39. Kanie T, Mizuno A, Takaoka Y, et al. Dipeptidyl peptidase-4 inhibitors, glucagon-like peptide 1 receptor agonists and sodium-glucose co-transporter-2 inhibitors for people with cardiovascular disease: a network meta-analysis. *Cochrane Database Syst Rev* 2021(10) doi: 10.1002/14651858.CD013650.pub2
40. Dumville JC, McFarlane E, Edwards P, et al. Preoperative skin antiseptics for preventing surgical wound infections after clean surgery. *Cochrane Database Syst Rev* 2015(4) doi: 10.1002/14651858.CD003949.pub4
41. Gurusamy KS, Pissanou T, Pikhart H, et al. Methods to decrease blood loss and transfusion requirements for liver transplantation. *Cochrane Database Syst Rev* 2011(12) doi: 10.1002/14651858.CD009052.pub2
42. Filippini G, Del Giovane C, Vacchi L, et al. Immunomodulators and immunosuppressants for multiple sclerosis: a network meta-analysis. *Cochrane Database Syst Rev* 2013(6) doi: 10.1002/14651858.CD008933.pub2
43. Holme Ø, Bretthauer M, Fretheim A, Odgaard-Jensen J, Hoff G. Flexible sigmoidoscopy versus faecal occult blood testing for colorectal cancer screening in asymptomatic individuals. *Cochrane Database Syst Rev* 2013(9) doi: 10.1002/14651858.CD009259.pub2
44. Le Cleach L, Trinquart L, Do G, et al. Oral antiviral therapy for prevention of genital herpes outbreaks in immunocompetent and nonpregnant patients. *Cochrane Database Syst Rev* 2014(8) doi: 10.1002/14651858.CD009036.pub2
45. Salehi M, Wenick AS, Law HA, Evans JR, Gehlbach P. Interventions for central serous chorioretinopathy: a network meta-analysis. *Cochrane Database Syst Rev* 2015(12) doi: 10.1002/14651858.CD011841.pub2

46. Moggia E, Rouse B, Simillis C, et al. Methods to decrease blood loss during liver resection: a network meta-analysis. *Cochrane Database Syst Rev* 2016(10) doi: 10.1002/14651858.CD010683.pub3
47. Pasquali S, Hadjinicolaou AV, Chiarion Sileni V, Rossi CR, Mocellin S. Systemic treatments for metastatic cutaneous melanoma. *Cochrane Database Syst Rev* 2018(2) doi: 10.1002/14651858.CD011123.pub2
48. Langhorne P, Collier JM, Bate PJ, Thuy MNT, Bernhardt J. Very early versus delayed mobilisation after stroke. *Cochrane Database Syst Rev* 2018(10) doi: 10.1002/14651858.CD006187.pub3
49. Forget P, Borovac JA, Thackeray EM, Pace NL. Transient neurological symptoms (TNS) following spinal anaesthesia with lidocaine versus other local anaesthetics in adult surgical patients: a network meta-analysis. *Cochrane Database Syst Rev* 2019(12) doi: 10.1002/14651858.CD003006.pub4
50. Buzzetti E, Linden A, Best LMJ, et al. Lifestyle modifications for nonalcohol-related fatty liver disease: a network meta-analysis. *Cochrane Database Syst Rev* 2021(6) doi: 10.1002/14651858.CD013156.pub2
51. Schwendicke F, Walsh T, Lamont T, et al. Interventions for treating cavitated or dentine carious lesions. *Cochrane Database Syst Rev* 2021(7) doi: 10.1002/14651858.CD013039.pub2
52. Komolafe O, Buzzetti E, Linden A, et al. Nutritional supplementation for nonalcohol-related fatty liver disease: a network meta-analysis. *Cochrane Database Syst Rev* 2021(7) doi: 10.1002/14651858.CD013157.pub2
53. Burton JK, Craig L, Yong SQ, et al. Non-pharmacological interventions for preventing delirium in hospitalised non-ICU patients. *Cochrane Database Syst Rev* 2021(11) doi: 10.1002/14651858.CD013307.pub3
54. Lindson N, Butler AR, McRobbie H, et al. Electronic cigarettes for smoking cessation. *Cochrane Database Syst Rev* 2024(1) doi: 10.1002/14651858.CD010216.pub8
55. Shi C, Dumville JC, Cullum N, et al. Beds, overlays and mattresses for preventing and treating pressure ulcers: an overview of Cochrane Reviews and network meta-analysis. *Cochrane Database Syst Rev* 2021(8) doi: 10.1002/14651858.CD013761.pub2
56. Bofill Rodriguez M, Dias S, Jordan V, et al. Interventions for heavy menstrual bleeding; overview of Cochrane reviews and network meta-analysis. *Cochrane Database Syst Rev* 2022(5) doi: 10.1002/14651858.CD013180.pub2
57. Hartmann-Boyce J, Livingstone-Banks J, Ordóñez-Mena JM, et al. Behavioural interventions for smoking cessation: an overview and network meta-analysis. *Cochrane Database Syst Rev* 2021(1) doi: 10.1002/14651858.CD013229.pub2
58. Cahill K, Stevens S, Perera R, Lancaster T. Pharmacological interventions for smoking cessation: an overview and network meta-analysis. *Cochrane Database Syst Rev* 2013(5) doi: 10.1002/14651858.CD009329.pub2
59. Singh JA, Wells GA, Christensen R, et al. Adverse effects of biologics: a network meta-analysis and Cochrane overview. *Cochrane Database Syst Rev* 2011(2) doi: 10.1002/14651858.CD008794.pub2
60. Singh JA, Hossain A, Tanjong Ghogomu E, et al. Biologics or tofacitinib for people with rheumatoid arthritis unsuccessfully treated with biologics: a systematic review and network meta-analysis. *Cochrane Database Syst Rev* 2017(3) doi: 10.1002/14651858.CD012591
61. Singh JA, Hossain A, Mudano AS, et al. Biologics or tofacitinib for people with rheumatoid arthritis naive to methotrexate: a systematic review and network meta-analysis. *Cochrane Database Syst Rev* 2017(5) doi: 10.1002/14651858.CD012657
62. Singh JA, Hossain A, Tanjong Ghogomu E, et al. Biologic or tofacitinib monotherapy for rheumatoid arthritis in people with traditional disease-modifying anti-rheumatic drug (DMARD) failure: a Cochrane Systematic Review and network meta-analysis (NMA). *Cochrane Database Syst Rev* 2016(11) doi: 10.1002/14651858.CD012437

63. Hazlewood GS, Barnabe C, Tomlinson G, et al. Methotrexate monotherapy and methotrexate combination therapy with traditional and biologic disease modifying anti-rheumatic drugs for rheumatoid arthritis: A network meta-analysis. *Cochrane Database Syst Rev* 2016(8) doi: 10.1002/14651858.CD010227.pub2
64. Singh JA, Hossain A, Tanjong Ghogomu E, et al. Biologics or tofacitinib for rheumatoid arthritis in incomplete responders to methotrexate or other traditional disease-modifying anti-rheumatic drugs: a systematic review and network meta-analysis. *Cochrane Database Syst Rev* 2016(5) doi: 10.1002/14651858.CD012183
65. Wu L, Norman G, Dumville JC, O'Meara S, Bell-Syer SEM. Dressings for treating foot ulcers in people with diabetes: an overview of systematic reviews. *Cochrane Database Syst Rev* 2015(7) doi: 10.1002/14651858.CD010471.pub2
66. Cates CJ, Wieland LS, Oleszczuk M, Kew KM. Safety of regular formoterol or salmeterol in adults with asthma: an overview of Cochrane reviews. *Cochrane Database Syst Rev* 2014(2) doi: 10.1002/14651858.CD010314.pub2
67. Wilhelmus KR. Antiviral treatment and other therapeutic interventions for herpes simplex virus epithelial keratitis. *Cochrane Database Syst Rev* 2015(1) doi: 10.1002/14651858.CD002898.pub5
68. Virgili G, Curran K, Lucenteforte E, Peto T, Parravano M. Anti-vascular endothelial growth factor for diabetic macular oedema: a network meta-analysis. *Cochrane Database Syst Rev* 2023(6) doi: 10.1002/14651858.CD007419.pub7
69. Dipper A, Jones HE, Bhatnagar R, et al. Interventions for the management of malignant pleural effusions: a network meta-analysis. *Cochrane Database Syst Rev* 2020(4) doi: 10.1002/14651858.CD010529.pub3
70. Chung EYM, Palmer SC, Saglimbene VM, et al. Erythropoiesis-stimulating agents for anaemia in adults with chronic kidney disease: a network meta-analysis. *Cochrane Database Syst Rev* 2023(2) doi: 10.1002/14651858.CD010590.pub3
71. Gonzalez-Lorenzo M, Ridley B, Minozzi S, et al. Immunomodulators and immunosuppressants for relapsing-remitting multiple sclerosis: a network meta-analysis. *Cochrane Database Syst Rev* 2024(1) doi: 10.1002/14651858.CD011381.pub3
72. Sbidian E, Chaimani A, Guelimi R, et al. Systemic pharmacological treatments for chronic plaque psoriasis: a network meta-analysis. *Cochrane Database Syst Rev* 2023(7) doi: 10.1002/14651858.CD011535.pub6
73. Westby MJ, Dumville JC, Soares MO, Stubbs N, Norman G. Dressings and topical agents for treating pressure ulcers. *Cochrane Database Syst Rev* 2017(6) doi: 10.1002/14651858.CD011947.pub2
74. Tramacere I, Virgili G, Perduca V, et al. Adverse effects of immunotherapies for multiple sclerosis: a network meta-analysis. *Cochrane Database Syst Rev* 2023(11) doi: 10.1002/14651858.CD012186.pub2
75. Mocellin S, Goodwin A, Pasquali S. Risk-reducing medications for primary breast cancer: a network meta-analysis. *Cochrane Database Syst Rev* 2019(4) doi: 10.1002/14651858.CD012191.pub2
76. Adams A, Scheckel B, Habsaoui A, et al. Intravenous iron versus oral iron versus no iron with or without erythropoiesis- stimulating agents (ESA) for cancer patients with anaemia: a systematic review and network meta-analysis. *Cochrane Database Syst Rev* 2022(6) doi: 10.1002/14651858.CD012633.pub2
77. Wang R, Danhof NA, Tjon-Kon-Fat RI, et al. Interventions for unexplained infertility: a systematic review and network meta-analysis. *Cochrane Database Syst Rev* 2019(9) doi: 10.1002/14651858.CD012692.pub2
78. Guaiana G, Meader N, Barbui C, et al. Pharmacological treatments in panic disorder in adults: a network meta-analysis. *Cochrane Database Syst Rev* 2023(11) doi: 10.1002/14651858.CD012729.pub3

79. Piechotta V, Adams A, Haque M, et al. Antiemetics for adults for prevention of nausea and vomiting caused by moderately or highly emetogenic chemotherapy: a network meta-analysis. *Cochrane Database Syst Rev* 2021(11) doi: 10.1002/14651858.CD012775.pub2
80. Jakob T, Tesfamariam YM, Macherey S, et al. Bisphosphonates or RANK-ligand-inhibitors for men with prostate cancer and bone metastases: a network meta-analysis. *Cochrane Database Syst Rev* 2020(12) doi: 10.1002/14651858.CD013020.pub2
81. Iogna Prat L, Wilson P, Freeman SC, et al. Antibiotic treatment for spontaneous bacterial peritonitis in people with decompensated liver cirrhosis: a network meta-analysis. *Cochrane Database Syst Rev* 2019(9) doi: 10.1002/14651858.CD013120.pub2
82. Benmassaoud A, Freeman SC, Roccarina D, et al. Treatment for ascites in adults with decompensated liver cirrhosis: a network meta-analysis. *Cochrane Database Syst Rev* 2020(1) doi: 10.1002/14651858.CD013123.pub2
83. Roberts D, Best LMJ, Freeman SC, et al. Treatment for bleeding oesophageal varices in people with decompensated liver cirrhosis: a network meta-analysis. *Cochrane Database Syst Rev* 2021(4) doi: 10.1002/14651858.CD013155.pub2
84. Iheozor-Ejiofor Z, Gordon M, Clegg A, et al. Interventions for maintenance of surgically induced remission in Crohn's disease: a network meta-analysis. *Cochrane Database Syst Rev* 2019(9) doi: 10.1002/14651858.CD013210.pub2
85. Al Said S, Alabed S, Kaier K, et al. Non-vitamin K antagonist oral anticoagulants (NOACs) post-percutaneous coronary intervention: a network meta-analysis. *Cochrane Database Syst Rev* 2019(12) doi: 10.1002/14651858.CD013252.pub2
86. Hanna C, Lawrie TA, Rogozińska E, et al. Treatment of newly diagnosed glioblastoma in the elderly: a network meta-analysis. *Cochrane Database Syst Rev* 2020(3) doi: 10.1002/14651858.CD013261.pub2
87. Imamura M, Scott NW, Wallace SA, et al. Interventions for treating people with symptoms of bladder pain syndrome: a network meta-analysis. *Cochrane Database Syst Rev* 2020(7) doi: 10.1002/14651858.CD013325.pub2
88. Caro P, Turner W, Caldwell DM, Macdonald G. Comparative effectiveness of psychological interventions for treating the psychological consequences of sexual abuse in children and adolescents: a network meta-analysis. *Cochrane Database Syst Rev* 2023(6) doi: 10.1002/14651858.CD013361.pub2
89. Lewis SR, Macey R, Stokes J, et al. Surgical interventions for treating intracapsular hip fractures in older adults: a network meta-analysis. *Cochrane Database Syst Rev* 2022(2) doi: 10.1002/14651858.CD013404.pub2
90. Lewis SR, Macey R, Lewis J, et al. Surgical interventions for treating extracapsular hip fractures in older adults: a network meta-analysis. *Cochrane Database Syst Rev* 2022(2) doi: 10.1002/14651858.CD013405.pub2
91. McBain C, Lawrie TA, Rogozińska E, et al. Treatment options for progression or recurrence of glioblastoma: a network meta-analysis. *Cochrane Database Syst Rev* 2021(1) doi: 10.1002/14651858.CD013579.pub2
92. Franco JVA, Jung JH, Imamura M, et al. Minimally invasive treatments for lower urinary tract symptoms in men with benign prostatic hyperplasia: a network meta-analysis. *Cochrane Database Syst Rev* 2021(7) doi: 10.1002/14651858.CD013656.pub2
93. Walter MA, Nesti C, Spanjol M, et al. Treatment for gastrointestinal and pancreatic neuroendocrine tumours: a network meta-analysis. *Cochrane Database Syst Rev* 2021(11) doi: 10.1002/14651858.CD013700.pub2
94. Hay S, Ovelman C, Zupancic JAF, et al. Systemic corticosteroids for the prevention of bronchopulmonary dysplasia, a network meta-analysis. *Cochrane Database Syst Rev* 2023(8) doi: 10.1002/14651858.CD013730.pub2
95. Oba Y, Anwer S, Patel T, Maduke T, Dias S. Addition of long-acting beta2 agonists or long-acting muscarinic antagonists versus doubling the dose of inhaled corticosteroids (ICS) in

adolescents and adults with uncontrolled asthma with medium dose ICS: a systematic review and network meta-analysis. *Cochrane Database Syst Rev* 2023(8) doi: 10.1002/14651858.CD013797.pub2
96. Aldin A, Besiroglu B, Adams A, et al. First-line therapy for adults with advanced renal cell carcinoma: a systematic review and network meta-analysis. *Cochrane Database Syst Rev* 2023(5) doi: 10.1002/14651858.CD013798.pub2
97. Oba Y, Anwer S, Maduke T, Patel T, Dias S. Effectiveness and tolerability of dual and triple combination inhaler therapies compared with each other and varying doses of inhaled corticosteroids in adolescents and adults with asthma: a systematic review and network meta-analysis. *Cochrane Database Syst Rev* 2022(12) doi: 10.1002/14651858.CD013799.pub2
98. Mitra S, Gardner CE, MacLellan A, et al. Prophylactic cyclo-oxygenase inhibitor drugs for the prevention of morbidity and mortality in preterm infants: a network meta-analysis. *Cochrane Database Syst Rev* 2022(4) doi: 10.1002/14651858.CD013846.pub2
99. Birkinshaw H, Friedrich CM, Cole P, et al. Antidepressants for pain management in adults with chronic pain: a network meta-analysis. *Cochrane Database Syst Rev* 2023(5) doi: 10.1002/14651858.CD014682.pub2
100. Lawrenson JG, Shah R, Huntjens B, et al. Interventions for myopia control in children: a living systematic review and network meta-analysis. *Cochrane Database Syst Rev* 2023(2) doi: 10.1002/14651858.CD014758.pub2
101. Lindson N, Theodoulou A, Ordóñez-Mena JM, et al. Pharmacological and electronic cigarette interventions for smoking cessation in adults: component network meta-analyses. *Cochrane Database Syst Rev* 2023(9) doi: 10.1002/14651858.CD015226.pub2
102. Wilson A, Hodgetts-Morton VA, Marson EJ, et al. Tocolytics for delaying preterm birth: a network meta-analysis (0924). *Cochrane Database Syst Rev* 2022(8) doi: 10.1002/14651858.CD014978.pub2
103. Ghosh J, Papadopoulou A, Devall AJ, et al. Methods for managing miscarriage: a network meta-analysis. *Cochrane Database Syst Rev* 2021(6) doi: 10.1002/14651858.CD012602.pub2
104. Janjua S, Mathioudakis AG, Fortescue R, et al. Prophylactic antibiotics for adults with chronic obstructive pulmonary disease: a network meta-analysis. *Cochrane Database Syst Rev* 2021(1) doi: 10.1002/14651858.CD013198.pub2
105. Rodríguez-Perálvarez M, Guerrero-Misas M, Thorburn D, et al. Maintenance immunosuppression for adults undergoing liver transplantation: a network meta-analysis. *Cochrane Database Syst Rev* 2017(3) doi: 10.1002/14651858.CD011639.pub2
106. Plaz Torres M, Best LMJ, Freeman SC, et al. Secondary prevention of variceal bleeding in adults with previous oesophageal variceal bleeding due to decompensated liver cirrhosis: a network meta-analysis. *Cochrane Database Syst Rev* 2021(3) doi: 10.1002/14651858.CD013122.pub2
107. Roccarina D, Best LMJ, Freeman SC, et al. Primary prevention of variceal bleeding in people with oesophageal varices due to liver cirrhosis: a network meta-analysis. *Cochrane Database Syst Rev* 2021(4) doi: 10.1002/14651858.CD013121.pub2
108. Piechotta V, Jakob T, Langer P, et al. Multiple drug combinations of bortezomib, lenalidomide, and thalidomide for first-line treatment in adults with transplant-ineligible multiple myeloma: a network meta-analysis. *Cochrane Database Syst Rev* 2019(11) doi: 10.1002/14651858.CD013487
109. Best LMJ, Leung J, Freeman SC, et al. Induction immunosuppression in adults undergoing liver transplantation: a network meta-analysis. *Cochrane Database Syst Rev* 2020(1) doi: 10.1002/14651858.CD013203.pub2
110. Scheiman M, Kulp MT, Cotter SA, et al. Interventions for convergence insufficiency: a network meta-analysis. *Cochrane Database Syst Rev* 2020(12) doi: 10.1002/14651858.CD006768.pub3

111. Weibel S, Rücker G, Eberhart LHJ, et al. Drugs for preventing postoperative nausea and vomiting in adults after general anaesthesia: a network meta-analysis. *Cochrane Database Syst Rev* 2020(10) doi: 10.1002/14651858.CD012859.pub2
112. Best LMJ, Freeman SC, Sutton AJ, et al. Treatment for hepatorenal syndrome in people with decompensated liver cirrhosis: a network meta-analysis. *Cochrane Database Syst Rev* 2019(9) doi: 10.1002/14651858.CD013103.pub2
113. Norman G, Westby MJ, Rithalia AD, et al. Dressings and topical agents for treating venous leg ulcers. *Cochrane Database Syst Rev* 2018(6) doi: 10.1002/14651858.CD012583.pub2
114. Burry L, Hutton B, Williamson DR, et al. Pharmacological interventions for the treatment of delirium in critically ill adults. *Cochrane Database Syst Rev* 2019(9) doi: 10.1002/14651858.CD011749.pub2
115. Sawangjit R, Dilokthornsakul P, Lloyd-Lavery A, et al. Systemic treatments for eczema: a network meta-analysis. *Cochrane Database Syst Rev* 2020(9) doi: 10.1002/14651858.CD013206.pub2
116. Devall AJ, Papadopoulou A, Podesek M, et al. Progestogens for preventing miscarriage: a network meta-analysis. *Cochrane Database Syst Rev* 2021(4) doi: 10.1002/14651858.CD013792.pub2
117. Pompoli A, Furukawa TA, Imai H, et al. Psychological therapies for panic disorder with or without agoraphobia in adults: a network meta-analysis. *Cochrane Database Syst Rev* 2016(4) doi: 10.1002/14651858.CD011004.pub2
118. Nevitt SJ, Sudell M, Cividini S, Marson AG, Tudur Smith C. Antiepileptic drug monotherapy for epilepsy: a network meta-analysis of individual participant data. *Cochrane Database Syst Rev* 2022(4) doi: 10.1002/14651858.CD011412.pub4
119. Kew KM, Dias S, Cates CJ. Long-acting inhaled therapy (beta-agonists, anticholinergics and steroids) for COPD: a network meta-analysis. *Cochrane Database Syst Rev* 2014(3) doi: 10.1002/14651858.CD010844.pub2
120. Suijkerbuijk YB, Schaafsma FG, van Mechelen JC, et al. Interventions for obtaining and maintaining employment in adults with severe mental illness, a network meta-analysis. *Cochrane Database Syst Rev* 2017(9) doi: 10.1002/14651858.CD011867.pub2
121. Oba Y, Keeney E, Ghatehorde N, Dias S. Dual combination therapy versus long-acting bronchodilators alone for chronic obstructive pulmonary disease (COPD): a systematic review and network meta-analysis. *Cochrane Database Syst Rev* 2018(12) doi: 10.1002/14651858.CD012620.pub2
122. Gallos ID, Papadopoulou A, Man R, et al. Uterotonic agents for preventing postpartum haemorrhage: a network meta-analysis. *Cochrane Database Syst Rev* 2018(12) doi: 10.1002/14651858.CD011689.pub3
123. de Bastos M, Stegeman BH, Rosendaal FR, et al. Combined oral contraceptives: venous thrombosis. *Cochrane Database Syst Rev* 2014(3) doi: 10.1002/14651858.CD010813.pub2
124. Walsh T, Worthington HV, Glenny AM, Marinho VCC, Jeroncic A. Fluoride toothpastes of different concentrations for preventing dental caries. *Cochrane Database Syst Rev* 2019(3) doi: 10.1002/14651858.CD007868.pub3
125. Komolafe O, Roberts D, Freeman SC, et al. Antibiotic prophylaxis to prevent spontaneous bacterial peritonitis in people with liver cirrhosis: a network meta-analysis. *Cochrane Database Syst Rev* 2020(1) doi: 10.1002/14651858.CD013125.pub2
126. Hetrick SE, McKenzie JE, Bailey AP, et al. New generation antidepressants for depression in children and adolescents: a network meta-analysis. *Cochrane Database Syst Rev* 2021(5) doi: 10.1002/14651858.CD013674.pub2
127. Ernst M, Folkerts AK, Gollan R, et al. Physical exercise for people with Parkinson's disease: a systematic review and network meta-analysis. *Cochrane Database Syst Rev* 2023(1) doi: 10.1002/14651858.CD013856.pub2
