# Supplementary material for: The impact of Cochrane Reviews that apply network meta-analysis in clinical guidelines: A systematic review
Source: PLoS One. 2024 Dec 26;19(12):e0315563. doi: 10.1371/journal.pone.0315563 (PMC11671017; doi:10.1371/journal.pone.0315563)
Supplement: S3 File — (PDF) [file pone.0315563.s005.pdf]

File S3: Matching pair-wise meta-analysis reviews to included network meta-analysis reviews

Included network meta-analysis	Matching process	Matched pair-wise meta-analyses
**Review ID:** CD012602.pub2[1]<br><br>**Review Group:** Pregnancy & Childbirth<br><br>**Date:** 1 June 2021	Matched to the first closest review.	**Review ID:** CD002958.pub2[2]<br><br>**Review Group:** Pregnancy & Childbirth<br><br>**Date:** 08 June 2021
**Review ID:** CD010813.pub2[3]<br><br>**Review Group:** Fertility Regulation<br><br>**Date:** 3 March 2014	Matched to the first closest review.	**Review ID:** CD004112.pub4[4]<br><br>**Review Group:** Fertility Regulation<br><br>**Date:** 30 March 2014
**Review ID:** CD014978.pub2[5]<br><br>**Review Group:** Pregnancy & Childbirth<br><br>**Date:** 10 August 2022	Matched to the first closest review.	**Review ID:** CD006764.pub4[6]<br><br>**Review Group:** Pregnancy & Childbirth<br><br>**Date:** 9 August 2022
**Review ID:** CD012620.pub2[7]<br><br>**Review Group:** Airways<br><br>**Date:** 3 December 2018	Matched to the first closest review.	**Review ID:** CD006922.pub4[8]<br><br>**Review Group:** Airways<br><br>**Date:** 3 December 2018
**Review ID:** CD013210.pub2[9]<br><br>**Review Group:** Gut<br><br>**Date:** 12 September 2019	Matched to the first closest review.	**Review ID:** CD009710.pub3[10]<br><br>**Review Group:** Gut<br><br>**Date:** 4 September 2019
**Review ID:** CD013792.pub2[11]<br><br>**Review Group:** Pregnancy & Childbirth<br><br>**Date:** 19 April 2021	Matched to the first closest review.	**Review ID:** CD009951.pub3[12]<br><br>**Review Group:** Pregnancy & Childbirth<br><br>**Date:** 19 April 2021

Included network meta-analysis	Matching process	Matched pair-wise meta-analyses
**Review ID:** CD011004.pub2[13]<br><br>**Review Group:** Common Mental Disorders<br><br>**Date:** 13 April 2016	Matched to the first closest review.	**Review ID:** CD010204.pub2[14]<br><br>**Review Group:** Common Mental Disorders<br><br>**Date:** 4 April 2016
**Review ID:** CD007868.pub3[15]<br><br>**Review Group:** Oral Health<br><br>**Date:** 4 March 2019	Matched to the first closest review.	**Review ID:** CD010526.pub3[16]<br><br>**Review Group:** Oral Health<br><br>**Date:** 5 March 2019
**Review ID:** CD007419.pub7[17]<br><br>**Review Group:** Eyes & Vision<br><br>**Date:** 27 June 2023	Matched to the first closest review.	**Review ID:** CD010735.pub3[18]<br><br>**Review Group:** Eyes & Vision<br><br>**Date:** 23 June 2023
**Review ID:** CD013674.pub2**[19]**<br><br>**Review Group:** Common Mental Disorders<br><br>**Date:** 24 May 2021	Matched to the first closest review.	**Review ID:** CD011710.pub3[20]<br><br>**Review Group:** Common Mental Disorders<br><br>**Date:** 20 May 2021
**Review ID:** CD011689.pub3[21]<br><br>**Review Group:** Pregnancy & Childbirth<br><br>**Date:** 19 December 2018	Matched to the first closest review.	**Review ID:** CD012203.pub2[22]<br><br>**Review Group:** Pregnancy & Childbirth<br><br>**Date:** 17 December 2018
**Review ID:** CD010590.pub3[23]<br><br>**Review Group:** Kidney & Transplant<br><br>**Date:** 13 February 2023	Matched to the first closest review.	**Review ID:** CD012478.pub2[24]<br><br>**Review Group:** Kidney & Transplant<br><br>**Date:** 22 February 2023
**Review ID:** CD006768.pub3[25]<br><br>**Review Group:** Eyes & Vision<br><br>**Date:** 02 December 2020	Matched to the first closest review.	**Review ID:** CD005656.pub3[26]<br><br>**Review Group:** Eyes & Vision<br><br>**Date:** 17 November 2020

Included network meta-analysis	Matching process	Matched pair-wise meta-analyses
**Review ID:** CD013198.pub2[27]<br><br>**Review Group:** Airways<br><br>**Date:** 15 January 2021	Matched to the first closest review.	**Review ID:** CD013040.pub2[28]<br><br>**Review Group:** Airways<br><br>**Date:** 29 January 2021
**Review ID:** CD013020.pub2[29]<br><br>**Review Group:** Urology<br><br>**Date:** 3 December 2020	Matched to the first closest review.	**Review ID:** CD013245.pub2[30]<br><br>**Review Group:** Urology<br><br>**Date:** 12 December 2020
**Review ID:** CD013252.pub2[31]<br><br>**Review Group:** Heart<br><br>**Date:** 20 December 2019	Matched to the first closest review.	**Review ID:** CD013319.pub2[32]<br><br>**Review Group:** Heart<br><br>**Date:** 20 December 2019
**Review ID:** CD013730.pub2[33]<br><br>**Review Group:** Neonatal<br><br>**Date:** 31 August 2023	Matched to the first closest review.	**Review ID:** CD014806[34]<br><br>**Review Group:** Neonatal<br><br>**Date:** 30 August 2023
**Review ID:** CD012633.pub2[35]<br><br>**Review Group:** Haematology<br><br>**Date:** 20 June 2022	Matched to the first closest review.	**Review ID:** CD015017.pub3[36]<br><br>**Review Group:** Haematology<br><br>**Date:** 21 June 2022
**Review ID:** CD012692.pub2[37]<br><br>**Review Group:** Gynaecology & Fertility<br><br>**Date:** 5 September 2019.	Matched to the first closest review.	**Review ID:** CD012858.pub2[38]<br><br>**Review Group:** Gynaecology & Fertility<br><br>**Date:**.28 August 2019
**Review ID:** CD013656.pub2[39]<br><br>**Review Group:** Urology<br><br>**Date:** 15 July 2021	Matched to the first closest review.	**Review ID:** CD004135.pub4[40]<br><br>**Review Group:** Urology<br><br>**Date:** 28 June 2021

Included network meta-analysis	Matching process	Matched pair-wise meta-analyses
**Review ID:** CD013206.pub2[41]<br><br>**Review Group:** Skin<br><br>**Date:** 14 September 2020	Matched to the first closest review.	**Review ID:** CD004834.pub3[42]<br><br>**Review Group:** Skin<br><br>**Date:** 27 August 2020
**Review ID:** CD011412.pub4[43]<br><br>**Review Group:** Epilepsy<br><br>**Date:** 1 April 2022	Matched to the first closest review.	**Review ID:** CD005612.pub5[44]<br><br>**Review Group:** Epilepsy<br><br>**Date:** 29 March 2022
**Review ID:** CD013700.pub2[45]<br><br>**Review Group:** Gynaecological, Neuro-oncology & Orphan Cancer<br><br>**Date:** 25 November 2021	Matched to the first closest review.	**Review ID:** CD006649.pub8[46]<br><br>**Review Group:** Gynaecological, Neuro-oncology & Orphan Cancer<br><br>**Date:** 08 December 2021
**Review ID:** CD013846.pub2[47]<br><br>**Review Group:** Neonatal<br><br>**Date:** 1 April 2022	Matched to the first closest review.	**Review ID:** CD011027.pub3[48]<br><br>**Review Group:** Neonatal<br><br>**Date:** 18 March 2022
**Review ID:** CD011867.pub2[49]<br><br>**Review Group:** Work<br><br>**Date:** 12 September 2017	Matched to the first closest review.	**Review ID:** CD011899.pub2[50]<br><br>**Review Group:** Work<br><br>**Date:** 30 August 2017
**Review ID:** CD013799.pub2[51]<br><br>**Review Group:** Airways<br><br>**Date:** 6 December 2022	Matched to the first closest review.	**Review ID:** CD013343.pub2[52]<br><br>**Review Group:** Airways<br><br>**Date:** 14 November 2022
**Review ID:** CD012775.pub2[53]<br><br>**Review Group:** Pain, Palliative & Supportive Care	Matched to the first closest review.	**Review ID:** CD013756.pub2[54]<br><br>**Review Group:** Pain, Palliative & Supportive Care

**Included network meta-analysis**	**Matching process**	**Matched pair-wise meta-analyses**
**Date:** 16 November 2021		**Date:** 2 December 2021
**Review ID:** CD014682.pub2[55]<br><br>**Review Group:** Pain, Palliative & Supportive Care<br><br>**Date:** 10 May 2023	Matched to the first closest review.	**Review ID:** CD014915.pub[56]<br><br>**Review Group:** Pain, Palliative & Supportive Care<br><br>**Date:** 5 June 2023
**Review ID:** CD013404.pub2[57]<br><br>**Review Group:** Bone, Joint & Muscle Trauma<br><br>**Date:** 14 February 2022	Matched to the first closest review.	**Review ID:** CD013410.pub2[58]<br><br>**Review Group:** Bone, Joint & Muscle Trauma<br><br>**Date:** 14 February 2022
**Review ID:** CD011639.pub2[59]<br><br>**Review Group:** Hepato-Biliary<br><br>**Date:** 31 March 2017	Matched to the ninth closest review. Closer reviews were network meta-analyses<br>(DOI: 10.1002/14651858.CD011646.pub2;<br>DOI: 10.1002/14651858.CD011640.pub2;<br>DOI: 10.1002/14651858.CD011650.pub2;<br>DOI: 10.1002/14651858.CD011648.pub2;<br>DOI: 10.1002/14651858.CD011343.pub2;<br>DOI: 10.1002/14651858.CD011645.pub2;<br>DOI: 10.1002/14651858.CD011649.pub2;<br>DOI: 10.1002/14651858.CD011647.pub2).	**Review ID:** CD011314.pub2[60]<br><br>**Review Group:** Hepato-Biliary<br><br>**Date: 07** March 2017
**Review ID:** CD013261.pub2[61]<br><br>**Review Group:** Gynaecological, Neuro-oncology & Orphan Cancer<br><br>**Date:** 23 March 2020.	Matched to the second closest review. The first closest review did not use meta-analysis (DOI: 10.1002/14651858.CD013253.pub2).	**Review ID:** CD005004.pub3**[62]**<br>**Review Group:** Gynaecological, Neuro-oncology & Orphan Cancer<br><br>**Date:** 2 March 2020.
**Review ID:** CD011947.pub2[63]<br><br>**Review Group:** Wounds<br><br>**Date:** 22 June 2017.	Matched to the second closest review. The first closest review did not use meta-analysis (DOI: 10.1002/14651858.CD011375.pub2).	**Review ID:** CD011979.pub2[64]<br><br>**Review Group:** Wounds<br><br>**Date:** 28 June 2017.
**Review ID:** CD012583.pub2[65]<br><br>**Review Group:** Wounds	Matched to the second closest review. The first closest review did not use meta-analysis (DOI: 10.1002/14651858.CD011842.pub2).	**Review ID:** CD012522.pub2[66]<br><br>**Review Group:** Wounds

Included network meta-analysis	Matching process	Matched pair-wise meta-analyses
**Date:** 15 June 2018		**Date:** 03 July 2018
**Review ID:** CD010844.pub2[67]<br><br>**Review Group:** Airways<br><br>**Date:** 26 March 2014	Matched to the second closest review. The first closest review used indirect comparisons (DOI: 10.1002/14651858.CD010115.pub2).	**Review ID:** CD009910.pub2[68]<br><br>**Review Group:** Airways<br><br>**Date:** 5 March 2014
**Review ID:** CD013579.pub2[69]<br><br>**Review Group:** Gynaecological, Neuro-oncology & Orphan Cancer<br><br>**Date:** 5 January 2021	Matched to the second closest review. The first closest review used network meta-analysis (DOI: 10.1002/14651858.CD013630.pub2).	**Review ID:** CD012863.pub2[70]<br><br>**Review Group:** Gynaecological, Neuro-oncology & Orphan Cancer<br><br>**Date:** 25 January 2021
**Review ID:** CD013103.pub2[71]<br><br>**Review Group:** Hepato-Biliary<br><br>**Date:** 12 September 2019	Matched to the third closest review. The first closest review used network meta-analysis (DOI: 10.1002/14651858.CD013120.pub2). The second closest review did not use meta-analysis ((DOI: 10.1002/14651858.CD010546.pub2).	**Review ID:** CD013107.pub2[72]<br><br>**Review Group:** Hepato-Biliary<br><br>**Date:** 22 August 2019
**Review ID:** CD013405.pub2[73]<br><br>**Review Group:** Bone, Joint & Muscle Trauma<br><br>**Date:** 10 February 2022	Matched to the third closest review. The first and second closest were an included network meta-analysis (CD013404.pub2) and a review (DOI: 10.1002/14651858.CD013410.pub2) that was already matched to an included network meta-analysis (CD013404.pub2).	**Review ID:** CD000093.pub6[74]<br><br>**Review Group:** Bone, Joint & Muscle Trauma<br><br>**Date:** 26 January 2022
**Review ID:** CD014758.pub2[75]<br><br>**Review Group:** Eyes & Vision<br><br>**Date:** 16 February 2023	Matched to the third closest review. The first closest review was a prognosis review (DOI: 10.1002/14651858.CD013775.pub2). The second closest did not use meta-analysis (DOI: 10.1002/14651858.CD001989.pub3).	**Review ID: C**D012648.pub3[76]<br><br>**Review Group:** Eyes & Vision<br><br>**Date:** 27 January 2023
**Review ID:** CD012859.pub2[77]<br><br>**Review Group:** Anaesthesia	Not matched. No other reviews published within one month (before or after).	NA

Included network meta-analysis	Matching process	Matched pair-wise meta-analyses
**Date**: 19 October 2020		
**Review ID:** CD013798.pub2[78]<br><br>**Review Group:** Urology<br><br>**Date:** 4 May 2023	Not matched. No other reviews published within one month (before or after).	NA
**Review ID:** CD012191.pub2[79]<br><br>**Review Group:** Breast Cancer<br><br>**Date:** 29 April 2019	Not matched. No other reviews published within one month (before or after).	NA
**Review ID:** CD011749.pub2[80]<br><br>**Review Group:** Emergency & Critical Care<br><br>**Date:** 3 September 2019	Not matched. No other reviews published within one month (before or after).	NA
**Review ID:** CD015226.pub2[81]<br><br>**Review Group:** Tobacco Addiction<br><br>**Date**: 12 September 2023	Not matched. No other reviews published within one month (before or after).	NA
**Review ID:** CD013325.pub2[82]<br><br>**Review Group:** Incontinence<br><br>**Date:** 30 July 2020	Not matched. No other reviews published within one month (before or after).	NA
**Review ID:** CD013856.pub2[83]<br><br>**Review Group:** Movement Disorders<br><br>**Date:** 5 January 2023	Not matched. No other reviews published within one month (before or after).	NA
**Review ID:** CD013797.pub2[84]<br><br>**Review Group:** Airways	Not matched. No other reviews published within one month (before or after).	NA

Included network meta-analysis	Matching process	Matched pair-wise meta-analyses
**Date:** 21 August 2023		
**Review ID:** CD012729.pub3[85]<br><br>**Review Group:** Common Mental Disorders<br><br>**Date:** 28 November 2023	Not matched. No other reviews published within one month (before or after).	NA
**Review ID:** CD011381.pub3[86]<br><br>**Review Group:** Multiple Sclerosis & Rare Diseases of the CNS<br><br>**Date:** 4 January 2024	Not matched. No other reviews published within one month (before or after).	NA
**Review ID:** CD012186.pub2[87]<br><br>**Review Group:** Multiple Sclerosis and Rare Diseases of the CNS<br><br>**Date:** 30 November 2023	Not matched. No other reviews published within one month (before or after).	NA
**Review ID:** CD013361.pub2[88]<br><br>**Review Group:** Developmental, Psychosocial & Learning Problems<br><br>**Date:** 5 June 2023	Not matched. One review without meta-analysis published within one month (before or after) (DOI: 10.1002/14651858.CD013851.pub2).	**NA**
**Review ID:** CD011535.pub6[89]<br><br>**Review Group:** Skin<br><br>**Date:** 12 July 2023	Not matched. One review without meta-analysis published within one month (before or after) (DOI: 10.1002/14651858.CD002292.pub4).	NA
**Review ID:** CD013487 [90]<br><br>**Review Group:** Haematology<br><br>**Date:** 25 November 2019	Not matched. One review without meta-analysis published within one month (before or after) (DOI: 10.1002/14651858.CD012745.pub2).	NA

Included network meta-analysis	Matching process	Matched pair-wise meta-analyses
**Review ID:** CD010529.pub3[91]<br><br>**Review Group:** Pain, Palliative & Supportive Care<br><br>**Date:** 21 April 2020	Not matched. One withdrawn review published within one month (before or after) (DOI: 10.1002/14651858.CD007705.pub3).	NA
**Review ID:** CD013123.pub2[92]<br><br>**Review Group:** Hepato-Biliary<br><br>**Date:** 16 January 2020	Not matched. The first and second closest reviews were included network meta-analyses (CD013203.pub2; CD013123.pub2). The third closed review (10.1002/14651858.CD011659.pub2) did not use meta-analysis.<br>The fourth closest review (10.1002/14651858.CD008717.pub3) did not use meta-analysis. The fifth closest review was withdrawn (10.1002/14651858.CD009051.pub3).	NA
**Review ID:** CD013125.pub2[93]<br><br>**Review Group:** Hepato-Biliary<br><br>**Date:** 16 January 2020	Not matched. The first and second closest reviews were included network meta-analyses (CD013203.pub2; CD013123.pub2). The third closed review (10.1002/14651858.CD011659.pub2) did not use meta-analysis.<br>The fourth closest review (10.1002/14651858.CD008717.pub3) did not use meta-analysis. The fifth closest review was withdrawn (10.1002/14651858.CD009051.pub3).	NA
**Review ID:** CD013203.pub2[94]<br><br>**Review Group:** Hepato-Biliary<br><br>**Date:** 16 January 2020	Not matched. The first and second closest reviews were included network meta-analyses (CD013203.pub2; CD013123.pub2). The third closed review (10.1002/14651858.CD011659.pub2) did not use meta-analysis.<br>The fourth closest review (10.1002/14651858.CD008717.pub3) did not use meta-analysis. The fifth closest review was withdrawn (10.1002/14651858.CD009051.pub3).	NA
**Review ID:** CD013120.pub2[95]<br><br>**Review Group:** Hepato-Biliary	Not matched. The first closest review used network meta-analysis (DOI: 10.1002/14651858.CD013103.pub2). The second	NA

Included network meta-analysis	Matching process	Matched pair-wise meta-analyses
**Date:** 16 September 2019	closest review did not use meta-analysis ((DOI: 10.1002/14651858.CD010546.pub2). The third closest review (10.1002/14651858.CD013107.pub2) was already matched to an included network meta-analysis (CD013103.pub2).	
**Review ID:** CD013122.pub2[96]<br><br>**Review Group:** Hepato-Biliary<br><br>**Date:** 30 March 2021	Not matched. Two network meta-analyses and one diagnostic review published within one month (before or after) (DOI: 10.1002/14651858.CD013346.pub2; DOI: 10.1002/14651858.CD013121.pub2; DOI: 10.1002/14651858.CD013155.pub2).	NA
**Review ID:** CD013155.pub2[97]<br><br>**Review Group:** Hepato-Biliary<br><br>**Date:** 10 April 2021	Not matched. Two network meta-analyses and one diagnostic review published within one month (before or after) (DOI: 10.1002/14651858.CD013346.pub2; DOI: 10.1002/14651858.CD013122.pub2; DOI: 10.1002/14651858.CD013121.pub2).	NA
**Review ID:** CD013121.pub2[98]<br><br>**Review Group:** Hepato-Biliary<br><br>**Date:** 6 April 2021	Not matched. Two network meta-analyses and one diagnostic review published within one month (before or after) (DOI: 10.1002/14651858.CD013346.pub2; DOI: 10.1002/14651858.CD013122.pub2; DOI: 10.1002/14651858.CD013155.pub2).	**NA**

References

1. Ghosh J, Papadopoulou A, Devall AJ, et al. Methods for managing miscarriage: a network meta-analysis. *Cochrane Database Syst Rev* 2021(6) doi: 10.1002/14651858.CD012602.pub2
2. Jones E, Stewart F, Taylor B, Davis PG, Brown SJ. Early postnatal discharge from hospital for healthy mothers and term infants. *Cochrane Database Syst Rev* 2021(6) doi: 10.1002/14651858.CD002958.pub2
3. de Bastos M, Stegeman BH, Rosendaal FR, et al. Combined oral contraceptives: venous thrombosis. *Cochrane Database Syst Rev* 2014(3) doi: 10.1002/14651858.CD010813.pub2
4. Cook LA, Pun A, Gallo MF, Lopez LM, Van Vliet H. Scalpel versus no-scalpel incision for vasectomy. *Cochrane Database Syst Rev* 2014(3) doi: 10.1002/14651858.CD004112.pub4
5. Wilson A, Hodgetts-Morton VA, Marson EJ, et al. Tocolytics for delaying preterm birth: a network meta-analysis (0924). *Cochrane Database Syst Rev* 2022(8) doi: 10.1002/14651858.CD014978.pub2

6. Williams MJ, Ramson JA, Brownfoot FC. Different corticosteroids and regimens for accelerating fetal lung maturation for babies at risk of preterm birth. *Cochrane Database Syst Rev* 2022(8) doi: 10.1002/14651858.CD006764.pub4
7. Oba Y, Keeney E, Ghatehorde N, Dias S. Dual combination therapy versus long-acting bronchodilators alone for chronic obstructive pulmonary disease (COPD): a systematic review and network meta-analysis. *Cochrane Database Syst Rev* 2018(12) doi: 10.1002/14651858.CD012620.pub2
8. Cates CJ, Schmidt S, Ferrer M, Sayer B, Waterson S. Inhaled steroids with and without regular salmeterol for asthma: serious adverse events. *Cochrane Database Syst Rev* 2018(12) doi: 10.1002/14651858.CD006922.pub4
9. Iheozor-Ejiofor Z, Gordon M, Clegg A, et al. Interventions for maintenance of surgically induced remission in Crohn’s disease: a network meta-analysis. *Cochrane Database Syst Rev* 2019(9) doi: 10.1002/14651858.CD013210.pub2
10. Peckham EJ, Cooper K, Roberts ER, et al. Homeopathy for treatment of irritable bowel syndrome. *Cochrane Database Syst Rev* 2019(9) doi: 10.1002/14651858.CD009710.pub3
11. Devall AJ, Papadopoulou A, Podesek M, et al. Progestogens for preventing miscarriage: a network meta-analysis. *Cochrane Database Syst Rev* 2021(4) doi: 10.1002/14651858.CD013792.pub2
12. Davidson SJ, Barrett HL, Price SA, Callaway LK, Dekker Nitert M. Probiotics for preventing gestational diabetes. *Cochrane Database Syst Rev* 2021(4) doi: 10.1002/14651858.CD009951.pub3
13. Pompoli A, Furukawa TA, Imai H, et al. Psychological therapies for panic disorder with or without agoraphobia in adults: a network meta-analysis. *Cochrane Database Syst Rev* 2016(4) doi: 10.1002/14651858.CD011004.pub2
14. Roberts NP, Roberts PA, Jones N, Bisson JI. Psychological therapies for post-traumatic stress disorder and comorbid substance use disorder. *Cochrane Database Syst Rev* 2016(4) doi: 10.1002/14651858.CD010204.pub2
15. Walsh T, Worthington HV, Glenny AM, Marinho VCC, Jeroncic A. Fluoride toothpastes of different concentrations for preventing dental caries. *Cochrane Database Syst Rev* 2019(3) doi: 10.1002/14651858.CD007868.pub3
16. Schenkel AB, Veitz-Keenan A. Dental cavity liners for Class I and Class II resin-based composite restorations. *Cochrane Database Syst Rev* 2019(3) doi: 10.1002/14651858.CD010526.pub3
17. Virgili G, Curran K, Lucenteforte E, Peto T, Parravano M. Anti-vascular endothelial growth factor for diabetic macular oedema: a network meta-analysis. *Cochrane Database Syst Rev* 2023(6) doi: 10.1002/14651858.CD007419.pub7
18. Narayan A, Evans JR, O'Brart D, et al. Laser-assisted cataract surgery versus standard ultrasound phacoemulsification cataract surgery. *Cochrane Database Syst Rev* 2023(6) doi: 10.1002/14651858.CD010735.pub3
19. Hetrick SE, McKenzie JE, Bailey AP, et al. New generation antidepressants for depression in children and adolescents: a network meta-analysis. *Cochrane Database Syst Rev* 2021(5) doi: 10.1002/14651858.CD013674.pub2
20. Simon N, Robertson L, Lewis C, et al. Internet-based cognitive and behavioural therapies for post-traumatic stress disorder (PTSD) in adults. *Cochrane Database Syst Rev* 2021(5) doi: 10.1002/14651858.CD011710.pub3
21. Gallos ID, Papadopoulou A, Man R, et al. Uterotonic agents for preventing postpartum haemorrhage: a network meta-analysis. *Cochrane Database Syst Rev* 2018(12) doi: 10.1002/14651858.CD011689.pub3

22. Wojcieszek AM, Shepherd E, Middleton P, et al. Care prior to and during subsequent pregnancies following stillbirth for improving outcomes. *Cochrane Database Syst Rev* 2018(12) doi: 10.1002/14651858.CD012203.pub2
23. Chung EYM, Palmer SC, Saglimbene VM, et al. Erythropoiesis-stimulating agents for anaemia in adults with chronic kidney disease: a network meta-analysis. *Cochrane Database Syst Rev* 2023(2) doi: 10.1002/14651858.CD010590.pub3
24. Briggs VR, Jacques RM, Fotheringham J, et al. Catheter insertion techniques for improving catheter function and clinical outcomes in peritoneal dialysis patients. *Cochrane Database Syst Rev* 2023(2) doi: 10.1002/14651858.CD012478.pub2
25. Scheiman M, Kulp MT, Cotter SA, et al. Interventions for convergence insufficiency: a network meta-analysis. *Cochrane Database Syst Rev* 2020(12) doi: 10.1002/14651858.CD006768.pub3
26. Rittiphairoj T, Mir TA, Li T, Virgili G. Intravitreal steroids for macular edema in diabetes. *Cochrane Database Syst Rev* 2020(11) doi: 10.1002/14651858.CD005656.pub3
27. Janjua S, Mathioudakis AG, Fortescue R, et al. Prophylactic antibiotics for adults with chronic obstructive pulmonary disease: a network meta-analysis. *Cochrane Database Syst Rev* 2021(1) doi: 10.1002/14651858.CD013198.pub2
28. Cox NS, Dal Corso S, Hansen H, et al. Telerehabilitation for chronic respiratory disease. *Cochrane Database Syst Rev* 2021(1) doi: 10.1002/14651858.CD013040.pub2
29. Jakob T, Tesfamariam YM, Macherey S, et al. Bisphosphonates or RANK-ligand-inhibitors for men with prostate cancer and bone metastases: a network meta-analysis. *Cochrane Database Syst Rev* 2020(12) doi: 10.1002/14651858.CD013020.pub2
30. Sathianathen NJ, Oestreich MC, Brown S, et al. Abiraterone acetate in combination with androgen deprivation therapy compared to androgen deprivation therapy only for metastatic hormone-sensitive prostate cancer. *Cochrane Database Syst Rev* 2020(12) doi: 10.1002/14651858.CD013245.pub2
31. Al Said S, Alabed S, Kaier K, et al. Non-vitamin K antagonist oral anticoagulants (NOACs) post-percutaneous coronary intervention: a network meta-analysis. *Cochrane Database Syst Rev* 2019(12) doi: 10.1002/14651858.CD013252.pub2
32. Kolkailah AA, Doukky R, Pelletier MP, et al. Transcatheter aortic valve implantation versus surgical aortic valve replacement for severe aortic stenosis in people with low surgical risk. *Cochrane Database Syst Rev* 2019(12) doi: 10.1002/14651858.CD013319.pub2
33. Hay S, Ovelman C, Zupancic JAF, et al. Systemic corticosteroids for the prevention of bronchopulmonary dysplasia, a network meta-analysis. *Cochrane Database Syst Rev* 2023(8) doi: 10.1002/14651858.CD013730.pub2
34. Yamada J, Bueno M, Santos L, et al. Sucrose analgesia for heel-lance procedures in neonates. *Cochrane Database Syst Rev* 2023(8) doi: 10.1002/14651858.CD014806
35. Adams A, Scheckel B, Habsaoui A, et al. Intravenous iron versus oral iron versus no iron with or without erythropoiesis- stimulating agents (ESA) for cancer patients with anaemia: a systematic review and network meta-analysis. *Cochrane Database Syst Rev* 2022(6) doi: 10.1002/14651858.CD012633.pub2
36. Popp M, Reis S, Schießer S, et al. Ivermectin for preventing and treating COVID-19. *Cochrane Database Syst Rev* 2022(6) doi: 10.1002/14651858.CD015017.pub3

37. Wang R, Danhof NA, Tjon-Kon-Fat RI, et al. Interventions for unexplained infertility: a systematic review and network meta-analysis. *Cochrane Database Syst Rev* 2019(9) doi: 10.1002/14651858.CD012692.pub2
38. van Lieshout LAM, Steenbeek MP, De Hullu JA, et al. Hysterectomy with opportunistic salpingectomy versus hysterectomy alone. *Cochrane Database Syst Rev* 2019(8) doi: 10.1002/14651858.CD012858.pub2
39. Franco JVA, Jung JH, Imamura M, et al. Minimally invasive treatments for lower urinary tract symptoms in men with benign prostatic hyperplasia: a network meta-analysis. *Cochrane Database Syst Rev* 2021(7) doi: 10.1002/14651858.CD013656.pub2
40. Franco JVA, Garegnani L, Escobar Liquitay CM, Borofsky M, Dahm P. Transurethral microwave thermotherapy for the treatment of lower urinary tract symptoms in men with benign prostatic hyperplasia. *Cochrane Database Syst Rev* 2021(6) doi: 10.1002/14651858.CD004135.pub4
41. Sawangjit R, Dilokthornsakul P, Lloyd-Lavery A, et al. Systemic treatments for eczema: a network meta-analysis. *Cochrane Database Syst Rev* 2020(9) doi: 10.1002/14651858.CD013206.pub2
42. Pinart M, Rueda JR, Romero GAS, et al. Interventions for American cutaneous and mucocutaneous leishmaniasis. *Cochrane Database Syst Rev* 2020(8) doi: 10.1002/14651858.CD004834.pub3
43. Nevitt SJ, Sudell M, Cividini S, Marson AG, Tudur Smith C. Antiepileptic drug monotherapy for epilepsy: a network meta-analysis of individual participant data. *Cochrane Database Syst Rev* 2022(4) doi: 10.1002/14651858.CD011412.pub4
44. Panebianco M, Bresnahan R, Marson AG. Pregabalin add-on for drug-resistant focal epilepsy. *Cochrane Database Syst Rev* 2022(3) doi: 10.1002/14651858.CD005612.pub5
45. Walter MA, Nesti C, Spanjol M, et al. Treatment for gastrointestinal and pancreatic neuroendocrine tumours: a network meta-analysis. *Cochrane Database Syst Rev* 2021(11) doi: 10.1002/14651858.CD013700.pub2
46. Kahale LA, Matar CF, Hakoum MB, et al. Anticoagulation for the initial treatment of venous thromboembolism in people with cancer. *Cochrane Database Syst Rev* 2021(12) doi: 10.1002/14651858.CD006649.pub8
47. Mitra S, Gardner CE, MacLellan A, et al. Prophylactic cyclo-oxygenase inhibitor drugs for the prevention of morbidity and mortality in preterm infants: a network meta-analysis. *Cochrane Database Syst Rev* 2022(4) doi: 10.1002/14651858.CD013846.pub2
48. Edwards T, Liu G, Battin M, et al. Oral dextrose gel for the treatment of hypoglycaemia in newborn infants. *Cochrane Database Syst Rev* 2022(3) doi: 10.1002/14651858.CD011027.pub3
49. Suijkerbuijk YB, Schaafsma FG, van Mechelen JC, et al. Interventions for obtaining and maintaining employment in adults with severe mental illness, a network meta-analysis. *Cochrane Database Syst Rev* 2017(9) doi: 10.1002/14651858.CD011867.pub2
50. Kuster AT, Dalsbø TK, Luong Thanh BY, et al. Computer-based versus in-person interventions for preventing and reducing stress in workers. *Cochrane Database Syst Rev* 2017(8) doi: 10.1002/14651858.CD011899.pub2
51. Oba Y, Anwer S, Maduke T, Patel T, Dias S. Effectiveness and tolerability of dual and triple combination inhaler therapies compared with each other and varying doses of inhaled corticosteroids in adolescents and adults with asthma: a systematic review and network meta-analysis. *Cochrane Database Syst Rev* 2022(12) doi: 10.1002/14651858.CD013799.pub2
52. Fraser A, Poole P. Immunostimulants versus placebo for preventing exacerbations in adults with chronic bronchitis or chronic obstructive pulmonary disease. *Cochrane Database Syst Rev* 2022(11) doi: 10.1002/14651858.CD013343.pub2

53. Piechotta V, Adams A, Haque M, et al. Antiemetics for adults for prevention of nausea and vomiting caused by moderately or highly emetogenic chemotherapy: a network meta-analysis. *Cochrane Database Syst Rev* 2021(11) doi: 10.1002/14651858.CD012775.pub2
54. O'Connell NE, Ferraro MC, Gibson W, et al. Implanted spinal neuromodulation interventions for chronic pain in adults. *Cochrane Database Syst Rev* 2021(12) doi: 10.1002/14651858.CD013756.pub2
55. Birkinshaw H, Friedrich CM, Cole P, et al. Antidepressants for pain management in adults with chronic pain: a network meta-analysis. *Cochrane Database Syst Rev* 2023(5) doi: 10.1002/14651858.CD014682.pub2
56. Häuser W, Welsch P, Radbruch L, et al. Cannabis-based medicines and medical cannabis for adults with cancer pain. *Cochrane Database Syst Rev* 2023(6) doi: 10.1002/14651858.CD014915.pub2
57. Lewis SR, Macey R, Stokes J, et al. Surgical interventions for treating intracapsular hip fractures in older adults: a network meta-analysis. *Cochrane Database Syst Rev* 2022(2) doi: 10.1002/14651858.CD013404.pub2
58. Lewis SR, Macey R, Parker MJ, Cook JA, Griffin XL. Arthroplasties for hip fracture in adults. *Cochrane Database Syst Rev* 2022(2) doi: 10.1002/14651858.CD013410.pub2
59. Rodríguez-Perálvarez M, Guerrero-Misas M, Thorburn D, et al. Maintenance immunosuppression for adults undergoing liver transplantation: a network meta-analysis. *Cochrane Database Syst Rev* 2017(3) doi: 10.1002/14651858.CD011639.pub2
60. Abdel-Rahman O, Elsayed Z. External beam radiotherapy for unresectable hepatocellular carcinoma. *Cochrane Database Syst Rev* 2017(3) doi: 10.1002/14651858.CD011314.pub2
61. Hanna C, Lawrie TA, Rogozińska E, et al. Treatment of newly diagnosed glioblastoma in the elderly: a network meta-analysis. *Cochrane Database Syst Rev* 2020(3) doi: 10.1002/14651858.CD013261.pub2
62. Filippini T, Malavolti M, Borrelli F, et al. Green tea (Camellia sinensis) for the prevention of cancer. *Cochrane Database Syst Rev* 2020(3) doi: 10.1002/14651858.CD005004.pub3
63. Westby MJ, Dumville JC, Soares MO, Stubbs N, Norman G. Dressings and topical agents for treating pressure ulcers. *Cochrane Database Syst Rev* 2017(6) doi: 10.1002/14651858.CD011947.pub2
64. Wang HT, Yuan JQ, Zhang B, et al. Phototherapy for treating foot ulcers in people with diabetes. *Cochrane Database Syst Rev* 2017(6) doi: 10.1002/14651858.CD011979.pub2
65. Norman G, Westby MJ, Rithalia AD, et al. Dressings and topical agents for treating venous leg ulcers. *Cochrane Database Syst Rev* 2018(6) doi: 10.1002/14651858.CD012583.pub2
66. Iheozor-Ejiofor Z, Newton K, Dumville JC, et al. Negative pressure wound therapy for open traumatic wounds. *Cochrane Database Syst Rev* 2018(7) doi: 10.1002/14651858.CD012522.pub2
67. Kew KM, Dias S, Cates CJ. Long-acting inhaled therapy (beta-agonists, anticholinergics and steroids) for COPD: a network meta-analysis. *Cochrane Database Syst Rev* 2014(3) doi: 10.1002/14651858.CD010844.pub2
68. Torrego A, Solà I, Munoz AM, et al. Bronchial thermoplasty for moderate or severe persistent asthma in adults. *Cochrane Database Syst Rev* 2014(3) doi: 10.1002/14651858.CD009910.pub2

69. McBain C, Lawrie TA, Rogozińska E, et al. Treatment options for progression or recurrence of glioblastoma: a network meta-analysis. *Cochrane Database Syst Rev* 2021(1) doi: 10.1002/14651858.CD013579.pub2
70. Aue-aungkul A, Kietpeerakool C, Rattanakanokchai S, et al. Postoperative interventions for preventing bladder dysfunction after radical hysterectomy in women with early-stage cervical cancer. *Cochrane Database Syst Rev* 2021(1) doi: 10.1002/14651858.CD012863.pub2
71. Best LMJ, Freeman SC, Sutton AJ, et al. Treatment for hepatorenal syndrome in people with decompensated liver cirrhosis: a network meta-analysis. *Cochrane Database Syst Rev* 2019(9) doi: 10.1002/14651858.CD013103.pub2
72. Kong DZ, Liang N, Yang GL, et al. Acupuncture for chronic hepatitis B. *Cochrane Database Syst Rev* 2019(8) doi: 10.1002/14651858.CD013107.pub2
73. Lewis SR, Macey R, Lewis J, et al. Surgical interventions for treating extracapsular hip fractures in older adults: a network meta-analysis. *Cochrane Database Syst Rev* 2022(2) doi: 10.1002/14651858.CD013405.pub2
74. Lewis SR, Macey R, Gill JR, Parker MJ, Griffin XL. Cephalomedullary nails versus extramedullary implants for extracapsular hip fractures in older adults. *Cochrane Database Syst Rev* 2022(1) doi: 10.1002/14651858.CD000093.pub6
75. Lawrenson JG, Shah R, Huntjens B, et al. Interventions for myopia control in children: a living systematic review and network meta-analysis. *Cochrane Database Syst Rev* 2023(2) doi: 10.1002/14651858.CD014758.pub2
76. Zamora-de La Cruz D, Bartlett J, Gutierrez M, Ng SM. Trifocal intraocular lenses versus bifocal intraocular lenses after cataract extraction among participants with presbyopia. *Cochrane Database Syst Rev* 2023(1) doi: 10.1002/14651858.CD012648.pub3
77. Weibel S, Rücker G, Eberhart LHJ, et al. Drugs for preventing postoperative nausea and vomiting in adults after general anaesthesia: a network meta-analysis. *Cochrane Database Syst Rev* 2020(10) doi: 10.1002/14651858.CD012859.pub2
78. Aldin A, Besiroglu B, Adams A, et al. First-line therapy for adults with advanced renal cell carcinoma: a systematic review and network meta-analysis. *Cochrane Database Syst Rev* 2023(5) doi: 10.1002/14651858.CD013798.pub2
79. Mocellin S, Goodwin A, Pasquali S. Risk-reducing medications for primary breast cancer: a network meta-analysis. *Cochrane Database Syst Rev* 2019(4) doi: 10.1002/14651858.CD012191.pub2
80. Burry L, Hutton B, Williamson DR, et al. Pharmacological interventions for the treatment of delirium in critically ill adults. *Cochrane Database Syst Rev* 2019(9) doi: 10.1002/14651858.CD011749.pub2
81. Lindson N, Theodoulou A, Ordóñez-Mena JM, et al. Pharmacological and electronic cigarette interventions for smoking cessation in adults: component network meta-analyses. *Cochrane Database Syst Rev* 2023(9) doi: 10.1002/14651858.CD015226.pub2
82. Imamura M, Scott NW, Wallace SA, et al. Interventions for treating people with symptoms of bladder pain syndrome: a network meta-analysis. *Cochrane Database Syst Rev* 2020(7) doi: 10.1002/14651858.CD013325.pub2
83. Ernst M, Folkerts AK, Gollan R, et al. Physical exercise for people with Parkinson's disease: a systematic review and network meta-analysis. *Cochrane Database Syst Rev* 2023(1) doi: 10.1002/14651858.CD013856.pub2
84. Oba Y, Anwer S, Patel T, Maduke T, Dias S. Addition of long-acting beta2 agonists or long-acting muscarinic antagonists versus doubling the dose of inhaled corticosteroids (ICS) in adolescents and adults with uncontrolled asthma with medium dose ICS: a systematic review and network meta-analysis. *Cochrane Database Syst Rev* 2023(8) doi: 10.1002/14651858.CD013797.pub2

85. Guaiana G, Meader N, Barbui C, et al. Pharmacological treatments in panic disorder in adults: a network meta-analysis. *Cochrane Database Syst Rev* 2023(11) doi: 10.1002/14651858.CD012729.pub3
86. Gonzalez-Lorenzo M, Ridley B, Minozzi S, et al. Immunomodulators and immunosuppressants for relapsing-remitting multiple sclerosis: a network meta-analysis. *Cochrane Database Syst Rev* 2024(1) doi: 10.1002/14651858.CD011381.pub3
87. Tramacere I, Virgili G, Perduca V, et al. Adverse effects of immunotherapies for multiple sclerosis: a network meta-analysis. *Cochrane Database Syst Rev* 2023(11) doi: 10.1002/14651858.CD012186.pub2
88. Caro P, Turner W, Caldwell DM, Macdonald G. Comparative effectiveness of psychological interventions for treating the psychological consequences of sexual abuse in children and adolescents: a network meta-analysis. *Cochrane Database Syst Rev* 2023(6) doi: 10.1002/14651858.CD013361.pub2
89. Sbidian E, Chaimani A, Guelimi R, et al. Systemic pharmacological treatments for chronic plaque psoriasis: a network meta-analysis. *Cochrane Database Syst Rev* 2023(7) doi: 10.1002/14651858.CD011535.pub6
90. Piechotta V, Jakob T, Langer P, et al. Multiple drug combinations of bortezomib, lenalidomide, and thalidomide for first-line treatment in adults with transplant-ineligible multiple myeloma: a network meta-analysis. *Cochrane Database Syst Rev* 2019(11) doi: 10.1002/14651858.CD013487
91. Dipper A, Jones HE, Bhatnagar R, et al. Interventions for the management of malignant pleural effusions: a network meta-analysis. *Cochrane Database Syst Rev* 2020(4) doi: 10.1002/14651858.CD010529.pub3
92. Benmassaoud A, Freeman SC, Roccarina D, et al. Treatment for ascites in adults with decompensated liver cirrhosis: a network meta-analysis. *Cochrane Database Syst Rev* 2020(1) doi: 10.1002/14651858.CD013123.pub2
93. Komolafe O, Roberts D, Freeman SC, et al. Antibiotic prophylaxis to prevent spontaneous bacterial peritonitis in people with liver cirrhosis: a network meta-analysis. *Cochrane Database Syst Rev* 2020(1) doi: 10.1002/14651858.CD013125.pub2
94. Best LMJ, Leung J, Freeman SC, et al. Induction immunosuppression in adults undergoing liver transplantation: a network meta-analysis. *Cochrane Database Syst Rev* 2020(1) doi: 10.1002/14651858.CD013203.pub2
95. Iogna Prat L, Wilson P, Freeman SC, et al. Antibiotic treatment for spontaneous bacterial peritonitis in people with decompensated liver cirrhosis: a network meta-analysis. *Cochrane Database Syst Rev* 2019(9) doi: 10.1002/14651858.CD013120.pub2
96. Plaz Torres M, Best LMJ, Freeman SC, et al. Secondary prevention of variceal bleeding in adults with previous oesophageal variceal bleeding due to decompensated liver cirrhosis: a network meta-analysis. *Cochrane Database Syst Rev* 2021(3) doi: 10.1002/14651858.CD013122.pub2
97. Roberts D, Best LMJ, Freeman SC, et al. Treatment for bleeding oesophageal varices in people with decompensated liver cirrhosis: a network meta-analysis. *Cochrane Database Syst Rev* 2021(4) doi: 10.1002/14651858.CD013155.pub2
98. Roccarina D, Best LMJ, Freeman SC, et al. Primary prevention of variceal bleeding in people with oesophageal varices due to liver cirrhosis: a network meta-analysis. *Cochrane Database Syst Rev* 2021(4) doi: 10.1002/14651858.CD013121.pub2
