# Supplementary material for: The impact of Cochrane Reviews that apply network meta-analysis in clinical guidelines: A systematic review
Source: PLoS One. 2024 Dec 26;19(12):e0315563. doi: 10.1371/journal.pone.0315563 (PMC11671017; doi:10.1371/journal.pone.0315563)
Supplement: S4 File — (PDF) [file pone.0315563.s006.pdf]

File S4: R code for figures and analyses

R code for Figure 4

```
library(survival)

data2<-read.csv("E:\\data1.csv", header=TRUE)

plot(Surv(data2$time,data2$cens), xlab="Time (years)", ylab="Citation probability", conf.int=FALSE)
```

data1.csv:

time	cens
2	1
0	1
0	1
0	1
0	1
0	1
6	1
0	1
1	1
2	1
1	1
1	1
0	1
3	1
1	1
2	1
2	1
3	1
1	1
3	1
2	1
3	1
2	1
0	1
0	1
0	1
1	0
4	0
1	0
0	0
1	0
7	0
1	0

5	0
2	0
5	0
1	0
3	0
4	0
5	0
4	0
3	0
5	0
5	0
4	0
4	0
1	0
2	0
2	0
3	0
3	0
3	0
1	0
1	0
1	0
2	0
2	0
1	0
1	0
1	0

R code for logistic regression models regarding whether a review was cited in guidelines

```
library(lme4)

events<-c(rep(1,15),rep(0,11),rep(0,9),rep(1,3),rep(1,15),rep(0,11),rep(1,9),rep(0,3))

type<-c(rep("NMA",38), rep("PW",38))

pairid<-rep(c(1:38),2)

type <- relevel(factor(type), ref="PW")

fit<- glm(events~type , family=binomial)   #not matched

fit<- glmer(events~type +(1|pairid), family=binomial)  #matched
```

R code for Poisson regression models regarding the number of times reviews were cited in guidelines

```
library(glm)

numbercites<-c(rep(0,11), rep(0,7), rep(0,2), rep(1,3), rep(1,2), rep(2,1), rep(2,1), rep(2,2),
rep(4,1), rep(5,1), rep(5,1), rep(5,1), rep(5,1), rep(5,1), rep(11,1), rep(12,1), rep(13,1), rep(0,11),
rep(1,7), rep(2,2), rep(1,3), rep(3,2), rep(0,1), rep(1,1), rep(2,2), rep(0,1), rep(0,1), rep(1,1),
rep(2,1), rep(5,1), rep(10,1), rep(2,1), rep(3,1), rep(3,1))

type<-c(rep("NMA",38), rep("PW",38))

pairid<-rep(c(1:38),2)

type <- relevel(factor(type), ref="PW")

fit<- glm(data3$numbercites~type, family=poisson (link=log))  #unmatched

fit<- glmer(numbercites~type +(1|pairid), family=poisson (link=log))  #matched
```

R code for Poisson regression models regarding the number of level one, level two, and level three impacts in guidelines

```
#Level 1

numbercites=c(rep(0,26),rep(0,1),rep(1,2),rep(1,2),rep(2,3),rep(2,1),rep(3,1),rep(3,1),rep(4,1),re
p(0,26),rep(1,1),rep(0,2),rep(1,2),rep(0,3),rep(1,1),rep(0,1),rep(1,1),rep(0,1))

type<-c(rep("NMA",38), rep("PW",38))

pairid<-rep(c(1:38),2)

type <- relevel(factor(type), ref="PW")

fit<- glm(numbercites~type, family=poisson (link=log))  #unmatched

fit<- glmer(numbercites~type +(1|pairid), family=poisson (link=log))  #matched
```

```
#Level 2

numbercites=c(rep(0,21), rep(0,4), rep(1,2), rep(1,3), rep(2,1), rep(2,1), rep(2,1), rep(2,1),
rep(3,2), rep(5,1), rep(7,1), rep(0,21), rep(1,4), rep(0,2), rep(1,3), rep(0,1), rep(1,1), rep(2,1),
rep(5,1), rep(0,2), rep(1,1), rep(0,1))

type<-c(rep("NMA",38), rep("PW",38))

pairid<-rep(c(1:38),2)

type <- relevel(factor(type), ref="PW")

fit<- glm(numbercites~type, family=poisson (link=log))  #unmatched

fit<- glmer(numbercites~type +(1|pairid), family=poisson (link=log))  #matched

#Level 3

numbercites=c(rep(0,20), rep(0,6), rep(0,1), rep(1,2), rep(1,1), rep(1,1), rep(1,1), rep(2,2),
rep(2,1), rep(3,1), rep(4,1), rep(4,1), rep(0,20), rep(1,6), rep(2,1), rep(0,2), rep(1,1), rep(2,1),
rep(4,1), rep(0,2), rep(5,1), rep(2,1), rep(1,1), rep(2,1))

type<-c(rep("NMA",38), rep("PW",38))

pairid<-rep(c(1:38),2)

type <- relevel(factor(type), ref="PW")

fit<- glm(numbercites~type, family=poisson (link=log))  #unmatched

fit<- glmer(numbercites~type +(1|pairid), family=poisson (link=log))  #matched
```

R code for Figure S3 and cox proportional hazards regression model for time to first citation in guidelines

```
library(survival)

data1<-read.csv("C:\\data2.csv", header=TRUE)

attach(data1)

plot(survfit(Surv(time,cens)~type), lty=c(1,2), xlab="Time (years)", ylab="Citation probability")

legend("topright", c("NMA", "PW-MA"), lty = c(1,2))

survdiff(Surv(time,cens)~type)

type <- relevel(factor(data1$type), ref="PW-MA")

fit<-coxph(Surv(time,cens)~type)
```

data2.csv:

type	time	cens
NMA	5	0
NMA	1	0
NMA	1	0
NMA	4	0
NMA	5	0
NMA	1	0
NMA	2	0
NMA	5	0
NMA	3	0
NMA	3	0
NMA	2	0
NMA	2	0
NMA	3	0
NMA	1	0
NMA	2	0
NMA	4	0
NMA	7	0
NMA	3	0
NMA	2	0
NMA	1	0
NMA	2	1
NMA	0	1
NMA	0	1
NMA	2	1
NMA	0	1
NMA	0	1
NMA	6	1
NMA	0	1
NMA	2	1
NMA	4	1
NMA	1	1
NMA	1	1
NMA	1	1
NMA	2	1
NMA	2	1
NMA	2	1
NMA	1	1
NMA	0	1
PW-MA	1	1
PW-MA	1	1
PW-MA	2	1
PW-MA	3	1

PW-MA	2	1
PW-MA	1	1
PW-MA	0	1
PW-MA	4	1
PW-MA	1	1
PW-MA	2	1
PW-MA	1	1
PW-MA	3	1
PW-MA	2	1
PW-MA	1	1
PW-MA	2	1
PW-MA	6	1
PW-MA	5	1
PW-MA	3	1
PW-MA	2	1
PW-MA	2	1
PW-MA	1	1
PW-MA	1	1
PW-MA	0	1
PW-MA	1	1
PW-MA	5	0
PW-MA	1	0
PW-MA	1	0
PW-MA	4	0
PW-MA	1	0
PW-MA	5	0
PW-MA	3	0
PW-MA	3	0
PW-MA	3	0
PW-MA	2	0
PW-MA	1	0
PW-MA	5	0
PW-MA	2	0
PW-MA	7	0
