# Supplementary material for: The impact of Cochrane Reviews that apply network meta-analysis in clinical guidelines: A systematic review
Source: PLoS One. 2024 Dec 26;19(12):e0315563. doi: 10.1371/journal.pone.0315563 (PMC11671017; doi:10.1371/journal.pone.0315563)
Supplement: S1 Table — (PDF) [file pone.0315563.s007.pdf]

Table S1: Data extracted for network meta-analysis reviews that were not cited in guidelines

Review	Eligibility criteria	Analysis
**Review ID:** CD007419.pub7[1]<br><br>**Review Group:** Eyes & Vision<br><br>**Date:** 2023<br><br>**Sources of support:** Azienda Ospedaliero-Universitaria Careggi & University of Florence, Italy; Public Health Agency, UK; Queen's University, Belfast.	**Studies:** RCTs<br><br>**Patients:** People with diabetic macular oedema for whom anti-VEGF treatment is indicated.<br><br>**Interventions analysed for primary outcome:** aflibercept, bevacizumab, brolucizumab, laser, ranibizumab with prompt laser, ranibizumab with deferred laser, ranibizumab (7).<br><br>**Outcome:** change in best-corrected visual acuity between baseline and 24 months (first primary).	**Outcome type and measure:** continuous; mean difference.<br>**Number of trials:** 8<br>**Number of patients:** not reported.<br>**Type of analysis:** Frequentist.<br><br>**Heterogeneity assessed:** Yes (I square, tausq).<br>**Heterogeneity found:** not reported.<br>**Consistency/transitivity assessed:** Yes (inconsistency model, node-splitting).<br>**Inconsistency/intransitivity found:** yes<br><br>**Grade classification:**<br>Very low, low, moderate.<br><br>**ROB (SG/AC):** cannot be extracted for specific analysis but all trials are low or unclear.
**Review ID:** CD010529.pub3[2]<br><br>**Review Group:** Pain, Palliative & Supportive Care<br><br>**Date:** 2020<br><br>**Sources of support:** National Institute for Health Research.	**Studies:** RCTs (including cluster and cross-over).<br><br>**Patients:** adults with malignant pleural effusion<br><br>**Interventions analysed for primary outcome:** interferon, C parvum, tetracycline, bleomycin, talc poudrage, talc slurry, indwelling pleural catheter (IPC) with daily drainage, silver nitrate, thoracoscopic mechanical pleurodesis, autologous blood<br>talc via IPC, viscum, Adriamycin, triethylenephosphoramide, doxycycline, mepacrine, mitoxantrone, mustine, placebo, indwelling pleural catheter, iodine (21).<br><br>**Outcome:** pleurodesis failure rate (first primary).	**Outcome type and measure:** dichotomous; odds ratio.<br>**Number of trials:** 55<br>**Number of patients:** 3758<br>**Type of analysis:** Bayesian and frequentist.<br><br>**Heterogeneity assessed:** Yes (I square, tausq).<br>**Heterogeneity found:** yes.<br>**Consistency/transitivity assessed:** Yes (inconsistency model, loops).<br>**Inconsistency/intransitivity found:** no.<br><br>**Grade classification:** low/moderate.<br><br>**ROB (SG/AC):** cannot be extracted for specific analysis but all trials are low or unclear.
**Review ID:** CD010590.pub3[3]<br><br>**Review Group:** Kidney & Transplant<br><br>**Date:** 2023<br><br>**Sources of support:** not reported.	**Studies:** RCTs<br><br>**Patients:** adults aged 18 years or older with anaemia due to chronic kidney disease.<br><br>**Interventions analysed for primary outcome:** Epoetin alfa, Epoetin beta, Darbepoetin Alfa, Methoxypolyethylene-glycol-epoetin beta Biosimilar Epoetin, Biosimilar Darbepoetin Alfa, Placebo (7).	**Outcome type and measure:** dichotomous; odds ratio.<br>**Number of trials:** 32<br>**Number of patients:** 11197<br>**Type of analysis:** Frequentist.<br><br>**Heterogeneity assessed:** Yes (I square, chi-square, tausq).<br>**Heterogeneity found:** Yes.<br>**Consistency/transitivity assessed:** Yes (inconsistency model, loops).<br>**Inconsistency/intransitivity found:** No.

	**Outcome:** Preventing blood transfusion (first primary).	**Grade classification:** very low/low/moderate.<br><br>**ROB (SG/AC):** low or unclear.
**Review ID:** CD011381.pub3[4]<br><br>**Review Group:** Multiple Sclerosis & Rare Diseases of the CNS<br><br>**Date:** 2024<br><br>**Sources of support:** Multiple Sclerosis International Federation; IRCCS Istituto delle Scienze Neurologiche di Bologna, Italy.	**Studies:** RCTs.<br><br>**Patients:** adults with a diagnosis of relapsing-remitting multiple sclerosis.<br><br>**Interventions analysed for primary outcome:**<br>fingolimod, daclizumab, azathioprine, placebo/no treatment, teriflunomide, mitoxantrone, natalizumab, pegylated interferon beta-1a, interferon beta-1a, interferon beta-1b, interferon beta-1a and 1b, immunoglobulins, glatiramer acetate (13).<br><br>**Outcome:** chance of experiencing one or more relapses over 12 month (first primary).	**Outcome type and measure:** dichotomous; risk ratio.<br>**Number of trials:** 18<br>**Number of patients:** 9310<br>**Type of analysis:** Frequentist.<br><br>**Heterogeneity assessed:** Yes (I square,tausq).<br>**Heterogeneity found:** No.<br>**Consistency/transitivity assessed:** Yes (inconsistency model, loops).<br>**Inconsistency/intransitivity found:** no.<br><br>**Grade classification:** very low/low/moderate/high.<br><br>**ROB (SG/AC):** some high risk trials.
**Review ID:** CD011535.pub6[5]<br><br>**Review Group:** Skin<br><br>**Date:** 2023<br><br>**Sources of support:** National institute for health research; the French Society of Dermatology; French Ministry of Health (French government).	**Studies:** RCTs.<br><br>**Patients:** adults (over 18 years of age) with moderate-to-severe plaque psoriasis (i.e. needed systemic treatment) or psoriatic arthritis whose skin had been clinically diagnosed with moderate-to-severe psoriasis and who were at any stage of treatment.<br><br>**Interventions analysed for primary outcome:** anti-IL17, anti-IL12/23, placebo, small molecules, non-biological conventional systemic agents, anti-TNF alpha, anti-IL23 (7).<br><br>**Outcome:** The proportion of participants who achieved clear or almost clear skin, that is, at least PASI 90 at induction phase (first primary).	**Outcome type and measure:** dichotomous; risk ratio.<br>**Number of trials:** 124<br>**Number of patients:** 51034<br>**Type of analysis:** Frequentist.<br><br>**Heterogeneity assessed:** Yes (I square, chi).<br>**Heterogeneity found:** No<br>**Consistency/transitivity assessed:** Yes (inconsistency model, node splitting).<br>**Inconsistency/intransitivity found:** no.<br><br>**Grade classification:** not reported for all comparisons.<br><br>**ROB (SG/AC):** cannot be extracted for specific analysis but all trials are low or unclear.
**Review ID:** CD011947.pub2[6]<br><br>**Review Group:** Wounds<br><br>**Date:** 2017.<br><br>**Sources of support:** National Institute for Health Research;	**Studies:** RCTs (excluding quasi-RCTs).<br><br>**Patients:** people with a diagnosis of pressure ulcers.<br><br>**Interventions analysed for primary outcome:** 12 eligible dressings (foam, hydrocolloid with or without alginate, hydrocolloid, protease-modulating, iodine-containing, soft polymer, vapour-permeable, silicone-foam combination, sequential hydrocolloid alginate, saline gauze, polyvinylpyrrolidone plus zinc oxide and basic wound contact), six	**Outcome type and measure:** dichotomous; risk ratio.<br>**Number of trials:** 39<br>**Number of patients:** 2116<br>**Type of analysis:** Frequentist.<br><br>**Heterogeneity assessed:** Yes (I square, tau).<br>**Heterogeneity found:** Yes.<br>**Consistency/transitivity assessed:** Yes (compared NMA and direct results).

Division of Nursing, University of Manchester, UK.	topical agents (hydrogel, dextranomer, collagenase ointment, phenytoin, tripeptide copper gel, and sugar plus egg white) (18).<br><br>**Outcome:** proportion with complete healing (first primary).	**Inconsistency/intransitivity found:** Yes.<br><br>**Grade classification:** Very low or low.<br><br>**ROB (SG/AC):** Some trials are high.
**Review ID:** CD012186.pub2[7]<br><br>**Review Group:** Multiple Sclerosis and Rare Diseases of the CNS<br><br>**Date:** 2023<br><br>**Sources of support:** Fondazione I.R.C.C.S. Istituto Neurologico Carlo Besta, Italy (research institute)	**Studies:** RCTs.<br><br>**Patients:** adults with multiple sclerosis OR clinically isolated syndrome<br><br>**Interventions analysed for primary outcome:** interferon beta-1a (avonex), dimethyl fumarate, daclizumab, glatiramer acetate, ocrelizumab, ozanimod, interferon beta-ib, interferon beta-1a, natalizumab, diroximel fumarate, immunoglobulins, p e g - i n t e r f e r o n beta-1a, fingolimod, teriflunomide, cladribine, rituximab, interferons, laquinimod, alemtuzumab, siponimod, ofatumumab, azathioprine, placebo (23).<br><br>**Outcome:** Number of participants with any (one or more) serious adverse events (first primary).	**Outcome type and measure:** dichotomous; risk ratio.<br>**Number of trials:** 84<br>**Number of patients:** 57682<br>**Type of analysis:** Frequentist.<br><br>**Heterogeneity assessed:** Yes (tausq, chi square, I square).<br>**Heterogeneity found:** Yes.<br>**Consistency/transitivity assessed:** Yes (back calculation, Q test).<br>**Inconsistency/intransitivity found:** no.<br><br>**Grade classification:** very low, low, moderate.<br><br>**ROB (SG/AC):** Some high risk trials.
**Review ID:** CD012191.pub2[8]<br><br>**Review Group:** Breast Cancer<br><br>**Date:** 2019<br><br>**Sources of support:** not reported.	**Studies:** RCTs (excluded quasi-RCTs).<br><br>**Patients:** Women with no personal history of breast cancer, but with above average risk of developing breast cancer.<br><br>**Interventions analysed for primary outcome:** Aromatase inhibitors, tamoxifen, placebo (3).<br><br>**Outcome:** Overall breast cancer incidence (first primary).	**Outcome type and measure:** dichotomous; risk ratio.<br>**Number of trials:** 5<br>**Number of patients:** 31256<br>**Type of analysis:** frequentist.<br><br>**Heterogeneity assessed:** Yes (I square)<br>**Heterogeneity found:** Yes.<br><br>**Consistency/transitivity assessed:** Yes (loop specific and design by treatment). Could not be applied.<br>**Inconsistency/intransitivity found:** Not applied.<br><br>**Grade classification:** Low<br>**ROB (SG/AC):** Low.
**Review ID:** CD012633.pub2[9]<br><br>**Review Group:** Haematology<br><br>**Date:** 2022<br><br>**Sources of support:** Federal Ministry of Education and Research, Germany;	**Studies:** RCTs<br><br>**Patients:** patients of any age with solid cancer and/or haematological malignancy undergoing chemotherapy, radiotherapy or no anti-cancer therapy<br><br>**Interventions analysed for primary outcome:**	**Outcome type and measure:** dichotomous; risk ratio.<br>**Number of trials:** 55.<br>**Number of patients:** 15074.<br>**Type of analysis:** Frequentist.<br><br>**Heterogeneity assessed:** Yes (I square, chi-square).<br>**Heterogeneity found:** No.<br>**Consistency/transitivity assessed:** Yes (bucher, Q).<br>**Inconsistency/intransitivity found:** No.

University Hospital Cologne, Germany; Institute of Medical Statistics and Computational Biology, Germany.	erythropoiesis-stimulating agent (ESA) plus IV iron, ESA plus oral iron, ESA without iron, no treatment, placebo +oral iron, placebo, oral iron, IV iron (8).<br><br>**Outcome:** on-study mortality (first primary).	**Grade classification:** low/moderate.<br><br>**ROB (SG/AC):** Cannot extract for specific analysis but all trial are low and unclear.
**Review ID:** CD012692.pub2[10]<br><br>**Review Group:** Gynaecology & Fertility<br><br>**Date:** 2019.<br><br>**Sources of support:** Australian government	**Studies:** RCTs including cross over trials.<br><br>**Patients:** Couples with unexplained infertility.<br><br>**Interventions analysed for primary outcome:**<br>expectant management, ovarian stimulation, intrauterine insemination, in vitro fertilisation, ovarian stimulation and intrauterine insemination (5).<br><br>**Outcome:** Live birth (first primary).	**Outcome type and measure:** dichotomous; odds ratio.<br>**Number of trials:** 10<br>**Number of patients:** 2725<br>**Type of analysis:** frequentist.<br><br>**Heterogeneity assessed:** Yes (Chi-square, I square),<br>**Heterogeneity found:** Yes.<br>**Consistency/transitivity assessed:** Yes (inconsistency models, side splitting).<br>**Inconsistency/intransitivity found:** No.<br><br>**Grade classification:** Low<br>**ROB (SG/AC):** Low and unclear.
**Review ID:** CD012729.pub3[11]<br><br>**Review Group:** Common Mental Disorders<br><br>**Date:** 2023<br><br>**Sources of support:** Western University, Canada; University of Verona, Italy; University of Bristol, UK; University of Toronto, Canada; Kyoto University, Japan; University of Oxford, UK; National Institute for Health and Care Research.	**Studies:** RCTs.<br><br>**Patients:** people with panic disorder diagnosis.<br><br>**Interventions analysed for primary outcome:** placebo, diazepam, alprazolam, clonazepam, escitalopram, fluoxetine, adinazolam, imipramine, paroxetine, venlafaxine, clomipramine, fluvoxamine, citalopram, sertraline, desipramine, buspirone, ritanserin, etizolam, reboxetine, moclobemide (20).<br><br>**Outcome:** response at end of treatment (first primary).	**Outcome type and measure:** dichotomous; risk ratio.<br>**Number of trials:** 48<br>**Number of patients:** 10118<br>**Type of analysis:** Bayesian.<br><br>**Heterogeneity assessed:** Yes (tausq).<br>**Heterogeneity found:** Yes<br>**Consistency/transitivity assessed:** Yes (node splitting).<br>**Inconsistency/intransitivity found:** Yes<br><br>**Grade classification:** threshold analysis.<br><br>**ROB (SG/AC):** cannot be extracted for specific analysis but all trials are low or unclear.
**Review ID:** CD012775.pub2[12]<br><br>**Review Group:** Pain, Palliative & Supportive Care<br><br>**Date:** 2021<br><br>**Sources of support:** German Ministry for Education and Research (German government); University Hospital	**Studies:** RCTs<br><br>**Patients:** adult cancer patients at risk for chemotherapy-induced nausea and vomiting caused by highly emetogenic chemotherapy<br><br>**Interventions analysed for primary outcome:** fosnetupitant with palonosetron, aprepitant with granisetron, granisetron, fosaprepitant with granisetron, fosaprepitant with ondansetron, palonosetron, aprepitant with ondansetron, ondansetron, aprepitant with	**Outcome type and measure:** dichotomous; risk ratio.<br>**Number of trials: 38**.<br>**Number of patients:** 21542.<br>**Type of analysis:** Frequentist.<br><br>**Heterogeneity assessed:** Yes (chi-square, I square).<br>**Heterogeneity found: Yes**.<br>**Consistency/transitivity assessed:** Yes (bucher, Q).<br>**Inconsistency/intransitivity found:** No.

Cologne, Germany; National Institute for Health Research.	palonosetron, aprepitant with ramosetron, casopitant with ondansetron, fosaprepitant with palonosetron, netupitant with palonosetron, rolapitant with ondansetron, rolapitant with granisetron (15).<br><br>**Outcome:** complete control of vomiting overall phase (first primary).	**Grade classification:** High/moderate/low<br><br>**ROB (SG/AC):** Cannot extract for specific analysis but all trials are low and unclear
**Review ID:** CD013020.pub2[13]<br><br>**Review Group:** Urology<br><br>**Date:** 2020<br><br>**Sources of support:** Federal Ministry for Education and Research (German government), Germany	**Studies:** RCTs.<br><br>**Patients:** adults with prostate cancer patients with bone metastases.<br><br>**Interventions analysed for primary outcome:** zoledronic acid, clodronate, risedronate, etidronate, no treatment/placebo (5).<br><br>**Outcome:** Proportion of participants with pain response (first primary).	**Outcome type and measure:** dichotomous; risk ratio.<br>**Number of trials:** 4.<br>**Number of patients:** 1013.<br>**Type of analysis:** Frequentist.<br><br>**Heterogeneity assessed:** Yes (I square, chi-square).<br>**Heterogeneity found:** No. One trial per comparison.<br>**Consistency/transitivity assessed:** Yes (Node-split, global).<br>**Inconsistency/intransitivity found:** Not applied. Not estimable.<br><br>**Grade classification:** moderate.<br><br>**ROB (SG/AC):** Low and unclear.
**Review ID:** CD013120.pub2[14]<br><br>**Review Group:** Hepato-Biliary<br><br>**Date:** 2019<br><br>**Sources of support:** National Institute for Health Research; University of Liverpool, UK; Keele University, UK; North Staffordshire Hospital Trust, UK; Danish State.	**Studies:** RCTs (excluded quasi-RCTs).<br><br>**Patients: A**dult participants with decompensated liver cirrhosis, who are undergoing treatment for spontaneous bacterial peritonitis.<br><br>**Interventions analysed for primary outcome:**<br>Ceftriaxone, cefotaxime, ciprofloxacin, ceftazidime amikacin, cefixime, cefonicid, meropenem + daptomycin, ofloxacin (9).<br><br>**Outcome:** All-cause mortality at maximal follow-up (first primary).	**Outcome type and measure:** time to event; hazard ratio.<br>**Number of trials:** 7<br>**Number of patients:** 458<br>**Type of analysis:** Bayesian.<br><br>**Heterogeneity assessed:** Yes (model comparison, I square)<br>**Heterogeneity found:** No (one trial per comparison).<br>**Consistency/transitivity assessed:** Yes (inconsistency model).<br>**Inconsistency/intransitivity found:** No.<br><br>**Grade classification:** Very low.<br><br>**ROB (high/low/unclear):** Low or unclear.
**Review ID:** CD013123.pub2[15]<br><br>**Review Group:** Hepato-Biliary<br><br>**Date:** 2020<br><br>**Sources of support:** National Institute for Health Research;	**Studies:** RCTs (including cluster and cross-over).<br><br>**Patients:** adults undergoing treatment for ascites with decompensated liver cirrhosis.<br><br>**Interventions analysed for primary outcome:** Aldosterone antagonists (AldoAnt), Systemic vasoconstrictors (Vasocons) + Alb (Albumin), Transjugular intrahepatic portosystemic shunt (TIPS), Paracentesis (Paracen) + Vasocons, Paracen + Reinfusion (Reinf), Paracen + Fluid	**Outcome type and measure:** time to event; hazard ratio.<br>**Number of trials:** 32<br>**Number of patients:** 2448<br>**Type of analysis:** Frequentist/bayesian.<br><br>**Heterogeneity assessed:** Yes (model comparison).<br>**Heterogeneity found:** No.<br>**Consistency/transitivity assessed:** Yes (inconsistency models).<br>**Inconsistency/intransitivity found:** no.

University College London, UK; Danish State.	replacement (Fluid), No treatment, Loop diuretics (LoopD), AldoAnt + LoopD + Systemic vasodilator (Vasodil), AldoAnt + LoopD + Vasocons + Vasodil, AldoAnt + LoopD + Vasocons+ Paracen + fluid, AldoAnt + LoopD + Vasocons, AldoAnt + LoopD + Paracen + fluid, AldoAnt + LoopD + Peritoneovenous shunt (PVShunt), AldoAnt + LoopD + Albumin (Alb), AldoAnt + LoopD (16).<br><br>**Outcome:** All-cause mortality at maximal follow-up (first primary).	**Grade classification**: very low.<br><br>**ROB (SG/AC):** low or unclear.
**Review ID:** CD013155.pub2[16]<br><br>**Review Group:** Hepato-Biliary<br><br>**Date:** 2021<br><br>**Sources of support:** National Institute for Health Research; University College London, UK; Danish State.	**Studies:** RCTs (including cluster and cross-over).<br><br>**Patients:** adults with acutely bleeding oesophageal varices due to decompensated liver cirrhosis<br><br>**Interventions analysed for primary outcome:**<br>sclerotherapy, somatostatin analogues, vasopressin analogues, sclerotherapy plus somatostatin analogues, variceal band ligation, somatostatin analogues plus variceal band ligation, balloon tamponade, nitrates plus vasopressin analogues, no active intervention, sclerotherapy plus variceal band ligation, balloon tamponade plus sclerotherapy, balloon tamponade plus somatostatin analogues, balloon tamponade plus vasopressin analogues, variceal band ligation plus vasopressin analogues, balloon tamponade plus nitrates plus vasopressin analogues, balloon tamponade plus variceal band ligation, portocaval shunt, sclerotherapy plus transjugular intrahepatic portosystemic shunt, sclerotherapy plus vasopressin analogues (19).<br><br>**Outcome:** All-cause mortality (first primary).	**Outcome type and measure:** dichotomous; odds ratio.<br>**Number of trials:** 45.<br>**Number of patients:** 3781<br>**Type of analysis:** Bayesian.<br><br>**Heterogeneity assessed:** Yes (fixed effect and random effect model comparison).<br>**Heterogeneity found:** No.<br>**Consistency/transitivity assessed:** Yes (inconsistency factor plots).<br>**Inconsistency/intransitivity found:** No.<br><br>**Grade classification:** Low/moderate.<br><br>**ROB (SG/AC):** Low and unclear.
**Review ID:** CD013210.pub2[17]<br><br>**Review Group:** Gut<br><br>**Date:** 2019.<br><br>**Sources of support:** Crohn's and Colitis Canada; National Institute for Health Research.	**Studies:** RCTs (excluding quasi-RCTs).<br><br>**Patients: S**urgically induced remission in Crohn's disease.<br><br>**Interventions analysed for primary outcome:** 5-aminosalicylic acid, adalimumab, antibiotics, budesonide, infliximab, probiotics, purine analogues, sulfasalazine, sulfasalazine + prednisolone, placebo (10).<br><br>**Outcome:** Clinical relapse (primary).	**Outcome type and measure:** time to event; hazard ratio.<br>**Number of trials:** 21.<br>**Number of patients:** 2245.<br>**Type of analysis:** Bayesian and frequentist.<br><br>**Heterogeneity assessed:** Yes (Chi-square, I square)<br>**Heterogeneity found:** No.<br>**Consistency/transitivity assessed:** Yes (inconsistency models).<br>**Inconsistency/intransitivity found:** No. 'Marginal.'<br><br>**Grade classification:** low.<br><br>**ROB (SG/AC):** Low and unclear.
**Review ID:** CD013252.pub2[18]	**Studies:** RCTs.	**Outcome type and measure:** dichotomous; risk ratio.<br>**Number of trials:** 5.

**Review Group:** Heart<br><br>**Date: 2019**<br><br>**Sources of support:** National Institute for Health Research.	**Patients:** adults who underwent percutaneous coronary intervention or acute coronary syndrome or chronic coronary syndromes and who have an indication for anticoagulation.<br><br>**Interventions analysed for primary outcome:** vitamin K antagonists, apixaban, rivaroxaban (high dose, 10 mg to 20 mg), rivaroxaban (low dose, 2.5 mg) (4).<br><br>**Outcome:** death from cardiovascular causes (first primary).	**Number of patients:** 8373.<br>**Type of analysis:** Frequentist.<br><br>**Heterogeneity assessed:** Yes (Chi-square, I square).<br>**Heterogeneity found:** Yes.<br>**Consistency/transitivity assessed:** Yes (Node-splitting).<br>**Inconsistency/intransitivity found:** Yes.<br><br>**Grade classification:** Moderate/low/very low.<br><br>**ROB (SG/AC):** Low.
**Review ID:** CD013261.pub2[19]<br><br>**Review Group:** Gynaecological, Neuro-oncology & Orphan Cancer<br><br>**Date:** 2020.<br><br>**Sources of support:** National Institute for Health Research.	**Studies:** RCTs.<br><br>**Patients:** elderly people with newly diagnosed glioblastoma.<br><br>**Interventions analysed for primary outcome:** radiotherapy with 40 Gy in 15 fractions, radiotherapy with 60 Gy in 30 fractions, chemoradiotherapy, chemoradiotherapy plus bevacizumab, radiotherapy plus bevacizumab, temozolomide, supportive care only (7).<br><br>**Outcome: Overall survival** (first primary).	**Outcome type and measure:** time to event; hazard ratio.<br>**Number of trials:** 7.<br>**Number of patients:** 1540.<br>**Type of analysis:** frequentist.<br><br>**Heterogeneity assessed:** Yes (I square).<br>**Heterogeneity found:** Not reported.<br>**Consistency/transitivity assessed:** Yes (node-splitting).<br>**Inconsistency/intransitivity found:** Not applied. No direct and indirect for the same comparison to compare.<br><br>**Grade classification:** Low/moderate.<br><br>**ROB (SG/AC):** Low and unclear.
**Review ID:** CD013325.pub2[20]<br><br>**Review Group:** Incontinence<br><br>**Date:** 2020<br><br>**Sources of support:** National Institute for Health Research; Chief Scientist Office of the Scottish Government Health and Social Care Directorates, UK.	**Studies:** RCTs (including cross-over, and quasi-RCTs).<br><br>**Patients:** adults were specified as having bladder pain syndrome, interstitial cystitis or painful bladder syndrome.<br><br>**Interventions analysed for primary outcome:** behavioural therapy, physical therapy, amino acid, local anaesthetics, antibiotics, antidepressants, antihistamines, calcium channel agonists, chondroitin sulfate, dimethyl sulfoxide, hyaluronic acid, hyperbaric oxygen, immune modulators, phosphodiesterase-5inhibitor, pentosan polysulfate, neuromuscular blockade, anticoagulants + local anaesthetics, pentosan polysulfate + antihistamines, chondroitin sulfate + hyaluronic acid, control (20).<br><br>**Outcome:** Proportion of participants whose symptoms were cured or improved (first primary).	**Outcome type and measure:** dichotomous; odds ratio.<br>**Number of trials:** 43.<br>**Number of patients:** not reported.<br>**Type of analysis:** Bayesian.<br><br>**Heterogeneity assessed:** Yes (I square).<br>**Heterogeneity found:** Yes.<br>**Consistency/transitivity assessed:** Yes (inconsistency models).<br>**Inconsistency/intransitivity found:** Yes.<br><br>**Grade classification:** Very low.<br><br>**ROB (SG/AC):** Not able to extract data.
**Review ID:** CD013361.pub2[21]	**Studies:** RCTs	**Outcome type and measure:** continuous; SMD.

**Review Group:** Developmental, Psychosocial & Learning Problems<br><br>**Date:** 2023<br><br>**Sources of support:** University of Los Andes (Colombia) and the Colombian Administrative Department of Science and Technology (Colombian government).	**Patients:** participants up to 18 years old who had experienced any form of child sexual abuse.<br><br>**Interventions analysed for primary outcome:** cognitive behavioural therapy delivered only to the carer, cognitive behavioural therapy delivered only to the child, cognitive behavioural therapy delivered to the child and the carer, child-centred therapy delivered only to the child, child-centred therapy delivered to the child and the carer, eye movement desensitisation and reprocessing, family therapy, management as usual, waiting list (9).<br><br>**Outcome:** post-traumatic stress disorder (first primary).	**Number of trials:** 11<br>**Number of patients:** 627.<br>**Type of analysis:** Frequentist.<br><br>**Heterogeneity assessed:** Yes (I square, Chi-square).<br>**Heterogeneity found: Yes**.<br>**Consistency/transitivity assessed:** Yes (inconsistency models, loops).<br>**Inconsistency/intransitivity found:** No.<br><br>**Grade classification:** Very low.<br><br>**ROB (SG/AC):** Some trials are high risk.
**Review ID:** CD013404.pub2[22]<br><br>**Review Group:** Bone, Joint & Muscle Trauma<br><br>**Date:** 2022<br><br>**Sources of support:** National Institute for Health Research.	**Studies:** RCTs (including quasi-RCTs).<br><br>**Patients:** older adults (> 60 years of age) with intracapsular hip fractures, most fractures in the included studies were displaced<br><br>**Interventions analysed for primary outcome:** dynamic fixed angle plates, Uncemented first-generation bipolar hemiarthroplasty, Uncemented modern bipolar hemiarthroplasty, cemented modern bipolar hemiarthroplasty, Uncemented first-generation unipolar hemiarthroplasty, uncemented modern unipolar hemiarthroplasty, Total hip arthroplasty (single articulation), Dual-mobility total hip arthroplasty, pins, screws, non-operative treatment, cemented modern unipolar hemiarthroplasty (12).<br><br>**Outcome:** mortality at 12 months (first primary).	**Outcome type and measure:** dichotomous; risk ratio.<br>**Number of trials:** 56<br>**Number of patients:** 9419<br>**Type of analysis:** Frequentist.<br><br>**Heterogeneity assessed:** Yes (chi-square, I square).<br>**Heterogeneity found:** Not reported.<br>**Consistency/transitivity assessed:** Yes (node-splitting).<br>**Inconsistency/intransitivity found:** No.<br><br>**Grade classification:** Very low/low.<br><br>**ROB (SG/AC):** Some high risk trials.
**Review ID:** CD013405.pub2[23]<br><br>**Review Group:** Bone, Joint & Muscle Trauma<br><br>**Date:** 2022<br><br>**Sources of support:** National Institute for Health Research.	**Studies:** RCTs (including quasi-RCTs).<br><br>**Patients:** older adults (> 60 years of age) with extracapsular hip fractures<br><br>**Interventions analysed for primary outcome:** dynamic fixed angle plates, total hip arthroplasty, static fixed angle plates, long cephalomedullary nails, short cephalomedullary nails, condylocephalic nails, external fixation, hemiarthroplasty, non-operative treatment (9).<br><br>**Outcome:** mortality at 12 months (first primary).	**Outcome type and measure:** dichotomous; risk ratio.<br>**Number of trials:** 56<br>**Number of patients:** 8407<br>**Type of analysis:** Frequentist.<br><br>**Heterogeneity assessed:** Yes (I square).<br>**Heterogeneity found:** Not reported.<br>**Consistency/transitivity assessed:** Yes (inconsistency models).<br>**Inconsistency/intransitivity found:** No.<br><br>**Grade classification:** Very low/low.<br><br>**ROB (SG/AC):** Some high risk trials.

**Review ID:** CD013579.pub2[24]<br><br>**Review Group:** Gynaecological, Neuro-oncology & Orphan Cancer<br><br>**Date: 2021**<br><br>**Sources of support:** National Institute for Health Research.	**Studies:** RCTs, quasi-RCTs, nonrandomised studies, and controlled before-and-after studies that included relevant concurrent comparison groups.<br><br>**Patients:** People aged 16 years of age and older diagnosed with recurrent or progressive disease following primary treatment (surgery and chemoradiotherapy) for glioblastoma.<br><br>**Interventions analysed for primary outcome:** bevacizumab (BEV), BEV + lomustine (LOM), LOM, regorafenib (REG), fotemustine (FOM), ABT414 + temozolomide (TMZ), BEV + irinotecan (IRI), BEV + onartuzumab (ONA), cediranib (CED), CED + LOM, ABT414 (11).<br><br>**Outcome:** Overall survival (first primary).	**Outcome type and measure:** time to event; hazard ratio.<br>**Number of trials:** 9.<br>**Number of patients:** 1734.<br>**Type of analysis:** Frequentist.<br><br>**Heterogeneity assessed:** Yes (I square).<br>**Heterogeneity found:** Not reported.<br>**Consistency/transitivity assessed:** Yes (Local/global).<br>**Inconsistency/intransitivity found:** No.<br><br>**Grade classification:** Very low/Low/moderate.<br><br>**ROB (SG/AC):** Low and unclear.
**Review ID:** CD013656.pub2[25]<br><br>**Review Group:** Urology<br><br>**Date:** 2021<br><br>**Sources of support:** National Institute for Health Research; Instituto Universitario Hospital Italiano, Argentina; Yonsei University Wonju College of Medicine, Korea, South; Minneapolis VA Health Care System, USA; University of Minnesota, USA.	**Studies:** RCTs<br><br>**Patients:** men with moderate to severe lower urinary symptoms due to benign prostatic hyperplasia<br><br>**Interventions analysed for main outcome:** prostatic urethral lift, temporary implantable nitinol device, transurethral microwave thermotherapy, transurethral resection of prostate, convective radiofrequency water vapor therapy, prostatic arterial embolization, sham (7).<br><br>**Outcome:** Urological symptom scores (first primary).	**Outcome type and measure:** continuous; mean difference.<br>**Number of trials:** 19<br>**Number of patients:** 1847<br>**Type of analysis:** Frequentist.<br><br>**Heterogeneity assessed:** Yes (Chi-square, I square).<br>**Heterogeneity found:** Yes.<br>**Consistency/transitivity assessed:** Yes (inconsistency factors).<br>**Inconsistency/intransitivity found:** No.<br><br>**Grade classification:** low.<br><br>**ROB (SG/AC):** Some high trials included.
**Review ID:** CD013700.pub2[26]<br><br>**Review Group:** Gynaecological, Neuro-oncology & Orphan Cancer<br><br>**Date:** 2021<br><br>**Sources of support:** National Institute for Health Research	**Studies:** RCTs (including cross-over).<br><br>**Patients:** People of any age with any type and any stage of gastroenteropancreatic neuroendocrine tumours<br><br>**Interventions analysed for primary outcome:** Everolimus, everolimus + somatostatin analogue, Interferon + SSA, Interferon, somatostatin analogues, surufatinib, sunitinib, placebo, dactolisib (9)<br><br>**Outcome:** Disease control after 12 months (first primary).	**Outcome type and measure:** dichotomous; odds ratio.<br>**Number of trials: 9**<br>**Number of patients:** 1757<br>**Type of analysis:** Frequentist<br><br>**Heterogeneity assessed:** Yes (chi-square, I-square).<br>**Heterogeneity found:** No.<br>**Consistency/transitivity assessed:** Yes (node splitting).<br>**Inconsistency/intransitivity found:** Yes<br><br>**Grade classification:** Very low/low/moderate/high<br><br>**ROB (SG/AC):** Low and unclear.
**Review ID:** CD013730.pub2[27]	**Studies:** RCTs	**Outcome type and measure:** dichotomous; risk ratio.

**Review Group:** Neonatal<br><br>**Date:** 2023<br><br>**Sources of support:** Noonan Foundation, USA; Vermont Oxford Network, USA.	**Patients:** preterm infants (< 37 weeks' gestation) with bronchopulmonary dysplasia risk.<br><br>**Interventions analysed for primary outcome:** High-dose dexamethasone, moderate-dose dexamethasone, low-dose dexamethasone, hydrocortisone, placebo/no treatment (5).<br><br>**Outcome:** BPD (at 36 weeks' PMA (first primary).	**Number of trials:** 26.<br>**Number of patients:** 4167.<br>**Type of analysis:** Frequentist.<br><br>**Heterogeneity assessed:** Yes (I square).<br>**Heterogeneity found:** Not reported.<br>**Consistency/transitivity assessed:** Yes (inconsistency models).<br>**Inconsistency/intransitivity found:** Not applied.<br><br>**Grade classification:** low/moderate.<br><br>**ROB (SG/AC):** Low and unclear.
**Review ID:** CD013797.pub2[28]<br><br>**Review Group:** Airways<br><br>**Date:** 2023<br><br>**Sources of support:** National Institute for Health Research.	**Studies:** RCTs<br><br>**Patients:** adolescents (aged 12 years and older) and adults with uncontrolled asthma who had been treated with or were eligible for medium dose-ICS monotherapy.<br><br>**Interventions analysed for primary outcome:** high-dose inhaled corticosteroids (HD-ICS), low-dose inhaled corticosteroids (LD-ICS)/ long-acting beta$_2$-agonist (LABA), medium-dose inhaled corticosteroids (MD-ICS)/ long-acting muscarinic antagonist (LAMA), MD-ICS/LABA, HD-ICS/LABA, MD-ICS (6).<br><br>**Outcome:** severe asthma exacerbation (first primary).	**Outcome type and measure:** time to event; hazard ratio.<br>**Number of trials:** 17.<br>**Number of patients:** 22819.<br>**Type of analysis:** Frequentist and Bayesian.<br><br>**Heterogeneity assessed:** Yes (fixed effect and random effect model comparison, chi-square, I square).<br>**Heterogeneity found:** Yes.<br>**Consistency/transitivity assessed:** Yes (node-splitting).<br>**Inconsistency/intransitivity found:** No.<br><br>**Grade classification:** low/moderate.<br><br>**ROB (SG/AC):** Low.
**Review ID:** CD013798.pub2[29]<br><br>**Review Group:** Urology<br><br>**Date: 2023**<br><br>**Sources of support:** German Federal Ministry of Education and Research (German government); University Hospital Cologne, Germany.	**Studies:** RCTs (including cross-over).<br><br>**Patients:** people with a confirmed diagnosis of advanced renal cell carcinoma (combined risk groups) without previous systemic anticancer therapy.<br><br>**Interventions analysed for primary outcome:** Atezolizumab (ATE), sunitinib (SUN), cabozantinib (CAB), everolimus (EVE), PLACEBO, pazopanib (PAZ), temsirolimus (TEM), interferon (IFN), nivolumab (NIV), ATE + bevacizumab (BEV), TEM+BEV, Lenvatinib (LEN) +PEM, LEN + EVE, NIV + ipilimumab (IPI), PEM + axitinib (AXI), Naptumomab (NAP) + IFN, IFN + BEV, EVE + BEV, IFN +PLACEBO (19).<br><br>**Outcome:** Overall survival (first primary).	**Outcome type and measure:** time to event; hazard ratio.<br>**Number of trials:** 17.<br>**Number of patients:** 9705.<br>**Type of analysis:** Frequentist.<br><br>**Heterogeneity assessed:** Yes (I square, chi-square).<br>**Heterogeneity found:** No.<br>**Consistency/transitivity assessed:** Yes (node splitting, Q, heat plot).<br>**Inconsistency/intransitivity found:** Not reported.<br><br>**Grade classification:** Very low/low/moderate.<br><br>**ROB (SG/AC):** Some high risk trials.
**Review ID:** CD013799.pub2[30]	**Studies:** RCTs	**Outcome type and measure:** time to event; hazard ratio.<br>**Number of trials:** 8

**Review Group:** Airways<br><br>**Date:** 2022<br><br>**Sources of support:** National Institute for Health Research.	**Patients:** adolescents and adults (age 12 years or older) with uncontrolled asthma who had been treated with or were eligible for medium-dose inhaled corticosteroids (MD-ICS)/ long-acting beta$_2$-agonist (LABA) combination therapy<br><br>**Interventions analysed for primary outcome:** High dose **(**HD) ICS/LABA, MD-TRIPLE, HD-TRIPLE, MD-ICS/LABA (4).<br><br>**Outcome:** Asthma severe exacerbations (first primary).	**Number of patients:** 9983<br>**Type of analysis:** Bayesian.<br><br>**Heterogeneity assessed:** Yes (comparing fixed effect and random effect model comparison).<br>**Heterogeneity found:** No.<br>**Consistency/transitivity assessed:** Yes (node splitting).<br>**Inconsistency/intransitivity found:** No.<br><br>**Grade classification:** low/moderate.<br><br>**ROB (SG/AC):** low
**Review ID:** CD013846.pub2[31]<br><br>**Review Group:** Neonatal<br><br>**Date:** 2022<br><br>**Sources of support:** Vermont Oxford Network.	**Studies:** RCTs<br><br>**Patients:** neonates that are preterm (born at less than 37 weeks' completed gestation) or of low birth weight<br><br>**Interventions analysed for primary outcome:** Indomethacin ibuprofen acetaminophen, placebo (4).<br><br>**Outcome:** Severe intraventricular haemorrhage (first primary).	**Outcome type and measure:** dichotomous; risk ratio.<br>**Number of trials:** 23<br>**Number of patients:** 3540<br>**Type of analysis:** Bayesian.<br><br>**Heterogeneity assessed:** Yes (I square).<br>**Heterogeneity found:** Not reported.<br>**Consistency/transitivity assessed:** Yes (inconsistency models, node splitting).<br>**Inconsistency/intransitivity found:** Not applied. Could not run them.<br><br>**Grade classification:** Very low/moderate.<br><br>**ROB (SG/AC):** Cannot extract for specific analysis but all trials are low and unclear.
**Review ID:** CD014682.pub2[32]<br><br>**Review Group:** Pain, Palliative & Supportive Care<br><br>**Date: 2023**<br><br>**Sources of support:** National Institute for Health Research.	**Studies:** RCTs (including cross-over).<br><br>**Patients:** adults with primary or secondary pain in any part of their body (except headache).<br><br>**Interventions analysed for primary outcome:** desvenlafaxine high dose, duloxetine low dose, duloxetine standard dose, duloxetine high dose, esreboxetine standard dose, esreboxetine high dose, milnacipran standard dose, milnacipran dose unable to be categorised, milnacipran high dose, mirtazapine standard dose, amitriptyline dose unable to be categorised, clomipramine standard dose, desvenlafaxine standard dose, esreboxetine dose unable to be categorised, imipramine standard dose, mianserin high dose, imipramine standard dose + pregabalin, venlafaxine standard dose, venlafaxine high dose, venlafaxine dose	**Outcome type and measure:** dichotomous; odds ratio.<br>**Number of trials:** 42<br>**Number of patients:** 14626<br>**Type of analysis:** Bayesian.<br><br>**Heterogeneity assessed:** Yes (chi-square, I square).<br>**Heterogeneity found:** Yes.<br>**Consistency/transitivity assessed:** Yes (inconsistency models, node-splitting).<br>**Inconsistency/intransitivity found:** No.<br><br>**Grade classification:** Very low/low/moderate.<br><br>**ROB (SG/AC):** Cannot extract data for specific analysis but all trials are low and unclear.

	unable to be categorised, carbamazepine, pregabalin, terbutaline, placebo (24).<br><br>**Outcome:** substantial pain relief (≥ 50% reduction in pain intensity from baseline) (first primary).	
**Review ID:** CD014758.pub2[33]<br><br>**Review Group:** Eyes & Vision<br><br>**Date: 2023**<br><br>**Sources of support:** National Institute for Health Research; City University of London, UK.	**Studies:** RCTs<br><br>**Patients:** children with progressive myopia<br><br>**Interventions analysed for primary outcome:** High-dose atropine, moderate-dose atropine, low-dose atropine, pirenzepine, 7-methyxanthine, multifocal soft contact lenses, rigid gas-permeable contact lenses, peripheral plus spectacle lenses, multifocal spectacle lenses, under corrected single vision spectacles, control (single vision spectacles, contact lenses, placebo eyedrops) (11).<br><br>**Outcome: Progression of myopia** (first primary).	**Outcome type and measure:** continuous; mean difference.<br>**Number of trials:** 36<br>**Number of patients:** 2846.<br>**Type of analysis:** Frequentist.<br><br>**Heterogeneity assessed:** Yes (chi-square and I -square).<br>**Heterogeneity found:** Yes.<br>**Consistency/transitivity assessed:** Yes (inconsistency models and node splitting).<br>**Inconsistency/intransitivity found:** No.<br><br>**Grade classification:** Very low/low/moderate.<br><br>**ROB (SG/AC):** Some trials are high risk.
**Review ID:** CD015226.pub2[34]<br><br>**Review Group:** Tobacco Addiction<br><br>**Date:** 2023<br><br>**Sources of support:** National Institute for Health Research; University of Oxford, UK; University of Leicester, UK	**Studies:** RCTs (including factorial and cluster-RCTs).<br><br>**Patients: A**dults who smoke cigarettes.<br><br>**Interventions analysed for primary outcome:** varenicline, cytisine, nicotine patch, nicotine other (fast acting nicotine replacement therapy, nicotine e-cigarette, placebo e-cigarette, bupropion, nortriptyline, nicotine tapering, nicotine patch plus nicotine other, control, placebo (12).<br><br>**Outcome:** smoking cessation at 6 months to 5 years (first primary).	**Outcome type and measure:** dichotomous; odds ratio.<br>**Number of trials:** 299.<br>**Number of patients:** 145460.<br>**Type of analysis:** Bayesian.<br><br>**Heterogeneity assessed:** Yes (prediction intervals).<br>**Heterogeneity found:** No.<br>**Consistency/transitivity assessed:** No.<br>**Inconsistency/intransitivity found:** N/A.<br><br>**Grade classification:** low/moderate/high.<br><br>**ROB (SG/AC):** Not reported.

Abbreviations: AC: allocation concealment; RCT: randomised controlled trial; SG: sequence generation.

1. Virgili G, Curran K, Lucenteforte E, Peto T, Parravano M. Anti-vascular endothelial growth factor for diabetic macular oedema: a network meta-analysis. *Cochrane Database Syst Rev* 2023(6) doi: 10.1002/14651858.CD007419.pub7
2. Dipper A, Jones HE, Bhatnagar R, et al. Interventions for the management of malignant pleural effusions: a network meta-analysis. *Cochrane Database Syst Rev* 2020(4) doi: 10.1002/14651858.CD010529.pub3
3. Chung EYM, Palmer SC, Saglimbene VM, et al. Erythropoiesis-stimulating agents for anaemia in adults with chronic kidney disease: a network meta-analysis. *Cochrane Database Syst Rev* 2023(2) doi: 10.1002/14651858.CD010590.pub3
4. Gonzalez-Lorenzo M, Ridley B, Minozzi S, et al. Immunomodulators and immunosuppressants for relapsing-remitting multiple sclerosis: a network meta-analysis. *Cochrane Database Syst Rev* 2024(1) doi: 10.1002/14651858.CD011381.pub3

5. Sbidian E, Chaimani A, Guelimi R, et al. Systemic pharmacological treatments for chronic plaque psoriasis: a network meta-analysis. *Cochrane Database Syst Rev* 2023(7) doi: 10.1002/14651858.CD011535.pub6
6. Westby MJ, Dumville JC, Soares MO, Stubbs N, Norman G. Dressings and topical agents for treating pressure ulcers. *Cochrane Database Syst Rev* 2017(6) doi: 10.1002/14651858.CD011947.pub2
7. Tramacere I, Virgili G, Perduca V, et al. Adverse effects of immunotherapies for multiple sclerosis: a network meta-analysis. *Cochrane Database Syst Rev* 2023(11) doi: 10.1002/14651858.CD012186.pub2
8. Mocellin S, Goodwin A, Pasquali S. Risk-reducing medications for primary breast cancer: a network meta-analysis. *Cochrane Database Syst Rev* 2019(4) doi: 10.1002/14651858.CD012191.pub2
9. Adams A, Scheckel B, Habsaoui A, et al. Intravenous iron versus oral iron versus no iron with or without erythropoiesis- stimulating agents (ESA) for cancer patients with anaemia: a systematic review and network meta-analysis. *Cochrane Database Syst Rev* 2022(6) doi: 10.1002/14651858.CD012633.pub2
10. Wang R, Danhof NA, Tjon-Kon-Fat RI, et al. Interventions for unexplained infertility: a systematic review and network meta-analysis. *Cochrane Database Syst Rev* 2019(9) doi: 10.1002/14651858.CD012692.pub2
11. Guaiana G, Meader N, Barbui C, et al. Pharmacological treatments in panic disorder in adults: a network meta-analysis. *Cochrane Database Syst Rev* 2023(11) doi: 10.1002/14651858.CD012729.pub3
12. Piechotta V, Adams A, Haque M, et al. Antiemetics for adults for prevention of nausea and vomiting caused by moderately or highly emetogenic chemotherapy: a network meta-analysis. *Cochrane Database Syst Rev* 2021(11) doi: 10.1002/14651858.CD012775.pub2
13. Jakob T, Tesfamariam YM, Macherey S, et al. Bisphosphonates or RANK-ligand-inhibitors for men with prostate cancer and bone metastases: a network meta-analysis. *Cochrane Database Syst Rev* 2020(12) doi: 10.1002/14651858.CD013020.pub2
14. Iogna Prat L, Wilson P, Freeman SC, et al. Antibiotic treatment for spontaneous bacterial peritonitis in people with decompensated liver cirrhosis: a network meta-analysis. *Cochrane Database Syst Rev* 2019(9) doi: 10.1002/14651858.CD013120.pub2
15. Benmassaoud A, Freeman SC, Roccarina D, et al. Treatment for ascites in adults with decompensated liver cirrhosis: a network meta-analysis. *Cochrane Database Syst Rev* 2020(1) doi: 10.1002/14651858.CD013123.pub2
16. Roberts D, Best LMJ, Freeman SC, et al. Treatment for bleeding oesophageal varices in people with decompensated liver cirrhosis: a network meta-analysis. *Cochrane Database Syst Rev* 2021(4) doi: 10.1002/14651858.CD013155.pub2
17. Iheozor-Ejiofor Z, Gordon M, Clegg A, et al. Interventions for maintenance of surgically induced remission in Crohn’s disease: a network meta-analysis. *Cochrane Database Syst Rev* 2019(9) doi: 10.1002/14651858.CD013210.pub2
18. Al Said S, Alabed S, Kaier K, et al. Non-vitamin K antagonist oral anticoagulants (NOACs) post-percutaneous coronary intervention: a network meta-analysis. *Cochrane Database Syst Rev* 2019(12) doi: 10.1002/14651858.CD013252.pub2
19. Hanna C, Lawrie TA, Rogozińska E, et al. Treatment of newly diagnosed glioblastoma in the elderly: a network meta-analysis. *Cochrane Database Syst Rev* 2020(3) doi: 10.1002/14651858.CD013261.pub2
20. Imamura M, Scott NW, Wallace SA, et al. Interventions for treating people with symptoms of bladder pain syndrome: a network meta-analysis. *Cochrane Database Syst Rev* 2020(7) doi: 10.1002/14651858.CD013325.pub2
21. Caro P, Turner W, Caldwell DM, Macdonald G. Comparative effectiveness of psychological interventions for treating the psychological consequences of sexual abuse in children and adolescents: a network meta-analysis. *Cochrane Database Syst Rev* 2023(6) doi: 10.1002/14651858.CD013361.pub2
22. Lewis SR, Macey R, Stokes J, et al. Surgical interventions for treating intracapsular hip fractures in older adults: a network meta-analysis. *Cochrane Database Syst Rev* 2022(2) doi: 10.1002/14651858.CD013404.pub2
23. Lewis SR, Macey R, Lewis J, et al. Surgical interventions for treating extracapsular hip fractures in older adults: a network meta-analysis. *Cochrane Database Syst Rev* 2022(2) doi: 10.1002/14651858.CD013405.pub2

24. McBain C, Lawrie TA, Rogozińska E, et al. Treatment options for progression or recurrence of glioblastoma: a network meta-analysis. *Cochrane Database Syst Rev* 2021(1) doi: 10.1002/14651858.CD013579.pub2
25. Franco JVA, Jung JH, Imamura M, et al. Minimally invasive treatments for lower urinary tract symptoms in men with benign prostatic hyperplasia: a network meta-analysis. *Cochrane Database Syst Rev* 2021(7) doi: 10.1002/14651858.CD013656.pub2
26. Walter MA, Nesti C, Spanjol M, et al. Treatment for gastrointestinal and pancreatic neuroendocrine tumours: a network meta-analysis. *Cochrane Database Syst Rev* 2021(11) doi: 10.1002/14651858.CD013700.pub2
27. Hay S, Ovelman C, Zupancic JAF, et al. Systemic corticosteroids for the prevention of bronchopulmonary dysplasia, a network meta-analysis. *Cochrane Database Syst Rev* 2023(8) doi: 10.1002/14651858.CD013730.pub2
28. Oba Y, Anwer S, Patel T, Maduke T, Dias S. Addition of long-acting beta2 agonists or long-acting muscarinic antagonists versus doubling the dose of inhaled corticosteroids (ICS) in adolescents and adults with uncontrolled asthma with medium dose ICS: a systematic review and network meta-analysis. *Cochrane Database Syst Rev* 2023(8) doi: 10.1002/14651858.CD013797.pub2
29. Aldin A, Besiroglu B, Adams A, et al. First-line therapy for adults with advanced renal cell carcinoma: a systematic review and network meta-analysis. *Cochrane Database Syst Rev* 2023(5) doi: 10.1002/14651858.CD013798.pub2
30. Oba Y, Anwer S, Maduke T, Patel T, Dias S. Effectiveness and tolerability of dual and triple combination inhaler therapies compared with each other and varying doses of inhaled corticosteroids in adolescents and adults with asthma: a systematic review and network meta-analysis. *Cochrane Database Syst Rev* 2022(12) doi: 10.1002/14651858.CD013799.pub2
31. Mitra S, Gardner CE, MacLellan A, et al. Prophylactic cyclo-oxygenase inhibitor drugs for the prevention of morbidity and mortality in preterm infants: a network meta-analysis. *Cochrane Database Syst Rev* 2022(4) doi: 10.1002/14651858.CD013846.pub2
32. Birkinshaw H, Friedrich CM, Cole P, et al. Antidepressants for pain management in adults with chronic pain: a network meta-analysis. *Cochrane Database Syst Rev* 2023(5) doi: 10.1002/14651858.CD014682.pub2
33. Lawrenson JG, Shah R, Huntjens B, et al. Interventions for myopia control in children: a living systematic review and network meta-analysis. *Cochrane Database Syst Rev* 2023(2) doi: 10.1002/14651858.CD014758.pub2
34. Lindson N, Theodoulou A, Ordóñez-Mena JM, et al. Pharmacological and electronic cigarette interventions for smoking cessation in adults: component network meta-analyses. *Cochrane Database Syst Rev* 2023(9) doi: 10.1002/14651858.CD015226.pub2
