# Supplementary material for: The impact of Cochrane Reviews that apply network meta-analysis in clinical guidelines: A systematic review
Source: PLoS One. 2024 Dec 26;19(12):e0315563. doi: 10.1371/journal.pone.0315563 (PMC11671017; doi:10.1371/journal.pone.0315563)
Supplement: S2 Table — (PDF) [file pone.0315563.s008.pdf]

Table S2: Data extracted for network meta-analysis reviews that were cited in guidelines

Review	Eligibility criteria	Analysis
**Review ID:** CD014978.pub2[1]<br><br>**Review Group:** Pregnancy & Childbirth<br><br>**Date:** 2022<br><br>**Sources of support:** National Institute for Health Research; World Health Organization, Switzerland; Birmingham Women's Hospital, UK; Tommy's National Centre for Miscarriage Research, University of Birmingham, UK.	**Studies:** RCTs (including cluster).<br><br>**Patients:** women with live fetus(es), with signs and symptoms of preterm labour.<br><br>**Interventions analysed for primary outcome:** betamimetics, calcium channel blockers, COX inhibitors, magnesium sulphate, nitric oxide donors, oxytocin receptor antagonists, combinations of tocolytics; no treatment or placebo (8).<br><br>**Outcome:** Delay in birth by 48 hours (first primary).	**Outcome type and measure:** dichotomous; risk ratio.<br>**Number of trials:** 86.<br>**Number of patients:** 9853<br>**Type of analysis:** Frequentist.<br><br>**Heterogeneity assessed:** Yes (I square, chi-square).<br>**Heterogeneity found:** Not reported.<br>**Consistency/transitivity assessed:** Yes (inconsistency model, loops).<br>**Inconsistency/intransitivity found:** not reported.<br><br>**Grade classification**: low/moderate.<br><br>**ROB (SG/AC):** cannot be extracted for specific analysis but all trials are low or unclear.
**Review ID:** CD012602.pub2[2]<br><br>**Review Group:** Pregnancy & Childbirth<br><br>**Date:** 2021<br><br>**Sources of support:** National Institute for Health Research; Tommy's National Centre for Miscarriage Research, UK.	**Studies:** RCTs (including cluster and quasi-).<br><br>**Patients:** women with missed miscarriage at T14 weeks gestation<br><br>**Interventions analysed for primary outcome:** suction aspiration, misoprostol, dilation and curettage, mifepristone plus misoprostol, suction aspiration plus cervical preparation, expectant management or placebo (6).<br><br>**Outcome:** Complete miscarriage (first primary).	**Outcome type and measure:** dichotomous; risk ratio.<br>**Number of trials:** ***59***.<br>**Number of patients:** not reported.<br>**Type of analysis:** Frequentist.<br><br>**Heterogeneity assessed:** Yes (I square, chi-square).<br>**Heterogeneity found:** yes<br>**Consistency/transitivity assessed:** Yes (inconsistency model, loops).<br>**Inconsistency/intransitivity found:** no.<br><br>**Grade classification**: low/moderate.<br><br>**ROB (SG/AC):** cannot be extracted for specific analysis but all trials are low or unclear.
**Review ID:** CD013198.pub2[3]<br><br>**Review Group:** Airways<br><br>**Date:** 2021<br><br>**Sources of support:** National Institute for Health Research.	**Studies:** RCTs<br><br>**Patients:** adults who had been diagnosed with COPD.<br><br>**Interventions analysed for primary outcome:** macrolide, tetracycline, quinolone, Comparison: placebo or standard care (4).<br><br>**Outcome:** COPD exacerbation (first primary).	**Outcome type and measure:** time to event; hazard ratio.<br>**Number of trials:** 9.<br>**Number of patients:** 2732<br>**Type of analysis:** Bayesian.<br><br>**Heterogeneity assessed:** Yes (model comparison).<br>**Heterogeneity found:** No.<br>**Consistency/transitivity assessed:** Yes (inconsistency model).<br>**Inconsistency/intransitivity found:** not applied (no loops).<br><br>**Grade classification**: threshold analysis.

		**ROB (SG/AC):** Low or unclear.
**Review ID:** CD011639.pub2[4]<br><br>**Review Group:** Hepato-Biliary<br><br>**Date:** 2017<br><br>**Sources of support:** National Institute for Health Research; University College London, UK; Danish State.	**Studies:** RCTs<br><br>**Patients:** adult participants undergoing liver transplantation (or liver retransplantation).<br><br>**Interventions analysed for primary outcome:** Tacrolimus, Cyclosporine A, Cyclosporine A plus azathioprine, Cyclosporine A plus azathioprine plus glucocorticosteroids, Cyclosporine A plus glucocorticosteroids, Cyclosporine A plus mycophenolate plus glucocorticosteroids, Everolimus, Tacrolimus plus azathioprine, Tacrolimus plus everolimus, Tacrolimus plus glucocorticosteroids, Tacrolimus plus mycophenolate plus glucocorticosteroids, Tacrolimus plus sirolimus (12).<br><br>**Outcome:** Mortality at maximal follow-up (first primary).	**Outcome type and measure:** time to event; hazard ratio.<br>**Number of trials:** 21.<br>**Number of patients:** 3492<br>**Type of analysis:** Bayesian and frequentist.<br><br>**Heterogeneity assessed:** Yes (model comparison).<br>**Heterogeneity found:** Not reported.<br>**Consistency/transitivity assessed:** Yes (inconsistency model).<br>**Inconsistency/intransitivity found:** no.<br><br>**Grade classification**: very low/low.<br><br>**ROB (SG/AC):** Low or unclear.
**Review ID:** CD013122.pub2[5]<br><br>**Review Group:** Hepato-Biliary<br><br>**Date:** 2021<br><br>**Sources of support:** National Institute for Health Research; Danish State.	**Studies:** RCTs (including cluster and cross-over).<br><br>**Patients:** adults with a history of oesophageal varices due to decompensated liver cirrhosis undergoing treatment for the prevention of rebleeding.<br><br>**Interventions analysed for primary outcome:** beta-blockers plus sclerotherapy, beta-blockers plus nitrates plus variceal band ligation, beta-blockers plus nitrates, beta-blockers, variceal band ligation, tips, sclerotherapy plus variceal band ligation, sclerotherapy plus nitrates, sclerotherapy, pc shunt, no active intervention, beta-blockers plus variceal band ligation (12).<br><br>**Outcome:** All-cause mortality at maximal follow-up (first primary).	**Outcome type and measure:** time to event; hazard ratio.<br>**Number of trials:** 42<br>**Number of patients:** 3369<br>**Type of analysis:** Frequentist/Bayesian.<br><br>**Heterogeneity assessed:** Yes (model comparison).<br>**Heterogeneity found:** No.<br>**Consistency/transitivity assessed:** Yes (inconsistency model).<br>**Inconsistency/intransitivity found:** Yes.<br><br>**Grade classification**: low.<br><br>**ROB (SG/AC):** low or unclear.
**Review ID:** CD013121.pub2[6]<br><br>**Review Group:** Hepato-Biliary<br><br>**Date:** 2021	**Studies:** RCTs (including cluster and cross-over).<br><br>**Patients:** adults with oesophageal varices due to liver cirrhosis undergoing treatment for the prevention of first variceal bleeding.	**Outcome type and measure:** dichotomous; odds ratio; and time to event; hazard ratio.<br>**Number of trials:** 57<br>**Number of patients:** 5911<br>**Type of analysis:** Bayesian<br><br>**Heterogeneity assessed:** Yes (model comparison).

**Sources of support:** National Institute for Health Research; University College London, UK; Danish State.	**Interventions analysed for primary outcome:** beta-blockers + sclerotherapy, beta-blockers + nitrates, beta-blockers, Variceal band ligation, Sclerotherapy, portocaval shunt, no active intervention, nitrates, betablockers + Variceal band ligation (9).<br><br>**Outcome:** All-cause mortality at maximal follow-up (first primary).	**Heterogeneity found:** Fixed-effect model not applied so not assessed.<br>**Consistency/transitivity assessed:** Yes (inconsistency model).<br>**Inconsistency/intransitivity found:** No.<br><br>**Grade classification**: low/very low.<br><br>**ROB (SG/AC):** low or unclear.
**Review ID:** CD013487[7]<br><br>**Review Group:** Haematology<br><br>**Date:** 2019<br><br>**Sources of support:** National Institute for Health Research; University Hospital Cologne, Germany; NHS Blood and Transplant, UK.	**Studies:** RCTs<br><br>**Patients:** newly diagnosed, transplant-ineligible adults with symptomatic multiple myeloma<br><br>**Interventions analysed for primary outcome:** lenalidomide plus dexamethasone (RD), thalidomide plus melphalan and prednisone (TMP), bortezomib plus melphalan and prednisone (VMP), continuous bortezomib plus lenalidomide plus dexamethasone (VRDc), melphalan and prednisone (MP), VTMPc, RDc, RCPc, RMPc, TMPc, TCD, MPc, RCD, RMP, TDc (15).<br><br>**Outcome:** Overall survival (first primary).	**Outcome type and measure:** time to event; hazard ratio.<br>**Number of trials:** 19<br>**Number of patients:** not reported<br>**Type of analysis:** Frequentist.<br><br>**Heterogeneity assessed:** Yes (I square).<br>**Heterogeneity found:** Yes.<br>**Consistency/transitivity assessed:** Yes (node-split, global Q).<br>**Inconsistency/intransitivity found:** not reported.<br><br>**Grade classification**: low/moderate.<br><br>**ROB (SG/AC):** cannot be extracted for specific analysis but all trials are low or unclear.
**Review ID:** CD013203.pub2[8]<br><br>**Review Group:** Hepato-Biliary<br><br>**Date:** 2020<br><br>**Sources of support:** National Institute for Health Research; University College London, UK; Danish State.	**Studies:** RCTs (including cluster and cross-over).<br><br>**Patients:** adult patients undergoing liver transplantation.<br><br>**Interventions analysed for primary outcome:** Basiliximab+Steriods, Basiliximab, Anti-thymocyte globulin + steroids, Anti-thymocyte globulin, steroids, no intervention, Daclizumab + steroids, Daclizumab (8).<br><br>**Outcome:** All-cause mortality at maximal follow-up (first primary).	**Outcome type and measure:** time to event; hazard ratio.<br>**Number of trials:** 21.<br>**Number of patients:** 2928<br>**Type of analysis:** Frequentist/bayesian.<br><br>**Heterogeneity assessed:** Yes (model comparison).<br>**Heterogeneity found:** No.<br>**Consistency/transitivity assessed:** Yes (inconsistency model, loops).<br>**Inconsistency/intransitivity found:** no.<br><br>**Grade classification**: low/very low.<br><br>**ROB (SG/AC):** low or unclear.
**Review ID:** CD006768.pub3[9]<br><br>**Review Group:** Eyes & Vision<br><br>**Date:** 2020	**Studies:** RCTs<br><br>**Patients:** participants had been treated for convergence insufficiency using non-surgical interventions. Details given for	**Outcome type and measure:** dichotomous; risk ratio.<br>**Number of trials:** 4<br>**Number of patients:** 737<br>**Type of analysis:** Frequentist.

**Sources of support:** National Institute for Health Research; Johns Hopkins Bloomberg School of Public Health, USA.	NA of children here (first reported) but also performed an NMA of adults.<br><br>**Interventions analysed for primary outcome:** office-based vergence/accommodative therapy with home reinforcement; home-based pencil/target push-ups; home-based computer vergence/accommodative therapy; placebo vergence/accommodative therapy or other placebo intervention (4).<br><br>**Outcome:** Composite convergence outcome: achieved normal and improved NPC and PFV (first primary).	**Heterogeneity assessed:** Yes (I square, chi-square).<br>**Heterogeneity found:** Not reported.<br>**Consistency/transitivity assessed:** Yes (loops).<br>**Inconsistency/intransitivity found:** not reported.<br><br>**Grade classification**: low/moderate/high.<br><br>**ROB (SG/AC):** low.
**Review ID:** CD012859.pub2[10]<br><br>**Review Group:** Anaesthesia<br><br>**Date:** 2020<br><br>**Sources of support:** Braun-Stifung, Germany; National Institute for Health Research; University of Würzburg, Germany; University of Utah, USA; University of Freiburg, Germany; Queen Elizabeth University Hospital, UK; Sapienza University of Rome, Italy; University Hospital Düsseldorf, Germany; Philipps-University Marburg, Germany.	**Studies:** RCTs<br><br>**Patients:** adult participants undergoing any type of surgery under general anaesthesia<br><br>**Interventions analysed for primary outcome:** aliz, amis, apre, apre-dexa, apre-dexa-onda, apre-dexa-prom, apre-onda, apre-palo, apre-ramo, apre-scop, beta, busp, caso, caso-onda, cp12, cp12-onda, cycl, dexa, dexa-dola, dexa-drop, dexa-drop-meto-onda, dexa-drop-onda, dexa-gran, dexa-halo, dexa-meto, dexa-meto-onda, dexa-onda, dexa-onda-prom, dexa-palo, dexa-prom, dexa-ramo, dexa-scop, dexa-trop, dime, dixy, dola, dola-drop, domp, drop, drop-gran, drop-onda, drop-palo, drop-trop, fosa, gran, halo, halo-onda, mecl-onda, meth-trop, meto, meto-onda, meto-prom, onda, onda-prom, onda-scop, onda-vest, palo, pene, pene-trop, plac, pred, proc, prom, ramo, rola, scop, trop (65 or 67).<br><br>**Outcome:** Vomiting (or dry retching) within 24 hours (first primary).	**Outcome type and measure:** dichotomous; risk ratio.<br>**Number of trials:** 282<br>**Number of patients:** 50812<br>**Type of analysis:** Frequentist.<br><br>**Heterogeneity assessed:** Yes (I square, chi-square).<br>**Heterogeneity found:** Yes.<br>**Consistency/transitivity assessed:** Yes (inconsistency model).<br>**Inconsistency/intransitivity found:** Yes,<br><br>**Grade classification**: high/moderate.<br><br>**ROB (SG/AC):** Some are high risk.
**Review ID:** CD013103.pub2[11]<br><br>**Review Group:** Hepato-Biliary<br><br>**Date:** 2019	**Studies:** RCTs<br><br>**Patients:** adult trial participants undergoing treatment for hepatorenal syndrome with decompensated liver cirrhosis.<br><br>**Interventions analysed for primary outcome:** albumin plus noradrenaline, albumin, albumin plus midodrine	**Outcome type and measure:** time to event; hazard ratio.<br>**Number of trials:** 19.<br>**Number of patients:** 1089<br>**Type of analysis:** Frequentist/bayesian.<br><br>**Heterogeneity assessed:** Yes (I square, model comparison).<br>**Heterogeneity found:** Yes.

**Sources of support:** National Institute for Health Research; University College London, UK; Danish State.	plusoctreotide, albumin plus midodrine plusoctreotide plus pentoxifylline, albumin plus octreotide, albumin plus terlipressin (6).<br><br>**Outcome:** All-cause mortality at maximal follow-up (first primary).	**Consistency/transitivity assessed:** Yes (inconsistency model; loops).<br>**Inconsistency/intransitivity found:** no.<br><br>**Grade classification**: very low.<br><br>**ROB (SG/AC):** low or unclear.
**Review ID:** CD012583.pub2[12]<br><br>**Review Group:** Wounds<br><br>**Date:** 2018<br><br>**Sources of support:** National Institute for Health Research; University of Manchester, UK.	**Studies:** RCTs (including cross-over).<br><br>**Patients:** adults described as having venous leg ulcers.<br><br>**Interventions analysed for primary outcome:** 12 dressings (foam, hydrocolloid, hydrofibre, alginate, ibuprofen-releasing foam, nonadherent, paste bandage, protease-modulating (PMM), PMM-silver, silver-containing, film, saline gauze); 10 topical agents (hydrogel, cadexomer iodine, gentian violet, hyaluronic acid, hyaluronic-acid with povidone iodine, octenidine, povidone iodine, silver sulfadiazine (SSD), sucralfate andzinc oxide), and 3 other (blood product, emollient cream, and growth factor) (25).<br><br>**Outcome:** proportion with complete healing (first primary).	**Outcome type and measure:** dichotomous; risk ratio.<br>**Number of trials:** 59<br>**Number of patients:** 5156<br>**Type of analysis:** Frequentist.<br><br>**Heterogeneity assessed:** Yes (I square).<br>**Heterogeneity found:** Yes..<br>**Consistency/transitivity assessed:** Yes (node-splitting, loops).<br>**Inconsistency/intransitivity found:** yes.<br><br>**Grade classification**: very low/low/moderate.<br><br>**ROB (SG/AC):** some high risk studies.
**Review ID:** CD011749.pub2[13]<br><br>**Review Group:** Emergency & Critical Care<br><br>**Date:** 2019<br><br>**Sources of support:** Mount Sinai Hospital, Canada; Canadian Institutes of Health Research.	**Studies:** RCTs (including quasi-).<br><br>**Patients:** critically ill adults with confirmed or at high risk of delirium<br><br>**Interventions analysed for primary outcome:** typical antipsychotic, atypical antipsychotic, statin (hmgcoa), alpha2 agonist, cholinesterase inhibitor, opioid, placebo (7).<br><br>**Outcome:** Duration of delirium (first primary).	**Outcome type and measure:** conitnuous; ratio of means.<br>**Number of trials:** 11<br>**Number of patients:** 1530<br>**Type of analysis:** Bayesian and frequentist<br><br>**Heterogeneity assessed:** Yes (I square, chi-square, model comparison).<br>**Heterogeneity found:** Yes.<br>**Consistency/transitivity assessed:** Yes (inconsistency model).<br>**Inconsistency/intransitivity found:** no<br><br>**Grade classification**: very low/moderate/high<br><br>**ROB (SG/AC):** low and unclear.
**Review ID:** CD013206.pub2[14]<br><br>**Review Group:** Skin<br><br>**Date:** 2020	**Studies:** RCTs (including cross-over).<br><br>**Patients:** patients with moderate to severe eczema.	**Outcome type and measure:** dichotomous; risk ratio.<br>**Number of trials:** 14<br>**Number of patients:** 3851<br>**Type of analysis:** Frequentist.

**Sources of support:** Mahasarakham University, Thailand; National Institute for Health Research.	**Interventions analysed for primary outcome:** Dupilumab, Tralokinumab, Tezepilumab, GBR830, Lebrikizumab, ASN002, Ustekinumab, placebo, (8).<br><br>**Outcome:** Proportions of participants who achieved EASI75 (achieved 75% improvement in EASI score) at short-term (≤ 16 weeks) (first primary).	**Heterogeneity assessed:** Yes (I square, chi-square).<br>**Heterogeneity found:** yes.<br>**Consistency/transitivity assessed:** Yes (inconsistency model).<br>**Inconsistency/intransitivity found:** no.<br><br>**Grade classification**: very low/low/high.<br><br>**ROB (SG/AC):** cannot be extracted for specific analysis but all trials are low or unclear.
**Review ID:** CD013792.pub2[15]<br><br>**Review Group:** Pregnancy & Childbirth<br><br>**Date:** 2021<br><br>**Sources of support:** National Institute for Health Research; Tommy's National Centre for Miscarriage Research, University of Birmingham, UK; Tommy's Charity, UK.	**Studies:** RCTs (including cluster).<br><br>**Patients:** omen with threatened miscarriage or a history of recurrent miscarriage.<br><br>**Interventions analysed for primary outcome:** Vaginal micronized progesterone, dydrogesterone, placebo (3).<br><br>**Outcome:** live birth hours (first primary).	**Outcome type and measure:** dichotomous; risk ratio.<br>**Number of trials:** 3.<br>**Number of patients:** 4496<br>**Type of analysis:** Frequentist.<br><br>**Heterogeneity assessed:** Yes (I square).<br>**Heterogeneity found:** No<br><br>**Consistency/transitivity assessed:** Yes (inconsistency model, loops).<br>**Inconsistency/intransitivity found:** not applied.<br><br>**Grade classification**: high/moderate.<br><br>**ROB (SG/AC):** cannot be extracted for specific analysis but all trials are low or unclear.
**Review ID:** CD011004.pub2[16]<br><br>**Review Group:** Common Mental Disorders<br><br>**Date:** 2016<br><br>**Sources of support:** not reported	**Studies:** RCTs (including cluster and cross over).<br><br>**Patients:** adult patients with panic disorder with or without agoraphobia<br><br>**Interventions analysed for primary outcome:** behaviour therapy, cognitive training, psychodynamic therapies, physiological therapies, supportive psychotherapy, wait list, no treatment, cognitive behaviour therapy (8).<br><br>**Outcome:** short term remission (first primary).	**Outcome type and measure:** dichotomous; odds ratio.<br>**Number of trials:** 40<br>**Number of patients:** 2491<br>**Type of analysis:** Frequentist.<br><br>**Heterogeneity assessed:** Yes (I square, chi-square).<br>**Heterogeneity found:** Yes.<br>**Consistency/transitivity assessed:** Yes (inconsistency model, loops).<br>**Inconsistency/intransitivity found:** no.<br><br>**Grade classification**: low/very low.<br><br>**ROB (SG/AC):** cannot be extracted for specific analysis but all trials are low or unclear.
**Review ID:** CD011412.pub4[17]<br><br>**Review Group:** Epilepsy	**Studies:** RCTs (including quasi-).<br><br>**Patients:** Children or adults with focal onset seizures (simple focal, complex focal, or secondarily generalised tonic-clonic	**Outcome type and measure:** time to event; hazard ratio.<br>**Number of trials:** not reported.<br>**Number of patients:** 10286 (for focal)<br>**Type of analysis:** Frequentist.

**Date:** 2022<br><br>**Sources of support:** National Institute for Health Research	seizures) or generalised onset tonic-clonic seizures (with or without other generalised seizure types).<br>Two networks produced (focal and generalised).<br><br>**Interventions analysed for primary outcome:** phenobarbitone, phenytoin, sodium valproate, lamotrigine, oxcarbazepine, topiramate, gabapentin, levetiracetam, zonisamide, carbamazepine and lacosamide (11).<br><br>**Outcome: t**ime to treatment failure (first primary).	**Heterogeneity assessed:** Yes (I square, chi-square).<br>**Heterogeneity found:** Yes.<br>**Consistency/transitivity assessed:** Yes (inconsistency model, node-splitting).<br>**Inconsistency/intransitivity found:** no.<br><br>**Grade classification**: high (for focal).<br><br>**ROB (SG/AC):** cannot be extracted
**Review ID:** CD010844.pub2[18]<br><br>**Review Group:** Airways<br><br>**Date:** 2014<br><br>**Sources of support:** National Institute for Health Research	**Studies:** RCTs<br><br>**Patients:** participants with a clinical diagnosis of COPD<br><br>**Interventions analysed for primary outcome:** placebo, LABA, LAMA, ICS, LABA/ICS (5).<br><br>**Outcome:** Quality of life (measured as change from baseline assessed with St George's Respiratory Questionnaire) (first primary).	**Outcome type and measure:** continuous; mean difference.<br>**Number of trials:** 25<br>**Number of patients:** 27024<br>**Type of analysis:** Bayesian.<br><br>**Heterogeneity assessed:** Yes (I square).<br>**Heterogeneity found:** No.<br>**Consistency/transitivity assessed:** Yes (inconsistency model, node-splitting).<br>**Inconsistency/intransitivity found:** Yes.<br><br>**Grade classification**: not assessed.<br><br>**ROB (SG/AC):** cannot be extracted for specific analysis but all trials are low or unclear.
**Review ID:** CD011867.pub2[19]<br><br>**Review Group:** Work<br><br>**Date:** 2017<br><br>**Sources of support:** Institute Gak, Netherlands.	**Studies:** RCTs (including cluster).<br><br>**Patients: a**dults aged between 18 and 70 years who had been diagnosed with severe mental illness.<br><br>**Interventions analysed for primary outcome:** augmented supported employment, supported employment, pre-vocational training, transitional employment, psychiatric care only (5).<br><br>**Outcome:** Number of participants who obtained competitive employment (follow up > 1 year (first primary).	**Outcome type and measure:** dichotomous; risk ratio.<br>**Number of trials:** 22<br>**Number of patients:**5233<br>**Type of analysis:** Frequentist.<br><br>**Heterogeneity assessed:** Yes (I square).<br>**Heterogeneity found:** yes.<br>**Consistency/transitivity assessed:** Yes (inconsistency model, loops).<br>**Inconsistency/intransitivity found:** yes.<br><br>**Grade classification**: very low/low/moderate.<br><br>**ROB (SG/AC):** cannot be extracted for specific analysis but all trials are low or unclear.
**Review ID:** CD012620.pub2[20]<br><br>**Review Group:** Airways	**Studies:** RCTs (at least 12 weeks duration).	**Outcome type and measure:** time to events; hazard ratio.<br>**Number of trials** 21<br>**Number of patients:** 25771

**Date:** 2018<br><br>**Sources of support:** National Institute for Health Research; Medical Research Council.	**Patients:** people aged 35 years or older with a diagnosis of COPD.<br>Results stratified by high and low risk; high risk reported first.<br><br>**Interventions analysed for primary outcome:** LABA; LAMA; LABA/ICS; LABA/LAMA (4).<br><br>**Outcome:** COPD exacerbations (moderate to severe and severe) (first primary).	**Type of analysis:** Frequentist.<br><br>**Heterogeneity assessed:** Yes (model comparison).<br>**Heterogeneity found:** No<br>**Consistency/transitivity assessed:** Yes (inconsistency model).<br>**Inconsistency/intransitivity found:** no<br><br>**Grade classification**: no reported.<br><br>**ROB (SG/AC):** cannot be extracted for specific analysis but all trials are low or unclear.
**Review ID:** CD011689.pub3[21]<br><br>**Review Group:** Pregnancy & Childbirth<br><br>**Date:** 2018<br><br>**Sources of support:** National Institute for Health Research; University of Birmingham, UK; World Health Organization, Switzerland; University of the Witwatersrand, South Africa; Birmingham Women's NHS Foundation Trust, UK; Ammalife, UK.	**Studies:** RCTs (including cluster).<br><br>**Patients:** women in the third stage of labour.<br><br>**Interventions analysed for primary outcome:** carbetocin, misoprostol, injectable prostaglandins, ergometrine, ergometrine plus oxytocin (Syntometrine ®), misoprostol plus oxytocin, oxytocin (7).<br><br>**Outcome:** Postpartum haemorrhage >= 500 mL (first primary).	**Outcome type and measure:** dichotomous; risk ratio.<br>**Number of trials:** 124.<br>**Number of patients:** not reported.<br>**Type of analysis:** Frequentist.<br><br>**Heterogeneity assessed:** Yes (I square).<br>**Heterogeneity found:** Not reported.<br>**Consistency/transitivity assessed:** Yes (inconsistency model).<br>**Inconsistency/intransitivity found:** not reported.<br><br>**Grade classification**: low/moderate.<br><br>**ROB (SG/AC):** some high risk trials.
**Review ID:** CD010813.pub2[22]<br><br>**Review Group:** Fertility Regulation<br><br>**Date:** 2014<br><br>**Sources of support:** CAPES-NUFFIC, Brazil.	**Studies:** RCTs and case-control, cohort, and nested case-control design<br><br>**Patients:** healthy women taking a combined oral contraceptive.<br><br>**Interventions analysed for primary outcome:** 1st, 2nd, 3rd generation, npn-use (4). Also compared individual drugs secondary.<br><br>**Outcome:** fatal or non-fatal first venous thrombosis event (first primary).	**Outcome type and measure:** dichotomous; risk ratio.<br>**Number of trials:** 23<br>**Number of patients:** not reported<br>**Type of analysis:** Frequentist.<br><br>**Heterogeneity assessed:** Yes (chi-square).<br>**Heterogeneity found:** Not reported.<br>**Consistency/transitivity assessed:** Yes (chi-square).<br>**Inconsistency/intransitivity found:** no.<br><br>**Grade classification**: not assessed.<br><br>**ROB (SG/AC):** cannot be extracted for specific analysis
**Review ID:** CD007868.pub3[23]<br><br>**Review Group:** Oral Health<br><br>**Date:** 2019	**Studies:** RCTs (including cluster).<br><br>**Patients: C**hildren and adolescents between 5 and 15 years of age at the start of the study	**Outcome type and measure: continuous**; SMD.<br>**Number of trials:** 81.<br>**Number of patients:** Not reported.<br>**Type of analysis:** Frequentist.

**Sources of support:** National Institute for Health Research; University of Manchester, UK; Academic Health Science Centre (MAHSC), UK.; Department of Health UK.	**Interventions analysed for primary outcome:** 0, 250, 440, 1000, 1450, 1700, 2400 flouride (7).<br><br>**Outcome: C**aries increment (surface index D3(M)FS) (first primary).	**Heterogeneity assessed:** Yes (I square, chi-square).<br>**Heterogeneity found:** Yes.<br>**Consistency/transitivity assessed:** Yes (inconsistency model, loops, node-splitting).<br>**Inconsistency/intransitivity found:** no.<br><br>**Grade classification**: low/moderate/high.<br><br>**ROB (SG/AC):** cannot be extracted for specific analysis but all trials are low or unclear.
**Review ID:** .CD013125.pub2[24]<br><br>**Review Group:** Hepato-Biliary<br><br>**Date:** 2020<br><br>**Sources of support:** National Institute for Health Research; University College London, UK.	**Studies:** RCTs (including cluster and cross-over).<br><br>**Patients: A**dults with liver cirrhosis, who were undergoing prophylactic treatment to prevent spontaneous bacterial peritonitis.<br><br>**Interventions analysed for primary outcome:** ciprofloxacin, neomycin, norfloxacin, norfloxacin plus neomycin, norfloxacin plus rifaximin, rifaximin, rufloxacin, sparfloxacin, sulfamethoxazole plus trimethoprim, and no active intervention (7).<br><br>**Outcome:** All-cause mortality at maximal follow-up (first primary).	**Outcome type and measure:** time to event; hazard ratio.<br>**Number of trials:** 17.<br>**Number of patients:** 2169.<br>**Type of analysis:** Bayesian.<br><br>**Heterogeneity assessed:** Yes (fixed effect and random effect model comparison).<br>**Heterogeneity found:** No.<br>**Consistency/transitivity assessed:** Yes (inconsistency models).<br>**Inconsistency/intransitivity found:** No.<br><br>**Grade classification:** Very low.<br><br>**ROB (SG/AC):** Low and unclear.
**Review ID:** CD013674.pub2**[25]**<br><br>**Review Group:** Common Mental Disorders<br><br>**Date:** 2021<br><br>**Sources of support:**<br>University of Auckland, New Zealand; Monash University, Australia; The University of Melbourne, Australia; Auckland Medical Research Foundation, New Zealand; A Better Start, National Science Challenge, Ministry of Business, Innovation & Employment, New Zealand; Cure Kids, New Zealand; National Health and Medical Research	**Studies:** RCTs (including cluster and cross-over).<br><br>**Patients**: children and/or adolescents with depression with acute phase treatment of clinically diagnosed major depressive disorder<br><br>**Interventions analysed for primary outcome:** placebo, fluoxetine, sertraline, citalopram, escitalopram, venlafaxine, mirtazapine, desvenlafaxine, duloxetine, vilazodone, vortioxetine, paroxetine (12)<br><br>**Outcome:** clinician-rated depression symptoms (secondary, no NMA or data for primary outcomes).	**Outcome type and measure:** continuous; mean difference.<br>**Number of trials:** 22<br>**Number of patients:** 5750<br>**Type of analysis:** frequentist.<br><br>**Heterogeneity assessed:** Yes (I square).<br>**Heterogeneity found:** Yes.<br>**Consistency/transitivity assessed:** Yes (inconsistency models).<br>**Inconsistency/intransitivity found:** No.<br><br>**Grade classification:** Very low/low/moderate/high<br><br>**ROB (SG/AC):** Not able to extract for specific analysis (all trials in review are unclear or low).

Council, Australia; National Institute for Health Research UK		
**Review ID:** CD013856.pub2[26]<br><br>**Review Group:** Movement Disorders<br><br>**Date:** 2023<br><br>**Sources of support:** German Federal Ministry of Education and Research; University Hospital of Cologne, Germany.	**Studies:** RCTs<br><br>**Patients:** adults with a confirmed diagnosis of idiopathic Parkinson's disease<br><br>**Interventions analysed for primary outcome:** dance, aqua-based training, gait/balance/functional training, multi-domain training, strength/resistance training, mind-body training, endurance training, flexibility training, gaming, LSVT BIG; passive control (11).<br><br>**Outcome:** Severity of motor signs (first primary).	**Outcome type and measure:** continuous; SMD.<br>**Number of trials:** 71<br>**Number of patients:** 3196.<br>**Type of analysis:** Frequentist.<br><br>**Heterogeneity assessed:** Yes (I square, chi-square).<br>**Heterogeneity found:** Yes<br>**Consistency/transitivity assessed:** Yes (Node-splitting).<br>**Inconsistency/intransitivity found:** Not reported.<br><br>**Grade classification:** Very low/low/high<br><br>**ROB (SG/AC):** Some high risk trials.

Abbreviations: AC: allocation concealment; RCT: randomised controlled trial; SG: sequence generation.

1. Wilson A, Hodgetts-Morton VA, Marson EJ, et al. Tocolytics for delaying preterm birth: a network meta-analysis (0924). *Cochrane Database Syst Rev* 2022(8) doi: 10.1002/14651858.CD014978.pub2
2. Ghosh J, Papadopoulou A, Devall AJ, et al. Methods for managing miscarriage: a network meta-analysis. *Cochrane Database Syst Rev* 2021(6) doi: 10.1002/14651858.CD012602.pub2
3. Janjua S, Mathioudakis AG, Fortescue R, et al. Prophylactic antibiotics for adults with chronic obstructive pulmonary disease: a network meta-analysis. *Cochrane Database Syst Rev* 2021(1) doi: 10.1002/14651858.CD013198.pub2
4. Rodríguez-Perálvarez M, Guerrero-Misas M, Thorburn D, et al. Maintenance immunosuppression for adults undergoing liver transplantation: a network meta-analysis. *Cochrane Database Syst Rev* 2017(3) doi: 10.1002/14651858.CD011639.pub2
5. Plaz Torres M, Best LMJ, Freeman SC, et al. Secondary prevention of variceal bleeding in adults with previous oesophageal variceal bleeding due to decompensated liver cirrhosis: a network meta-analysis. *Cochrane Database Syst Rev* 2021(3) doi: 10.1002/14651858.CD013122.pub2
6. Roccarina D, Best LMJ, Freeman SC, et al. Primary prevention of variceal bleeding in people with oesophageal varices due to liver cirrhosis: a network meta-analysis. *Cochrane Database Syst Rev* 2021(4) doi: 10.1002/14651858.CD013121.pub2
7. Piechotta V, Jakob T, Langer P, et al. Multiple drug combinations of bortezomib, lenalidomide, and thalidomide for first-line treatment in adults with transplant-ineligible multiple myeloma: a network meta-analysis. *Cochrane Database Syst Rev* 2019(11) doi: 10.1002/14651858.CD013487
8. Best LMJ, Leung J, Freeman SC, et al. Induction immunosuppression in adults undergoing liver transplantation: a network meta-analysis. *Cochrane Database Syst Rev* 2020(1) doi: 10.1002/14651858.CD013203.pub2
9. Scheiman M, Kulp MT, Cotter SA, et al. Interventions for convergence insufficiency: a network meta-analysis. *Cochrane Database Syst Rev* 2020(12) doi: 10.1002/14651858.CD006768.pub3
10. Weibel S, Rücker G, Eberhart LHJ, et al. Drugs for preventing postoperative nausea and vomiting in adults after general anaesthesia: a network meta-analysis. *Cochrane Database Syst Rev* 2020(10) doi: 10.1002/14651858.CD012859.pub2
11. Best LMJ, Freeman SC, Sutton AJ, et al. Treatment for hepatorenal syndrome in people with decompensated liver cirrhosis: a network meta-analysis. *Cochrane Database Syst Rev* 2019(9) doi: 10.1002/14651858.CD013103.pub2

12. Norman G, Westby MJ, Rithalia AD, et al. Dressings and topical agents for treating venous leg ulcers. *Cochrane Database Syst Rev* 2018(6) doi: 10.1002/14651858.CD012583.pub2
13. Burry L, Hutton B, Williamson DR, et al. Pharmacological interventions for the treatment of delirium in critically ill adults. *Cochrane Database Syst Rev* 2019(9) doi: 10.1002/14651858.CD011749.pub2
14. Sawangjit R, Dilokthornsakul P, Lloyd-Lavery A, et al. Systemic treatments for eczema: a network meta-analysis. *Cochrane Database Syst Rev* 2020(9) doi: 10.1002/14651858.CD013206.pub2
15. Devall AJ, Papadopoulou A, Podesek M, et al. Progestogens for preventing miscarriage: a network meta-analysis. *Cochrane Database Syst Rev* 2021(4) doi: 10.1002/14651858.CD013792.pub2
16. Pompoli A, Furukawa TA, Imai H, et al. Psychological therapies for panic disorder with or without agoraphobia in adults: a network meta-analysis. *Cochrane Database Syst Rev* 2016(4) doi: 10.1002/14651858.CD011004.pub2
17. Nevitt SJ, Sudell M, Cividini S, Marson AG, Tudur Smith C. Antiepileptic drug monotherapy for epilepsy: a network meta-analysis of individual participant data. *Cochrane Database Syst Rev* 2022(4) doi: 10.1002/14651858.CD011412.pub4
18. Kew KM, Dias S, Cates CJ. Long-acting inhaled therapy (beta-agonists, anticholinergics and steroids) for COPD: a network meta-analysis. *Cochrane Database Syst Rev* 2014(3) doi: 10.1002/14651858.CD010844.pub2
19. Suijkerbuijk YB, Schaafsma FG, van Mechelen JC, et al. Interventions for obtaining and maintaining employment in adults with severe mental illness, a network meta-analysis. *Cochrane Database Syst Rev* 2017(9) doi: 10.1002/14651858.CD011867.pub2
20. Oba Y, Keeney E, Ghatehorde N, Dias S. Dual combination therapy versus long-acting bronchodilators alone for chronic obstructive pulmonary disease (COPD): a systematic review and network meta-analysis. *Cochrane Database Syst Rev* 2018(12) doi: 10.1002/14651858.CD012620.pub2
21. Gallos ID, Papadopoulou A, Man R, et al. Uterotonic agents for preventing postpartum haemorrhage: a network meta-analysis. *Cochrane Database Syst Rev* 2018(12) doi: 10.1002/14651858.CD011689.pub3
22. de Bastos M, Stegeman BH, Rosendaal FR, et al. Combined oral contraceptives: venous thrombosis. *Cochrane Database Syst Rev* 2014(3) doi: 10.1002/14651858.CD010813.pub2
23. Walsh T, Worthington HV, Glenny AM, Marinho VCC, Jeroncic A. Fluoride toothpastes of different concentrations for preventing dental caries. *Cochrane Database Syst Rev* 2019(3) doi: 10.1002/14651858.CD007868.pub3
24. Komolafe O, Roberts D, Freeman SC, et al. Antibiotic prophylaxis to prevent spontaneous bacterial peritonitis in people with liver cirrhosis: a network meta-analysis. *Cochrane Database Syst Rev* 2020(1) doi: 10.1002/14651858.CD013125.pub2
25. Hetrick SE, McKenzie JE, Bailey AP, et al. New generation antidepressants for depression in children and adolescents: a network meta-analysis. *Cochrane Database Syst Rev* 2021(5) doi: 10.1002/14651858.CD013674.pub2
26. Ernst M, Folkerts AK, Gollan R, et al. Physical exercise for people with Parkinson's disease: a systematic review and network meta-analysis. *Cochrane Database Syst Rev* 2023(1) doi: 10.1002/14651858.CD013856.pub2
