# Supplementary material for: The impact of Cochrane Reviews that apply network meta-analysis in clinical guidelines: A systematic review
Source: PLoS One. 2024 Dec 26;19(12):e0315563. doi: 10.1371/journal.pone.0315563 (PMC11671017; doi:10.1371/journal.pone.0315563)
Supplement: S3 Table — (PDF) [file pone.0315563.s009.pdf]

Table S3: Data extracted for guidelines that cited network meta-analysis reviews

Review	Review year	Number of citations	Guideline location	Guideline year	Different version of the same guideline cited for a review	Guideline titles	Guideline author	Use of review in guideline	Guideline citation
CD006768.pub3[1]	2020	1	USA	2022	No	Esotropia and Exotropia (AAO Preferred Practice Pattern Guidelines)	American Academy of Ophthalmology	Referenced alongside a recommendation.	American Academy of Ophthalmology. Esotropia and Exotropia (AAO Preferred Practice Pattern Guidelines). San Francisco (CA): American Academy of Ophthalmology; December 2022. Available from: https://www.aao.org/preferred-practice-pattern/esotropia-exotropia-ppp
CD007868.pub3[2]	2019	5	Canada	2020	No	Oral Health: Supporting Adults Who Require Assistance (Second Edition)	Registered Nurses' Association of Ontario	Giving information	Registered Nurses' Association of Ontario. Oral Health: Supporting Adults Who Require Assistance (Second Edition). Toronto (ON): Registered Nurses' Association of Ontario; 2020. Available from: https://rnao.ca/sites/rnao-ca/files/bpg/RNAO_Oral_Health_Supporting_Adults_Who_Require_Assistance_Second_Edition_final.pdf
CD007868.pub3[2]			Iran	2023	No	Clinical practice guideline adaptation for risk-based caries management in 18-55 year-old Iranian adults	Pakdaman, Gholizadeh, Kharazifard, Eshrati	Referenced alongside a recommendation.	Pakdaman A, Gholizadeh N, Kharazifard MJ, Eshrati M. Clinical practice guideline adaptation for risk-based caries management in 18-55 year-old Iranian adults. BMC Oral Health. 2023 Jan 6;23(1):7. doi: 10.1186/s12903-022-02699-w. Available from: https://www.ncbi.nlm.nih.gov/pmc/articles/PMC9824988/pdf/12903_2022_Article_2699.pdf
CD007868.pub3[2]			Australia	2020	No	Guidelines for the use of fluorides in	Australian Research Centre	Giving information	Do LG; Australian Research Centre for Population Oral Health.

Review	Review year	Number of citations	Guideline location	Guideline year	Different version of the same guideline cited for a review	Guideline titles	Guideline author	Use of review in guideline	Guideline citation
						Australia: update 2019	for Population Oral Health	leading to recommendations.	Guidelines for the use of fluorides in Australia: update 2019. Australian Dental Journal. 2020 Mar;65(1):30-38. doi: 10.1111/adj.12742. Available from: https://www.adelaide.edu.au/arcpoh/dperu/fluoride/Guidelines_for_use_of_fluorides_in_Australia__update_2019.pdf
CD007868.pub3[2]			Europe	2019	No	Guidelines on the use of fluoride for caries prevention in children: an updated EAPD policy document	European Academy of Paediatric Dentistry	Giving information leading to recommendations.	Toumba KJ, Twetman S, Splieth C, Parnell C, van Loveren C, Lygidakis NA. Guidelines on the use of fluoride for caries prevention in children: an updated EAPD policy document. Eur Arch Paediatr Dent. 2019 Dec;20(6):507-516. doi: 10.1007/s40368-019-00464-2. Available from: https://link.springer.com/content/pdf/10.1007/s40368-019-00464-2.pdf
CD007868.pub3[2]			Europe	2020	No	How to Intervene in the Caries Process in Children: A Joint ORCA and EFCD Expert Delphi Consensus Statement	European Organization for Caries Research, European Federation of Conservative Dentistry, German Association of Conservative Dentistry	Giving information leading to recommendations.	Splieth CH, Banerjee A, Bottenberg P, Breschi L, Campus G, Ekstrand KR, Giacaman RA, Haak R, Hannig M, Hickel R, Juric H, Lussi A, Machiulskiene V, Manton DJ, Jablonski-Momeni A, Opdam NJM, Paris S, Santamaría RM, Schwendicke F, Tassery H, Ferreira Zandona A, Zero DT, Zimmer S, Doméjean S. How to Intervene in the Caries Process in Children: A Joint ORCA and EFCD Expert Delphi Consensus Statement. Caries Res. 2020;54(4):297-305. doi:

Review	Review year	Number of citations	Guideline location	Guideline year	Different version of the same guideline cited for a review	Guideline titles	Guideline author	Use of review in guideline	Guideline citation
									10.1159/000507692. Available from: https://www.karger.com/Article/PDF/507692
CD010813.pub2[3]	2014	18	Spain	2014	No	[Preventive activities in women]	PAPPS Women's Group	Giving information leading to recommendations.	López García-Franco A, Arribas Mir L, del Cura González I, Bailón Muñoz E, Iglesias Piñeiro MJ, Gutiérrez Teira B, Landa Goñi J, Ojuel Solsona J, Fuentes Pujol M, Alonso Coello P; Grupo de la Mujer del PAPPS. [Preventive activities in women]. Aten Primaria. 2014 Jun;46(Suppl 4):82-98. Available from: http://www.sciencedirect.com/science/article/pii/S0212656714700538
CD010813.pub2[3]			Spain	2019	No	[Clinical Practice Guidelines on Hormone and Intrauterine Contraception]	Clinical Practice Guideline for Hormonal and Intrauterine Contraception Working Group	Giving information leading to recommendations.	Working Group of the Clinical Practice Guidelines for Hormonal and Intrauterine Contraception. Clinical Practice Guidelines for Hormonal and Intrauterine Contraception. Ministry of Health, Consumer Affairs and Social Welfare. Aragonese Institute of Sciences of the Bless you; 2019. Clinical Practice Guidelines in the NHS
CD010813.pub2[3]			Finland	2023	No	[Duodecim Current Care Guidelines: Deep vein thrombosis and pulmonary embolism]	Finnish Medical Society Duodecim, Finnish Angiology Association, Finnish Cardiological Society	Giving information.	Deep vein thrombosis and pulmonary embolism. Current Care Recommendation. Working group appointed by the Finnish Medical Society Duodecim and the Finnish Society of Cardiology. Helsinki: Finnish Medical Society Duodecim, 2023. Available Internet: www.kaypahoito.fi

Review	Revie w year	Number of citations	Guideline location	Guideline year	Different version of the same guideline cited for a review	Guideline titles	Guideline author	Use of review in guideline	Guideline citation
CD010813.pub 2[3]			Netherland s	2020	Yes	[Dutch College of General Practitioners – Guideline on contraception (version 2.0 – Guideline M02)]	Dutch College of General Practitioners working group.	Cannot access.	Barnhoorn PC, Bruinsma ACA, Bouma M, Damen Z, De Swart SM, Koetsier MJE, Kurver MJ, Van der Sande R, Van der Wijden CL, Van Groeningen COM, NHG Werkgroep. NHG-Standaard Anticonceptie (versie 2.0 – NHG Standaard M02). [Dutch College of General Practitioners – Guideline on contraception (version 2.0 – Guideline M02)]. Utrecht: Nederlands Huisartsen Genootschap; May 2020. Available from: https://richtlijnen.nhg.org/standaarden/anticonceptie#volledige-tekst
CD010813.pub 2[3]			Netherland s	2021	Yes	[Dutch College of General Practitioners – Guideline on contraception (version 2.2 – Guideline M02)]	Dutch College of General Practitioners working group.	Cannot access.	Barnhoorn PC, Bruinsma ACA, Bouma M, Damen Z, De Swart SM, Koetsier MJE, Kurver MJ, Van der Sande R, Van der Wijden CL, Van Groeningen COM, NHG Werkgroep. NHG-Standaard Anticonceptie (versie 2.2 – NHG Standaard M02). [Dutch College of General Practitioners – Guideline on contraception (version 2.2 – Guideline M02)]. Utrecht: Nederlands Huisartsen Genootschap; February 2021. Available from: https://richtlijnen.nhg.org/files/pdf/100_Anticonceptie_februari-2021.pdf

Review	Review year	Number of citations	Guideline location	Guideline year	Different version of the same guideline cited for a review	Guideline titles	Guideline author	Use of review in guideline	Guideline citation
CD010813.pub2[3]			Netherlands	2023	Yes	[Dutch College of General Practitioners – Guideline on contraception (version 2.3 - Guideline M02]	Dutch College of General Practitioners working group.	Cannot access.	Barnhoorn PC, Bruinsma ACA, Bouma M, Damen Z, De Swart SM, Koetsier MJE, Kurver MJ, Van der Sande R, Van der Wijden CL, Van Groeningen COM, NHG Werkgroep. NHG-Standaard Anticonceptie (versie 3.1 - NHG Standaard M02). [Dutch College of General Practitioners – Guideline on contraception (version 2.3 - Guideline M02]. Utrecht: Nederlands Huisartsen Genootschap; February 2023. Available from: https://richtlijnen.nhg.org/files/pdf/100_Anticonceptie_februari-2023.pdf
CD010813.pub2[3]			Netherlands	2023	Yes	[Dutch College of General Practitioners – Guideline on contraception (version 2.4 - Guideline M02)]	Dutch College of General Practitioners working group.	Giving information.	Barnhoorn PC, Bruinsma ACA, Bouma M, Damen Z, De Swart SM, Koetsier MJE, Kurver MJ, Van der Sande R, Van der Wijden CL, Van Groeningen COM, NHG Werkgroep. NHG-Standaard Anticonceptie (versie 2.4 - NHG Standaard M02). [Dutch College of General Practitioners – Guideline on contraception (version 2.4 - Guideline M02)]. Utrecht: Nederlands Huisartsen Genootschap; April 2023. Available from: https://richtlijnen.nhg.org/files/pdf/100_Anticonceptie_april-2023.pdf
CD010813.pub2[3]			Germany	2022	No	[S2k-Guideline: Secondary	German Society of Neurology,	Giving information.	Olma M. C., Röther J., Grau A., Kurth T. et al., Secondary prophylaxis of

Review	Review year	Number of citations	Guideline location	Guideline year	Different version of the same guideline cited for a review	Guideline titles	Guideline author	Use of review in guideline	Guideline citation
						prevention of ischemic stroke and transient ischemic attack - part 2: lifestyle, arterial stenosis, other antithrombotic indications, hormones, diabetes mellitus, sleep apnoea]	German Stroke Society		ischemic stroke and transient ischemic attack – Part 2, S2k guideline, 2022, German Society of Neurology (DGN) and German Stroke Society (DSG), Online: www.dgn.org/leitlinien.
CD010813.pub2[3]			Germany	2019	No	[S3 Guideline: Hormonal contraception]	German Society of Gynecology and Obstetrics, Austrian Society of Gynecology and Obstetrics, Swiss Society of Gynecology and Obstetrics	Referenced alongside a recommendation.	German Society of Gynaecology and Obstetrics (DGGG); Austrian Society of Gynaecology and Obstetrics (OEGGG); Swiss Society of Gynaecology and Obstetrics (SGGG). S3 Guideline: Hormonal contraception. Berlin: German Society of Gynaecology and Obstetrics (DGGG); 2019. Available from: https://www.awmf.org/leitlinien/detail/ll/015-015.html
CD010813.pub2[3]			Europe	2020	No	2019 ESC Guidelines for the diagnosis and management of acute pulmonary embolism developed in collaboration with the European Respiratory Society (ERS)	European Society of Cardiology and European Respiratory Society.	Giving information leading to recommendations	Konstantinides SV, Meyer G, Becattini C, Bueno H, Geersing GJ, Harjola VP, Huisman MV, Humbert M, Jennings CS, Jiménez D, Kucher N, Lang IM, Lankeit M, Lorusso R, Mazzolai L, Meneveau N, Ní Áinle F, Prandoni P, Pruszczyk P, Righini M, Torbicki A, Van Belle E, Zamorano JL; ESC Scientific Document Group. 2019 ESC Guidelines for the diagnosis and management of acute

Review	Review year	Number of citations	Guideline location	Guideline year	Different version of the same guideline cited for a review	Guideline titles	Guideline author	Use of review in guideline	Guideline citation
									pulmonary embolism developed in collaboration with the European Respiratory Society (ERS). European Heart Journal. 2020 Jan;41(4):543-603. doi: 10.1093/eurheartj/ehz405. Available from: https://www.ncbi.nlm.nih.gov/pubmed/31504429
CD010813.pub2[3]			UK	2020	Yes	Combined Hormonal Contraception (Faculty of Sexual & Reproductive Healthcare Clinical Guidance)	Clinical Effectiveness Unit, Faculty of Sexual & Reproductive Healthcare, Royal College of Obstetricians & Gynaecologists	Cannot access.	Clinical Effectiveness Unit, Faculty of Sexual & Reproductive Healthcare, Royal College of Obstetricians & Gynaecologists. Combined Hormonal Contraception (Faculty of Sexual & Reproductive Healthcare Clinical Guidance). London: Royal College of Obstetricians & Gynaecologists; 2020. [Issued January 2011; amended November 2020]. Available from: https://www.fsrh.org/standards-and-guidance/documents/combined-hormonal-contraception/
CD010813.pub2[3]			UK	2023	Yes	Combined Hormonal Contraception (Faculty of Sexual & Reproductive Healthcare Clinical Guidance)	Clinical Effectiveness Unit, Faculty of Sexual & Reproductive Healthcare, Royal College of Obstetricians & Gynaecologists	Giving information leading to recommendations.	Clinical Effectiveness Unit, Faculty of Sexual & Reproductive Healthcare, Royal College of Obstetricians & Gynaecologists. Combined Hormonal Contraception (Faculty of Sexual & Reproductive Healthcare Clinical Guidance). London: Royal College of Obstetricians & Gynaecologists; 2023. [Issued January 2011; amended October 2023]. Available from:

Review	Review year	Number of citations	Guideline location	Guideline year	Different version of the same guideline cited for a review	Guideline titles	Guideline author	Use of review in guideline	Guideline citation
									https://www.fsrh.org/standards-and-guidance/documents/combined-hormonal-contraception/
CD010813.pub2[3]			UK	2019	Yes	Combined Hormonal Contraception. (Faculty of Sexual & Reproductive Healthcare Clinical Guidance)	Clinical Effectiveness Unit, Faculty of Sexual & Reproductive Healthcare, Royal College of Obstetricians & Gynaecologists	Cannot access.	Clinical Effectiveness Unit; Faculty of Sexual & Reproductive Healthcare; Royal College of Obstetricians & Gynaecologist. Combined Hormonal Contraception. (Faculty of Sexual & Reproductive Healthcare Clinical Guidance). London: Royal College of Obstetricians & Gynaecologists; 2019. [Issued October 2011; updated July 2019. Available from: https://www.fsrh.org/standards-and-guidance/documents/combined-hormonal-contraception/
CD010813.pub2[3]			USA	2017	No	Combined hormonal contraception and the risk of venous thromboembolism: a guideline	Practice Committee of the American Society for Reproductive Medicine	Cannot access	Practice Committee of the American Society for Reproductive Medicine. Combined hormonal contraception and the risk of venous thromboembolism: a guideline. Fertil Steril. 2017 Jan;107(1):43-51. doi: 10.1016/j.fertnstert.2016.09.027. Available from: http://www.asrm.org/globalassets/asrm/asrm-content/news-and-publications/practice-guidelines/for-non-members/combined_hormonal_contraception_vte-pdfnoprint.pdf

Review	Review year	Number of citations	Guideline location	Guideline year	Different version of the same guideline cited for a review	Guideline titles	Guideline author	Use of review in guideline	Guideline citation
CD010813.pub2[3]			Canada	2018	No	Contraceptive care for Canadian youth (position statement)	Canadian Paediatric Society Adolescent Health Committee	Giving information leading to recommendations.	Di Meglio G; Crowther C; Simms J; Canadian Paediatric Society Adolescent Health Committee. Contraceptive care for Canadian youth (position statement). Paediatr Child Health. 2018 Jun; 23(4):271-277. Available from: https://www.cps.ca/en/documents/position/contraceptive-care
CD010813.pub2[3]			Global	2023	No	International Evidence-based Guideline for the assessment and management of polycystic ovary syndrome 2023	Centre for Research Excellence in Women's Health in Reproductive Life, American Society of Reproductive Medicine, Endocrine Society, European Society of Endocrinology, European Society of Human Reproduction and Embryology	Giving information	Teede H, Tay CT, Laven J, Dokras A, Moran L, Piltonen T, Costello M, Boivin J, Redman L, Boyle J, Norman R, Mousa A, Joham A; Centre for Research Excellence in Women's Health in Reproductive Life (CRE WHiRL); American Society of Reproductive Medicine (ASRM); Endocrine Society; European Society of Endocrinology; European Society of Human Reproduction and Embryology (ESHRE). International Evidence-based Guideline for the assessment and management of polycystic ovary syndrome 2023. Melbourne: Monash University; 2023. [Issued 2011; last updated February 2023]. Available from: https://www.monash.edu/__data/assets/pdf_file/0003/3379521/Evidence-Based-Guidelines-2023.pdf
CD010813.pub2[3]			UK	2019	No	Overweight, Obesity & Contraception	Clinical Effectiveness Unit, Faculty of Sexual & Reproductive	Giving information leading to	Clinical Effectiveness Unit, Faculty of Sexual and Reproductive Healthcare (FSRH); Royal College of Obstetricians & Gynaecologists.

Review	Review year	Number of citations	Guideline location	Guideline year	Different version of the same guideline cited for a review	Guideline titles	Guideline author	Use of review in guideline	Guideline citation
							Healthcare, Royal College of Obstetricians & Gynaecologists	recommendations.	FSRH Guideline: Overweight, Obesity & Contraception (Faculty of Sexual & Reproductive Healthcare Clinical Guideline). London: Royal College of Obstetricians & Gynaecologists; 2019. [Issued April 2019]. Available from: https://www.fsrh.org/standards-and-guidance/documents/fsrh-clinical-guideline-overweight-obesity-and-contraception/
CD010813.pub2[3]			UK	2014	No	Venous Thromboembolism (VTE) and Hormonal Contraception. (Faculty of Sexual & Reproductive Healthcare Statement)	Clinical Effectiveness Unit, Faculty of Sexual & Reproductive Healthcare, Royal College of Obstetricians & Gynaecologists	Cannot access.	Clinical Effectiveness Unit, Faculty of Sexual & Reproductive Healthcare, Royal College of Obstetricians & Gynaecologists. Venous Thromboembolism (VTE) and Hormonal Contraception. (Faculty of Sexual & Reproductive Healthcare Statement). London: Royal College of Obstetricians & Gynaecologists; 2014 November. Available from: http://www.fsrh.org/pdfs/FSRHStatementVTEandHormonalContraception.pdf
CD010844.pub2[4]	2014	16	Australia and New Zealand	2018	Yes	The COPD-X Plan: Australian and New Zealand Guidelines for the management of Chronic Obstructive Pulmonary Disease 2018	Lung Foundation Australia; Thoracic Society of Australia and New Zealand	Cannot access	Yesang IA, Dabscheck E, George J, Jenkins S, McDonald CF, McDonald V, Smith B, Zwar N; on behalf of the Lung Foundation Australia and the Thoracic Society of Australia and New Zealand. The COPD-X Plan: Australian and New Zealand Guidelines for the management of Chronic Obstructive Pulmonary Disease 2018. Version 2.56,

Review	Review year	Number of citations	Guideline location	Guideline year	Different version of the same guideline cited for a review	Guideline titles	Guideline author	Use of review in guideline	Guideline citation
									December 2018. Sydney, NSW, Australia: Lung Foundation Australia and Thoracic Society of Australia and New Zealand; 2018. Available from: http://copdx.org.au/
CD010844.pub2[4]			UK	2016	No	Chronic obstructive pulmonary disease in over 16s: diagnosis and management: surveillance report for Guidance Executive relevant to NICE clinical guideline NG115 'Chronic obstructive pulmonary disease in over 16s: diagnosis and management' (2010)	Centre for Clinical Practice Surveillance Programme, National Institute for Health and Care Excellence (commissioner)	Not cited in the NICE guideline (but may have been referenced in supporting evidence).	National Clinical Guideline Centre; National Institute for Health and Care Excellence (commissioner). Surveillance report 2016 – Chronic obstructive pulmonary disease in over 16s: diagnosis and management (2010) NICE guideline CG101. London: National Clinical Guideline Centre, Royal College of Physicians; April 2016. Available from: https://www.nice.org.uk/guidance/ng115/resources/surveillance-report-2016-chronic-obstructive-pulmonary-disease-in-over-16s-diagnosis-and-management-2010-nice-guideline-cg101-pdf-3143275330021
CD010844.pub2[4]			South Africa	2019	No	Management of chronic obstructive pulmonary disease - A position statement of the South African Thoracic Society: 2019 update	South African Thoracic Society	Referenced alongside a recommendation.	Abdool-Gaffar MS, Calligaro G, Wong ML, Smith C, Lalloo UG, Koegelenberg CFN, Dheda K, Allwood BW, Goolam-Mahomed A, van Zyl-Smit RN. Management of chronic obstructive pulmonary disease - A position statement of the South African Thoracic Society: 2019 update. J Thorac Dis. 2019 Nov;11(11):4408-4427. doi: 10.21037/jtd.2019.10.65. Available

Review	Revie w year	Number of citations	Guideline location	Guideline year	Different version of the same guideline cited for a review	Guideline titles	Guideline author	Use of review in guideline	Guideline citation
									from: https://www.ncbi.nlm.nih.gov/pmc/articles/PMC6940223/pdf/jtd-11-11-4408.pdf
CD010844.pub2[4]			UK	2015	Yes	Preterm labour and birth	National Collaborating Centre for Women's and Children's Health, National Institute for Health and Care Excellence (commissioner)	Not cited in the NICE guideline (but may have been referenced in supporting evidence).	National Collaborating Centre for Women's and Children's Health, National Institute for Health and Care Excellence (commissioner). Preterm labour and birth. London: National Institute for Health and Care Excellence; 2015. (NICE NG25). [Issued November 2015]. Available from: https://www.nice.org.uk/guidance/ng25
CD010844.pub2[4]			UK	2019	Yes	Preterm labour and birth	National Collaborating Centre for Women's and Children's Health, National Institute for Health and Care Excellence (commissioner)	Not cited in the NICE guideline (but may have been referenced in supporting evidence).	National Collaborating Centre for Women's and Children's Health, National Institute for Health and Care Excellence (commissioner). Preterm labour and birth. London: National Institute for Health and Care Excellence; 2019. (NICE NG25). [Issued November 2015, updated August 2019]. Available from: https://www.nice.org.uk/guidance/ng25
CD010844.pub2[4]			UK	2022	Yes	Preterm labour and birth	National Collaborating Centre for Women's and Children's Health, National Institute for Health and	Not cited in the NICE guideline (but may have been referenced in supporting evidence).	National Collaborating Centre for Women's and Children's Health; National Institute for Health and Care Excellence (commissioner). Preterm labour and birth. London: National Institute for Health and Care Excellence; 2022. (NICE NG25). [Issued November 2015; last

Review	Review year	Number of citations	Guideline location	Guideline year	Different version of the same guideline cited for a review	Guideline titles	Guideline author	Use of review in guideline	Guideline citation
							Care Excellence (commissioner)		updated June 2022]. Available from: https://www.nice.org.uk/guidance/ng25
CD010844.pub2[4]			Australia and New Zealand	2014	Yes	The COPD-X Plan: Australian and New Zealand Guidelines for the management of Chronic Obstructive Pulmonary Disease 2014	Lung Foundation Australia and the Thoracic Society of Australia and New Zealand	Cannot access.	Abramson M; Brown J; Crockett AJ; Dabscheck E; Frith PA; George J; Glasgow N; Jenkins S; McDonald CF; McDonald V; McKenzie DK; Wood-Baker R; Yesang I; Zwar N; on behalf of the Lung Foundation Australia and the Thoracic Society of Australia and New Zealand. The COPD-X Plan: Australian and New Zealand Guidelines for the management of Chronic Obstructive Pulmonary Disease 2014. Version 2.39, October 2014. Sydney, NSW, Australia: Lung Foundation Australia and Thoracic Society of Australia and New Zealand; 2014. Available from: http://copdx.org.au/
CD010844.pub2[4]			Australia and New Zealand	2015	Yes	The COPD-X Plan: Australian and New Zealand Guidelines for the management of Chronic Obstructive Pulmonary Disease 2015	Lung Foundation Australia and the Thoracic Society of Australia and New Zealand	Cannot access	Yesang IA; Dabscheck E; George J; Jenkins S; McDonald CF; McDonald V; Smith B; Zwar N; on behalf of the Lung Foundation Australia and the Thoracic Society of Australia and New Zealand. The COPD-X Plan: Australian and New Zealand Guidelines for the management of Chronic Obstructive Pulmonary Disease 2015. Version 2.44, December 2015. Sydney, NSW, Australia: Lung Foundation Australia and Thoracic Society of Australia and

Review	Revie w year	Number of citations	Guideline location	Guideline year	Different version of the same guideline cited for a review	Guideline titles	Guideline author	Use of review in guideline	Guideline citation
									New Zealand; 2015. Available from: http://copdx.org.au/
CD010844.pub 2[4]			Australia and New Zealand	2016	Yes	The COPD-X Plan: Australian and New Zealand Guidelines for the management of Chronic Obstructive Pulmonary Disease 2016	Lung Foundation Australia and the Thoracic Society of Australia and New Zealand	Cannot access	Yesang IA, Dabscheck E, George J, Jenkins S, McDonald CF, McDonald V, Smith B, Zwar N.; on behalf of the Lung Foundation Australia and the Thoracic Society of Australia and New Zealand. The COPD-X Plan: Australian and New Zealand Guidelines for the management of Chronic Obstructive Pulmonary Disease 2016. Version 2.48, December 2016. Sydney, NSW, Australia: Lung Foundation Australia and Thoracic Society of Australia and New Zealand; 2016. Available from: http://copdx.org.au/
CD010844.pub 2[4]			Australia and New Zealand	2017	Yes	The COPD-X Plan: Australian and New Zealand Guidelines for the management of Chronic Obstructive Pulmonary Disease 2017	Lung Foundation Australia and the Thoracic Society of Australia and New Zealand	Cannot access	Yesang IA, Dabscheck E, George J, Jenkins S, McDonald CF, McDonald V, Smith B, Zwar N; on behalf of the Lung Foundation Australia and the Thoracic Society of Australia and New Zealand. The COPD-X Plan: Australian and New Zealand Guidelines for the management of Chronic Obstructive Pulmonary Disease 2017. Version 2.49, March 2017. Sydney, NSW, Australia: Lung Foundation Australia and Thoracic Society of Australia and New Zealand; 2017. Available from: http://copdx.org.au/

Review	Review year	Number of citations	Guideline location	Guideline year	Different version of the same guideline cited for a review	Guideline titles	Guideline author	Use of review in guideline	Guideline citation
CD010844.pub2[4]			Australia and New Zealand	2019	Yes	The COPD-X Plan: Australian and New Zealand Guidelines for the management of Chronic Obstructive Pulmonary Disease 2019	Lung Foundation Australia, Thoracic Society of Australia and New Zealand	Cannot access	The COPD-X Plan: Australian and New Zealand Guidelines for the management of Chronic Obstructive Pulmonary Disease 2019. Version 2.59, August 2019. Sydney, NSW, Australia: Lung Foundation Australia and Thoracic Society of Australia and New Zealand; 2019. Available from: https://copdx.org.au/wp-content/uploads/2019/11/COPDX-V2-59-Aug-2019-FINAL2.pdf
CD010844.pub2[4]			Australia and New Zealand	2020	Yes	The COPD-X Plan: Australian and New Zealand Guidelines for the management of Chronic Obstructive Pulmonary Disease 2020	Lung Foundation Australia, Thoracic Society of Australia and New Zealand	Giving information	Yesang IA, Dabscheck E, George J, Jenkins S, McDonald CF, McDonald V, Smith B, Zwar N; on behalf of the Lung Foundation Australia and the Thoracic Society of Australia and New Zealand. The COPD-X Plan: Australian and New Zealand Guidelines for the management of Chronic Obstructive Pulmonary Disease 2020. Version 2.62, October 2020. Sydney, NSW, Australia: Lung Foundation Australia and Thoracic Society of Australia and New Zealand; 2020. Available from: https://copdx.org.au/wp-content/uploads/2021/02/COPDX-V2.62-June_Oct-2020-PUBLISHED.pdf
CD010844.pub2[4]			Australia and New Zealand	2021	Yes	The COPD-X Plan: Australian and New Zealand Guidelines for the management of Chronic	Lung Foundation Australia, Thoracic Society of Australia and New Zealand	Giving information.	Yang IA, Dabscheck E, George J, Jenkins S, McDonald CF, McDonald V, Smith B, Zwar N; on behalf of the Lung Foundation Australia and the Thoracic Society of Australia and

Review	Review year	Number of citations	Guideline location	Guideline year	Different version of the same guideline cited for a review	Guideline titles	Guideline author	Use of review in guideline	Guideline citation
						Obstructive Pulmonary Disease 2021			New Zealand. The COPD-X Plan: Australian and New Zealand Guidelines for the management of Chronic Obstructive Pulmonary Disease 2021. Version 2.63, February 2021. Sydney, NSW, Australia: Lung Foundation Australia and Thoracic Society of Australia and New Zealand; 2021. Available from: https://copdx.org.au/wp-content/uploads/2021/04/COPDX-V2-63-Feb-2021_FINAL-PUBLISHED.pdf
CD010844.pub2[4]			Australia and New Zealand	2022	Yes	The COPD-X Plan: Australian and New Zealand Guidelines for the management of Chronic Obstructive Pulmonary Disease 2022	Lung Foundation Australia, Thoracic Society of Australia and New Zealand	Giving information.	Yang IA, Dabscheck E, George J, Jenkins S, McDonald CF, McDonald V, Smith B, Zwar N; on behalf of the Lung Foundation Australia and the Thoracic Society of Australia and New Zealand. The COPD-X Plan: Australian and New Zealand Guidelines for the management of Chronic Obstructive Pulmonary Disease 2022. Version 2.66, April 2022. Sydney, NSW, Australia: Lung Foundation Australia and Thoracic Society of Australia and New Zealand; 2022. Available from: https://copdx.org.au/wp-content/uploads/2022/12/COPDX-V2-66-Q1-2022_PUBLISHED.pdf
CD010844.pub2[4]			Australia and New Zealand	2023	Yes	The COPD-X Plan: Australian and New Zealand Guidelines for the management	Lung Foundation Australia, Thoracic Society of Australia and New Zealand	Giving information.	Yang IA, Dabscheck E, George J, McNamara R, McDonald CF, McDonald V, Smith B, Zwar N; on behalf of the Lung Foundation

Review	Revie w year	Number of citations	Guideline location	Guideline year	Different version of the same guideline cited for a review	Guideline titles	Guideline author	Use of review in guideline	Guideline citation
						of Chronic Obstructive Pulmonary Disease 2023			Australia and the Thoracic Society of Australia and New Zealand. The COPD-X Plan: Australian and New Zealand Guidelines for the management of Chronic Obstructive Pulmonary Disease 2023. Version 2.7, March 2023. Sydney, NSW, Australia: Lung Foundation Australia and Thoracic Society of Australia and New Zealand; 2023. Available from: https://copdx.org.au/wp-content/uploads/2023/09/WEBSITE_COPDX-V2-70_FINAL.pdf
CD010844.pub 2[4]			USA	2021	No	VA/DoD clinical practice guideline for the management of Chronic Obstructive Pulmonary Disease	The Management of Chronic Obstructive Pulmonary Disease Work Group	Referenced alongside a recommendatio n	The Management of Chronic Obstructive Pulmonary Disease Work Group. VA/DoD clinical practice guideline for the management of Chronic Obstructive Pulmonary Disease. Washington (DC): Department of Veterans Affairs, Department of Defense; 2021. Available from: https://www.healthquality.va.gov/guidelines/CD/copd/VADoDCOPDCPGFinal508.pdf
CD011004.pub 2[5]	2016	5	Australia	2016	No	Handbook for Non Drug Interventions: CBT for Panic Disorder	Royal Australian College of General Practitioners	In reference list but not cited.	Royal Australian College of General Practitioners. Handbook for Non Drug Interventions: CBT for Panic Disorder. East Melbourne, Victoria: The Royal Australian College of General Practitioners (RACGP); 2016. Available from: http://www.racgp.org.au/your-practice/guidelines/handi/interventi

Review	Review year	Number of citations	Guideline location	Guideline year	Different version of the same guideline cited for a review	Guideline titles	Guideline author	Use of review in guideline	Guideline citation
									ons/mental-health/cbt-for-panic-disorder/
CD011004.pub2[5]			Russia	2021	No	[Clinical guidelines: Anxiety and phobia disorders in adults]	Russian Society of Psychiatrists, (approved by the Scientific and Practical Council of the Ministry of Health of the Russian Federation)	Referenced alongside a recommendation.	Russian Society of Psychiatrists. [Clinical guidelines: Anxiety and phobia disorders in adults] 2021. Available from: https://cr.minzdrav.gov.ru/schema/455_2
CD011004.pub2[5]			Russia	2021	No	[Clinical guidelines: Panic disorder in adults]	Russian Society of Psychiatrists, (approved by the Scientific and Practical Council of the Ministry of Health of the Russian Federation)	Referenced alongside a recommendation.	Russian Society of Psychiatrists. [Clinical guidelines: Panic disorder in adults]. 2021. Available from: https://cr.minzdrav.gov.ru/schema/456_2
CD011004.pub2[5]			Finland	2019	No	[Duodecim Current Care Guidelines: Anxiety disorders]	Finnish Medical Society Duodecim, Finnish Psychiatric Association, Finnish Youth Psychiatric Association	In reference list but not cited.	Anxiety disorders. Current Care Recommendation. Finnish Medical Society Duodecim, Working group appointed by the Finnish Psychiatric Association and the Finnish Adolescent Psychiatric Association. Helsinki: Finnish Medical Society Duodecim, 2019. Available Internet: www.kaypahoito.fi
CD011004.pub2[5]			Germany	2021	No	[S3-Guideline: Treatment of anxiety disorders - Version 2]	German Society for Psychosomatic Medicine and Medical Psychotherapy	Giving information leading to recommendations.	Bandelow B, Aden I, Alpers GW, Benecke C, Deckert J, Domschke K, Eckhardt-Henn A, Geiser F, Gerlach AL, Harfst T, Hau S, Hoffmann S, Hoyer J, Hunger-Schoppe C, Kellner

Review	Review year	Number of citations	Guideline location	Guideline year	Different version of the same guideline cited for a review	Guideline titles	Guideline author	Use of review in guideline	Guideline citation
									M, Köllner V, Kopp I, Langs G, Liebeck H, Matzat J, Ohly M, Rüddel HP, Rudolf S, Scheufele E, Simon R, Staats H, Ströhle A, Waldherr B, Wedekind D, Werner AM, Wiltink J, Wolters JP, Zwanzger P, Beutel ME; German Society for Psychosomatic Medicine and Medical Psychotherapy (DGPM). [S3-Guideline: Treatment of anxiety disorders - Version 2]. Berlin: German Society for Psychosomatic Medicine and Medical Psychotherapy; 2021. Available from: https://www.awmf.org/uploads/tx_szleitlinien/051-028l_S3_Behandlung-von-Angststoerungen_2021-06.pdf
CD011412.pub4[6]	2022	4	UK	2022	No	Epilepsies in children, young people and adults	National Clinical Guideline Centre, National Institute for Health and Care Excellence (commissioner)	Collaborative/commissioned to provide evidence for the guideline.	National Clinical Guideline Centre; National Institute for Health and Care Excellence (commissioner). Epilepsies in children, young people and adults. London: National Institute for Health and Care Excellence; 2022. (NICE NG217). [Issued April 2022; updates and replaces NICE guideline CG137]. Available from: https://www.nice.org.uk/guidance/ng217
CD011412.pub4[6]			Netherlands	2023	No	[Guideline: Epilepsy]	Federation of Medical Specialists	Referenced alongside a recommendation.	Nederlandse Vereniging voor Neurologie, Dutch Society for Neurology. Epilepsie. [Guideline: Epilepsy]. Utrecht: Federatie

Review	Review year	Number of citations	Guideline location	Guideline year	Different version of the same guideline cited for a review	Guideline titles	Guideline author	Use of review in guideline	Guideline citation
									Medisch Specialisten; May 2023. Available from: http://epilepsie.neurologie.nl/
CD011412.pub4[6]			Germany	2023	No	[S1 Guidelines for diagnostics and therapy in neurology: First epileptic seizure and epilepsy in adulthood]	German Society of Neurology	Giving information leading to recommendations	Holtkamp M, May TW, Berkenfeld R, Bien CG, Coban I, Knake S, Michaelis R, Rémi J, Seeck M, Surges R, Weber Y, et al; Deutsche Gesellschaft für Neurologie. S1 Leitlinien für Diagnostik und Therapie in der Neurologie: Erster epileptischer Anfall und Epilepsien im Erwachsenenalter. [S1 Guidelines for diagnostics and therapy in neurology: First epileptic seizure and epilepsy in adulthood]. Berlin: Deutsche Gesellschaft für Neurologie (DGN); September 2023. Available from: https://register.awmf.org/assets/guidelines/030-041l_S2k_Erster-epileptischer-Anfall-Epilepsien-Erwachsenenalter_2023-09.pdf
CD011412.pub4[6]			Global	2023	No	Mental Health Gap Action Programme (mhGAP) guideline for mental, neurological and substance use disorders	World Health Organization	Referenced alongside a recommendation.	Mental Health Gap Action Programme (mhGAP) guideline for mental, neurological and substance use disorders. Geneva: World Health Organization; 2023. Licence: CC BY-NC-SA 3.0 IGO
CD011639.pub2[7]	2017	1	Germany	2023	No	[S2-guideline: liver transplantation]	German Society for Gastroenterology, Digestive and Metabolic	Giving information leading to recommendations.	German Society for Gastroenterology, Digestive and Metabolic Diseases and the German Society of General and Visceral Surgery. [S2-guideline: liver

Review	Review year	Number of citations	Guideline location	Guideline year	Different version of the same guideline cited for a review	Guideline titles	Guideline author	Use of review in guideline	Guideline citation
							Diseases and the German Society of General and Visceral Surgery.		transplantation]. December 2023. Available from: https://register.awmf.org/assets/guidelines/021-029l_S2k_Lebertransplantation_2023-12.pdf
CD011689.pub3[8]	2018	12	Russia	2022	No	[Clinical guidelines: Postpartum hemorrhage]	Russian Society of Obstetricians and Gynecologists, Association of Anesthesiologists and Resuscitators, Association of Obstetric Anesthesiologists-Resuscitators, National Association of Patient Blood Management Specialists, (approved by the Scientific and Practical Council of the Ministry of Health of the Russian Federation)	Giving information leading to recommendations	Russian Society of Obstetricians and Gynecologists, Association of Anesthesiologists and Resuscitators, Association of Obstetric Anesthesiologists-Resuscitators, National Association of Patient Blood Management Specialists, (approved by the Scientific and Practical Council of the Ministry of Health of the Russian Federation). [Clinical guidelines: Postpartum hemorrhage].2022. Available from: https://cr.minzdrav.gov.ru/schema/119_2
CD011689.pub3[8]			Germany, Austria, Switzerland	2022	No	[S2k-Guideline: Peripartum bleeding, diagnostics and therapy]	German Society of Gynaecology and Obstetrics, Austrian Society of Gynaecology and Obstetrics, Swiss	Giving information.	Annecke T, Girard T, Helmer H, von Heymann C, Kainer F, Kehl S, Korte W, Kühnert M, Lier H, Mader S, Mahnken A, Maul H, Pfanner G, Ramsell A, Schlembach D, Surbek D, Tiebel O, Zinßer L, Abou-Dakn M,

Review	Review year	Number of citations	Guideline location	Guideline year	Different version of the same guideline cited for a review	Guideline titles	Guideline author	Use of review in guideline	Guideline citation
							Society of Gynaecology and Obstetrics		Haslinger C, Henrich W, Mattern E, Schmidt M, Schöll W, Vockelmann C; German Society of Gynaecology and Obstetrics (DGGG); Austrian Society of Gynaecology and Obstetrics (OEGGG); Swiss Society of Gynaecology and Obstetrics (SGGG). [S2k-Guideline: Peripartum bleeding, diagnostics and therapy]. Berlin: German Society of Gynaecology and Obstetrics; 2022. Available from: https://www.awmf.org/uploads/tx_szleitlinien/015-063l_S2k_Peripartale_Blutungen_Diagnostik_Therapie_PPH_2022-09_2.pdf
CD011689.pub3[8]			Germany	2020	No	[S3-Guideline: Vaginal birth at term]	German Society of Gynaecology and Obstetrics, German Society for Midwifery Science	Referenced alongside a recommendation.	Abou-Dakn M, Asmushen K, Bässler-Weber S, Boes U, Bosch A, Ehm D, Fischer T, Greening M, Hartmann K, Heller G, Kapp C, von Kaisenberg C, Kayer B, Kehl S, Kranke P, Lawrenz B, Loytved C, Lütje W, Mattern E, Nielsen R, Reister F, Schäfers R, Schlösser R, Schwarz C, Stephan V, Stocker Kalberer B, Valet A, Wenk M; German Society of Gynaecology and Obstetrics (DGGG); German Society for Midwifery Science. [S3-Guideline: Vaginal birth at term]. Berlin: German Society of Gynaecology and Obstetrics; 2020. Available from: https://www.awmf.org/uploads/tx_szleitlinien/015-083l_S3_Vaginale-Geburt-am-Termin_2021-01.pdf

Review	Review year	Number of citations	Guideline location	Guideline year	Different version of the same guideline cited for a review	Guideline titles	Guideline author	Use of review in guideline	Guideline citation
CD011689.pub3[8]			Canada	2023	No	Clinical Practice Guideline: Hypertensive Disorders of Pregnancy	Association of Ontario Midwives	Giving information leading to recommendations.	Association of Ontario Midwives. Clinical Practice Guideline 15: Hypertensive Disorders of Pregnancy. Available from: https://www.ontariomidwives.ca/sites/default/files/2023-05/CPG-HDP-2023-PUB.pdf
CD011689.pub3[8]			Ireland	2022	No	National Clinical Practice Guideline: Prevention and Management of Primary Postpartum Haemorrhage	The National Women and Infants Programme (NWIHP), Institute of Obstetricians and Gynaecologists of the Royal College of Physicians of Ireland	Referenced alongside a recommendation.	Byrne B, Spring A, Barrett N, Power J, McKernan J, Brophy, D, Houston C, Faryal R, McMahon E, Manning C, Murphy P, Ni Ainle F; The National Women and Infants Programme (NWIHP); Institute of Obstetricians and Gynaecologists of the Royal College of Physicians of Ireland. National Clinical Practice Guideline: Prevention and Management of Primary Postpartum Haemorrhage. Dublin: Health Service Executive; December 2022. Available from: https://www.hse.ie/eng/about/who/acute-hospitals-division/woman-infants/clinical-guidelines/prevention-and-management-of-primary-postpartum-haemorrhage1.pdf
CD011689.pub3[8]			Canada	2022	No	Postpartum Hemorrhage and Hemorrhagic Shock	Society of Obstetricians and Gynaecologists of Canada	Cannot access	Robinson D, Basso M, Chan C, Duckitt K, Lett R; Society of Obstetricians and Gynaecologists of Canada. Postpartum Hemorrhage and Hemorrhagic Shock. (SOGC Clinical Practice Guideline no. 431). J Obstet Gynaecol Can. 2022 Dec;44(12):1293-1310.e1. doi:

Review	Review year	Number of citations	Guideline location	Guideline year	Different version of the same guideline cited for a review	Guideline titles	Guideline author	Use of review in guideline	Guideline citation
									10.1016/j.jogc.2022.10.002. Available from: https://www.jogc.com/article/S1701-2163(22)00668-5/fulltext
CD011689.pub3[8]			Australia	2019	Yes	Primary postpartum haemorrhage	Queensland Maternity and Neonatal Clinical Guidelines Program	Cannot access	Queensland Maternity and Neonatal Clinical Guidelines Program. Primary postpartum haemorrhage (MN18.1-V8-R23 replaces MN18.1-V7-R23). Brisbane, Australia: Queensland Health; 2019. [Issued March 2018; updated April 2019]. Available from: https://www.health.qld.gov.au/__data/assets/pdf_file/0015/140136/g-pph.pdf
CD011689.pub3[8]			Australia	2021	Yes	Primary postpartum haemorrhage	Queensland Maternity and Neonatal Clinical Guidelines	Referenced alongside a recommendation.	Queensland Clinical Guidelines. Postpartum haemorrhage Guideline No. MN18.1- V10-R23 Queensland Health.2021. Available from: http://www.health.qld.gov.au/qcg
CD011689.pub3[8]			UK	2019	No	Twin and triplet pregnancy	National Collaborating Centre for Women's and Children's Health, National Institute for Health and Care Excellence (commissioner)	Used to make a decision on a NICE submission.	National Collaborating Centre for Women's and Children's Health; National Institute for Health and Care Excellence (commissioner). Twin and triplet pregnancy. London: Royal College of Obstetricians and Gynaecologists; 2019 September (NICE NG137). [originally NICE CG129 issued September 2011, now replaced by NG137 September 2019. Available from: https://www.nice.org.uk/guidance/ng137
CD011689.pub3[8]			Global	2020	No	WHO recommendation on	World Health Organization	Giving information.	World Health Organization. WHO recommendation on routes of

Review	Review year	Number of citations	Guideline location	Guideline year	Different version of the same guideline cited for a review	Guideline titles	Guideline author	Use of review in guideline	Guideline citation
						routes of oxytocin administration for the prevention of postpartum haemorrhage after vaginal birth			oxytocin administration for the prevention of postpartum haemorrhage after vaginal birth. Geneva: World Health Organization; 2020. Available from: https://www.who.int/publications/i/item/9789240013926
CD011689.pub3[8]			Global	2020	No	WHO recommendation on umbilical vein injection of oxytocin for the treatment of retained placenta	World Health Organization	Giving information.	World Health Organization. WHO recommendation on umbilical vein injection of oxytocin for the treatment of retained placenta. Geneva: World Health Organization; 2020. Available from: https://www.who.int/publications/i/item/9789240013940
CD011689.pub3[8]			Global	2018	No	WHO recommendations: uterotonics for the prevention of postpartum haemorrhage (2018 update)	World Health Organization	Collaborative/commissioned to provide evidence for the guideline.	World Health Organization. WHO recommendations: uterotonics for the prevention of postpartum haemorrhage (2018 update). Geneva: World Health Organization; 2018. Available from: https://www.who.int/reproductivehealth/publications/uterotonics-pph/en/
CD011749.pub2[9]	2019	2	Germany	2020	No	[S1-Guideline: Delirium and confusional states including alcohol withdrawal delirium]	German Society of Neurology	Giving information	Maschke M. et al., Delirium and states of confusion including alcohol withdrawal delirium, S1 guideline, 2020, in: German Society of Neurology (ed.), Guidelines for Diagnostics and Therapy in Neurology. Online: www.dgn.org/leitlinien
CD011749.pub2[9]			Denmark	2021	No	[National Clinical Guideline on the	Danish Health Authority	Giving information	Danish Health Authority, National clinical guideline: Prevention and

Review	Review year	Number of citations	Guideline location	Guideline year	Different version of the same guideline cited for a review	Guideline titles	Guideline author	Use of review in guideline	Guideline citation
						prevention and treatment of delirium]			treatment of organic delirium. Copenhagen: National Board of Health, Danish Health Authority; May 2021. Available from: https://www.sst.dk/-/media/Udgivelser/2021/NKR-delirium/1_-National-klinisk-retningslinje-for-forebyggelse-og-behandling-af-organisk-delirium.ashx?la=da&hash=AC37112646602F84A7503F940D526CB493E3F267
CD011867.pub2[10]	2017	5	Germany	2019	No	[S3 Guideline: Schizophrenia]	German Society for Psychiatry and Psychotherapy, Psychosomatics and Neurology	Referenced alongside a recommendation.	German Society for Psychiatry and Psychotherapy, Psychosomatics and Neurology (DGPPN). S3 Guideline: Schizophrenia. Berlin: German Society for Psychiatry and Psychotherapy, Psychosomatics and Neurology (DGPPN); 2019 March. Available from: https://www.awmf.org/uploads/tx_szleitlinien/038-009l_S3_Schizophrenie_2019-03.pdf.
CD011867.pub2[10]			Germany	2019	No	[S3 Guideline: Psychosocial therapies for severe mental illnesses]	German Society for Psychiatry and Psychotherapy, Psychosomatics and Neurology	Giving information.	German Society for Psychiatry and Psychotherapy, Psychosomatics and Neurology (DGPPN). [S3 Guideline: Psychosocial therapies for severe mental illnesses]. Berlin: German Society for Psychiatry and Psychotherapy, Psychosomatics and Neurology (DGPPN); 2019. Available from: https://register.awmf.org/assets/guidelines/038-

Review	Review year	Number of citations	Guideline location	Guideline year	Different version of the same guideline cited for a review	Guideline titles	Guideline author	Use of review in guideline	Guideline citation
									020l_S3_Psychosoziale_Therapien_bei_schweren_psychischen_Erkrankungen_2019-07.pdf
CD011867.pub2[10]			Europe	2021	No	EPA guidance on treatment of negative symptoms in schizophrenia	European Psychiatric Association	Referenced alongside a recommendation.	Galderisi S, Kaiser S, Bitter I, Nordentoft M, Mucci A, Sabé M, Giordano GM, Nielsen MØ, Glenthøj LB, Pezzella P, Falkai P, Dollfus S, Gaebel W. EPA guidance on treatment of negative symptoms in schizophrenia. Eur Psychiatry. 2021 Mar 17;64(1):e21. doi: 10.1192/j.eurpsy.2021.13. Available from: https://www.europsy.net/app/uploads/2021/03/epa-guidance-on-treatment-of-negative-symptoms-in-schizophrenia.pdf
CD011867.pub2[10]			USA	2021	No	The American Psychiatric Association Practice Guideline for the Treatment of Patients with Schizophrenia (Third Edition)	American Psychiatric Association	Giving information leading to recommendations.	American Psychiatric Association. The American Psychiatric Association Practice Guideline for the Treatment of Patients with Schizophrenia (Third Edition). 2021. Available from: https://psychiatryonline.org/doi/pdf/10.1176/appi.books.9780890424841
CD011867.pub2[10]			Global	2022	No	WHO guidelines on mental health at work	World Health Organization	Giving information leading to recommendations.	WHO guidelines on mental health at work. Geneva: World Health Organization; 2022. Licence: CC BY-NC-SA 3.0 IGO.
CD012583.pub2[11]	2018	6	UK	2019	Yes	Clinical Knowledge Summaries: Leg ulcer - venous	National Institute for Health and Care Excellence	Cannot access	National Institute for Health and Care Excellence. Clinical Knowledge Summaries: Leg ulcer - venous.

Review	Review year	Number of citations	Guideline location	Guideline year	Different version of the same guideline cited for a review	Guideline titles	Guideline author	Use of review in guideline	Guideline citation
									London: National Institute for Health and Care Excellence; 2021. [last revised August 2021]. Available from: https://cks.nice.org.uk/topics/leg-ulcer-venous/
CD012583.pub2[11]			UK	2020	Yes	Clinical Knowledge Summaries: Leg ulcer - venous	National Institute for Health and Care Excellence	Cannot access	National Institute for Health and Care Excellence. Clinical Knowledge Summaries: Leg ulcer - venous. London: National Institute for Health and Care Excellence; 2020. [last revised February 2020]. Available from: http://cks.nice.org.uk/leg-ulcer-venou
CD012583.pub2[11]			UK	2021	Yes	Clinical Knowledge Summaries: Leg ulcer - venous	National Institute for Health and Care Excellence	Cannot access	National Institute for Health and Care Excellence. Clinical Knowledge Summaries: Leg ulcer - venous. London: National Institute for Health and Care Excellence; 2021. [last revised January 2021]. Available from: https://cks.nice.org.uk/topics/leg-ulcer-venous
CD012583.pub2[11]			UK	2021	Yes	Clinical Knowledge Summaries: Leg ulcer - venous	National Institute for Health and Care Excellence	Cannot access	National Institute for Health and Care Excellence. Clinical Knowledge Summaries: Leg ulcer - venous. London: National Institute for Health and Care Excellence; 2021. [last revised August 2021]. Available from: https://cks.nice.org.uk/topics/leg-ulcer-venous/

Review	Review year	Number of citations	Guideline location	Guideline year	Different version of the same guideline cited for a review	Guideline titles	Guideline author	Use of review in guideline	Guideline citation
CD012583.pub2[11]			UK	2023	Yes	Clinical Knowledge Summaries: Leg ulcer - venous	National Institute for Health and Care Excellence	Giving information leading to recommendations.	National Institute for Health and Care Excellence. Clinical Knowledge Summaries: Leg ulcer - venous. London: National Institute for Health and Care Excellence; 2023. [last revised December 2023]. Available from: https://cks.nice.org.uk/topics/leg-ulcer-venous/
CD012583.pub2[11]			Europe	2022	No	Editor's Choice - European Society for Vascular Surgery (ESVS) 2022 Clinical Practice Guidelines on the Management of Chronic Venous Disease of the Lower Limbs	European Society for Vascular Surgery	Giving information leading to recommendations.	De Maeseneer MG, Kakkos SK, Aherne T, Baekgaard N, Black S, Blomgren L, Giannoukas A, Gohel M, de Graaf R, Hamel-Desnos C, Jawien A, Jaworucka-Kaczorowska A, Lattimer CR, Mosti G, Noppeney T, van Rijn MJ, Stansby G, Esvs Guidelines Committee, Kolh P, Bastos Goncalves F, Chakfé N, Coscas R, de Borst GJ, Dias NV, Hinchliffe RJ, Koncar IB, Lindholt JS, Trimarchi S, Tulamo R, Twine CP, Vermassen F, Wanhainen A, Document Reviewers, Björck M, Labropoulos N, Lurie F, Mansilha A, Nyamekye IK, Ramirez Ortega M, Ulloa JH, Urbanek T, van Rij AM, Vuylsteke ME. Editor's Choice - European Society for Vascular Surgery (ESVS) 2022 Clinical Practice Guidelines on the Management of Chronic Venous Disease of the Lower Limbs. Eur J Vasc Endovasc Surg. 2022 Feb;63(2):184-267. doi: 10.1016/j.ejvs.2021.12.024.

Review	Review year	Number of citations	Guideline location	Guideline year	Different version of the same guideline cited for a review	Guideline titles	Guideline author	Use of review in guideline	Guideline citation
									Available from: https://www.ejves.com/action/showPdf?pii=S1078-5884%2821%2900979-5
CD012602.pub2[12]	2021	2	Australia	2022	No	Early pregnancy loss	Queensland Maternity and Neonatal Clinical Guidelines Program	Giving information leading to recommendations.	Queensland Maternity and Neonatal Clinical Guidelines Program. Early pregnancy loss. (MN22.29-V6-R27 replaces MN17.29-V5-R22). Brisbane: Queensland Health; 2022. [Issued 2011, amended October 2022] Available from: https://www.health.qld.gov.au/__data/assets/pdf_file/0033/139947/g-epl.pdf
CD012602.pub2[12]			UK	2023	No	Clinical Knowledge Summaries: Miscarriage	National Institute for Health and Care Excellence	Giving information	National Institute for Health and Care Excellence. Clinical Knowledge Summaries: Miscarriage. London: National Institute for Health and Care Excellence; 2023. [last revised October 2023]. Available from: https://cks.nice.org.uk/topics/miscarriage/
CD012620.pub2[13]	2018	20	UK	2019	Yes	Chronic obstructive pulmonary disease in over 16s: diagnosis and management	National Clinical Guideline Centre, National Institute for Health and Care Excellence (commissioner)	Not cited in the NICE guideline (but may have been referenced in supporting evidence).	National Clinical Guideline Centre; National Institute for Health and Care Excellence (commissioner). Chronic obstructive pulmonary disease in over 16s: diagnosis and management. London: Royal College of Physicians of London; 2019 July (NICE NG115). [issued December 2018, updated July 2019]. Available from: https://www.nice.org.uk/guidance/ng115

Review	Review year	Number of citations	Guideline location	Guideline year	Different version of the same guideline cited for a review	Guideline titles	Guideline author	Use of review in guideline	Guideline citation
CD012620.pub2[13]			Finland	2019	Yes	[Duodecim Current Care Guidelines: Chronic Obstructive Pulmonary Disease]	Finnish Medical Society Duodecim, Finnish Association of Respiratory Physicians	Cannot access.	Duodecim Current Care: COPD. [Duodecim Current Care Guidelines: Chronic Obstructive Pulmonary Disease]. Working group appointed by the Finnish Medical Society Duodecim, the Finnish Association of Pulmonologists, Finnish Medical Society Duodecim, Finnish Association of Respiratory Physicians. Helsinki: Finnish Medical Society Duodecim, 2019. Available from: https://www.kaypahoito.fi/hoi06040
CD012620.pub2[13]			Finland	2020	Yes	[Duodecim Current Care Guidelines: Chronic obstructive pulmonary disease]	Finnish Medical Society Duodecim, Finnish Association of Respiratory Physicians	Giving information	COPD. Current Care Recommendation. Appointed by the Finnish Medical Society Duodecim and the Finnish Association of Pulmonologists working group. Helsinki: Finnish Medical Society Duodecim, 2020. Available online: www.kaypahoito.fi
CD012620.pub2[13]			Netherlands	2021	Yes	[Dutch College of General Practitioners – Guideline on COPD (version 5.0 – Guideline M26)]	Dutch College of General Practitioners working group.	Cannot access.	Bischoff E, Bouma M, Broekhuizen L, Donkers J, Hallensleben C, De Jong J, Snoeck-Stroband J, In 't Veen JC, Van Vugt S, Wagenaar M, NHG Werkgroep. NHG-Standaard COPD (versie 5.0 – NHG Standaard M26). [Dutch College of General Practitioners – Guideline on COPD (version 5.0 – Guideline M26)]. Utrecht: Nederlands Huisartsen Genootschap; April 2021. Available from:

Review	Review year	Number of citations	Guideline location	Guideline year	Different version of the same guideline cited for a review	Guideline titles	Guideline author	Use of review in guideline	Guideline citation
									https://richtlijnen.nhg.org/files/pdf/102_COPD_april-2021.pdf
CD012620.pub2[13]			Netherlands	2022	Yes	[Dutch College of General Practitioners – Guideline on COPD (version 5.1 - Guideline M26]	Dutch College of General Practitioners working group.	Giving information	Bischoff E, Bouma M, Broekhuizen L, Donkers J, Hallensleben C, De Jong J, Snoeck-Stroband J, In ‘t Veen JC, Van Vugt S, Wagenaar M, NHG Werkgroep. NHG-Standaard COPD (versie 5.1 - NHG Standaard M26). [Dutch College of General Practitioners – Guideline on COPD (version 5.1 - Guideline M26]. Utrecht: Nederlands Huisartsen Genootschap; December 2022. Available from: https://richtlijnen.nhg.org/files/pdf/102_COPD_december-2022.pdf
CD012620.pub2[13]			Germany	2021	No	[National Care Guideline COPD. 2nd Edition, Version 1]	German Medical Association, Association of German Medical Associations, National Association of Statutory Health Insurance Physicians, Association of Scientific Medical Societies.	Giving information	German Medical Association (BÄK), National Association of Statutory Health Insurance Physicians (KBV), Association of Scientific Medical Societies (AWMF). National Care Guideline COPD – Partial publication of the Long version, 2nd edition. Version 1. 2021. DOI: 10.6101/AZQ/000477. www.leitlinien.de/copd.
CD012620.pub2[13]			UK	2018	Yes	Chronic obstructive pulmonary disease in over 16s: diagnosis and management	National Clinical Guideline Centre, National Institute for Health and	Cannot access.	National Clinical Guideline Centre; National Institute for Health and Care Excellence (commissioner). Chronic obstructive pulmonary disease in over 16s: diagnosis and

Review	Review year	Number of citations	Guideline location	Guideline year	Different version of the same guideline cited for a review	Guideline titles	Guideline author	Use of review in guideline	Guideline citation
							Care Excellence (commissioner)		management. London: National Clinical Guideline Centre, Royal College of Physicians; 2018 (NICE NG115). [Issued December 2018]. Available from: https://www.nice.org.uk/guidance/ng115.
CD012620.pub2[13]			Global	2023	No	Global strategy for the diagnosis, management and prevention of chronic obstructive pulmonary disease: 2023 report	Global Initiative for Chronic Obstructive Lung Disease	Referenced alongside a recommendation	Global Initiative for Chronic Obstructive Lung Disease. Global strategy for the diagnosis, management and prevention of chronic obstructive pulmonary disease: 2023 report. Illinois, USA: Global Initiative for Chronic Obstructive Lung Disease (GOLD); 2023. Available from: https://goldcopd.org/2023-gold-report-2/
CD012620.pub2[13]			Ireland	2021	No	Management of Chronic Obstructive Pulmonary Disease (COPD)	COPD Guideline Development Group	Referenced alongside a recommendation	COPD Guideline Development Group. Management of Chronic Obstructive Pulmonary Disease (COPD). (NCEC National Clinical Guideline No. 27). Dublin: Department of Health; 2021. Available from: https://www.gov.ie/en/publication/5df41-national-clinical-guideline-no27-management-of-chronic-obstructive-pulmonary-disease-copd/
CD012620.pub2[13]			UK	2023	No	Technology appraisal guidance: Tofacitinib for treating active	National Institute for Health and Care Excellence	Not cited in the NICE guideline (but may have been	National Institute for Health and Care Excellence. Technology appraisal guidance: Tofacitinib for treating active ankylosing

Review	Review year	Number of citations	Guideline location	Guideline year	Different version of the same guideline cited for a review	Guideline titles	Guideline author	Use of review in guideline	Guideline citation
						ankylosing spondylitis		referenced in supporting evidence).	spondylitis. London: National Institute for Health and Care Excellence; 2023. (NICE TA920). [Issued October 2023]. Available from: https://www.nice.org.uk/guidance/ta920
CD012620.pub2[13]			UK	2022	No	Technology appraisal guidance: Upadacitinib for treating active ankylosing spondylitis	National Institute for Health and Care Excellence	Used to make decision on a NICE submission.	National Institute for Health and Care Excellence. Technology appraisal guidance: Upadacitinib for treating active ankylosing spondylitis. London: National Institute for Health and Care Excellence; 2022. (NICE TA829). [Issued September 2022]. Available from: https://www.nice.org.uk/guidance/ta829
CD012620.pub2[13]			Australia and New Zealand	2019	Yes	The COPD-X Plan: Australian and New Zealand Guidelines for the management of Chronic Obstructive Pulmonary Disease 2019	Lung Foundation Australia, Thoracic Society of Australia and New Zealand	Cannot access	Yang IA, Dabscheck E, George J, Jenkins S, McDonald CF, McDonald V, Smith B, Zwar N; on behalf of the Lung Foundation Australia and the Thoracic Society of Australia and New Zealand. The COPD-X Plan: Australian and New Zealand Guidelines for the management of Chronic Obstructive Pulmonary Disease 2019. Version 2.59, August 2019. Sydney, NSW, Australia: Lung Foundation Australia and Thoracic Society of Australia and New Zealand; 2019. Available from: https://copdx.org.au/wp-

Review	Review year	Number of citations	Guideline location	Guideline year	Different version of the same guideline cited for a review	Guideline titles	Guideline author	Use of review in guideline	Guideline citation
									content/uploads/2019/11/COPDX-V2-59-Aug-2019-FINAL2.pdf
CD012620.pub2[13]			Australia and New Zealand	2020	Yes	The COPD-X Plan: Australian and New Zealand Guidelines for the management of Chronic Obstructive Pulmonary Disease 2020	Lung Foundation Australia, Thoracic Society of Australia and New Zealand	Giving information	Yang IA, Dabscheck E, George J, Jenkins S, McDonald CF, McDonald V, Smith B, Zwar N; on behalf of the Lung Foundation Australia and the Thoracic Society of Australia and New Zealand. The COPD-X Plan: Australian and New Zealand Guidelines for the management of Chronic Obstructive Pulmonary Disease 2020. Version 2.62, October 2020. Sydney, NSW, Australia: Lung Foundation Australia and Thoracic Society of Australia and New Zealand; 2020. Available from: https://copdx.org.au/wp-content/uploads/2021/02/COPDX-V2.62-June_Oct-2020-PUBLISHED.pdf
CD012620.pub2[13]			Australia and New Zealand	2021	Yes	The COPD-X Plan: Australian and New Zealand Guidelines for the management of Chronic Obstructive Pulmonary Disease 2021	Lung Foundation Australia, Thoracic Society of Australia and New Zealand	Giving information	Yang IA, Dabscheck E, George J, Jenkins S, McDonald CF, McDonald V, Smith B, Zwar N; on behalf of the Lung Foundation Australia and the Thoracic Society of Australia and New Zealand. The COPD-X Plan: Australian and New Zealand Guidelines for the management of Chronic Obstructive Pulmonary Disease 2021. Version 2.63, February 2021. Sydney, NSW, Australia: Lung Foundation Australia and Thoracic Society of Australia and New Zealand; 2021. Available from:

Review	Revie w year	Number of citations	Guideline location	Guideline year	Different version of the same guideline cited for a review	Guideline titles	Guideline author	Use of review in guideline	Guideline citation
									https://copdx.org.au/wp-content/uploads/2021/04/COPDX-V2-63-Feb-2021_FINAL-PUBLISHED.pdf
CD012620.pub2[13]			Australia and New Zealand	2022	Yes	The COPD-X Plan: Australian and New Zealand Guidelines for the management of Chronic Obstructive Pulmonary Disease 2022	Lung Foundation Australia, Thoracic Society of Australia and New Zealand	Giving information	Yang IA, Dabscheck E, George J, Jenkins S, McDonald CF, McDonald V, Smith B, Zwar N; on behalf of the Lung Foundation Australia and the Thoracic Society of Australia and New Zealand. The COPD-X Plan: Australian and New Zealand Guidelines for the management of Chronic Obstructive Pulmonary Disease 2022. Version 2.66, April 2022. Sydney, NSW, Australia: Lung Foundation Australia and Thoracic Society of Australia and New Zealand; 2022. Available from: https://copdx.org.au/wp-content/uploads/2022/12/COPDX-V2-66-Q1-2022_PUBLISHED.pdf
CD012620.pub2[13]			Australia and New Zealand	2023	Yes	The COPD-X Plan: Australian and New Zealand Guidelines for the management of Chronic Obstructive Pulmonary Disease 2023	Lung Foundation Australia, Thoracic Society of Australia and New Zealand	Giving information	Yang IA, Dabscheck E, George J, McNamara R, McDonald CF, McDonald V, Smith B, Zwar N; on behalf of the Lung Foundation Australia and the Thoracic Society of Australia and New Zealand. The COPD-X Plan: Australian and New Zealand Guidelines for the management of Chronic Obstructive Pulmonary Disease 2023. Version 2.7, March 2023. Sydney, NSW, Australia: Lung Foundation Australia and Thoracic Society of Australia and

Review	Review year	Number of citations	Guideline location	Guideline year	Different version of the same guideline cited for a review	Guideline titles	Guideline author	Use of review in guideline	Guideline citation
									New Zealand; 2023. Available from: https://copdx.org.au/wp-content/uploads/2023/09/WEBSITE_COPDX-V2-70_FINAL.pdf
CD012620.pub2[13]			Taiwan	2021	No	Update on guidelines for the treatment of COPD in Taiwan using evidence and GRADE system-based recommendations	Cheng, Lin, Chu, Chiu, Lin, Lin, Ko, Chen, Chen, Sheu, Huang, Yang, Wei, Chien, Wang, Lin	Giving information leading to recommendations	Cheng SL, Lin CH, Chu KA, Chiu KL, Lin SH, Lin HC, Ko HK, Chen YC, Chen CH, Sheu CC, Huang WC, Yang TM, Wei YF, Chien JY, Wang HC, Lin MC. Update on guidelines for the treatment of COPD in Taiwan using evidence and GRADE system-based recommendations. J Formos Med Assoc. 2021 Oct;120(10):1821-1844. doi: 10.1016/j.jfma.2021.06.007. Available from: https://www.sciencedirect.com/science/article/pii/S0929664621002850?via%3Dihub
CD012620.pub2[13]			USA	2021	No	VA/DoD clinical practice guideline for the management of Chronic Obstructive Pulmonary Disease	The Management of Chronic Obstructive Pulmonary Disease Work Group	Referenced alongside a recommendation	The Management of Chronic Obstructive Pulmonary Disease Work Group. VA/DoD clinical practice guideline for the management of Chronic Obstructive Pulmonary Disease. Washington (DC): Department of Veterans Affairs, Department of Defense; 2021. Available from: https://www.healthquality.va.gov/guidelines/CD/copd/VADoDCOPDCPGFinal508.pdf
CD012620.pub2[13]			UK	2020	Yes	Venous thromboembolic diseases: diagnosis, management and	National Clinical Guideline Centre, National Institute for Health and	Cannot access.	National Clinical Guideline Centre; National Institute for Health and Clinical Excellence (commissioner). Venous thromboembolic diseases:

Review	Revie w year	Number of citations	Guideline location	Guideline year	Different version of the same guideline cited for a review	Guideline titles	Guideline author	Use of review in guideline	Guideline citation
						thrombophilia testing	Clinical Excellence (commissioner)		diagnosis, management and thrombophilia testing. London: National Institute for Health and Care Excellence; 2020. (NICE NG158). [Issued June 2012; last updated March 2020]. Available from: https://www.nice.org.uk/guidance/ng158
CD012620.pub 2[13]			UK	2023	Yes	Venous thromboembolic diseases: diagnosis, management and thrombophilia testing	National Clinical Guideline Centre, National Institute for Health and Clinical Excellence	Not cited in the NICE guideline (but may have been referenced in supporting evidence).	National Clinical Guideline Centre; National Institute for Health and Clinical Excellence (commissioner). Venous thromboembolic diseases: diagnosis, management and thrombophilia testing. London: National Institute for Health and Care Excellence; 2023. (NICE NG158). [Issued June 2012; last updated August 2023]. Available from: https://www.nice.org.uk/guidance/ng158
CD012859.pub 2[14]	2020	1	Germany	2023	No	[S3-Guideline: Perioperative Management of gastrointestinal tumours (POMGAT)]	Guideline Program Oncology of the Association of Scientific Scientists Medical Societies, German Cancer Society and the German Cancer Aid Foundation.	Referenced alongside a recommendation.	Oncology Guideline Program (German Cancer Society, German Cancer Aid, AWMF): Perioperative Management of Gastrointestinal Tumors (POMGAT), Long version 1.0, 2023, AWMF registration number: 088-010OL https://www.leitlinienprogramm-onkologie.de/leitlinien/perioperatives-managementbei-gastrointestinalen-tumoren-pomgat

Review	Review year	Number of citations	Guideline location	Guideline year	Different version of the same guideline cited for a review	Guideline titles	Guideline author	Use of review in guideline	Guideline citation
CD013103.pub2[15]	2019	2	Finland	2020	No	[Duodecim Current Care Guidelines: Acute kidney injury]	Working group appointed by the Finnish Medical Society Duodecim, the Finnish Society of Anaesthesiology, the Intensive Care Medicine Subdivision and the Finnish Nephrological Society	Giving information	Kidney damage (acute). Current Care Recommendation. Finnish Medical Society Duodecim, Finnish Association of Anaesthesiologists, Intensive Care Medicine working group appointed by the subdivision and the Finnish Nephrological Society. Helsinki: Finnish Finnish Medical Society Duodecim, 2020. Available online: www.kaypahoito.fi
CD013103.pub2[15]			China	2022	No	Practice guidance for the use of terlipressin for liver cirrhosis-related complications	Hepatobiliary Study Group of the Chinese Society of Gastroenterology of the Chinese Medical Association, Hepatology Committee of the Chinese Research Hospital Association	Giving information	Qi X, Bai Z, Zhu Q, et al. Practice guidance for the use of terlipressin for liver cirrhosis–related complications. Therapeutic Advances in Gastroenterology. 2022;15. doi:10.1177/17562848221098253
CD013121.pub2[16]	2021	2	Europe	2023	No	Management of severe peri-operative bleeding: Guidelines from the European Society of Anaesthesiology and Intensive Care: Second update 2022	European Society of Anaesthesiology and Intensive Care	Giving information leading to recommendations	Kietaibl S, Ahmed A, Afshari A, Albaladejo P, Aldecoa C, Barauskas G, De Robertis E, Faraoni D, Filipescu DC, Fries D, Godier A, Haas T, Jacob M, Lancé MD, Llau JV, Meier J, Molnar Z, Mora L, Rahe-Meyer N, Samama CM, Scarlatescu E, Schlimp C, Wikkelsø AJ, Zacharowski K. Management of severe peri-

Review	Review year	Number of citations	Guideline location	Guideline year	Different version of the same guideline cited for a review	Guideline titles	Guideline author	Use of review in guideline	Guideline citation
									operative bleeding: Guidelines from the European Society of Anaesthesiology and Intensive Care: Second update 2022. Eur J Anaesthesiol. 2023 Apr 1;40(4):226-304. doi: 10.1097/EJA.0000000000001803. Available from: https://journals.lww.com/ejanaesthesiology/fulltext/2023/04000/management_of_severe_peri_operative_bleeding_.2.aspx
CD013121.pub2[16]			UK	2023	No	Cirrhosis in over 16s: Assessment and management	National Clinical Guideline Centre (hosted by the Royal College of Physicians), National Institute for Health and Care Excellence (commissioner)	Not cited in the NICE guideline (referenced in supporting evidence).	National Clinical Guideline Centre; National Institute for Health and Care Excellence (commissioner). Cirrhosis in over 16s: Assessment and management. London: Royal College of Physicians of London; 2023 September (NICE NG50). [issued July 2016, updated September 2023]. Available from: https://www.nice.org.uk/guidance/ng50
CD013122.pub2[17]	2021	1	Europe	2023	No	Management of severe peri-operative bleeding: Guidelines from the European Society of Anaesthesiology and Intensive Care: Second update 2022	European Society of Anaesthesiology and Intensive Care	Giving information leading to recommendations.	Kietaibl S, Ahmed A, Afshari A, Albaladejo P, Aldecoa C, Barauskas G, De Robertis E, Faraoni D, Filipescu DC, Fries D, Godier A, Haas T, Jacob M, Lancé MD, Llau JV, Meier J, Molnar Z, Mora L, Rahe-Meyer N, Samama CM, Scarlatescu E, Schlimp C, Wikkelsø AJ, Zacharowski K. Management of severe peri-operative bleeding: Guidelines from the European Society of

Review	Review year	Number of citations	Guideline location	Guideline year	Different version of the same guideline cited for a review	Guideline titles	Guideline author	Use of review in guideline	Guideline citation
									Anaesthesiology and Intensive Care: Second update 2022. Eur J Anaesthesiol. 2023 Apr 1;40(4):226-304. doi: 10.1097/EJA.0000000000001803. Available from: https://journals.lww.com/ejanaesthesiology/fulltext/2023/04000/management_of_severe_peri_operative_bleeding_.2.aspx
CD013125.pub2[18]	2020	1	UK	2023	No	**Cirrhosis in over 16s: Assessment and management**	National Clinical Guideline Centre (hosted by the Royal College of Physicians), National Institute for Health and Care Excellence (commissioner)	Not cited in the NICE guideline (but may have been referenced in supporting evidence).	National Clinical Guideline Centre; National Institute for Health and Care Excellence (commissioner). Cirrhosis in over 16s: Assessment and management. London: Royal College of Physicians of London; 2023 September (NICE NG50). [issued July 2016, updated September 2023]. Available from: https://www.nice.org.uk/guidance/ng50
CD013198.pub2[19]	2021	2	Australia and New Zealand	2022	Yes	The COPD-X Plan: Australian and New Zealand Guidelines for the management of Chronic Obstructive Pulmonary Disease 2022	Lung Foundation Australia, Thoracic Society of Australia and New Zealand	Giving information.	Yang IA, Dabscheck E, George J, Jenkins S, McDonald CF, McDonald V, Smith B, Zwar N; on behalf of the Lung Foundation Australia and the Thoracic Society of Australia and New Zealand. The COPD-X Plan: Australian and New Zealand Guidelines for the management of Chronic Obstructive Pulmonary Disease 2022. Version 2.66, April 2022. Sydney, NSW, Australia: Lung Foundation Australia and Thoracic Society of Australia and New

Review	Review year	Number of citations	Guideline location	Guideline year	Different version of the same guideline cited for a review	Guideline titles	Guideline author	Use of review in guideline	Guideline citation
									Zealand; 2022. Available from: https://copdx.org.au/wp-content/uploads/2022/12/COPDX-V2-66-Q1-2022_PUBLISHED.pdf
CD013198.pub2[19]			Australia and New Zealand	2023	Yes	The COPD-X Plan: Australian and New Zealand Guidelines for the management of Chronic Obstructive Pulmonary Disease 2023	Lung Foundation Australia, Thoracic Society of Australia and New Zealand	Giving information.	Yang IA, Dabscheck E, George J, McNamara R, McDonald CF, McDonald V, Smith B, Zwar N; on behalf of the Lung Foundation Australia and the Thoracic Society of Australia and New Zealand. The COPD-X Plan: Australian and New Zealand Guidelines for the management of Chronic Obstructive Pulmonary Disease 2023. Version 2.7, March 2023. Sydney, NSW, Australia: Lung Foundation Australia and Thoracic Society of Australia and New Zealand; 2023. Available from: https://copdx.org.au/wp-content/uploads/2023/09/WEBSITE_COPDX-V2-70_FINAL.pdf
CD013203.pub2[20]	2020	1	USA	2023	No	Dual-Organ Transplantation: Indications, Evaluation, and Outcomes for Heart-Kidney and Heart-Liver Transplantation: A Scientific Statement From the American Heart Association	American Heart Association Heart Failure and Transplantation Committee of the Council on Clinical Cardiology, Council on the Kidney in Cardiovascular Disease, Council on Cardiovascular Surgery and Anesthesia,	Referenced alongside a recommendation.	Kittleson MM, Sharma K, Brennan DC, Cheng XS, Chow SL, Colvin M, DeVore AD, Dunlay SM, Fraser M, Garonzik-Wang J, Khazanie P, Korenblat KM, Pham DT; American Heart Association Heart Failure and Transplantation Committee of the Council on Clinical Cardiology; Council on the Kidney in Cardiovascular Disease; Council on Cardiovascular Surgery and Anesthesia; Council on Cardiovascular and Stroke Nursing;

Review	Review year	Number of citations	Guideline location	Guideline year	Different version of the same guideline cited for a review	Guideline titles	Guideline author	Use of review in guideline	Guideline citation
							Council on Cardiovascular and Stroke Nursing, Council on Quality of Care and Outcomes Research, Council on Lifelong Congenital Heart Disease and Heart Health in the Young		Council on Quality of Care and Outcomes Research; and Council on Lifelong Congenital Heart Disease and Heart Health in the Young. Dual-Organ Transplantation: Indications, Evaluation, and Outcomes for Heart-Kidney and Heart-Liver Transplantation: A Scientific Statement From the American Heart Association. Circulation. 2023 Aug 15;148(7):622-636. doi: 10.1161/CIR.0000000000001155. Available from: https://www.ahajournals.org/doi/epdf/10.1161/CIR.0000000000001155
CD013206.pub2[21]	2020	2	UK	2022	No	Technology appraisal guidance: Abrocitinib, tralokinumab or upadacitinib for treating moderate to severe atopic dermatitis	National Institute for Health and Care Excellence	Used to make decision on a NICE company submission.	National Institute for Health and Care Excellence. Technology appraisal guidance: Abrocitinib, tralokinumab or upadacitinib for treating moderate to severe atopic dermatitis. London: National Institute for Health and Care Excellence; 2022. (NICE TA814). [Issued August 2022]. Available from: https://www.nice.org.uk/guidance/ta814/evidence
CD013206.pub2[21]			Taiwan	2022	No	Taiwan guidelines for the diagnosis and management of pediatric atopic dermatitis: Consensus statement of the	Committee of the Taiwan Academy of Pediatric Allergy, Asthma and Immunology	Giving information leading to recommendations.	Yao TC, Wang IJ, Sun HL, Ou LS, Yu HH, Wang L, Hung CH; Committee of the Taiwan Academy of Pediatric Allergy, Asthma and Immunology. Taiwan guidelines for the diagnosis and management of pediatric atopic dermatitis: Consensus statement of

Review	Review year	Number of citations	Guideline location	Guideline year	Different version of the same guideline cited for a review	Guideline titles	Guideline author	Use of review in guideline	Guideline citation
						Taiwan Academy of Pediatric Allergy, Asthma and Immunology			the Taiwan Academy of Pediatric Allergy, Asthma and Immunology. J Microbiol Immunol Infect. 2022 Aug;55(4):561-572. doi: 10.1016/j.jmii.2022.03.004. Available from: https://www.sciencedirect.com/science/article/pii/S1684118222000512?via%3Dihub
CD013487[22]	2019	1	Germany	2022	No	[S3-Guideline: Diagnostics, therapy and aftercare for patients with monoclonal gammopathy of undetermined significance (MGUS) or multiple myeloma]	Guideline Program Oncology of the Association of Scientific Scientists Medical Societies, German Cancer Society and the German Cancer Aid Foundation.	Referenced alongside a recommendation.	Oncology Guideline Program (German Cancer Society; German Cancer Aid; AWMF). [S3-Guideline: Diagnostics, therapy and aftercare for patients with monoclonal gammopathy of undetermined significance (MGUS) or multiple myeloma]. Berlin: Oncology Guideline Program; 2022. Available from: https://www.awmf.org/uploads/tx_szleitlinien/018-035OLl_S3_Diagnostik-Therapie-Nachsorge-monoklonaler-Gammopathie-unklarer-Signifikanz-MGUS-Multiplem-Myelom_2022-02.pdf
CD013674.pub2**[23]**	2021	1	Global	2023	No	**Mental Health Gap Action Programme (mhGAP) guideline for mental, neurological and substance use disorders**	World Health Organization	Referenced alongside a recommendation.	Mental Health Gap Action Programme (mhGAP) guideline for mental, neurological and substance use disorders. Geneva: World Health Organization; 2023. Licence: CC BY-NC-SA 3.0 IGO

Review	Review year	Number of citations	Guideline location	Guideline year	Different version of the same guideline cited for a review	Guideline titles	Guideline author	Use of review in guideline	Guideline citation
CD013792.pub2[24]	2021	6	UK	2021	Yes	Ectopic pregnancy and miscarriage: diagnosis and initial management	National Guideline Alliance (hosted by the Royal College of Obstetricians and Gynaecologists), National Institute for Health and Care Excellence (commissioner)	Cannot access	National Guideline Alliance (hosted by the Royal College of Obstetricians and Gynaecologists), National Institute for Health and Care Excellence (commissioner). Ectopic pregnancy and miscarriage: diagnosis and initial management. London: National Institute for Health and Care Excellence; 2021. (NICE NG126). [Issued November 2021; updates and replaces NICE CG154). Available from: https://www.nice.org.uk/guidance/ng126
CD013792.pub2[24]			Russia	2022	No	[Clinical guidelines: Recurrent miscarriage]	Russian Society of Obstetricians and Gynecologists, (approved by the Scientific and Practical Council of the Ministry of Health of the Russian Federation)	Referenced alongside a recommendation.	Russian Society of Obstetricians and Gynecologists, (approved by the Scientific and Practical Council of the Ministry of Health of the Russian Federation). [Clinical guidelines: Recurrent miscarriage]. 2022. Available from: https://cr.minzdrav.gov.ru/schema/721_1
CD013792.pub2[24]			Germany	2024	No	[S2e guideline: First Trimester Diagnostics and Therapy @ 11-13+6 weeks of pregnancy]	German Society for Ultrasound in Medicine, German Society for Gynaecology and Obstetrics.	Giving information	Kaisenberg C, Kozlowski P, Kagan KO, Hoopmann M, Heling KS, Chaoui R, Klaritsch P, Pertl B, Burkhardt T, Tercanli S, Frenzel J, C. Mundlos.AWMF 085-002 S2e LL First Trimester Diagnostics and Therapy @ 11-13+6Weeks of pregnancy Available at: https://register.awmf.org/de/leitlinien/detail/085-00

Review	Review year	Number of citations	Guideline location	Guideline year	Different version of the same guideline cited for a review	Guideline titles	Guideline author	Use of review in guideline	Guideline citation
CD013792.pub2[24]			Germany, Austria, Switzerland	2022	No	[S2k Guideline: Diagnosis and treatment of women with repeated spontaneous abortions]	German Society of Gynecology and Obstetrics, Austrian Society of Gynecology and Obstetrics, Swiss Society of Gynecology and Obstetrics, German Society of Gynecology and Obstetrics	Giving information leading to recommendations.	German Society of Gynaecology and Obstetrics (DGGG); Austrian Society of Gynaecology and Obstetrics (OEGGG); Swiss Society of Gynaecology and Obstetrics (SGGG). [S2k Guideline: Diagnosis and treatment of women with repeated spontaneous abortions]. Berlin: German Society of Gynaecology and Obstetrics (DGGG); 2022. Available from: https://www.awmf.org/uploads/tx_szleitlinien/015-050l_S2k_Diagnostik-Therapie-wiederholte-Spontanaborte_2022-05_01.pdf
CD013792.pub2[24]			UK	2023	Yes	Ectopic pregnancy and miscarriage: diagnosis and initial management	National Guideline Alliance (hosted by the Royal College of Obstetricians and Gynaecologists), National Institute for Health and Care Excellence (commissioner)	Collaborative/commissioned to provide evidence for the guideline.	National Guideline Alliance (hosted by the Royal College of Obstetricians and Gynaecologists) // National Institute for Health and Care Excellence (commissioner). Ectopic pregnancy and miscarriage: diagnosis and initial management. London: National Institute for Health and Care Excellence; 2023. (NICE NG126). [Issued November 2021; updated August 2023). Available from: https://www.nice.org.uk/guidance/ng126
CD013792.pub2[24]			Ireland	2023	No	National Clinical Practice Guideline: Recurrent Miscarriage	The National Women and Infants Programme (NWIHP), Institute of Obstetricians	Giving information	Linehan L, Hennessy M, Khalid A, Whelan J, O'Donoghue K; The National Women and Infants Programme (NWIHP); Institute of Obstetricians and Gynaecologists of

Review	Review year	Number of citations	Guideline location	Guideline year	Different version of the same guideline cited for a review	Guideline titles	Guideline author	Use of review in guideline	Guideline citation
							and Gynaecologists of the Royal College of Physicians of Ireland		the Royal College of Physicians of Ireland. National Clinical Practice Guideline: Recurrent Miscarriage. Dublin: Health Service Executive; January 2023. Available from: https://www.hse.ie/eng/about/who/acute-hospitals-division/woman-infants/clinical-guidelines/recurrent-miscarriage.pdf
CD013856.pub2[25]	2023	2	Germany	2023	No	[S2k-Guidelines for diagnostics and therapy in neurology: Parkinson's Disease]	German Society of Neurology	Giving information.	Höglinger G., Trenkwalder C. et al., Parkinson's disease, S2k guideline, 2023, in: German Society of Neurology (Ed.), Guidelines for Diagnostics and therapy in neurology. Online: www.dgn.org/leitlinien
CD013856.pub2**[25]**			Global	2023	No	**Clinical Knowledge Summaries: Parkinson's disease**	National Institute for Health and Care Excellence	Referenced alongside a recommendation.	National Institute for Health and Care Excellence. Clinical Knowledge Summaries: Parkinson's disease. London: National Institute for Health and Care Excellence; 2023. [last revised August 2023]. Available from: https://cks.nice.org.uk/topics/parkinsons-disease/
CD014978.pub2[26]	2022	1	Global	2022	No	WHO recommendation on Tocolytic therapy for improving preterm birth outcomes	World Health Organization	Collaborative/commissioned to provide evidence for the guideline.	World Health Organization. WHO recommendation on Tocolytic therapy for improving preterm birth outcomes. Geneva: World Health Organization; 2022. Available from: https://www.who.int/publications/i/item/9789240057227

1. Scheiman M, Kulp MT, Cotter SA, et al. Interventions for convergence insufficiency: a network meta-analysis. *Cochrane Database Syst Rev* 2020(12) doi: 10.1002/14651858.CD006768.pub3
2. Walsh T, Worthington HV, Glenny AM, Marinho VCC, Jeroncic A. Fluoride toothpastes of different concentrations for preventing dental caries. *Cochrane Database Syst Rev* 2019(3) doi: 10.1002/14651858.CD007868.pub3
3. de Bastos M, Stegeman BH, Rosendaal FR, et al. Combined oral contraceptives: venous thrombosis. *Cochrane Database Syst Rev* 2014(3) doi: 10.1002/14651858.CD010813.pub2
4. Kew KM, Dias S, Cates CJ. Long-acting inhaled therapy (beta-agonists, anticholinergics and steroids) for COPD: a network meta-analysis. *Cochrane Database Syst Rev* 2014(3) doi: 10.1002/14651858.CD010844.pub2
5. Pompoli A, Furukawa TA, Imai H, et al. Psychological therapies for panic disorder with or without agoraphobia in adults: a network meta-analysis. *Cochrane Database Syst Rev* 2016(4) doi: 10.1002/14651858.CD011004.pub2
6. Nevitt SJ, Sudell M, Cividini S, Marson AG, Tudur Smith C. Antiepileptic drug monotherapy for epilepsy: a network meta-analysis of individual participant data. *Cochrane Database Syst Rev* 2022(4) doi: 10.1002/14651858.CD011412.pub4
7. Rodríguez-Perálvarez M, Guerrero-Misas M, Thorburn D, et al. Maintenance immunosuppression for adults undergoing liver transplantation: a network meta-analysis. *Cochrane Database Syst Rev* 2017(3) doi: 10.1002/14651858.CD011639.pub2
8. Gallos ID, Papadopoulou A, Man R, et al. Uterotonic agents for preventing postpartum haemorrhage: a network meta-analysis. *Cochrane Database Syst Rev* 2018(12) doi: 10.1002/14651858.CD011689.pub3
9. Burry L, Hutton B, Williamson DR, et al. Pharmacological interventions for the treatment of delirium in critically ill adults. *Cochrane Database Syst Rev* 2019(9) doi: 10.1002/14651858.CD011749.pub2
10. Suijkerbuijk YB, Schaafsma FG, van Mechelen JC, et al. Interventions for obtaining and maintaining employment in adults with severe mental illness, a network meta-analysis. *Cochrane Database Syst Rev* 2017(9) doi: 10.1002/14651858.CD011867.pub2
11. Norman G, Westby MJ, Rithalia AD, et al. Dressings and topical agents for treating venous leg ulcers. *Cochrane Database Syst Rev* 2018(6) doi: 10.1002/14651858.CD012583.pub2
12. Ghosh J, Papadopoulou A, Devall AJ, et al. Methods for managing miscarriage: a network meta-analysis. *Cochrane Database Syst Rev* 2021(6) doi: 10.1002/14651858.CD012602.pub2
13. Oba Y, Keeney E, Ghatehorde N, Dias S. Dual combination therapy versus long-acting bronchodilators alone for chronic obstructive pulmonary disease (COPD): a systematic review and network meta-analysis. *Cochrane Database Syst Rev* 2018(12) doi: 10.1002/14651858.CD012620.pub2
14. Weibel S, Rücker G, Eberhart LHJ, et al. Drugs for preventing postoperative nausea and vomiting in adults after general anaesthesia: a network meta-analysis. *Cochrane Database Syst Rev* 2020(10) doi: 10.1002/14651858.CD012859.pub2
15. Best LMJ, Freeman SC, Sutton AJ, et al. Treatment for hepatorenal syndrome in people with decompensated liver cirrhosis: a network meta-analysis. *Cochrane Database Syst Rev* 2019(9) doi: 10.1002/14651858.CD013103.pub2
16. Roccarina D, Best LMJ, Freeman SC, et al. Primary prevention of variceal bleeding in people with oesophageal varices due to liver cirrhosis: a network meta-analysis. *Cochrane Database Syst Rev* 2021(4) doi: 10.1002/14651858.CD013121.pub2
17. Plaz Torres M, Best LMJ, Freeman SC, et al. Secondary prevention of variceal bleeding in adults with previous oesophageal variceal bleeding due to decompensated liver cirrhosis: a network meta-analysis. *Cochrane Database Syst Rev* 2021(3) doi: 10.1002/14651858.CD013122.pub2
18. Komolafe O, Roberts D, Freeman SC, et al. Antibiotic prophylaxis to prevent spontaneous bacterial peritonitis in people with liver cirrhosis: a network meta-analysis. *Cochrane Database Syst Rev* 2020(1) doi: 10.1002/14651858.CD013125.pub2

19. Janjua S, Mathioudakis AG, Fortescue R, et al. Prophylactic antibiotics for adults with chronic obstructive pulmonary disease: a network meta-analysis. *Cochrane Database Syst Rev* 2021(1) doi: 10.1002/14651858.CD013198.pub2
20. Best LMJ, Leung J, Freeman SC, et al. Induction immunosuppression in adults undergoing liver transplantation: a network meta-analysis. *Cochrane Database Syst Rev* 2020(1) doi: 10.1002/14651858.CD013203.pub2
21. Sawangjit R, Dilokthornsakul P, Lloyd-Lavery A, et al. Systemic treatments for eczema: a network meta-analysis. *Cochrane Database Syst Rev* 2020(9) doi: 10.1002/14651858.CD013206.pub2
22. Piechotta V, Jakob T, Langer P, et al. Multiple drug combinations of bortezomib, lenalidomide, and thalidomide for first-line treatment in adults with transplant-ineligible multiple myeloma: a network meta-analysis. *Cochrane Database Syst Rev* 2019(11) doi: 10.1002/14651858.CD013487
23. Hetrick SE, McKenzie JE, Bailey AP, et al. New generation antidepressants for depression in children and adolescents: a network meta-analysis. *Cochrane Database Syst Rev* 2021(5) doi: 10.1002/14651858.CD013674.pub2
24. Devall AJ, Papadopoulou A, Podesek M, et al. Progestogens for preventing miscarriage: a network meta-analysis. *Cochrane Database Syst Rev* 2021(4) doi: 10.1002/14651858.CD013792.pub2
25. Ernst M, Folkerts AK, Gollan R, et al. Physical exercise for people with Parkinson's disease: a systematic review and network meta-analysis. *Cochrane Database Syst Rev* 2023(1) doi: 10.1002/14651858.CD013856.pub2
26. Wilson A, Hodgetts-Morton VA, Marson EJ, et al. Tocolytics for delaying preterm birth: a network meta-analysis (0924). *Cochrane Database Syst Rev* 2022(8) doi: 10.1002/14651858.CD014978.pub2
