# Supplementary material for: The impact of Cochrane Reviews that apply network meta-analysis in clinical guidelines: A systematic review
Source: PLoS One. 2024 Dec 26;19(12):e0315563. doi: 10.1371/journal.pone.0315563 (PMC11671017; doi:10.1371/journal.pone.0315563)
Supplement: S4 Table — (PDF) [file pone.0315563.s010.pdf]

Table S4: Data extracted for pairwise meta-analysis reviews that were not cited in guidelines

Matched pair-wise meta-analyses	Eligibility criteria	Analysis
**Review ID:** CD009710.pub3[1]<br><br>**Review Group:** Gut<br><br>**Date:** 4 September 2019<br><br>**Sources of support:** University of Leeds; Homeopathy Research Institute, UK; ScHARR, UK.<br><br>**Matched NMA:** CD013210.pub2[2]	**Studies:** RCTs, quasi-RCTs, cohort and case-control studies.<br><br>**Patients:** patients with irritable bowel syndrome.<br><br>**Interventions analysed for primary outcome:** homeopathy versus placebo; homeopathy versus usual care; homeopathy plus usual care versus usual care; homeopathy plus usual care versus supportive listening plus usual care (5).<br><br>**Outcome:** global improvement of symptoms (first primary). Sub-grouped by Asafoetida only versus Asafoetida + nux vom.	**Outcome type and measure:** dichotomous; risk ratio.<br>**Number of trials:** 4<br>**Number of patients:** 307<br>**Type of analysis:** Frequentist.<br><br>**Heterogeneity assessed:** Yes (I square, chi-square).<br>**Heterogeneity found:** no.<br><br>**Grade classification:** very low, low.<br><br>**ROB (SG/AC):** low and unclear.
**Review ID:** CD010735.pub3[3]<br><br>**Review Group:** Eyes & Vision<br><br>**Date:** 23 June 2023<br><br>**Sources of support:** National Institute for Health Research; Public Health Agency.<br><br>**Matched NMA:** CD007419.pub7[4]	**Studies:** RCTs<br><br>**Patients:** people with age-related cataract<br><br>**Interventions analysed for primary outcome:** Laser-assisted cataract surgery versus standard ultrasound phacoemulsification cataract surgery (2).<br><br>**Outcome:** intraoperative complications (first primary). Sub-grouped by anterior capsule tear versus posterior capsule tear.	**Outcome type and measure:** dichotomous; peto odds ratio.<br>**Number of trials:** 42<br>**Number of patients:** 5831<br>**Type of analysis:** Frequentist.<br><br>**Heterogeneity assessed:** Yes (I square, chi-square).<br>**Heterogeneity found:** no.<br><br>**Grade classification: l**ow.<br><br>**ROB (SG/AC):** cannot be extracted for specific analysis but all trials are low or unclear.
**Review ID:** CD012478.pub2[5]<br><br>**Review Group:** Kidney & Transplant<br><br>Date: 22 February 2023<br><br>**Sources of support:** Sheffield Kidney Institute, UK; University of Sheffield; Baxter Clinical Evidence Council Grant, UK.	**Studies:** RCTs, quasi-RCTs.<br><br>**Patients:** patients requiring peritoneal dialysis catheter placement<br><br>**Interventions analysed for primary outcome:**<br>laparoscopic versus open surgical PD catheter insertion; medical versus open surgical PD catheter insertion; percutaneous versus open surgical PD catheter insertion; peritoneoscopic versus open surgical PD catheter insertion (5).	**Outcome type and measure:** dichotomous; risk ratio.<br>**Number of trials:** 17<br>**Number of patients:** 1305<br>**Type of analysis:** Frequentist.<br><br>**Heterogeneity assessed:** Yes (I square).<br>**Heterogeneity found:** no.<br><br>**Grade classification:** very low, low.

Matched pair-wise meta-analyses	Eligibility criteria	Analysis
**Matched NMA:** CD010590.pub3[6]	**Outcome:** Early PD catheter function (first primary).	**ROB (SG/AC):** cannot be extracted for specific analysis but some trials were high risk.
**Review ID:** CD013245.pub2[7]<br><br>**Review Group:** Urology<br><br>Date: 12 December 2020<br><br>**Sources of support:** University of Minnesota, USA.<br><br>**Matched NMA:** CD013020.pub2[8]	**Studies:** RCTs<br><br>**Patients:** men with metastatic, hormone-sensitive prostate cancer<br><br>**Interventions analysed for primary outcome:** Abiraterone + androgen deprivation therapy versus androgen deprivation therapy alone in metastatic (2).<br><br>**Outcome:** Time to death due to any cause (first primary).	**Outcome type and measure:** time to event; hazard ratio.<br>**Number of trials**: 2<br>**Number of patients:** 2201<br>**Type of analysis:** Frequentist.<br><br>**Heterogeneity assessed:** Yes (I square, confidence interval overlap).<br>**Heterogeneity found:** no.<br><br>**Grade classification:** high.<br><br>**ROB (SG/AC):** low.
**Review ID:** CD014806[9]<br><br>**Review Group:** Neonatal<br><br>**Date:** 30 August 2023<br><br>**Sources of support:** University of Toronto; UTCSP Pain Scientist Scholarship; The Hospital for Sick Children, Canada; The SickKids Summer Research Program; Canadian Institutes of Health Research; Vermont Oxford Network, USA.<br><br>**Matched NMA:** CD013730.pub2[10]	**Studies:** RCTs<br><br>**Patients:** neonate infants, with maximum postnatal age of 28 days after reaching 40 weeks' postmenstrual age.<br><br>**Interventions analysed for primary outcome:** sucrose (any concentration, any dose, with or without NNS) compared to control (water, placebo, or no intervention); sucrose (any concentration, any dose) compared to NNS; sucrose (any concentration, any dose) + NNS compared to NNS (with or without water) (4).<br><br>**Outcome:** Premature Infant Pain Profile (first primary). Sub-grouped by pre-term and post-term and time points.	**Outcome type and measure:** continuous; mean difference.<br>**Number of trials:** 55<br>**Number of patients:** 6273<br>**Type of analysis:** Frequentist.<br><br>**Heterogeneity assessed:** Yes (I square).<br>**Heterogeneity found:** yes.<br><br>**Grade classification:** very low, low, moderate.<br><br>**ROB (SG/AC):** Some high risk in analysis.
**Review ID:** CD012858.pub2[11]<br><br>**Review Group:** Gynaecology & Fertility	**Studies:** RCTs, cohort studies (both retrospective and prospective) and case-control studies.	**Outcome type and measure:** continuous; mean difference.<br>**Number of trials:** 7<br>**Number of patients:** 250<br>**Type of analysis:** Frequentist.

Matched pair-wise meta-analyses	Eligibility criteria	Analysis
**Date:**.28 August 2019<br><br>**Sources of support:** not reported.<br><br>**Matched NMA:** CD012692.pub2[12]	**Patients:** premenopausal women undergoing hysterectomy for benign gynaecological indications<br><br>**Interventions analysed for primary outcome:** hysterectomy with opportunistic salpingectomy compared with hysterectomy without opportunistic salpingectomy (2).<br><br>**Outcome:** postoperative hormonal status (secondary, no meta-analysis for preceding outcomes.	**Heterogeneity assessed:** Yes (I square).<br>**Heterogeneity found:** no.<br><br>**Grade classification:** low.<br><br>**ROB (SG/AC):** low or unclear.
**Review ID:** CD004135.pub4[13]<br><br>**Review Group:** Urology<br><br>**Date:** 28 June 2021<br><br>**Sources of support:** University of Minnesota; Instituto Universitario Hospital Italiano, Argentina.<br><br>**Matched NMA:** CD013656.pub2[14]	**Studies:** parallel-group RCTs and cluster-RCTs<br><br>**Patients:** men with lower urinary tract symptoms due to benign prostatic hyperplasia.<br><br>**Interventions analysed for primary outcome:** transurethral microwave thermotherapy compared to transurethral resection of the prostate; transurethral microwave thermotherapy compared to sham treatment (3).<br><br>**Outcome:** Urologic symptom scores (first primary).	**Outcome type and measure:** continuous; mean difference.<br>**Number of trials:** 16<br>**Number of patients:** 1919<br>**Type of analysis:** Frequentist.<br><br>**Heterogeneity assessed:** Yes (I square).<br>**Heterogeneity found:** no.<br><br>**Grade classification:** moderate.<br><br>**ROB (SG/AC):** cannot be extracted for specific analysis but all trials are low or unclear.
**Review ID:** CD005612.pub5 [15]<br><br>**Review Group:** Epilepsy<br><br>**Date:** 29 March 2022<br><br>**Sources of support:** National Institute for Health Research<br><br>**Matched NMA:** CD011412.pub4[16]	**Studies:** RCTs (including cross-over)<br><br>**Patients:** drug-resistant focal epilepsy<br><br>**Interventions analysed for primary outcome:**<br>Pregabalin compared to placebo; Pregabalin compared to any active comparator (gabapentin, lamotrigine and levetiracetam) (3).<br><br>**Outcome:** 50% or greater reduction in seizure frequency (first primary).	**Outcome type and measure:** dichotomous; risk ratio.<br>**Number of trials:** 11<br>**Number of patients:** 3949<br>**Type of analysis:** Frequentist.<br><br>**Heterogeneity assessed:** Yes (I square, chi-square).<br>**Heterogeneity found:** yes.<br><br>**Grade classification:** low.<br><br>**ROB (SG/AC):** cannot be extracted for specific analysis but all trials are low or unclear.

Matched pair-wise meta-analyses	Eligibility criteria	Analysis
**Review ID:** CD011899.pub2[17]<br><br>**Review Group:** Work<br><br>**Date:** 30 August 2017<br><br>**Sources of support:** Khon Kaen University, Thailand; Norwegian Institute of Public Health, Norway; Hue University of Medicine and Pharmacy, Vietnam; University Hospital of Brest, France; Thailand Research Fund, Thailand.<br><br>**Matched NMA:** CD011867.pub2[18]	**Studies:** RCTs<br><br>**Patients:** participants were full-time, part-time, or self-employed working individuals over 18 years of age.<br><br>**Interventions analysed for primary outcome:** computer-based interventions compared to in-person interventions (2).<br><br>**Outcome:** stress (first primary).	**Outcome type and measure:** continuous; standardised mean difference.<br>**Number of trials:** 2<br>**Number of patients:** 159<br>**Type of analysis:** Frequentist.<br><br>**Heterogeneity assessed:** Yes (I square).<br>**Heterogeneity found:** no.<br><br>**Grade classification:** Very low.<br><br>**ROB (SG/AC):** low.
**Review ID:** CD013756.pub2[19]<br><br>**Review Group:** Pain, Palliative & Supportive Care<br><br>**Date:** 2 December 2021<br><br>**Sources of support:** National Institute for Health Research.<br><br>**Matched NMA:** CD012775.pub2[20]	**Studies:** RCTs of parallel, cross-over, or cluster design<br><br>**Patients: adults with chronic pain**<br><br>**Interventions analysed for primary outcome:** active stimulation versus placebo; active stimulation + other intervention versus other intervention alone (4).<br><br>**Outcome:** Pain intensity (first primary). Sub-grouped by short, medium and long term follow up.	**Outcome type and measure:** continuous; mean difference.<br>**Number of trials:** 15<br>**Number of patients:** 908<br>**Type of analysis:** Frequentist.<br><br>**Heterogeneity assessed:** Yes (I square, chi-square).<br>**Heterogeneity found:** yes.<br><br>**Grade classification:** Very low, low.<br><br>**ROB (SG/AC):** some high risk in analysis.
**Review ID:** CD012863.pub2[21]<br><br>**Review Group:** Gynaecological, Neuro-oncology & Orphan Cancer<br><br>**Date:** 25 January 2021	**Studies:** RCTs<br><br>**Patients:** women with early-stage cervical cancer undergoing radical hysterectomy	**Outcome type and measure:** continuous; mean difference.<br>**Number of trials:** 4<br>**Number of patients:** 214<br>**Type of analysis:** Frequentist.<br><br>**Heterogeneity assessed:** Yes (I square, chi-square).<br>**Heterogeneity found:** no

Matched pair-wise meta-analyses	Eligibility criteria	Analysis
**Sources of support:** National Institute for Health Research.<br><br>**Matched NMA:** CD013579.pub2[22]	**Interventions analysed for primary outcome:** bethanechol versus placebo; suprapubic catheterisation versus intermittent self-catheterisation (4).<br><br>**Outcome:** Time after surgery to a post-void residual urine volume of 100 mL or less (days) (secondary outcome, no meta-analysis for preceding outcomes.	**Grade classification:** not rated.<br><br>**ROB (SG/AC):** low and unclear.
**Review ID:** CD000093.pub6 [23]<br><br>**Review Group:** Bone, Joint & Muscle Trauma<br><br>**Date:** 26 January 2022<br><br>**Sources of support:** National Institute for Health Research<br><br>**Matched NMA:** CD013405.pub2[24]	**Studies:** RCTs and quasi-RCTs.<br><br>**Patients:** older adults with stable or unstable extracapsular hip fractures<br><br>**Interventions analysed for primary outcome:** Cephalomedullary nails compared to extramedullary implants (2).<br><br>**Outcome:** Activities of daily living (first primary).	**Outcome type and measure:** continuous; standardised mean difference.<br>**Number of trials:** 76<br>**Number of patients:** 10,979<br>**Type of analysis:** Frequentist.<br><br>**Heterogeneity assessed:** Yes (I square).<br>**Heterogeneity found:** yes.<br><br>**Grade classification:** very low.<br><br>**ROB (SG/AC):** some high risk in analysis.
**Review ID:** CD013107.pub2[25]<br><br>**Review Group:** Hepato-Biliary<br><br>Date: 22 August 2019<br><br>**Sources of support:** Danish State; 2017 Open-end Fund of Education Ministry Key Laboratory for Research and Application of 'Zang Xiang' Theory in Liaoning University of Traditional Chinese Medicine (zyzx1702), China; Science and Technology Research Project of Department of Education of Liaoning	**Studies:** RCTs<br><br>**Patients:** chronic hepatitis B<br><br>**Interventions analysed for primary outcome:** acupuncture compared with no intervention (2).<br><br>**Outcome:** Proportion of participants with one or more serious adverse events (second primary, no data for first primary).	**Outcome type and measure:** dichotomous; risk ratio.<br>**Number of trials:** 8<br>**Number of patients:** 555.<br>**Type of analysis:** Frequentist.<br><br>**Heterogeneity assessed:** Yes (I square, chi-square).<br>**Heterogeneity found:** no.<br><br>**Grade classification:** Very low.<br><br>**ROB (SG/AC):** low and unclear.

Matched pair-wise meta-analyses	Eligibility criteria	Analysis
Province (L201713), China'; National Center for Complementary and Integrative Health, USA.<br><br>**Matched NMA:** CD013103.pub2[26]		
**Review ID: C**D012648.pub3[27]<br><br>**Review Group:** Eyes & Vision<br><br>**Date:** 27 January 2023<br><br>**Sources of support:** National Institutes of Health, USA; Queen's University Belfast, UK.<br><br>**Matched NMA:** CD014758.pub2[28]	**Studies:** RCTs<br><br>**Patients:** people (> 30 years) with cataract and presbyopia<br><br>**Interventions analysed for primary outcome:** trifocal intraocular lenses versus bifocal intraocular lenses (2).<br><br>**Outcome:** mean uncorrected (without the aid of spectacles or contact lenses) distance visual acuity measured by logarithm of the minimum angle of resolution (LogMAR) chart at one-year follow-up (first primary).	**Outcome type and measure:** continuous; mean difference.<br>**Number of trials:** 7<br>**Number of patients:** 331<br>**Type of analysis:** Frequentist.<br><br>**Heterogeneity assessed:** Yes (I square, chi-square).<br>**Heterogeneity found:** no.<br><br>**Grade classification:** low.<br><br>**ROB (SG/AC):** low and unclear.

Abbreviations: AC: allocation concealment; NMA: network meta-analysis; RCT: randomised controlled trial; SG: sequence generation.

1. Peckham EJ, Cooper K, Roberts ER, et al. Homeopathy for treatment of irritable bowel syndrome. *Cochrane Database Syst Rev* 2019(9) doi: 10.1002/14651858.CD009710.pub3
2. Iheozor-Ejiofor Z, Gordon M, Clegg A, et al. Interventions for maintenance of surgically induced remission in Crohn's disease: a network meta-analysis. *Cochrane Database Syst Rev* 2019(9) doi: 10.1002/14651858.CD013210.pub2
3. Narayan A, Evans JR, O'Brart D, et al. Laser-assisted cataract surgery versus standard ultrasound phacoemulsification cataract surgery. *Cochrane Database Syst Rev* 2023(6) doi: 10.1002/14651858.CD010735.pub3
4. Virgili G, Curran K, Lucenteforte E, Peto T, Parravano M. Anti-vascular endothelial growth factor for diabetic macular oedema: a network meta-analysis. *Cochrane Database Syst Rev* 2023(6) doi: 10.1002/14651858.CD007419.pub7
5. Briggs VR, Jacques RM, Fotheringham J, et al. Catheter insertion techniques for improving catheter function and clinical outcomes in peritoneal dialysis patients. *Cochrane Database Syst Rev* 2023(2) doi: 10.1002/14651858.CD012478.pub2
6. Chung EYM, Palmer SC, Saglimbene VM, et al. Erythropoiesis-stimulating agents for anaemia in adults with chronic kidney disease: a network meta-analysis. *Cochrane Database Syst Rev* 2023(2) doi: 10.1002/14651858.CD010590.pub3

7. Sathianathen NJ, Oestreich MC, Brown S, et al. Abiraterone acetate in combination with androgen deprivation therapy compared to androgen deprivation therapy only for metastatic hormone-sensitive prostate cancer. *Cochrane Database Syst Rev* 2020(12) doi: 10.1002/14651858.CD013245.pub2
8. Jakob T, Tesfamariam YM, Macherey S, et al. Bisphosphonates or RANK-ligand-inhibitors for men with prostate cancer and bone metastases: a network meta-analysis. *Cochrane Database Syst Rev* 2020(12) doi: 10.1002/14651858.CD013020.pub2
9. Yamada J, Bueno M, Santos L, et al. Sucrose analgesia for heel-lance procedures in neonates. *Cochrane Database Syst Rev* 2023(8) doi: 10.1002/14651858.CD014806
10. Hay S, Ovelman C, Zupancic JAF, et al. Systemic corticosteroids for the prevention of bronchopulmonary dysplasia, a network meta-analysis. *Cochrane Database Syst Rev* 2023(8) doi: 10.1002/14651858.CD013730.pub2
11. van Lieshout LAM, Steenbeek MP, De Hullu JA, et al. Hysterectomy with opportunistic salpingectomy versus hysterectomy alone. *Cochrane Database Syst Rev* 2019(8) doi: 10.1002/14651858.CD012858.pub2
12. Wang R, Danhof NA, Tjon-Kon-Fat RI, et al. Interventions for unexplained infertility: a systematic review and network meta-analysis. *Cochrane Database Syst Rev* 2019(9) doi: 10.1002/14651858.CD012692.pub2
13. Franco JVA, Garegnani L, Escobar Liquitay CM, Borofsky M, Dahm P. Transurethral microwave thermotherapy for the treatment of lower urinary tract symptoms in men with benign prostatic hyperplasia. *Cochrane Database Syst Rev* 2021(6) doi: 10.1002/14651858.CD004135.pub4
14. Franco JVA, Jung JH, Imamura M, et al. Minimally invasive treatments for lower urinary tract symptoms in men with benign prostatic hyperplasia: a network meta-analysis. *Cochrane Database Syst Rev* 2021(7) doi: 10.1002/14651858.CD013656.pub2
15. Panebianco M, Bresnahan R, Marson AG. Pregabalin add-on for drug-resistant focal epilepsy. *Cochrane Database Syst Rev* 2022(3) doi: 10.1002/14651858.CD005612.pub5
16. Nevitt SJ, Sudell M, Cividini S, Marson AG, Tudur Smith C. Antiepileptic drug monotherapy for epilepsy: a network meta-analysis of individual participant data. *Cochrane Database Syst Rev* 2022(4) doi: 10.1002/14651858.CD011412.pub4
17. Kuster AT, Dalsbø TK, Luong Thanh BY, et al. Computer-based versus in-person interventions for preventing and reducing stress in workers. *Cochrane Database Syst Rev* 2017(8) doi: 10.1002/14651858.CD011899.pub2
18. Suijkerbuijk YB, Schaafsma FG, van Mechelen JC, et al. Interventions for obtaining and maintaining employment in adults with severe mental illness, a network meta-analysis. *Cochrane Database Syst Rev* 2017(9) doi: 10.1002/14651858.CD011867.pub2
19. O'Connell NE, Ferraro MC, Gibson W, et al. Implanted spinal neuromodulation interventions for chronic pain in adults. *Cochrane Database Syst Rev* 2021(12) doi: 10.1002/14651858.CD013756.pub2
20. Piechotta V, Adams A, Haque M, et al. Antiemetics for adults for prevention of nausea and vomiting caused by moderately or highly emetogenic chemotherapy: a network meta-analysis. *Cochrane Database Syst Rev* 2021(11) doi: 10.1002/14651858.CD012775.pub2
21. Aue-aungkul A, Kietpeerakool C, Rattanakanokchai S, et al. Postoperative interventions for preventing bladder dysfunction after radical hysterectomy in women with early-stage cervical cancer. *Cochrane Database Syst Rev* 2021(1) doi: 10.1002/14651858.CD012863.pub2
22. McBain C, Lawrie TA, Rogozińska E, et al. Treatment options for progression or recurrence of glioblastoma: a network meta-analysis. *Cochrane Database Syst Rev* 2021(1) doi: 10.1002/14651858.CD013579.pub2

23. Lewis SR, Macey R, Gill JR, Parker MJ, Griffin XL. Cephalomedullary nails versus extramedullary implants for extracapsular hip fractures in older adults. *Cochrane Database Syst Rev* 2022(1) doi: 10.1002/14651858.CD000093.pub6
24. Lewis SR, Macey R, Lewis J, et al. Surgical interventions for treating extracapsular hip fractures in older adults: a network meta-analysis. *Cochrane Database Syst Rev* 2022(2) doi: 10.1002/14651858.CD013405.pub2
25. Kong DZ, Liang N, Yang GL, et al. Acupuncture for chronic hepatitis B. *Cochrane Database Syst Rev* 2019(8) doi: 10.1002/14651858.CD013107.pub2
26. Best LMJ, Freeman SC, Sutton AJ, et al. Treatment for hepatorenal syndrome in people with decompensated liver cirrhosis: a network meta-analysis. *Cochrane Database Syst Rev* 2019(9) doi: 10.1002/14651858.CD013103.pub2
27. Zamora-de La Cruz D, Bartlett J, Gutierrez M, Ng SM. Trifocal intraocular lenses versus bifocal intraocular lenses after cataract extraction among participants with presbyopia. *Cochrane Database Syst Rev* 2023(1) doi: 10.1002/14651858.CD012648.pub3
28. Lawrenson JG, Shah R, Huntjens B, et al. Interventions for myopia control in children: a living systematic review and network meta-analysis. *Cochrane Database Syst Rev* 2023(2) doi: 10.1002/14651858.CD014758.pub2
