# Supplementary material for: The impact of Cochrane Reviews that apply network meta-analysis in clinical guidelines: A systematic review
Source: PLoS One. 2024 Dec 26;19(12):e0315563. doi: 10.1371/journal.pone.0315563 (PMC11671017; doi:10.1371/journal.pone.0315563)
Supplement: S5 Table — (PDF) [file pone.0315563.s011.pdf]

Table S5: Data extracted for pairwise meta-analysis reviews that were cited in guidelines

Matched pair-wise meta-analyses	Eligibility criteria	Analysis
**Review ID:** CD002958.pub2[1]<br><br>**Review Group:** Pregnancy & Childbirth<br><br>**Date:** 08 June 2021<br><br>**Sources of support:** National Institute for Health Research; La Trobe University, Australia; Royal Women's Hospital, Australia; Murdoch Childrens Research Institute, Australia.<br><br>**Matched NMA:** CD012602.pub2[2]	**Studies:** RCTs, quasi-RCTs, cluster RCTs.<br><br>**Patients:** healthy mothers and term infants.<br><br>**Interventions analysed for primary outcome:** Early discharge compared to standard discharge (2).<br><br>**Outcome:** Infants readmitted for neonatal morbidity (first primary). Sub-grouped by time points.	**Outcome type and measure:** dichotomous; risk ratio.<br>**Number of trials:** 17<br>**Number of patients:** 9409<br>**Type of analysis:** Frequentist.<br><br>**Heterogeneity assessed:** Yes (I square).<br>**Heterogeneity found:** no.<br><br>**Grade classification:** moderate.<br><br>**ROB (SG/AC): s**ome high risk in analysis.
**Review ID:** CD004112.pub4[3]<br><br>**Review Group:** Fertility Regulation<br><br>**Date:** 30 March 2014<br><br>**Sources of support:** U.S. Agency for International Development, USA; National Institute of Child Health and Human Development, USA.<br><br>**Matched NMA:** CD010813.pub2[4]	**Studies:** RCTs<br><br>**Patients:** Men of reproductive age undergoing vasectomy for sterilization.<br><br>**Interventions analysed for primary outcome:** no-scalpel and the incisional method (2).<br><br>**Outcome:** hematoma (first primary).	**Outcome type and measure:** dichotomous; peto odds ratio.<br>**Number of trials:** 2<br>**Number of patients:** 1529<br>**Type of analysis:** Frequentist.<br><br>**Heterogeneity assessed:** not reported methods (reported statistics in forest plots).<br>**Heterogeneity found:** no.<br><br>**Grade classification:** not rated.<br><br>**ROB (SG/AC):** low or unclear.
**Review ID:** CD006764.pub4[5]<br><br>**Review Group:** Pregnancy & Childbirth<br><br>**Date:** 9 August 2022<br><br>**Sources of support:** National Institute for Health Research. ARCH, The Robinson	**Studies:** RCTs and quasi-RCTs.<br><br>**Patients:** women at risk of preterm birth.<br><br>**Interventions analysed for primary outcome:**<br>Dexamethasone compared to betamethasone;<br>Dexamethasone: oral compared to intramuscular;	**Outcome type and measure:** dichotomous; risk ratio.<br>**Number of trials:** 11<br>**Number of patients:** 2762<br>**Type of analysis:** Frequentist.<br><br>**Heterogeneity assessed:** Yes (I square, tausq, chi-square).<br>**Heterogeneity found:** no.

Matched pair-wise meta-analyses	Eligibility criteria	Analysis
Institute, Discipline of Obstetrics and Gynaecology, The University of Adelaide, Australia; Department of Health and Ageing, Australia; National Health and Medical Research Council, Australia; World Health Organization; and the UNDP-UNFPA-UNICEF-WHO-World Bank Special Programme of Research, Development and Research Training in Human Reproduction (HRP) Switzerland.<br><br>**Matched NMA:** CD014978.pub2[6]	Betamethasone acetate + phosphate compared to betamethasone phosphate;<br>Betamethasone 12-hourly compared to 24-hourly dosing (8).<br><br>**Outcome:** Any known death after randomisation (a primary, no meta-analysis for preceding outcomes).	**Grade classification:** very low, moderate.<br><br>**ROB (SG/AC):** low or unclear.
**Review ID:** CD006922.pub4[7]<br><br>**Review Group:** Airways<br><br>**Date:** 3 December 2018<br><br>**Sources of support:** National Institute for Health Research; NHS R&D, UK.; European Union.<br><br>**Matched NMA:** CD012620.pub2[8]	**Studies:** RCTs<br><br>**Patients:** adults and children with chronic asthma<br><br>**Interventions analysed for primary outcome:** regular salmeterol in addition to regular inhaled corticosteroid (2).<br><br>**Outcome:** All-cause mortality (first primary). Sub-grouped by adults vs children.	**Outcome type and measure:** dichotomous; odds ratio.<br>**Number of trials:** 49<br>**Number of patients:** 36,404<br>**Type of analysis:** Frequentist.<br><br>**Heterogeneity assessed:** Yes (I square).<br>**Heterogeneity found:** no.<br><br>**Grade classification:** moderate.<br><br>**ROB (SG/AC):** low and unclear.
**Review ID:** CD009951.pub3[9]<br><br>**Review Group:** Pregnancy & Childbirth<br><br>Date: 19 April 2021<br><br>**Sources of support:** National Institute for Health Research; The University of Queensland, Australia; Royal Brisbane and Women's Hospital, Australia; University of Melbourne, Australia; Mater Health,	**Studies:** RCTs and cluster-RCTs.<br><br>**Patients:** preventing gestational diabetes<br><br>**Interventions analysed for primary outcome:** Probiotics compared to placebo (2).<br><br>**Outcome:** Diagnosis of gestational diabetes mellitus (first primary).	**Outcome type and measure:** dichotomous; risk ratio.<br>**Number of trials:** 7<br>**Number of patients:** 1647<br>**Type of analysis:** Frequentist.<br><br>**Heterogeneity assessed:** Yes (I square, chi-square tausq).<br>**Heterogeneity found:** yes.<br><br>**Grade classification:** Low.<br><br>**ROB (SG/AC): low**

Matched pair-wise meta-analyses	Eligibility criteria	Analysis
Australia; NHMRC Early Career Research Fellowship, Australia<br><br>**Matched NMA:** CD013792.pub2[10]		
**Review ID:** CD010204.pub2[11]<br><br>**Review Group:** Common Mental Disorders<br><br>Date: 4 April 2016<br><br>**Sources of support:** National Institute for Health Research.<br><br>**Matched NMA:**<br>CD011004.pub2[12]	**Studies:** RCTs and cluster-RCTs.<br><br>**Patients: I**ndividuals with post-traumatic stress disorder and comorbid substance use disorder<br><br>**Interventions analysed for primary outcome:** Trauma-focused psychological therapy compared to control intervention; Trauma-focused psychological therapy compared to active psychological therapy for SUD only; Non-trauma-focused psychological therapy for PTSD and SUD or PTSD only compared to control intervention (4).<br><br>**Outcome:** Severity of traumatic stress symptoms (first primary).	**Outcome type and measure:** continuous; standardised mean difference.<br>**Number of trials:** 14<br>**Number of patients:** 1506<br>**Type of analysis:** Frequentist.<br><br>**Heterogeneity assessed:** Yes (I square, chi-square).<br>**Heterogeneity found:** no.<br><br>**Grade classification:** Very low, low.<br><br>**ROB (SG/AC):** cannot be extracted for specific analysis but all trials are low or unclear.
**Review ID:** CD010526.pub3[13]<br><br>**Review Group:** Oral Health<br><br>**Date:** 5 March 2019<br><br>**Sources of support:** New York University College of Dentistry; Cochrane Oral Health Global Alliance (American Association of Public Health Dentistry; AS-Akademie, Germany; the British Association for the Study of Community Dentistry; the British Society of Paediatric Dentistry; the Canadian Dental Hygienists Association; the Centre for Dental Education and Research at All India Institute of Medical Sciences; the National Center for Dental Hygiene Research &	**Studies:** RCTs<br><br>**Patients:** patients requiring Class I or Class II resin-based composite restorations<br><br>**Interventions analysed for primary outcome:** Liner versus no liner (2).<br><br>**Outcome:** Postoperative hypersensitivity (first primary).	**Outcome type and measure:** dichotomous; risk ratio.<br>**Number of trials:** 7<br>**Number of patients: '**over 700'.<br>**Type of analysis:** Frequentist.<br><br>**Heterogeneity assessed:** Yes (I square, chi-square).<br>**Heterogeneity found:** no.<br><br>**Grade classification**: low.<br><br>**ROB (SG/AC):** cannot be extracted for specific analysis but all trials are low or unclear.

Matched pair-wise meta-analyses	Eligibility criteria	Analysis
Practice, USA; New York University College of Dentistry, USA; NHS Education for Scotland, UK; and the Swiss Society for Endodontology, Switzerland); National Institute for Health Research.<br><br>**Matched NMA:** CD007868.pub3[14]		
**Review ID:** CD011710.pub3[15]<br><br>**Review Group:** Common Mental Disorders<br><br>**Date:** 20 May 2021<br><br>**Sources of support:** National Institute for Health Research; Cardiff University; National Centre of Mental Health, UK.<br><br>**Matched NMA:** CD013674.pub2**[16]**	**Studies:** RCTs, randomised crossover trials, and cluster-randomised trials<br><br>**Patients:** adults aged ≥ 16 years with PTSD<br><br>**Interventions analysed for primary outcome:** Internet-based cognitive and behavioural therapy compared to face-to-face non-CBT; Internet-based cognitive and behavioural therapy compared to wait list for post-traumatic stress disorder in adults; Internet-based cognitive and behavioural therapy compared to I-non-C/BT (4).<br><br>**Outcome:** Severity of PTSD symptoms (first primary).	**Outcome type and measure:** continuous; standardised mean difference.<br>**Number of trials:** 13<br>**Number of patients:** 808<br>**Type of analysis:** Frequentist.<br><br>**Heterogeneity assessed:** Yes (I square, chi-square).<br>**Heterogeneity found:** yes.<br><br>**Grade classification:** Very low.<br><br>**ROB (SG/AC):** cannot be extracted for specific analysis but all trials are low or unclear.
**Review ID:** CD012203.pub2[17]<br><br>**Review Group:** Pregnancy & Childbirth<br><br>Date: 17 December 2018<br><br>**Sources of support:** National Institute for Health Research; University of Queensland; University of Adelaide; National Health and Medical Research Council, Australia.<br><br>**Matched NMA:** CD011689.pub3[18]	**Studies:** randomised controlled trials, quasi-randomised controlled trials, and cluster-randomised trials<br><br>**Patients:** women with a previous stillbirth of ≥ 20 weeks' gestation who are pregnant or considering a subsequent pregnancy<br><br>**Interventions analysed for primary outcome:** Low-molecular-weight heparin compared to no treatment/standard care; low-dose aspirin compared to placebo (4).<br><br>**Outcome:** Stillbirth (first primary).	**Outcome type and measure:** dichotomous; risk ratio.<br>Number of trials: 10<br>Number of patients: 222<br>**Type of analysis:** Frequentist.<br><br>**Heterogeneity assessed:** Yes (I square, chi-square, tausq).<br>**Heterogeneity found:** no.<br><br>**Grade classification:** Very low, low.<br><br>**ROB (SG/AC): low or unclear.**
**Review ID:** CD005656.pub3[19]	**Studies:** RCTs	**Outcome type and measure:** continuous; mean difference.<br>**Number of trials:** 10

Matched pair-wise meta-analyses	Eligibility criteria	Analysis
**Review Group:** Eyes & Vision<br><br>Date: 17 November 2020<br><br>**Sources of support:**<br>National Institutes of Health, USA; National Institute for Health Research (NIHR), UK.<br><br>**Matched NMA:** CD006768.pub3[20]	**Patients:** macular edema in diabetes<br><br>**Interventions analysed for primary outcome:** Intravitreal dexamethasone implant 0.7 mg compared to sham; Intravitreal dexamethasone implant 0.7 mg compared to intravitreal antiVEGF; Intravitreal fluocinolone implant 0.19 mg compared to sham; Intravitreal triamcinolone acetonide injection 4 mg compared to sham; ntravitreal triamcinolone acetonide injection 4 mg compared to macular laser; Intravitreal triamcinolone acetonide injection 4 mg compared to antiVEGF (6).<br><br>**Outcome:** visual acuity (first primary). Sub-grouped by time point.	**Number of patients:** 4348.<br>**Type of analysis:** Frequentist.<br><br>**Heterogeneity assessed:** Yes (I square, chi-square).<br>**Heterogeneity found:** no.<br><br>**Grade classification: very low, low,** moderate.<br><br>**ROB (SG/AC):** cannot be extracted for specific analysis but all trials are low or unclear.
**Review ID:** CD013040.pub2[21]<br><br>**Review Group:** Airways<br><br>Date: 29 January 2021<br><br>**Sources of support:** National Health and Medical Research Council, Australia; National Institute for Health.<br><br>**Matched NMA:** CD013198.pub2[22]	**Studies:** RCTs and controlled clinical trials (CCTs)<br><br>**Patients:** Chronic respiratory disease<br><br>**Interventions analysed for primary outcome:** telerehabilitation compared to centre-based (outpatient) pulmonary rehabilitation; telerehabilitation compared to no rehabilitation control (3).<br><br>**Outcome:** Exercise capacity (first primary).	**Outcome type and measure:** continuous; mean difference.<br>**Number of trials:** 15<br>**Number of patients:** 1904<br>**Type of analysis:** Frequentist.<br><br>**Heterogeneity assessed:** Yes (I square).<br>**Heterogeneity found:** no.<br><br>**Grade classification:** low, moderate.<br><br>**ROB (SG/AC):** some high risk in analysis
**Review ID:** CD013319.pub2[23]<br><br>**Review Group:** Heart<br><br>**Date:** 20 December 2019<br><br>**Sources of support:** National Institute for Health Research.	**Studies:** RCTs<br><br>**Patients:** adults with severe aortic stenosis who are at a low surgical risk.<br><br>**Interventions analysed for primary outcome:** Transcatheter aortic valve implantation compared to surgical aortic valve replacement (2).	**Outcome type and measure:** dichotomous; risk ratio.<br>**Number of trials:** 4<br>**Number of patients: 2**818.<br>**Type of analysis:** Frequentist.<br><br>**Heterogeneity assessed:** Yes (I square, chi-square, overlap of confidence intervals, tausq).<br>**Heterogeneity found:** no

Matched pair-wise meta-analyses	Eligibility criteria	Analysis
**Matched NMA:** CD013252.pub2[24]	**Outcome:** All-cause mortality (first primary).	**Grade classification:** moderate.<br><br>**ROB (SG/AC):** low or unclear.
**Review ID:** CD015017.pub3[25]<br><br>**Review Group:** Haematology<br><br>Date: 21 June 2022<br><br>**Sources of support:**<br>Federal Ministry of Education and Research; National Center for Complementary and Integrative Health; UK aid; University Hospital Wuerzburg; Liverpool School of Tropical Medicine; UK Foreign, Commonwealth, and Development Office.<br><br>**Matched NMA:** CD012633.pub2[26]	**Studies:** RCTs<br><br>**Patients:** people with moderate to severe disease (WHO scale 4–9); all trials contributing results to the summary of findings table investigated people with moderate disease (WHO scale 4 or 5) only<br><br>**Interventions analysed for primary outcome:** ivermectin plus standard of care versus standard of care plus/minus placebo (2).<br><br>**Outcome:** All-cause mortality at day 28 (first primary). Sub-grouped by mild and moderate disease.	**Outcome type and measure:** dichotomous; risk ratio.<br>**Number of trials:** 11<br>**Number of patients:** 3409<br>**Type of analysis:** Frequentist.<br><br>**Heterogeneity assessed:** Yes (I square, tausq).<br>**Heterogeneity found:** no.<br><br>**Grade classification:** Very low, moderate.<br><br>**ROB (SG/AC**): low or unclear.
**Review ID:** CD004834.pub3[27]<br><br>**Review Group:** Skin<br><br>**Date:** 27 August 2020<br><br>**Sources of support:** National Institute for Health Research.<br><br>**Matched NMA:** CD013206.pub2[28]	**Studies:** RCTs<br><br>**Patients:** people with American cutaneous and mucocutaneous leishmaniasis<br><br>**Interventions analysed for primary outcome:** Meglumine antimoniate (IMMA) (20 mg/kg/d for 20 days) compared to placebo (3 tablets/4 times a day for 28 d); Oral miltefosine (50 mg for 28 days) compared to placebo (same regimen); Oral miltefosine (1.2 to 3.3 mg/kg/d 28 days) compared to meglumine antimoniate (20 mg/kg 20 days); Azithromycin (500 mg 20 - 28 days) compared to meglumine antimoniate (15 - 20 mg/kg/d for 20 - 28 days); Topical imiquimod 5% (3 times/week) + meglumine antimoniate (IVMA) (20 mg/kg/d for 20 days) compared to placebo + IVMA; Thermotherapy one session (at 50 ºC for 30 seconds) compared to meglumine antimoniate (20 mg Sb5/kg/day for 20 days);	**Outcome type and measure:** dichotomous; risk ratio.<br>**Number of trials:** 75<br>**Number of patients:** 6533<br>**Type of analysis:** Frequentist.<br><br>**Heterogeneity assessed:** Yes (I square, chi-square).<br>**Heterogeneity found:** yes.<br><br>**Grade classification:** low, moderate, high.<br><br>**ROB (SG/AC):** cannot be extracted for specific analysis but all trials are low, high, unclear.

Matched pair-wise meta-analyses	Eligibility criteria	Analysis
	Pentoxifylline (oral 400 mg thrice daily) plus meglumine antimoniate (IMMA) (20 mg/ kg /day for 20 days) compared to IMMA plus placebo (12).<br><br>**Outcome:** Percentage of participants 'cured' at least three months after the end of treatment. (first primary).	
**Review ID:** CD006649.pub8 [29]<br><br>**Review Group:** Gynaecological, Neuro-oncology & Orphan Cancer<br><br>**Date:** 08 December 2021<br><br>**Sources of support:** National Institute for Health Research; American Society of Hematology, USA<br><br>**Matched NMA:** CD013700.pub2[30]	**Studies:** RCTs<br><br>**Patients:** people with cancer with venous thromboembolism<br><br>**Interventions analysed for primary outcome:** low molecular weight heparin compared to unfractionated heparin; Fondaparinux compared to heparin; Dalteparin compared to tinzaparin (6).<br><br>**Outcome:** All-cause mortality (first primary).	**Outcome type and measure:** dichotomous; risk ratio.<br>**Number of trials:** 15<br>**Number of patients:** 1615<br>**Type of analysis:** Frequentist.<br><br>**Heterogeneity assessed:** Yes (I square, chi-square, confidence interval overlap).<br>**Heterogeneity found:** no.<br><br>**Grade classification:** low.<br><br>**ROB (SG/AC):** cannot be extracted for specific analysis but all trials are low or high.
**Review ID:** CD011027.pub3[31]<br><br>**Review Group:** Neonatal<br><br>**Date:** 18 March 2022<br><br>**Sources of support:** Vermont Oxford Network, USA; National Institutes of Health, USA; University of Auckland; Waikato District Health Board; Auckland District Health Board; Aotearoa Foundation.<br><br>**Matched NMA:** CD013846.pub2[32]	**Studies:** RCTs<br><br>**Patients:** newborn infants with hypoglycaemia<br><br>**Interventions analysed for primary outcome:** Oral dextrose gel versus control (placebo gel or no gel) (2).<br><br>**Outcome:** Receipt of intravenous treatment for hypoglycaemia before discharge home (for each infant) (secondary, no meta-analysis for preceding outcomes).	**Outcome type and measure:** dichotomous; risk ratio.<br>**Number of trials:** 2<br>**Number of patients:** 312<br>**Type of analysis:** Frequentist.<br><br>**Heterogeneity assessed:** Yes (I square, chi-square).<br>**Heterogeneity found:** yes.<br><br>**Grade classification:** very low.<br><br>**ROB (SG/AC):** low or unclear.
**Review ID:** CD013343.pub2[33]<br><br>**Review Group:** Airways	**Studies:** RCTs	**Outcome type and measure:** dichotomous; odds ratio.<br>**Number of trials:** 36<br>**Number of patients:** 6192

Matched pair-wise meta-analyses	Eligibility criteria	Analysis
**Date:** 14 November 2022<br><br>**Sources of support:** Auckland, Waitemata and Counties Manukau District Health Boards; Bay of Plenty District Health Board; University of Auckland; National Institute for Health and Care Research.<br><br>**Matched NMA:** CD013799.pub2[34]	**Patients:** adults with chronic bronchitis or chronic obstructive pulmonary disease<br><br>**Interventions analysed for primary outcome: i**mmunostimulant vs. placebo (2).<br><br>**Outcome:** Number of participants with no exacerbations during the study period. (first primary).	**Type of analysis:** Frequentist.<br><br>**Heterogeneity assessed:** Yes (I square).<br>**Heterogeneity found:** yes.<br><br>**Grade classification:** moderate.<br><br>**ROB (SG/AC):** low and unclear.
**Review ID:** CD014915.pub [35]<br><br>**Review Group:** Pain, Palliative & Supportive Care<br><br>**Date:** 5 June 2023<br><br>**Sources of support:** National Institute for Health Research.<br><br>**Matched NMA:** CD014682.pub2[36]	**Studies:** RCTs<br><br>**Patients:** adults with cancer pain<br><br>**Interventions analysed for primary outcome: c**annabis-based medicines (oromucosal THC with or without CBD) compared with oromucosal placebo (2).<br><br>**Outcome:** Patient Global Impression of Change of much improved or very much improved (secondary, no data for preceding outcomes).	**Outcome type and measure:** dichotomous; risk difference.<br>**Number of trials:** 14<br>**Number of patients:** 1823<br>**Type of analysis:** Frequentist.<br><br>**Heterogeneity assessed:** Yes (I square).<br>**Heterogeneity found:** no.<br><br>**Grade classification:** moderate.<br><br>**ROB (SG/AC):** cannot be extracted for specific analysis but all trials are low or unclear.
**Review ID:** CD013410.pub2[37]<br><br>**Review Group:** Bone, Joint & Muscle Trauma<br><br>**Date:** 14 February 2022<br><br>**Sources of support:** National Institute for Health Research<br><br>**Matched NMA:** CD013404.pub2[38]	**Studies:** RCTs and quasi-RCTs.<br><br>**Patients:** adults with displaced and undisplaced hip fractures; included studies were for intracapsular fractures, except for one study of extracapsular fractures<br><br>**Interventions analysed for primary outcome:** Cemented versus uncemented hemiarthroplasty; Bipolar hemiarthroplasty compared with unipolar hemiarthroplasty; Total hip arthroplasty compared with hemiarthroplasty (6).<br><br>**Outcome:** Activities of daily living (first primary).	**Outcome type and measure:** continuous; mean difference; and dichotomous; risk ratio.<br>**Number of trials:** 58<br>**Number of patients:** 10,654<br>**Type of analysis:** Frequentist.<br><br>**Heterogeneity assessed:** Yes (I square, overlap of confidence intervals).<br>**Heterogeneity found:** yes.<br><br>**Grade classification:** Very low, moderate.

Matched pair-wise meta-analyses	Eligibility criteria	Analysis
		**ROB (SG/AC):** cannot be extracted for specific analysis but all trials are low, high, or unclear.
**Review ID:** CD011314.pub2[39]<br><br>**Review Group:** Hepato-Biliary<br><br>**Date: 07** March 2017<br><br>**Sources of support:** Danish State.<br><br>**Matched NMA:** CD011639.pub2[40]	**Studies:** RCTs<br><br>**Patients:** people with unresectable hepatocellular carcinoma<br><br>**Interventions analysed for primary outcome:** External beam radiotherapy (EBRT) plus transarterial chemoembolization (TACE) versus TACE alone; External beam radiotherapy (EBRT) versus transarterial chemoembolization (3).<br><br>**Outcome:** All-cause mortality (first primary).	**Outcome type and measure:** dichotomous; risk ratio.<br>**Number of trials:** 9<br>**Number of patients:** 879<br>**Type of analysis:** Frequentist.<br><br>**Heterogeneity assessed:** Yes (I square, chi-square).<br>**Heterogeneity found:** no.<br><br>**Grade classification:** Very low, low.<br><br>**ROB (SG/AC):** cannot be extracted for specific analysis but all trials are low or unclear.
**Review ID:** CD005004.pub3[41]<br><br>**Review Group:** Gynaecological, Neuro-oncology & Orphan Cancer<br><br>**Date:** 2 March 2020.<br><br>**Sources of support:** National Institute for Health Research Pilkington Family Trusts, UK; AG Biologische Krebstherapie, Deutsche Krebshilfe, Bonn, Germany; Nordic Cochrane Centre / ViFab, Denmark.<br><br>**Matched NMA:** CD013261.pub2[42]	**Studies:** RCTs and cohort and case-control observational studies<br><br>**Patients:** adults (aged at least 18 years)<br><br>**Interventions analysed for primary outcome:** Green tea extract supplementation compared with placebo; Highest compared with lowest green tea exposure (4).<br><br>**Outcome: t**he number of participants developing cancer (incidence) (first primary).	**Outcome type and measure:** dichotomous; risk ratio.<br>**Number of trials:** 142<br>**Number of patients:** over 1,101,795<br>**Type of analysis:** Frequentist.<br><br>**Heterogeneity assessed:** Yes (I square, chi-square).<br>**Heterogeneity found:** yes.<br><br>**Grade classification:** low.<br><br>**ROB (SG/AC):** cannot be extracted.
**Review ID:** CD011979.pub2[43]<br><br>**Review Group:** Wounds<br><br>**Date:** 28 June 2017.	**Studies:** RCTs or cluster RCTs<br><br>**Patients:** Diabetes with foot ulcers.<br><br>**Interventions analysed for primary outcome:** Phototherapy compared with placebo/no phototherapy (2).	**Outcome type and measure:** dichotomous; risk ratio.<br>**Number of trials:** 8<br>**Number of patients:** 316<br>**Type of analysis:** Frequentist.<br><br>**Heterogeneity assessed:** Yes (I square).<br>**Heterogeneity found:** no.

Matched pair-wise meta-analyses	Eligibility criteria	Analysis
**Sources of support:** National Institute for Health Research; Natural Science Funds of Shaanxi Province China (2014JM4180), China.<br><br>**Matched NMA:** CD011947.pub2[44]	**Outcome:** Complete wound healing (first primary).	**Grade classification:** low.<br><br>**ROB (SG/AC):** cannot be extracted for specific analysis but all trials are low or unclear.
**Review ID:** CD012522.pub2[45]<br><br>**Review Group:** Wounds<br><br>**Date:** 03 July 2018<br><br>**Sources of support:** University of Manchester; National Institute for Health Research.<br><br>**Matched NMA:** CD012583.pub2[46]	**Studies:** RCTs or cluster RCTs<br><br>**Patients:** open fracture wounds, other open traumatic wounds<br><br>**Interventions analysed for primary outcome:**<br>NPWT 125 mmHg compared with standard care;<br>NPWT 75 mmHg compared with standard care;<br>NPWT 125 mmHg compared with NPWT 75 mmHg (3).<br><br>**Outcome:** wound infection (second primary, no meta-analysis for preceding outcome). Sub-grouped by wound type.	**Outcome type and measure:** dichotomous; risk ratio.<br>**Number of trials:** 7<br>**Number of patients:** 1377<br>**Type of analysis:** Frequentist.<br><br>**Heterogeneity assessed:** Yes (I square, chi-square).<br>**Heterogeneity found:** yes<br><br>**Grade classification:** Very low, low<br><br>**ROB (SG/AC):** cannot be extracted for specific analysis but all trials are low or unclear.
**Review ID:** CD009910.pub2[47]<br><br>**Review Group:** Airways<br><br>**Date:** 5 March 2014<br><br>**Sources of support:** Iberoamerican Cochrane Centre, Spain; Programa Enlaza-Mundos. Alcaldía de Medellín 2011, Colombia.<br><br>**Matched NMA:** CD010844.pub2[48]	**Studies:** RCTs<br><br>**Patients:** adult patients with asthma<br><br>**Interventions analysed for primary outcome:** Bronchial thermoplasty compared with any active control (medical management or sham intervention) (2).<br><br>**Outcome:** Health-related quality of life (first primary).	**Outcome type and measure:** continuous; mean difference.<br>**Number of trials:** 3<br>**Number of patients:** 429<br>**Type of analysis:** Frequentist.<br><br>**Heterogeneity assessed:** Yes (I square).<br>**Heterogeneity found:** no.<br><br>**Grade classification:** moderate.<br><br>**ROB (SG/AC):** cannot be extracted for specific analysis but all trials are low or unclear.

Abbreviations: AC: allocation concealment; NMA: network meta-analysis; RCT: randomised controlled trial; SG: sequence generation.

1. Jones E, Stewart F, Taylor B, Davis PG, Brown SJ. Early postnatal discharge from hospital for healthy mothers and term infants. *Cochrane Database Syst Rev* 2021(6) doi: 10.1002/14651858.CD002958.pub2
2. Ghosh J, Papadopoulou A, Devall AJ, et al. Methods for managing miscarriage: a network meta-analysis. *Cochrane Database Syst Rev* 2021(6) doi: 10.1002/14651858.CD012602.pub2
3. Cook LA, Pun A, Gallo MF, Lopez LM, Van Vliet H. Scalpel versus no-scalpel incision for vasectomy. *Cochrane Database Syst Rev* 2014(3) doi: 10.1002/14651858.CD004112.pub4
4. de Bastos M, Stegeman BH, Rosendaal FR, et al. Combined oral contraceptives: venous thrombosis. *Cochrane Database Syst Rev* 2014(3) doi: 10.1002/14651858.CD010813.pub2
5. Williams MJ, Ramson JA, Brownfoot FC. Different corticosteroids and regimens for accelerating fetal lung maturation for babies at risk of preterm birth. *Cochrane Database Syst Rev* 2022(8) doi: 10.1002/14651858.CD006764.pub4
6. Wilson A, Hodgetts-Morton VA, Marson EJ, et al. Tocolytics for delaying preterm birth: a network meta-analysis (0924). *Cochrane Database Syst Rev* 2022(8) doi: 10.1002/14651858.CD014978.pub2
7. Cates CJ, Schmidt S, Ferrer M, Sayer B, Waterson S. Inhaled steroids with and without regular salmeterol for asthma: serious adverse events. *Cochrane Database Syst Rev* 2018(12) doi: 10.1002/14651858.CD006922.pub4
8. Oba Y, Keeney E, Ghatehorde N, Dias S. Dual combination therapy versus long-acting bronchodilators alone for chronic obstructive pulmonary disease (COPD): a systematic review and network meta-analysis. *Cochrane Database Syst Rev* 2018(12) doi: 10.1002/14651858.CD012620.pub2
9. Davidson SJ, Barrett HL, Price SA, Callaway LK, Dekker Nitert M. Probiotics for preventing gestational diabetes. *Cochrane Database Syst Rev* 2021(4) doi: 10.1002/14651858.CD009951.pub3
10. Devall AJ, Papadopoulou A, Podesek M, et al. Progestogens for preventing miscarriage: a network meta-analysis. *Cochrane Database Syst Rev* 2021(4) doi: 10.1002/14651858.CD013792.pub2
11. Roberts NP, Roberts PA, Jones N, Bisson JI. Psychological therapies for post-traumatic stress disorder and comorbid substance use disorder. *Cochrane Database Syst Rev* 2016(4) doi: 10.1002/14651858.CD010204.pub2
12. Pompoli A, Furukawa TA, Imai H, et al. Psychological therapies for panic disorder with or without agoraphobia in adults: a network meta-analysis. *Cochrane Database Syst Rev* 2016(4) doi: 10.1002/14651858.CD011004.pub2
13. Schenkel AB, Veitz-Keenan A. Dental cavity liners for Class I and Class II resin-based composite restorations. *Cochrane Database Syst Rev* 2019(3) doi: 10.1002/14651858.CD010526.pub3
14. Walsh T, Worthington HV, Glenny AM, Marinho VCC, Jeroncic A. Fluoride toothpastes of different concentrations for preventing dental caries. *Cochrane Database Syst Rev* 2019(3) doi: 10.1002/14651858.CD007868.pub3
15. Simon N, Robertson L, Lewis C, et al. Internet-based cognitive and behavioural therapies for post-traumatic stress disorder (PTSD) in adults. *Cochrane Database Syst Rev* 2021(5) doi: 10.1002/14651858.CD011710.pub3
16. Hetrick SE, McKenzie JE, Bailey AP, et al. New generation antidepressants for depression in children and adolescents: a network meta-analysis. *Cochrane Database Syst Rev* 2021(5) doi: 10.1002/14651858.CD013674.pub2

17. Wojcieszek AM, Shepherd E, Middleton P, et al. Care prior to and during subsequent pregnancies following stillbirth for improving outcomes. *Cochrane Database Syst Rev* 2018(12) doi: 10.1002/14651858.CD012203.pub2
18. Gallos ID, Papadopoulou A, Man R, et al. Uterotonic agents for preventing postpartum haemorrhage: a network meta-analysis. *Cochrane Database Syst Rev* 2018(12) doi: 10.1002/14651858.CD011689.pub3
19. Rittiphairoj T, Mir TA, Li T, Virgili G. Intravitreal steroids for macular edema in diabetes. *Cochrane Database Syst Rev* 2020(11) doi: 10.1002/14651858.CD005656.pub3
20. Scheiman M, Kulp MT, Cotter SA, et al. Interventions for convergence insufficiency: a network meta-analysis. *Cochrane Database Syst Rev* 2020(12) doi: 10.1002/14651858.CD006768.pub3
21. Cox NS, Dal Corso S, Hansen H, et al. Telerehabilitation for chronic respiratory disease. *Cochrane Database Syst Rev* 2021(1) doi: 10.1002/14651858.CD013040.pub2
22. Janjua S, Mathioudakis AG, Fortescue R, et al. Prophylactic antibiotics for adults with chronic obstructive pulmonary disease: a network meta-analysis. *Cochrane Database Syst Rev* 2021(1) doi: 10.1002/14651858.CD013198.pub2
23. Kolkailah AA, Doukky R, Pelletier MP, et al. Transcatheter aortic valve implantation versus surgical aortic valve replacement for severe aortic stenosis in people with low surgical risk. *Cochrane Database Syst Rev* 2019(12) doi: 10.1002/14651858.CD013319.pub2
24. Al Said S, Alabed S, Kaier K, et al. Non-vitamin K antagonist oral anticoagulants (NOACs) post-percutaneous coronary intervention: a network meta-analysis. *Cochrane Database Syst Rev* 2019(12) doi: 10.1002/14651858.CD013252.pub2
25. Popp M, Reis S, Schießer S, et al. Ivermectin for preventing and treating COVID-19. *Cochrane Database Syst Rev* 2022(6) doi: 10.1002/14651858.CD015017.pub3
26. Adams A, Scheckel B, Habsaoui A, et al. Intravenous iron versus oral iron versus no iron with or without erythropoiesis- stimulating agents (ESA) for cancer patients with anaemia: a systematic review and network meta-analysis. *Cochrane Database Syst Rev* 2022(6) doi: 10.1002/14651858.CD012633.pub2
27. Pinart M, Rueda JR, Romero GAS, et al. Interventions for American cutaneous and mucocutaneous leishmaniasis. *Cochrane Database Syst Rev* 2020(8) doi: 10.1002/14651858.CD004834.pub3
28. Sawangjit R, Dilokthornsakul P, Lloyd-Lavery A, et al. Systemic treatments for eczema: a network meta-analysis. *Cochrane Database Syst Rev* 2020(9) doi: 10.1002/14651858.CD013206.pub2
29. Kahale LA, Matar CF, Hakoum MB, et al. Anticoagulation for the initial treatment of venous thromboembolism in people with cancer. *Cochrane Database Syst Rev* 2021(12) doi: 10.1002/14651858.CD006649.pub8
30. Walter MA, Nesti C, Spanjol M, et al. Treatment for gastrointestinal and pancreatic neuroendocrine tumours: a network meta-analysis. *Cochrane Database Syst Rev* 2021(11) doi: 10.1002/14651858.CD013700.pub2
31. Edwards T, Liu G, Battin M, et al. Oral dextrose gel for the treatment of hypoglycaemia in newborn infants. *Cochrane Database Syst Rev* 2022(3) doi: 10.1002/14651858.CD011027.pub3
32. Mitra S, Gardner CE, MacLellan A, et al. Prophylactic cyclo-oxygenase inhibitor drugs for the prevention of morbidity and mortality in preterm infants: a network meta-analysis. *Cochrane Database Syst Rev* 2022(4) doi: 10.1002/14651858.CD013846.pub2

33. Fraser A, Poole P. Immunostimulants versus placebo for preventing exacerbations in adults with chronic bronchitis or chronic obstructive pulmonary disease. *Cochrane Database Syst Rev* 2022(11) doi: 10.1002/14651858.CD013343.pub2
34. Oba Y, Anwer S, Maduke T, Patel T, Dias S. Effectiveness and tolerability of dual and triple combination inhaler therapies compared with each other and varying doses of inhaled corticosteroids in adolescents and adults with asthma: a systematic review and network meta-analysis. *Cochrane Database Syst Rev* 2022(12) doi: 10.1002/14651858.CD013799.pub2
35. Häuser W, Welsch P, Radbruch L, et al. Cannabis-based medicines and medical cannabis for adults with cancer pain. *Cochrane Database Syst Rev* 2023(6) doi: 10.1002/14651858.CD014915.pub2
36. Birkinshaw H, Friedrich CM, Cole P, et al. Antidepressants for pain management in adults with chronic pain: a network meta-analysis. *Cochrane Database Syst Rev* 2023(5) doi: 10.1002/14651858.CD014682.pub2
37. Lewis SR, Macey R, Parker MJ, Cook JA, Griffin XL. Arthroplasties for hip fracture in adults. *Cochrane Database Syst Rev* 2022(2) doi: 10.1002/14651858.CD013410.pub2
38. Lewis SR, Macey R, Stokes J, et al. Surgical interventions for treating intracapsular hip fractures in older adults: a network meta-analysis. *Cochrane Database Syst Rev* 2022(2) doi: 10.1002/14651858.CD013404.pub2
39. Abdel-Rahman O, Elsayed Z. External beam radiotherapy for unresectable hepatocellular carcinoma. *Cochrane Database Syst Rev* 2017(3) doi: 10.1002/14651858.CD011314.pub2
40. Rodríguez-Perálvarez M, Guerrero-Misas M, Thorburn D, et al. Maintenance immunosuppression for adults undergoing liver transplantation: a network meta-analysis. *Cochrane Database Syst Rev* 2017(3) doi: 10.1002/14651858.CD011639.pub2
41. Filippini T, Malavolti M, Borrelli F, et al. Green tea (Camellia sinensis) for the prevention of cancer. *Cochrane Database Syst Rev* 2020(3) doi: 10.1002/14651858.CD005004.pub3
42. Hanna C, Lawrie TA, Rogozińska E, et al. Treatment of newly diagnosed glioblastoma in the elderly: a network meta-analysis. *Cochrane Database Syst Rev* 2020(3) doi: 10.1002/14651858.CD013261.pub2
43. Wang HT, Yuan JQ, Zhang B, et al. Phototherapy for treating foot ulcers in people with diabetes. *Cochrane Database Syst Rev* 2017(6) doi: 10.1002/14651858.CD011979.pub2
44. Westby MJ, Dumville JC, Soares MO, Stubbs N, Norman G. Dressings and topical agents for treating pressure ulcers. *Cochrane Database Syst Rev* 2017(6) doi: 10.1002/14651858.CD011947.pub2
45. Iheozor-Ejiofor Z, Newton K, Dumville JC, et al. Negative pressure wound therapy for open traumatic wounds. *Cochrane Database Syst Rev* 2018(7) doi: 10.1002/14651858.CD012522.pub2
46. Norman G, Westby MJ, Rithalia AD, et al. Dressings and topical agents for treating venous leg ulcers. *Cochrane Database Syst Rev* 2018(6) doi: 10.1002/14651858.CD012583.pub2
47. Torrego A, Solà I, Munoz AM, et al. Bronchial thermoplasty for moderate or severe persistent asthma in adults. *Cochrane Database Syst Rev* 2014(3) doi: 10.1002/14651858.CD009910.pub2
48. Kew KM, Dias S, Cates CJ. Long-acting inhaled therapy (beta-agonists, anticholinergics and steroids) for COPD: a network meta-analysis. *Cochrane Database Syst Rev* 2014(3) doi: 10.1002/14651858.CD010844.pub2
