# Supplementary material for: The impact of Cochrane Reviews that apply network meta-analysis in clinical guidelines: A systematic review
Source: PLoS One. 2024 Dec 26;19(12):e0315563. doi: 10.1371/journal.pone.0315563 (PMC11671017; doi:10.1371/journal.pone.0315563)
Supplement: S6 Table — (PDF) [file pone.0315563.s012.pdf]

Table S6: Data extracted for guidelines that cited pairwise meta-analysis reviews

Review	Review year	Number of citations	Guideline location	Guideline year	Different version of the same guideline cited for a review	Guideline titles	Guideline author	Use of review in guideline	Guideline citation
CD002958.pub2[1]	2021	2	India	2023	No	Care of the normal newborn (v1.1)	National Neonatology Forum of India	Giving information leading to a recommendation	Care of the normal newborn. 25 November 2023. New Delhi: National Neonatology Forum of India; 2023 (NNF/ 2023/CPG/ 2023.2.0). License: CC BY-NC-SA 4.0
CD002958.pub2[1]			Global	2022	No	WHO recommendations on maternal and newborn care for a positive postnatal experience	World Health Organization	Referenced alongside a recommendation.	WHO recommendations on maternal and newborn care for a positive postnatal experience. Geneva: World Health Organization; 2022. Licence: CC BY-NC-SA 3.0 IGO.
CD004112.pub4[2]	2014	6	Germany, Austria, Switzerland	2024	No	[S2k-Guideline: Non-hormonal contraception]	German Society of Gynecology and Obstetrics, Austrian Society of Gynecology and Obstetrics, Swiss Society	Giving information	Non-hormonal contraception. Guideline of the German Society of Gynecology and Obstetrics (Sk2-Level, AWMF Registry No. 015/095, December 2023). Available from: https://register.awmf.org/assets/guidelines/015-095l_S2k_Nicht-hormonelle-

Review	Review year	Number of citations	Guideline location	Guideline year	Different version of the same guideline cited for a review	Guideline titles	Guideline author	Use of review in guideline	Guideline citation
							of Gynecology and Obstetrics		Empfaengnisverhuetung_2024-01.pdf
CD004112.pub4[2]			Canada	2015	No	**Canadian contraception consensus: part 2 of 4**	The Society of Obstetricians and Gynaecologists of Canada	Cannot access full version	Black A, Guilbert E; Co-Authors; Costescu D, Dunn S, Fisher W, Kives S, Mirosh M, Norman WV, Pymar H, Reid R, Roy G, Varto H, Waddington A, Wagner MS, Whelan AM; Special Contributors; Ferguson C, Fortin C, Kielly M, Mansouri S, Todd N. Canadian Contraception Consensus (Part 2 of 4). J Obstet Gynaecol Can. 2015 Nov;37(11):1033-9. English, French. doi: 10.1016/s1701-2163(16)30054-8. PMID: 26629725.
CD004112.pub4[2]			Europe	2016	Yes	**Guidelines on male infertility**	European Association of Urology	Giving information	EAU Guidelines on male infertility European Association of Urology. 2016. Available from: https://d56bochluxqnz.cloudfront.net/media/EAU-Guidelines-Male-Infertility-2016-2.pdf

Review	Review year	Number of citations	Guideline location	Guideline year	Different version of the same guideline cited for a review	Guideline titles	Guideline author	Use of review in guideline	Guideline citation
CD004112.pub4[2]			Europe	2017	Yes	Guidelines on male infertility	European Association of Urology	Giving information	EAU Guidelines on male infertility European Association of Urology. 2017. Available from: https://d56bochluxqnz.cloudfront.net/media/17-Male-Infertility_2017_web.pdf
CD004112.pub4[2]			Europe	2018	Yes	Guidelines on male infertility	European Association of Urology	Giving information	EAU Guidelines on male infertility European Association of Urology. 2018. Available from https://d56bochluxqnz.cloudfront.net/media/EAU-Guidelines-on-Male-Infertility-2018-large-text.pdf
CD004112.pub4[2]			Europe	2019	Yes	Guidelines on Male Infertility	European Association of Urology	Giving information	EAU Guidelines on male infertility European Association of Urology. 2019. Available from https://d56bochluxqnz.cloudfront.net/media/EAU-Guidelines-on-Male-Infertility-2019.pdf
CD004834.pub3[3]	2020	1	Global	2022	No	Guideline for the Treatment of Leishmaniasis in the Americas	World Health Organization	Giving information leading to recommendations	Pan American Health Organization. Guideline for the treatment of leishmaniasis in the Americas. Second

Review	Review year	Number of citations	Guideline location	Guideline year	Different version of the same guideline cited for a review	Guideline titles	Guideline author	Use of review in guideline	Guideline citation
						(Second Edition)			edition. Washington, DC: PAHO; 2022. Available from: https://doi.org/10.37774/9789275125038.
CD005004.pub3[4]	2020	3	Germany	2023	No	[S3-guideline for diagnosis and treatment of biliary carcinomas - long version]	German Society for Gastroenterology, Digestive and Metabolic Diseases	Not cited in text but in reference list.	Oncology Guideline Program (German Cancer Society, German Cancer Aid, AWMF): Diagnosis and Therapy of Hepatocellular Carcinoma and Biliary Carcinomas Long Version 3.0, 2022, AWMF registration number: 032/053OL, https://www.leitlinienprogramm-onkologie.de/leitlinien/hcc-und-biliaere-karzinome/
CD005004.pub3[4]			Germany	2021	Yes	[S3-Guideline: Hepatocellular carcinoma and biliary carcinomas]	Oncology Guideline Program (German Cancer Society; German Cancer Aid; The	Giving information	Oncology Guideline Program (German Cancer Society, German Cancer Aid, AWMF): Diagnosis and Therapy of Hepatocellular Carcinoma and Biliary Carcinoma Carciomas, long version 2.0, 2021,

Review	Review year	Number of citations	Guideline location	Guideline year	Different version of the same guideline cited for a review	Guideline titles	Guideline author	Use of review in guideline	Guideline citation
							Association of Scientific Medical Societies)		AWMF registration number: 032/053OL, https://www.leitlinienprogramm-onkologie.de/leitlinien/hcc-und-billiäre-karzinome
CD005004.pub3[4]			Germany	2023	Yes	[S3-Guideline: Hepatocellular carcinoma and biliary carcinomas]	Oncology Guideline Program (German Cancer Society; German Cancer Aid; The Association of Scientific Medical Societies)	Giving information	Oncology Guideline Program (German Cancer Society, German Cancer Aid, AWMF): Diagnosis and Therapy of Hepatocellular Carcinoma and Biliary Carcinoma, Long Version 4.0, 2023, AWMF Registration Number: 032-053OL https://www.leitlinienprogramm-onkologie.de/leitlinien/hcc-und-biliaere-karzinome/

Review	Review year	Number of citations	Guideline location	Guideline year	Different version of the same guideline cited for a review	Guideline titles	Guideline author	Use of review in guideline	Guideline citation
CD005656.pub3[5]	2020	1	UK	2022	No	**Technology appraisal guidance: Faricimab for treating diabetic macular oedema**	National Institute for Health and Care Excellence	Used to make a decision on a NICE submission	National Institute for Health and Care Excellence (commissioner). Faricimab for treating diabetic macular oedema NICE guideline TA799. London: National Clinical Guideline Centre, Royal College of Physicians; 2022. Available from: https://www.nice.org.uk/guidance/ta799/evidence
CD006649.pub8[6]	2021	1	Global	2022	No	**2022 international clinical practice guidelines for the treatment and prophylaxis of venous thromboembolism in patients with cancer, including patients with COVID-19**	International Initiative on Thrombosis and Cancer (ITAC) advisory panel	Giving information	Farge D, Frere C, Connors JM, Khorana AA, Kakkar A, Ay C, Muñoz A, Brenner B, Prata PH, Brilhante D, Antic D, Casais P, Guillermo Esposito MC, Ikezoe T, Abutalib SA, Meillon-García LA, Bounameaux H, Pabinger I, Douketis J; International Initiative on Thrombosis and Cancer (ITAC) advisory panel. 2022 international

Review	Review year	Number of citations	Guideline location	Guideline year	Different version of the same guideline cited for a review	Guideline titles	Guideline author	Use of review in guideline	Guideline citation
									clinical practice guidelines for the treatment and prophylaxis of venous thromboembolism in patients with cancer, including patients with COVID-19. Lancet Oncol. 2022 Jul;23(7):e334-e347. doi: 10.1016/S1470-2045(22)00160-7. PMID: 35772465; PMCID: PMC9236567.
CD006764.pub4[7]	2022	1	Global	2022	No	WHO recommendations on antenatal corticosteroids for improving preterm birth outcomes	World Health Organization	Not cited in text but in reference list.	WHO recommendations on antenatal corticosteroids for improving preterm birth outcomes. Geneva: World Health Organization; 2022. Licence: CC BY-NC-SA 3.0 IGO.
CD006922.pub4[8]	2018	7	Netherlands	2022	No	[Dutch College of General Practitioners – Guideline on asthma in children (version 5.0 –	Dutch College of General Practitioners	Giving information	Dutch College of General Practitioners. [Dutch College of General Practitioners – Guideline on asthma in children (version 5.0 – Guideline M24)]. 2022. Available

Review	Review year	Number of citations	Guideline location	Guideline year	Different version of the same guideline cited for a review	Guideline titles	Guideline author	Use of review in guideline	Guideline citation
						Guideline M24)]			from: https://richtlijnen.nhg.org/files/pdf/71_Astma%20bij%20kinderen_mei-2022.pdf
CD006922.pub4[8]			Global	2020	Yes	Global Strategy for Asthma Management and Prevention (Updated 2020)	Global Initiative for Asthma	Giving information leading to recommendations	Global Initiative for Asthma. Global Strategy for Asthma Management and Prevention. 2020. Available from: https://ginasthma.org/wp-content/uploads/2020/04/GINA-2020-full-report_-final-_wms.pdf
CD006922.pub4[8]			Global	2021	Yes	Global Strategy for Asthma Management and Prevention (Updated 2021)	Global Initiative for Asthma	Giving information leading to recommendations	Global Initiative for Asthma. Global Strategy for Asthma Management and Prevention. 2021. Available from: http://ginasthma.org/wp-content/uploads/2021/05/GINA-Main-Report-2021-V2-WMS.pdf
CD006922.pub4[8]			Global	2022	Yes	Global Strategy for Asthma Management and Prevention (Updated 2022)	Global Initiative for Asthma	Giving information leading to recommendations	Global Initiative for Asthma. Global Strategy for Asthma Management and Prevention. 2022. Available from:

Review	Review year	Number of citations	Guideline location	Guideline year	Different version of the same guideline cited for a review	Guideline titles	Guideline author	Use of review in guideline	Guideline citation
									http://ginasthma.org/wp-content/uploads/2022/07/GINA-Main-Report-2022-FINAL-22-07-01-WMS.pdf
CD006922.pub4[8]			Global	2023	Yes	Global Strategy for Asthma Management and Prevention (Updated 2023)	Global Initiative for Asthma	Giving information leading to recommendations	Global Initiative for Asthma. Global Strategy for Asthma Management and Prevention. 2023. Available from: http;/ginasthma.org/wp-content/uploads/2023/07/GINA-2023-Full-report-23_07_06-WMS.pdf
CD006922.pub4[8]			Saudi Arabia	2021	Yes	The Saudi Initiative for Asthma - 2021 Update: Guidelines for the diagnosis and management of asthma in adults and children	Saudi Initiative for Asthma Group, Saudi Thoracic Society	Giving information leading to a recommendation	Al-Moamary MS, Alhaider SA, Alangari AA, Idrees MM, Zeitouni MO, Al Ghobain MO, Alanazi AF, Al-Harbi AS, Yousef AA, Alorainy HS, Al-Hajjaj MS. The Saudi Initiative for Asthma - 2021 Update: Guidelines for the diagnosis and management of asthma in adults and children. Ann Thorac Med. 2021 Jan-Mar;16(1):4-56. doi:

Review	Review year	Number of citations	Guideline location	Guideline year	Different version of the same guideline cited for a review	Guideline titles	Guideline author	Use of review in guideline	Guideline citation
									10.4103/atm.ATM_697_20. Epub 2021 Jan 14. PMID: 33680125; PMCID: PMC7908897.
CD006922.pub4[8]			Saudi Arabia	2024	Yes	The Saudi Initiative for Asthma - 2024 Update: Guidelines for the diagnosis and management of asthma in adults and children	Saudi Initiative for Asthma Group, Saudi Thoracic Society	Referenced alongside a recommendation	Al-Moamary, Mohamed Saad; Alhaider, Sami A.1; Allehebi, Riyad2; Idrees, Majdy M.3; Zeitouni, Mohammed O.4; Al Ghobain, Mohammed O.; Alanazi, Abdullah F.; Al-Harbi, Adel S.5; Yousef, Abdullah A.6; Alorainy, Hassan S.7; Al-Hajjaj, Mohamed S.8. The Saudi initiative for asthma – 2024 update: Guidelines for the diagnosis and management of asthma in adults and children. Annals of Thoracic Medicine 19(1):p 1-55, Jan–Mar 2024. | DOI: 10.4103/atm.atm_248_23
CD009910.pub2[9]	2014	13	Finland	2022	No	[Duodecim Current Care Guidelines: Asthma]	Finnish Medical Society Duodecim, the	Giving information	Asthma. Current Care Recommendation. Finnish Medical Society Duodecim, Finnish

Review	Review year	Number of citations	Guideline location	Guideline year	Different version of the same guideline cited for a review	Guideline titles	Guideline author	Use of review in guideline	Guideline citation
							Finnish Association of Pulmonologists, the Finnish Paediatric Association, the Allergology Association of Finnish Paediatricians, the Finnish Society of Clinical Physiology,		Association of Pulmonologists, Finnish Paediatric Association A working group appointed by the Finnish Association of Clinical Physiology. Helsinki: Finnish Finnish Medical Society Duodecim, 2022 Available online: www.kaypahoito.fi
CD009910.pub2[9]			Spain	2015	Yes	[GEMA(4.0). Guidelines for Asthma Management]	Spanish Guide to the Management of Asthma Executive Committee	Giving information	DOI: 10.1016/S0300-2896(15)32812-X
CD009910.pub2[9]			Spain	2020	Yes	[GEMA5.0 Guidelines for Asthma Management]	Spanish Association of Primary Care Pediatrics, Latin American Thoracic Association, Primary Care	Giving information	GUÍA ESPAÑOLA PARA EL MANEJO DEL ASMA. [GEMA5.0 Guidelines for Asthma Management]. 2020. Available from: https://www.semg.es/images/documentos/GEMA_5.0.pdf

Review	Review year	Number of citations	Guideline location	Guideline year	Different version of the same guideline cited for a review	Guideline titles	Guideline author	Use of review in guideline	Guideline citation
							Respiratory Society, Spanish Society of Allergology and Clinical Immunology, Spanish Society of Family and Community Pharmacy, Spanish Society of Clinical Pharmacology, Spanish Society of Hospital Pharmacy, Spanish Society of Clinical Immunology, Allergology and Paediatric Asthma, Spanish Society of Physicians of		

Review	Review year	Number of citations	Guideline location	Guideline year	Different version of the same guideline cited for a review	Guideline titles	Guideline author	Use of review in guideline	Guideline citation
							Primary Care, Spanish Society of Emergency Medicine, Spanish Society of Family and Community Medicine, Spanish Society of General and Family Physicians, Spanish Society of Paediatric Pneumology, Spanish Society of Otolaryngology and Head and Neck Surgery, Spanish Society of Pneumology and Thoracic		

Review	Review year	Number of citations	Guideline location	Guideline year	Different version of the same guideline cited for a review	Guideline titles	Guideline author	Use of review in guideline	Guideline citation
							Surgery, Spanish Society of Paediatrics Outpatient and Primary Care, Portuguese Society of Pneumology		
CD009910.pub2[9]			Spain	2022	Yes	[GEMA5.2 Guidelines for Asthma Management].	Spanish Association of Primary Care Paediatrics, Latin American Thoracic Association, Primary Care Respiratory Society, Spanish Society of Allergology and Clinical Immunology, Spanish Society of Family and Community	Giving information	GUÍA ESPAÑOLA PARA EL MANEJO DEL ASMA. [GEMA5.2 Guidelines for Asthma Management]. 2022. Available from: https://gemasma.com/sites/default/files/2022-11/GEMA_52020922.pdf

Review	Review year	Number of citations	Guideline location	Guideline year	Different version of the same guideline cited for a review	Guideline titles	Guideline author	Use of review in guideline	Guideline citation
							Pharmacy, Spanish Society of Clinical Pharmacology, Spanish Society of Hospital Pharmacy, Spanish Society of Clinical Immunology, Paediatric Allergology and Asthma, Spanish Society of Primary Care Physicians, Spanish Society of Emergency Medicine, Spanish Society of Family and Community Medicine, Spanish		

Review	Review year	Number of citations	Guideline location	Guideline year	Different version of the same guideline cited for a review	Guideline titles	Guideline author	Use of review in guideline	Guideline citation
							Society of General and Family Physicians, Spanish Society of Paediatric Pneumology, Spanish Society of Otolaryngology and Head and Neck Surgery, Spanish Society of Pneumology and Thoracic Surgery, Spanish Society of Out-of-Hospital Paediatrics and Primary Care, Portuguese Society of Pneumology		

Review	Review year	Number of citations	Guideline location	Guideline year	Different version of the same guideline cited for a review	Guideline titles	Guideline author	Use of review in guideline	Guideline citation
CD009910.pub2[9]			Spain	July 2023	Yes	**[GEMA5.3 Guidelines for Asthma Management]**	Spanish Association of Primary Care Paediatrics, Latin American Thoracic Association, Primary Care Respiratory Society, Spanish Society of Allergology and Clinical Immunology, Spanish Society of Family and Community Pharmacy, Spanish Society of Clinical Pharmacology, Spanish Society of Hospital Pharmacy, Spanish	Giving information	GUÍA ESPAÑOLA PARA EL MANEJO DEL ASMA. [GEMA5.3 Guidelines for Asthma Management]. 2023. Available from: https://www.gemasma.com/sites/default/files/2023-07/GEMA_53_26072023.pdf

Review	Review year	Number of citations	Guideline location	Guideline year	Different version of the same guideline cited for a review	Guideline titles	Guideline author	Use of review in guideline	Guideline citation
							Society of Clinical Immunology, Allergology and Paediatric Asthma, Spanish Society of Primary Care Physicians, Spanish Society of Emergency Medicine, Spanish Society of Family and Community Medicine, Spanish Society of General and Family Physicians, Spanish Society of Paediatric Pneumology, Spanish Society of		

Review	Review year	Number of citations	Guideline location	Guideline year	Different version of the same guideline cited for a review	Guideline titles	Guideline author	Use of review in guideline	Guideline citation
							Otolaryngology and Head and Neck Surgery, Spanish Society of Pneumology and Thoracic Surgery, Spanish Society of Outpatient Paediatrics and Primary Care, Portuguese Society of Pneumology		
CD009910.pub2[9]			Netherlands	2020	No	**[Guideline: Diagnosis and treatment of severe asthma]**	Dutch Association of Physicians for Lung Diseases and Tuberculosis.	Providing information	Dutch Association of Physicians for Lung Diseases and Tuberculosis. [Guideline: Diagnosis and treatment of severe asthma]. 2020. Available from: https://richtlijnendatabase.nl/richtlijn/diagnostiek_en_behandeling_van_ernstig_astma/startpagina_-_ernstig_astma.html

Review	Review year	Number of citations	Guideline location	Guideline year	Different version of the same guideline cited for a review	Guideline titles	Guideline author	Use of review in guideline	Guideline citation
CD009910.pub2[9]			Spain	2015	No	[Guidelines for severe uncontrolled asthma]	Cisneros Serrano, Melero Moreno, Almonacid Sánchez, Perpiñá Tordera, Picado Valles, Martínez Moragón, Pérez de Llano, Soto Campos, Urrutia Landa, García Hernández	Referenced alongside a recommendation.	DOI: 10.1016/j.arbr.2015.03.010
CD009910.pub2[9]			Germany	2020	No	[National Care Guideline: Asthma, Edition 4 2020, Version 1]	Association of German Medical Associations, National Association of Statutory Health Insurance Physicians, Association of Scientific	Giving information	Bundesärztekammer (BÄK), Kassenärztliche Bundesvereinigung (KBV), Arbeitsgemeinschaft der Wissenschaftlichen Medizinischen Fachgesellschaften (AWMF). Nationale VersorgungsLeitlinie Asthma – Langfassung, 4. Auflage. Version 1. 2020 DOI:

Review	Review year	Number of citations	Guideline location	Guideline year	Different version of the same guideline cited for a review	Guideline titles	Guideline author	Use of review in guideline	Guideline citation
							Medical Societies		10.6101/AZQ/000469. www.asthma.versorgungsleitlinien.de
CD009910.pub2[9]			UK	July 2016	No	Alair bronchial thermoplasty system for adults with severe difficult to control asthma: Medtech innovation briefing	National Institute for Health and Care Excellence	Used to make a decision on a NICE submission	National Institute for Health and Care Excellence. Alair bronchial thermoplasty system for adults with severe difficult to control asthma. 2016. Medtech innovation briefing [MIB71] Available from: https://www.nice.org.uk/advice/mib71/chapter/Technology-overview
CD009910.pub2[9]			UK	2019	No	British guideline on the management of asthma	Scottish Intercollegiate Guidelines Network, British Thoracic Society	Giving information leading to a recommendation.	Scottish Intercollegiate Guidelines Network, British Thoracic Society. British guideline on the management of asthma. 2018. https://www.sign.ac.uk/media/1773/sign158-updated.pdf
CD009910.pub2[9]			UK	2018	No	Bronchial thermoplasty for severe asthma	National institute for Health and Care Excellence	Used to make a decision on a NICE submission	National Institute for Health and Care Excellence. Bronchial thermoplasty for severe asthma 2018. Interventional

Review	Review year	Number of citations	Guideline location	Guideline year	Different version of the same guideline cited for a review	Guideline titles	Guideline author	Use of review in guideline	Guideline citation
									procedures guidance [IPG635] Available from: https://www.nice.org.uk/guidance/ipg635
CD009910.pub2[9]			UK	2018	No	**Interventional procedure overview of bronchial thermoplasty for severe asthma**	National Institute for Health and Care Excellence	Used to make a decision on a NICE submission	National Institute for Health and Care Excellence. Interventional procedure overview of bronchial thermoplasty for severe asthma 2018. Interventional procedures guidance [IPG635] Available from: https://www.nice.org.uk/guidance/ipg635/evidence
CD009910.pub2[9]			Malaysia	2017	No	**Management of Asthma in Adults**	Ministry of Health Malaysia, Malaysian Thoracic Society, Academy of Medicine Malaysia	Giving information leading to a recommendation.	Ministry of Health Malaysia, Malaysian Thoracic Society, Academy of Medicine Malaysia. Management of Asthma in Adults. 2017. https://www.acadmed.org.my/index.cfm?&menuid=67
CD009951.pub3[10]	2021	1	Canada	2023	No	**Clinical Practice Guideline: Hypertensive**	Association of Ontario Midwives	Giving information leading to a	Association of Ontario Midwives. Hypertensive Disorders of Pregnancy.

Review	Review year	Number of citations	Guideline location	Guideline year	Different version of the same guideline cited for a review	Guideline titles	Guideline author	Use of review in guideline	Guideline citation
						**Disorders of Pregnancy**		recommendation.	2023 (Clinical Practice Guideline No. 15)
CD010204.pub2[11]	2016	5	Canada	2023	No	**A Guideline for the Clinical Management of Opioid Use Disorder**	British Columbia Centre on Substance Use, BC Ministry of Health, BC Ministry of Mental Health and Addictions	Giving information.	British Columbia Centre on Substance Use, BC Ministry of Health, and BC Ministry of Mental Health and Addictions. A Guideline for the Clinical Management of Opioid Use Disorder. Published November 2023. Available at: https://www.bccsu.ca/opioid-use-disorder/
CD010204.pub2[11]			Canada	2023	No	**Canadian Guideline for the Clinical Management of High-Risk Drinking and Alcohol Use Disorder**	Canadian Research Initiative in Substance Misuse	Giving information	Canadian Research Initiative in Substance Misuse. Canadian Guideline for the Clinical Management of High-Risk Drinking and Alcohol Use Disorder. October 2023. www.helpwithdrinking.ca
CD010204.pub2[11]			Australia	2019	No	**Clinical guideline for the diagnosis and management of**	Mazza, Brijnath, Chakraborty, Guideline	Giving information	Mazza, D., Brijnath, B., Chakraborty, S.P. and the Guideline Development Group. 2019. Clinical guideline

Review	Review year	Number of citations	Guideline location	Guideline year	Different version of the same guideline cited for a review	Guideline titles	Guideline author	Use of review in guideline	Guideline citation
						**work-related mental health conditions in general practice**	Development Group		for the diagnosis and management of work-related mental health conditions in general practice. Melbourne: Monash University
CD010204.pub2[11]			Canada	2017	No	**Crisis Intervention for Adults Using a Trauma-Informed Approach: Initial Four Weeks of Management (3rd Edition) Best Practice Guidelines**	Registered Nurses' Association of Ontario	Giving information	Registered Nurses' Association of Ontario. (2017). Crisis intervention for adults using a trauma-informed approach: Initial four weeks of management (3rd ed.). Toronto, ON: Author.
CD010204.pub2[11]			Canada	2019	No	**Provincial Guideline for the Clinical Management of High-Risk Drinking and Alcohol Use Disorder**	British Columbia Centre on Substance Use, BC Ministry of Health, BC Ministry of Mental Health and Addictions	Giving information	British Columbia Centre on Substance Use (BCCSU), B.C. Ministry of Health and B.C. Ministry of Mental Health and Addictions. Provincial Guideline for the Clinical Management of High-Risk Drinking and Alcohol Use Disorder. 2019. Vancouver, B.C.: BCCSU. Available at:

Review	Review year	Number of citations	Guideline location	Guideline year	Different version of the same guideline cited for a review	Guideline titles	Guideline author	Use of review in guideline	Guideline citation
									https://www.bccsu.ca/clinical-care-guidance/
CD010526.pub3[12]	2019	2	Finland	2023	No	[Duodecim Current Care Guidelines: Tooth replacement treatment]	Finnish Medical Society Duodecim, Finnish Dental Association Apollonia	Giving information	Tooth filling. Current Care Recommendation. Finnish Medical Society Duodecim and a working group appointed by the Finnish Dental Society Apollonia. Helsinki: Finnish Finnish Medical Society Duodecim, 2023 Available: www.kaypahoito.fi
CD010526.pub3[12]			UK	2022	No	The use of general anaesthesia in special care dentistry: A clinical guideline from the British Society for Disability and Oral Health	British Society for Disability and Oral Health	Referenced alongside a recommendation.	DOI: 10.1111/scd.12652
CD011027.pub3[13]			Finland	2022	Yes	[Duodecim Current Care Guidelines: Gestational diabetes]	Finnish Medical Society Duodecim, Medical Council of the	Cannot access full version.	Gestational. Current Care Recommendation. The Finnish Medical Society Duodecim, the Medical Council of the Finnish Diabetes

Review	Review year	Number of citations	Guideline location	Guideline year	Different version of the same guideline cited for a review	Guideline titles	Guideline author	Use of review in guideline	Guideline citation
							Finnish Diabetes Association, Finnish Gynaecological Association		Association and the Finnish Diabetes Association Working group appointed by the Finnish Association of Gynaecologists. Helsinki: Finnish Medical Society Duodecim, 2022 Available online: www.kaypahoito.fi
CD011027.pub3[13]	2022	2	Finland	2024	Yes	[Duodecim Current Care Guidelines: Gestational diabetes]	Finnish Medical Society Duodecim, Medical Council of the Finnish Diabetes Association, Finnish Gynaecological Association	Giving information	Gestational. Current Care Recommendation. The Finnish Medical Society Duodecim, the Medical Council of the Finnish Diabetes Association and the Finnish Diabetes Association Working group appointed by the Finnish Association of Gynaecologists. Helsinki: Finnish Medical Society Duodecim, 2024 Available online: www.kaypahoito.fi
CD011314.pub2[14]	2017	5	Ukraine	2024	No	[Hepatocellular Carcinoma, Evidence-based	The State Expert Center of the Ministry of	Giving information	The State Expert Center of the Ministry of Health of Ukraine. [Hepatocellular

Review	Review year	Number of citations	Guideline location	Guideline year	Different version of the same guideline cited for a review	Guideline titles	Guideline author	Use of review in guideline	Guideline citation
						Clinical Guidelines]	Health of Ukraine		Carcinoma, Evidence-based Clinical Guidelines]. 2024. Available from: https://www.dec.gov.ua/wp-content/uploads/2024/01/2024_gczk_kn.pdf
CD011314.pub2[14]			Europe	2018	No	EASL Clinical Practice Guidelines: Management of hepatocellular carcinoma	European Association for the Study of the Liver	Giving information leading to a recommendation.	DOI: 10.1016/j.jhep.2018.03.019
CD011314.pub2[14]			USA	2020	Yes	NCCN Clinical Practice Guidelines in Oncology: Hepatobiliary Cancers, Version 2.2020	National Comprehensive Cancer Network	Cannot access	National Comprehensive Cancer Network. NCCN Clinical Practice Guidelines in Oncology: Hepatobiliary Cancers. 2020.
CD011314.pub2[14]			USA	2021	Yes	NCCN Clinical Practice Guidelines in Oncology: Hepatobiliary Cancers, Version 1.2021	National Comprehensive Cancer Network	Cannot access	National Comprehensive Cancer Network. NCCN Clinical Practice Guidelines in Oncology: Hepatobiliary Cancers. 2021.
CD011314.pub2[14]			USA	2022	Yes	NCCN Clinical Practice	National Comprehensiv	Cannot access	National Comprehensive Cancer Network. NCCN

Review	Review year	Number of citations	Guideline location	Guideline year	Different version of the same guideline cited for a review	Guideline titles	Guideline author	Use of review in guideline	Guideline citation
						Guidelines in Oncology: Hepatobiliary Cancers, Version 5.2022	e Cancer Network		Clinical Practice Guidelines in Oncology: Hepatobiliary Cancers. 2022.
CD011710.pub3[15]	2021	1	USA	2023	No	VA/DoD Clinical Practice Guideline for the Management of Posttraumatic Stress Disorder and Acute Stress Disorder. Version 4.0	The Management of Posttraumatic Stress Disorder Work Group	Referenced alongside a recommendation.	Management of Posttraumatic Stress Disorder and Acute Stress Disorder Work Group. VA/DoD Clinical Practice Guideline for the Management of Posttraumatic Stress Disorder and Acute Stress Disorder. Version 4.0. 2023. https://www.healthquality.va.gov/guidelines/MH/ptsd/VA-DoD-CPG-PTSD-Full-CPG.pdf
CD011979.pub2[16]	2017	1	Germany	2023	No	[S3 guideline Local therapy for difficult-to-heal and/or chronic wounds due to peripheral arterial disease, diabetes mellitus or	German Society for Wound Healing and Wound Treatment	Giving information	German Society for Wound Healing and Wound Treatment. [S3 guideline Local therapy for difficult-to-heal and/or chronic wounds due to peripheral arterial disease, diabetes mellitus or chronic venous insufficiency].

Review	Review year	Number of citations	Guideline location	Guideline year	Different version of the same guideline cited for a review	Guideline titles	Guideline author	Use of review in guideline	Guideline citation
						chronic venous insufficiency]			2023. Available from: https://register.awmf.org/assets/guidelines/091-001l_S3_Lokaltherapie-schwerheilender-chronischer-Wunden_2023-11.pdf
CD012203.pub2[17]	2018	2	Ireland	2023	No	National Clinical Practice Guideline: Stillbirth: Prevention, Investigation, Management and Care	The National Women and Infants Programme, Institute of Obstetricians and Gynaecologists of the Royal College of Physicians of Ireland	Providing information	McDonnell A, Butler M, White J, Escañuela Sánchez T, Cullen S, Cotter R, Murphy M, O'Donoghue K. National Clinical Practice Guideline: Stillbirth: Prevention, Investigation, Management and Care. National Women and Infants Health Programme and The Institute of Obstetricians and Gynaecologists. January 2023
CD012203.pub2[17]			Australia	2023	No	Stillbirth care	Queensland Maternity and Neonatal Clinical Guidelines Program	Giving information	Queensland Clinical Guidelines. Stillbirth care. Guideline No. MN23.24-V10-R28. Queensland Health. 2023. Available from:

Review	Review year	Number of citations	Guideline location	Guideline year	Different version of the same guideline cited for a review	Guideline titles	Guideline author	Use of review in guideline	Guideline citation
									http://www.health.qld.gov.au/qcg
CD012522.pub2[18]	2018	2	UK	2022	No	Fractures (complex): assessment and management	National Institute for Health and Care Excellence	Used to make a decision on a NICE submission	National Institute for Health and Care Excellence. Fractures (complex): assessment and management 2022. NICE guideline [NG37] Available from: https://www.nice.org.uk/guidance/ng37
CD012522.pub2[18]			UK	2021	No	Medical technologies guidance: The VAC Veraflo Therapy system for acute infected or chronic wounds that are failing to heal	National Institute for Health and Care Excellence	Used to make a decision on a NICE submission	National Institute for Health and Care Excellence. Medical technologies guidance: The VAC Veraflo Therapy system for acute infected or chronic wounds that are failing to heal 2021. Medical technologies guidance [MTG54] Available from: https://www.nice.org.uk/guidance/mtg54/history
CD013040.pub2[19]	2021	5	Global	2021	Yes	Global strategy for the diagnosis, management and prevention of chronic	Global Initiative for Chronic Obstructive Lung Disease	Giving information	Global Initiative for Chronic Obstructive Lung Disease. Global strategy for the diagnosis, management and prevention of chronic

Review	Review year	Number of citations	Guideline location	Guideline year	Different version of the same guideline cited for a review	Guideline titles	Guideline author	Use of review in guideline	Guideline citation
						**obstructive pulmonary disease: 2022 report**			obstructive pulmonary disease: 2022 report. 2021. Available from: https://goldcopd.org/wp - content/uploads/2021/1 2/GOLD-REPORT-2022- v1.1- 22Nov2021_WMV.pdf
CD013040.pub2[19]			Global	2023	Yes	**Global strategy for the diagnosis, management and prevention of chronic obstructive pulmonary disease: 2023 report**	Global Initiative for Chronic Obstructive Lung Disease	Giving information	Global Initiative for Chronic Obstructive Lung Disease. Global strategy for the diagnosis, management and prevention of chronic obstructive pulmonary disease: 2023 report. 2023. Available from: https://goldcopd.org/20 23-gold-report-2/
CD013040.pub2[19]			Global	2024	Yes	**Global strategy for the diagnosis, management and prevention of chronic obstructive pulmonary disease: 2024 report**	Global Initiative for Chronic Obstructive Lung Disease	Giving information	Global Initiative for Chronic Obstructive Lung Disease. Global strategy for the diagnosis, management and prevention of chronic obstructive pulmonary disease: 2024 report. 2024. Available from: https://goldcopd.org/wp

Review	Review year	Number of citations	Guideline location	Guideline year	Different version of the same guideline cited for a review	Guideline titles	Guideline author	Use of review in guideline	Guideline citation
									-content/uploads/2024/01/GOLD-2024_v1.2-11Jan24_WMV-1.pdf
CD013040.pub2[19]			USA	2023	No	**Pulmonary Rehabilitation for Adults with Chronic Respiratory Disease: An Official American Thoracic Society Clinical Practice Guideline**	American Thoracic Society	Giving information leading to a recommendation	DOI: 10.1164/rccm.202306-1066ST
CD013040.pub2[19]			Australia and New Zealand	2023	No	**The COPD-X Plan: Australian and New Zealand Guidelines for the management of Chronic Obstructive Pulmonary Disease 2023**	Lung Foundation Australia, Thoracic Society of Australia and New Zealand	Providing information	Yang IA, Dabscheck E, George J, McNamara R, McDonald CF, McDonald V, Smith B, Zwar N; on behalf of the Lung Foundation Australia and the Thoracic Society of Australia and New Zealand. The COPD-X Plan: Australian and New Zealand Guidelines for the management of Chronic Obstructive Pulmonary Disease 2023.

Review	Review year	Number of citations	Guideline location	Guideline year	Different version of the same guideline cited for a review	Guideline titles	Guideline author	Use of review in guideline	Guideline citation
									Version 2.7, March 2023. Sydney, NSW, Australia: Lung Foundation Australia and Thoracic Society of Australia and New Zealand; 2023. Available from: https://copdx.org.au/wp-content/uploads/2023/09/WEBSITE_COPDX-V2-70_FINAL.pdf
CD013319.pub2[20]	2019	1	UK	2021	No	**Heart valve disease presenting in adults: investigation and management**	National Guideline Centre, National Institute for Health and Care Excellence (commissioner)	Used to make a decision on a NICE submission	National Institute for Health and Care Excellence. Heart valve disease presenting in adults: investigation and management 2021. NICE guideline [NG208] Available from: https://www.nice.org.uk/guidance/ng208
CD013343.pub2[21]	2022	2	Global	2024	No	**Global strategy for the diagnosis, management and prevention of chronic obstructive pulmonary**	Global Initiative for Chronic Obstructive Lung Disease	Giving information	Global Initiative for Chronic Obstructive Lung Disease. Global strategy for the diagnosis, management and prevention of chronic obstructive pulmonary disease: 2024 report.

Review	Review year	Number of citations	Guideline location	Guideline year	Different version of the same guideline cited for a review	Guideline titles	Guideline author	Use of review in guideline	Guideline citation
						**disease: 2024 report**			2024. Available from: https://goldcopd.org/wp-content/uploads/2024/01/GOLD-2024_v1.2-11Jan24_WMV-1.pdf
CD013343.pub2[21]			Australia and New Zealand	2023	No	**The COPD-X Plan: Australian and New Zealand Guidelines for the management of Chronic Obstructive Pulmonary Disease 2023**	Lung Foundation Australia, Thoracic Society of Australia and New Zealand	Giving information	Yang IA, Dabscheck E, George J, McNamara R, McDonald CF, McDonald V, Smith B, Zwar N; on behalf of the Lung Foundation Australia and the Thoracic Society of Australia and New Zealand. The COPD-X Plan: Australian and New Zealand Guidelines for the management of Chronic Obstructive Pulmonary Disease 2023. Version 2.7, March 2023. Sydney, NSW, Australia: Lung Foundation Australia and Thoracic Society of Australia and New Zealand; 2023. Available from: https://copdx.org.au/wp-content/uploads/2023/0

Review	Review year	Number of citations	Guideline location	Guideline year	Different version of the same guideline cited for a review	Guideline titles	Guideline author	Use of review in guideline	Guideline citation
									9/WEBSITE_COPDX-V2-70_FINAL.pdf
CD013410.pub2[22]	2022	1	UK	2023	No	The management of hip fracture in adults	National Institute for Health and Care Excellence	Used to make a decision on a NICE submission	National Institute for Health and Care Excellence. The management of hip fracture in adults 2023. Clinical guideline [CG124] Available from: https://www.nice.org.uk/guidance/cg124/
CD014915.pub[23]	2023	1	USA	2023	No	NCCN Clinical Practice Guidelines in Oncology: Adult Cancer Pain, Version 2.2023	National Comprehensive Cancer Network	Cannot access	National Comprehensive Cancer Network. NCCN Clinical Practice Guidelines in Oncology: Hepatobiliary Cancers. 2023. https://www.nccn.org/guidelines/guidelines-detail?category=1&id=1438
CD015017.pub3[24]	2022	3	Netherlands	2022	Yes	[Dutch College of General Practitioners – Guideline on COVID-19 (version 1.4 - Guideline M111]	Dutch College of General Practitioners	Cannot access.	Dutch College of General Practitioners. [Dutch College of General Practitioners – Guideline on COVID-19 (version 1.4 - Guideline M111]. 2022.

Review	Review year	Number of citations	Guideline location	Guideline year	Different version of the same guideline cited for a review	Guideline titles	Guideline author	Use of review in guideline	Guideline citation
CD015017.pub3[24]			Netherlands	2023	Yes	**[Dutch College of General Practitioners – Guideline on COVID-19 (version 1.5 – Guideline M111)]**	Dutch College of General Practitioners	Cannot access.	Dutch College of General Practitioners. [Dutch College of General Practitioners – Guideline on COVID-19 (version 1.5 – Guideline M111)]. 2023.
CD015017.pub3[24]			Netherlands	2023	Yes	**[Dutch College of General Practitioners – Guideline on COVID-19 (version 2 – NHG Guideline M111)]**	Dutch College of General Practitioners	Providing information	Dutch College of General Practitioners. [Dutch College of General Practitioners – Guideline on COVID-19 (version 2 – NHG Guideline M111)]. 2023. Available from: https://richtlijnen.nhg.org/files/pdf/1682_COVID-19_november-2023.pdf

1. Jones E, Stewart F, Taylor B, Davis PG, Brown SJ. Early postnatal discharge from hospital for healthy mothers and term infants. *Cochrane Database Syst Rev* 2021(6) doi: 10.1002/14651858.CD002958.pub2
2. Cook LA, Pun A, Gallo MF, Lopez LM, Van Vliet H. Scalpel versus no-scalpel incision for vasectomy. *Cochrane Database Syst Rev* 2014(3) doi: 10.1002/14651858.CD004112.pub4
3. Pinart M, Rueda JR, Romero GAS, et al. Interventions for American cutaneous and mucocutaneous leishmaniasis. *Cochrane Database Syst Rev* 2020(8) doi: 10.1002/14651858.CD004834.pub3
4. Filippini T, Malavolti M, Borrelli F, et al. Green tea (Camellia sinensis) for the prevention of cancer. *Cochrane Database Syst Rev* 2020(3) doi: 10.1002/14651858.CD005004.pub3
5. Rittiphairoj T, Mir TA, Li T, Virgili G. Intravitreal steroids for macular edema in diabetes. *Cochrane Database Syst Rev* 2020(11) doi: 10.1002/14651858.CD005656.pub3

6. Kahale LA, Matar CF, Hakoum MB, et al. Anticoagulation for the initial treatment of venous thromboembolism in people with cancer. *Cochrane Database Syst Rev* 2021(12) doi: 10.1002/14651858.CD006649.pub8
7. Williams MJ, Ramson JA, Brownfoot FC. Different corticosteroids and regimens for accelerating fetal lung maturation for babies at risk of preterm birth. *Cochrane Database Syst Rev* 2022(8) doi: 10.1002/14651858.CD006764.pub4
8. Cates CJ, Schmidt S, Ferrer M, Sayer B, Waterson S. Inhaled steroids with and without regular salmeterol for asthma: serious adverse events. *Cochrane Database Syst Rev* 2018(12) doi: 10.1002/14651858.CD006922.pub4
9. Torrego A, Solà I, Munoz AM, et al. Bronchial thermoplasty for moderate or severe persistent asthma in adults. *Cochrane Database Syst Rev* 2014(3) doi: 10.1002/14651858.CD009910.pub2
10. Davidson SJ, Barrett HL, Price SA, Callaway LK, Dekker Nitert M. Probiotics for preventing gestational diabetes. *Cochrane Database Syst Rev* 2021(4) doi: 10.1002/14651858.CD009951.pub3
11. Roberts NP, Roberts PA, Jones N, Bisson JI. Psychological therapies for post-traumatic stress disorder and comorbid substance use disorder. *Cochrane Database Syst Rev* 2016(4) doi: 10.1002/14651858.CD010204.pub2
12. Schenkel AB, Veitz-Keenan A. Dental cavity liners for Class I and Class II resin-based composite restorations. *Cochrane Database Syst Rev* 2019(3) doi: 10.1002/14651858.CD010526.pub3
13. Edwards T, Liu G, Battin M, et al. Oral dextrose gel for the treatment of hypoglycaemia in newborn infants. *Cochrane Database Syst Rev* 2022(3) doi: 10.1002/14651858.CD011027.pub3
14. Abdel-Rahman O, Elsayed Z. External beam radiotherapy for unresectable hepatocellular carcinoma. *Cochrane Database Syst Rev* 2017(3) doi: 10.1002/14651858.CD011314.pub2
15. Simon N, Robertson L, Lewis C, et al. Internet-based cognitive and behavioural therapies for post-traumatic stress disorder (PTSD) in adults. *Cochrane Database Syst Rev* 2021(5) doi: 10.1002/14651858.CD011710.pub3
16. Wang HT, Yuan JQ, Zhang B, et al. Phototherapy for treating foot ulcers in people with diabetes. *Cochrane Database Syst Rev* 2017(6) doi: 10.1002/14651858.CD011979.pub2
17. Wojcieszek AM, Shepherd E, Middleton P, et al. Care prior to and during subsequent pregnancies following stillbirth for improving outcomes. *Cochrane Database Syst Rev* 2018(12) doi: 10.1002/14651858.CD012203.pub2
18. Iheozor-Ejiofor Z, Newton K, Dumville JC, et al. Negative pressure wound therapy for open traumatic wounds. *Cochrane Database Syst Rev* 2018(7) doi: 10.1002/14651858.CD012522.pub2
19. Cox NS, Dal Corso S, Hansen H, et al. Telerehabilitation for chronic respiratory disease. *Cochrane Database Syst Rev* 2021(1) doi: 10.1002/14651858.CD013040.pub2
20. Kolkailah AA, Doukky R, Pelletier MP, et al. Transcatheter aortic valve implantation versus surgical aortic valve replacement for severe aortic stenosis in people with low surgical risk. *Cochrane Database Syst Rev* 2019(12) doi: 10.1002/14651858.CD013319.pub2
21. Fraser A, Poole P. Immunostimulants versus placebo for preventing exacerbations in adults with chronic bronchitis or chronic obstructive pulmonary disease. *Cochrane Database Syst Rev* 2022(11) doi: 10.1002/14651858.CD013343.pub2

22. Lewis SR, Macey R, Parker MJ, Cook JA, Griffin XL. Arthroplasties for hip fracture in adults. *Cochrane Database Syst Rev* 2022(2) doi: 10.1002/14651858.CD013410.pub2
23. Häuser W, Welsch P, Radbruch L, et al. Cannabis-based medicines and medical cannabis for adults with cancer pain. *Cochrane Database Syst Rev* 2023(6) doi: 10.1002/14651858.CD014915.pub2
24. Popp M, Reis S, Schießer S, et al. Ivermectin for preventing and treating COVID-19. *Cochrane Database Syst Rev* 2022(6) doi: 10.1002/14651858.CD015017.pub3
