# Supplementary material for: The impact of Cochrane Reviews that apply network meta-analysis in clinical guidelines: A systematic review
Source: PLoS One. 2024 Dec 26;19(12):e0315563. doi: 10.1371/journal.pone.0315563 (PMC11671017; doi:10.1371/journal.pone.0315563)
Supplement: S7 Table — (PDF) [file pone.0315563.s013.pdf]

Table S7: NMA and PW-MA review characteristics

Characteristic		Number of NMAs not cited in guidelines (N=20)	Number of NMAs cited in guidelines (N=18)	NMAs	Number of PW-MAs not cited in guidelines (N=14)	Number of PW-MAs cited in guidelines (N=24)	PW-MAs
Review Group (n (%))	Airways	1 (25)	3 (75)	4	0 (0)	4 (100)	4
	Bone, Joint & Muscle Trauma	2 (100)	0 (0)	2	1 (50)	1 (50)	2
	Common Mental Disorders	0 (0)	2 (100)	2	0 (0)	2 (100)	2
	Epilepsy	0 (0)	1 (100)	1	1 (100)	0 (0)	1
	Eyes & Vision	2 (67)	1 (33)	3	2 (67)	1 (33)	3
	Fertility Regulation	0 (0)	1 (100)	1	0 (0)	1 (100)	1
	Gut	1 (100)	0 (0)	1	1 (100)	0 (0)	1
	Gynaecological, Neuro-oncology & Orphan Cancer	3 (100)	0 (0)	3	1 (33)	2 (67)	3
	Gynaecology & Fertility	1 (100)	0 (0)	1	1 (100)	0 (0)	1
	Haematology	1 (100)	0 (0)	1	0 (0)	1 (100)	1
	Heart	1 (100)	0 (0)	1	0 (0)	1 (100)	1
	Hepato-Biliary	0 (0)	2 (100)	2	1 (50)	1 (50)	2
	Kidney & Transplant	1 (100)	0 (0)	1	1 (100)	0 (0)	1
	Neonatal	2 (100)	0 (0)	2	1 (50)	1 (50)	2
	Oral Health	0 (0)	1 (100)	1	0 (0)	1 (100)	1
	Pain, Palliative & Supportive Care	2 (100)	0 (0)	2	1 (50)	1 (50)	2
	Pregnancy & Childbirth	0 (0)	4 (100)	4	0 (0)	4 (100)	4
	Skin	0 (0)	1 (100)	1	0 (0)	1 (100)	1
	Urology	2 (100)	0 (0)	2	2 (100)	0 (0)	2
	Work	0 (0)	1 (100)	1	1 (100)	0 (0)	1
	Wounds	1 (50)	1 (50)	2	0 (0)	2 (100)	2
Sources of support (n (%))	Government	3 (100)	0 (0)	3	3 (23)	10 (77)	13
	Not reported	1 (50)	1 (50)	2	3 (100)	0 (0)	3
	Health agency	1 (100)	0 (0)	1	0 (0)	0 (0)	0
	Hospitals	1 (100)	0 (0)	1	0 (0)	0 (0)	0
	Funding bodies	10 (50)	10 (50)	20	5 (83)	1 (17)	6
	Funding body and government	1 (33)	2 (67)	3	0 (0)	2 (100)	2
	Funding body and hospitals	1 (100)	0 (0)	1	0 (0)	2 (100)	2
	Hospitals and charity	1 (100)	0 (0)	1	0 (0)	0 (0)	0
	Funding body and Health Agency.	0 (0)	0 (0)	0	2 (67)	1 (33)	3
	Funding body and charity	1 (50)	1 (50)	2	0 (0)	0 (0)	0
	Funding body, government, and health agency	0 (0)	1 (100)	1	0 (0)	1 (100)	1
	Funding body, health agency, hospitals	0 (0)	1 (100)	1	0 (0)	1 (100)	1
	Funding body, hospitals, government	0 (0)	1 (100)	1	1 (33)	2 (67)	3
	Funding body, health agency, hospitals, charity	0 (0)	1 (100)	1	0 (0)	0 (0)	0
	Funding body, charity, Cochrane Centre, agency	0 (0)	0 (0)	0	0 (0)	1 (100)	1
	Funding body, Health agency, charity	0 (0)	0 (0)	0	0 (0)	1 (100)	1
	Hospital; company	0 (0)	0 (0)	0	1 (100)	0 (0)	1
	Cochrane Centre, Health agency	0 (0)	0 (0)	0	0 (0)	1 (100)	1
Studies (n (%))	RCTs	17 (53)	15 (47)	32	10 (36)	18 (64)	28
	RCTs (including quasi-RCTs)	2 (50)	2 (50)	4	2 (33)	4 (67)	6
	RCTs and non-RCTs	1 (50)	1 (50)	2	2 (50)	2 (50)	4
