# Supplementary material for: The impact of Cochrane Reviews that apply network meta-analysis in clinical guidelines: A systematic review
Source: PLoS One. 2024 Dec 26;19(12):e0315563. doi: 10.1371/journal.pone.0315563 (PMC11671017; doi:10.1371/journal.pone.0315563)
Supplement: S8 Table — (PDF) [file pone.0315563.s014.pdf]

Table S8: Characteristics of the NMAs and PW-MAs for the first outcome

Characteristic		Number of NMAs not cited in guidelines (N=20)	Number of NMAs cited in guidelines (N=18)	Total NMAs	Number of PW-MAs not cited in guidelines (N=14)	Number of PW-MAs cited in guidelines (N=24)	Total PW-MAs
**Number of interventions (median, range)**		7.5 (4-24)	6.5 (3-25)	7 (3-25)	2.5 (2-5)	2.5 (2-12)	2.5 (2-12)
**Number of trials (median, range)**		22 (4-56)	22 (3-124)	22 (3-124)	9.5 (2-76)	11 (2-142)	11 (2-142)
**Number of patients (median, range)**		3,540 (1,013-21,542)	4,826 (737-27,024)	4,167 (737-27,024)	1,106.5 (159-10,979)	1,647 (222-1,101,795)	1,615 (159-1,101,795)
**Grade (n (%))**	Lowest grading was very low for at least one comparison	11 (61)	7 (39)	18	7 (41)	10 (59)	17
	Lowest grading was low for at least one comparison	8 (62)	5 (38)	13	4 (36)	7 (64)	11
	Lowest grading was moderate for at least one comparison	1 (50)	1 (50)	2	1 (14)	6 (86)	7
	High for all comparisons	0 (0)	1 (100)	1	1 (100)	0 (0)	1
	Threshold analysis/ not reported	0 (0)	4 (100)	4	1 (50)	1 (50)	2
**Type of analysis (n (%))**	Frequentist	16 (53)	14 (47)	30	14 (37)	24 (63)	38
	Bayesian	3 (60)	2 (40)	5	0 (0)	0 (0)	0
	Frequentist and Bayesian	1 (33)	2 (67)	3	0 (0)	0 (0)	0
**Risk of bias (n (%))**	Low	2 (67)	1 (33)	3	2 (67)	1 (33)	3
	Low and unclear or not reported but all trials in review are unclear or low	13 (50)	13 (50)	26	8 (32)	17 (68)	25
	One or more high risk trial in analysis	5 (71)	2 (29)	7	3 (60)	2 (40)	5
	Not reported	0 (0)	2 (100)	2	1 (20)	4 (80)	5
**Heterogeneity present (n (%))**	Yes	8 (47)	9 (53)	17	4 (33)	8 (67)	12
	no	5 (56)	4 (44)	9	10 (37)	16 (62)	26
	Not reported/applied	7 (58)	5 (42)	12	0 (0)	0 (0)	0
**Inconsistency present (n (%))**	Yes	4 (57)	3 (43)	7	-	-	-
	No	12 (55)	10 (45)	22	-	-	-
	Not reported/applied	4 (44)	5 (56)	9	-	-	-
