# Supplementary material for: The impact of Cochrane Reviews that apply network meta-analysis in clinical guidelines: A systematic review
Source: PLoS One. 2024 Dec 26;19(12):e0315563. doi: 10.1371/journal.pone.0315563 (PMC11671017; doi:10.1371/journal.pone.0315563)
Supplement: S9 Table — (PDF) [file pone.0315563.s015.pdf]

Table S9: Guideline groups that cited NMA and PW-MA reviews.

Guideline Group
**Cited NMA and PW reviews**
• Association of Ontario Midwives<br>• Co-authors<br>• Dutch College of General Practitioners<br>• German Society of Gynecology and Obstetrics, Austrian Society of Gynecology and Obstetrics, Swiss Society of Gynecology and Obstetrics<br>• Global Initiative for Chronic Obstructive Lung Disease<br>• Lung Foundation Australia, Thoracic Society of Australia and New Zealand<br>• National Institute for Health and Care Excellence<br>• Queensland Maternity and Neonatal Clinical Guidelines Program<br>• Registered Nurses' Association of Ontario<br>• The National Women and Infants Programme, Institute of Obstetricians and Gynaecologists of the Royal College of Physicians of Ireland<br>• World Health Organization
**Cited NMA reviews only**
• American Academy of Ophthalmology<br>• American Psychiatric Association<br>• American Society for Reproductive Medicine<br>• Australian Research Centre for Population Oral Health<br>• Canadian Paediatric Society Adolescent Health Committee<br>• Centre for Research Excellence in Women's Health in Reproductive Life, American Society of Reproductive Medicine, Endocrine Society, European Society of Endocrinology, European Society of Human Reproduction and Embryology<br>• Clinical Effectiveness Unit, Faculty of Sexual & Reproductive Healthcare, Royal College of Obstetricians & Gynaecologists<br>• Clinical Practice Guideline for Hormonal and Intrauterine Contraception Working Group<br>• Committee of the Taiwan Academy of Pediatric Allergy, Asthma and Immunology<br>• COPD Guideline Development Group<br>• European Academy of Paediatric Dentistry<br>• European Organization for Caries Research, European Federation of Conservative Dentistry, German Association of Conservative Dentistry<br>• European Psychiatric Association<br>• European Society for Vascular Surgery<br>• European Society of Cardiology and European Respiratory Society.<br>• Federation of Medical Specialists<br>• Finnish Medical Society Duodecim, Finnish Angiology Association, Finnish Cardiological Society<br>• Finnish Medical Society Duodecim, Finnish Association of Respiratory Physicians

Guideline Group
• Finnish Medical Society Duodecim, Finnish Psychiatric Association, Finnish Youth Psychiatric Association<br>• Finnish Medical Society Duodecim, the Finnish Society of Anaesthesiology, the Intensive Care Medicine Subdivision and the Finnish Nephrological Society<br>• German Medical Association, Association of German Medical Associations, National Association of Statutory Health Insurance Physicians, Association of Scientific Medical Societies.<br>• German Society for Gastroenterology, Digestive and Metabolic Diseases and the German Society of General and Visceral Surgery.<br>• German Society for Psychiatry and Psychotherapy, Psychosomatics and Neurology<br>• German Society for Psychosomatic Medicine and Medical Psychotherapy<br>• German Society for Ultrasound in Medicine, German Society for Gynaecology and Obstetrics.<br>• German Society of Gynaecology and Obstetrics, German Society for Midwifery Science<br>• German Society of Neurology<br>• German Society of Neurology, German Stroke Society<br>• Hepatobiliary Study Group of the Chinese Society of Gastroenterology of the Chinese Medical Association, Hepatology Committee of the Chinese Research Hospital Association<br>• PAPPS Women's Group<br>• Royal Australian College of General Practitioners<br>• Russian Society of Obstetricians and Gynecologists,<br>• Russian Society of Obstetricians and Gynecologists, Association of Anesthesiologists and Resuscitators, Association of Obstetric Anesthesiologists-Resuscitators, National Association of Patient Blood Management Specialists,<br>• Russian Society of Psychiatrists<br>• Society of Obstetricians and Gynaecologists of Canada<br>• South African Thoracic Society<br>• The Management of Chronic Obstructive Pulmonary Disease Work Group
**Cited PW reviews only**<br>• American Thoracic Society<br>• Association of German Medical Associations, National Association of Statutory Health Insurance Physicians, Association of Scientific Medical Societies<br>• British Columbia Centre on Substance Use, BC Ministry of Health, BC Ministry of Mental Health and Addictions<br>• British Society for Disability and Oral Health<br>• Canadian Research Initiative in Substance Misuse<br>• Dutch Association of Physicians for Lung Diseases and Tuberculosis.<br>• European Association for the Study of the Liver<br>• European Association of Urology<br>• Finnish Medical Society Duodecim, Finnish Dental Association Apollonia

Guideline Group
• Finnish Medical Society Duodecim, Medical Council of the Finnish Diabetes Association, Finnish Gynaecological Association<br>• Finnish Medical Society Duodecim, the Finnish Association of Pulmonologists, the Finnish Paediatric Association, the Allergology Association of Finnish Paediatricians, the Finnish Society of Clinical Physiology,<br>• German Society for Gastroenterology, Digestive and Metabolic Diseases<br>• German Society for Wound Healing and Wound Treatment<br>• Global Initiative for Asthma<br>• International Initiative on Thrombosis and Cancer (ITAC) advisory panel<br>• Ministry of Health Malaysia, Malaysian Thoracic Society, Academy of Medicine Malaysia<br>• National Comprehensive Cancer Network<br>• National Neonatology Forum of India<br>• Oncology Guideline Program<br>• (German Cancer Society; German Cancer Aid; The Association of Scientific Medical Societies)<br>• Saudi Initiative for Asthma Group, Saudi Thoracic Society<br>• Scottish Intercollegiate Guidelines Network, British Thoracic Society<br>• Spanish Association of Primary Care Paediatrics, Latin American Thoracic Association, Primary Care Respiratory Society, Spanish Society of Allergology and Clinical Immunology, Spanish Society of Family and Community Pharmacy, Spanish Society of Clinical Pharmacology, Spanish Society of Hospital Pharmacy, Spanish Society of Clinical Immunology, Allergology and Paediatric Asthma, Spanish Society of Primary Care Physicians, Spanish Society of Emergency Medicine, Spanish Society of Family and Community Medicine, Spanish Society of General and Family Physicians, Spanish Society of Paediatric Pneumology, Spanish Society of Otolaryngology and Head and Neck Surgery, Spanish Society of Pneumology and Thoracic Surgery, Spanish Society of Outpatient Paediatrics and Primary Care, Portuguese Society of Pneumology<br>• The Management of Posttraumatic Stress Disorder Work Group<br>• The Society of Obstetricians and Gynaecologists of Canada<br>• The State Expert Center of the Ministry of Health of Ukraine
