# Supplementary figures and images for: The impact of Cochrane Reviews that apply network meta-analysis in clinical guidelines: A systematic review
Source: PLoS One. 2024 Dec 26;19(12):e0315563. doi: 10.1371/journal.pone.0315563 (PMC11671017; doi:10.1371/journal.pone.0315563)

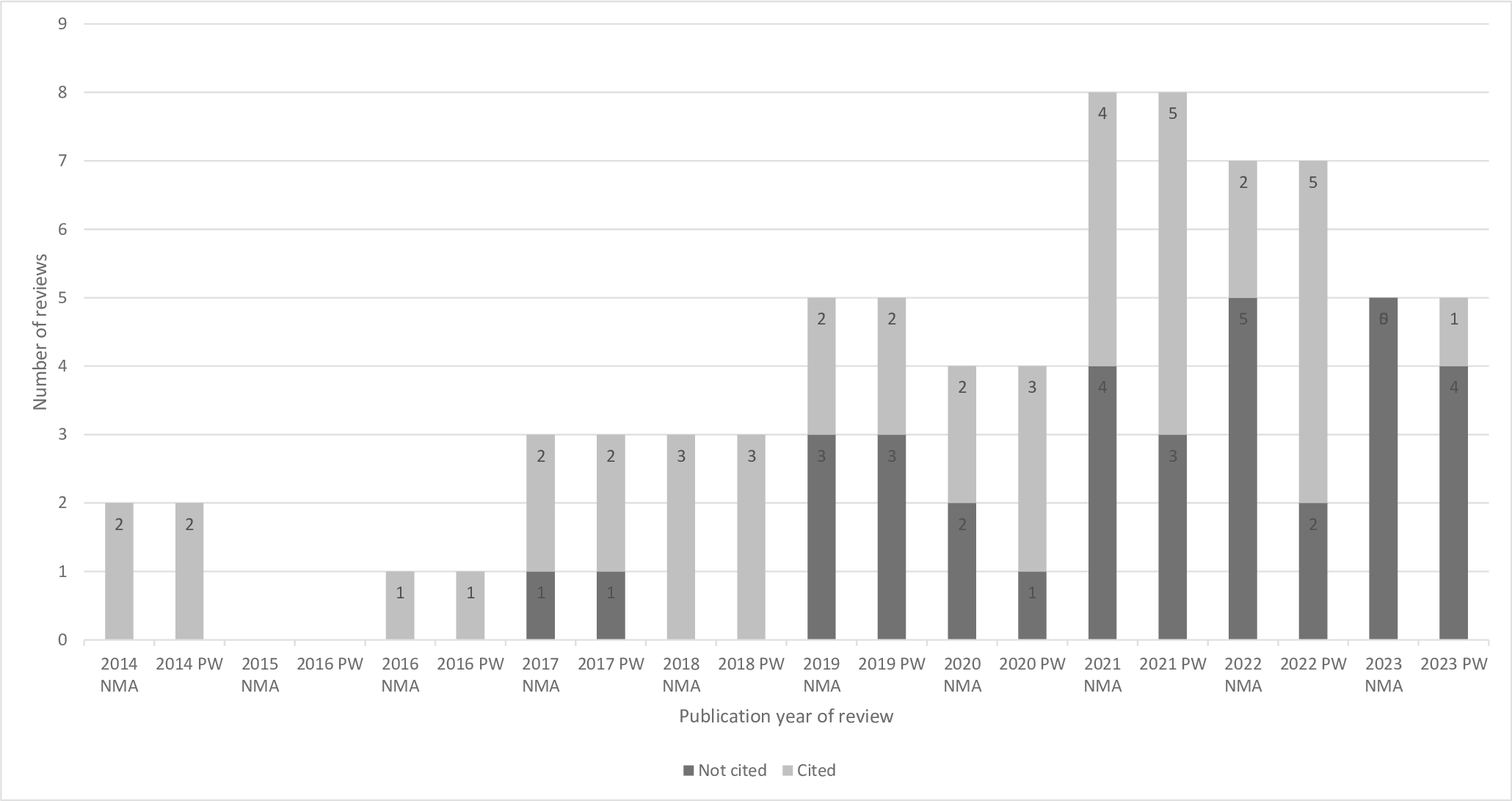

Supplement: S1 Fig — (TIF) [file pone.0315563.s016.tif]

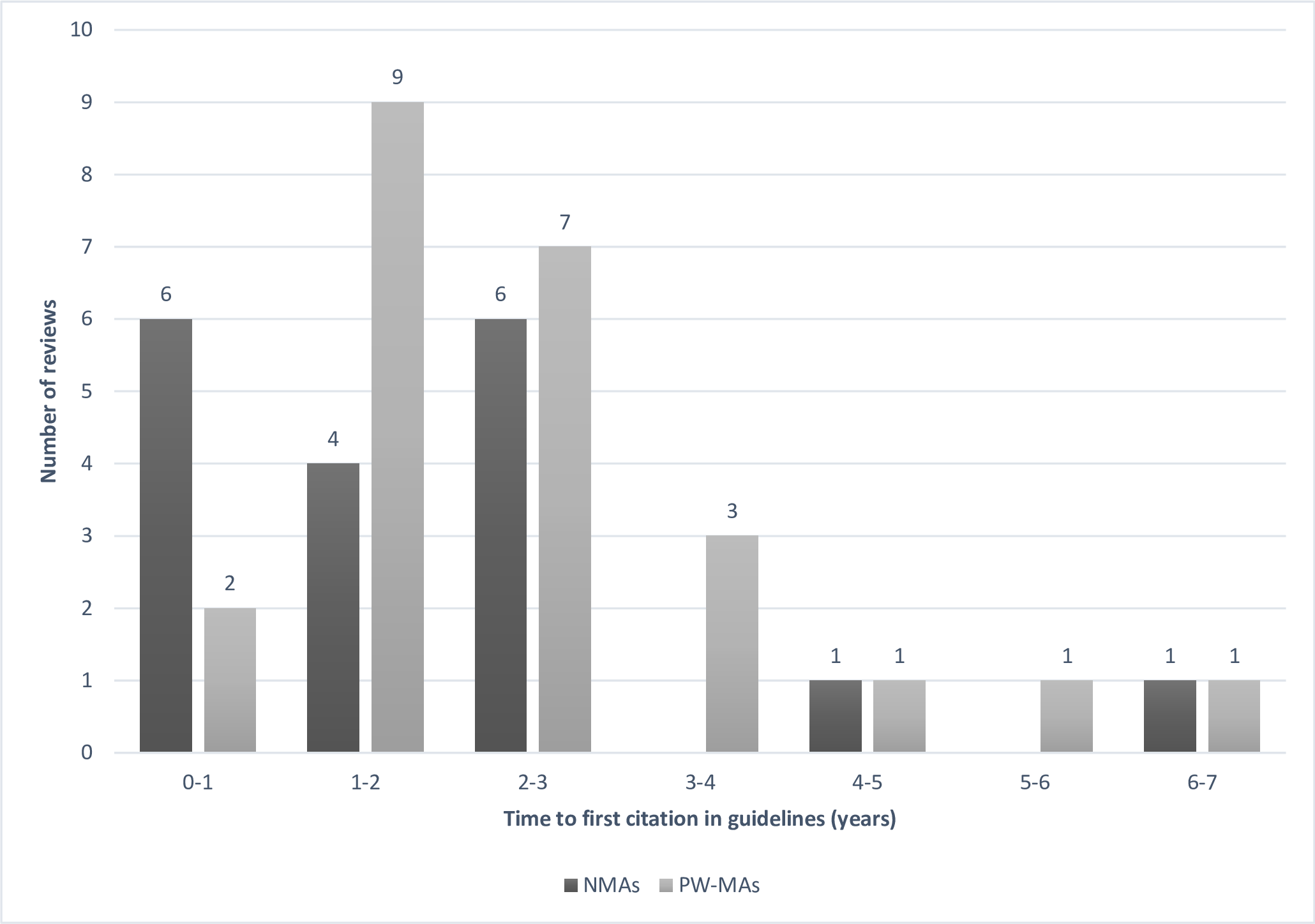

Supplement: S2 Fig — (TIF) [file pone.0315563.s017.tif]

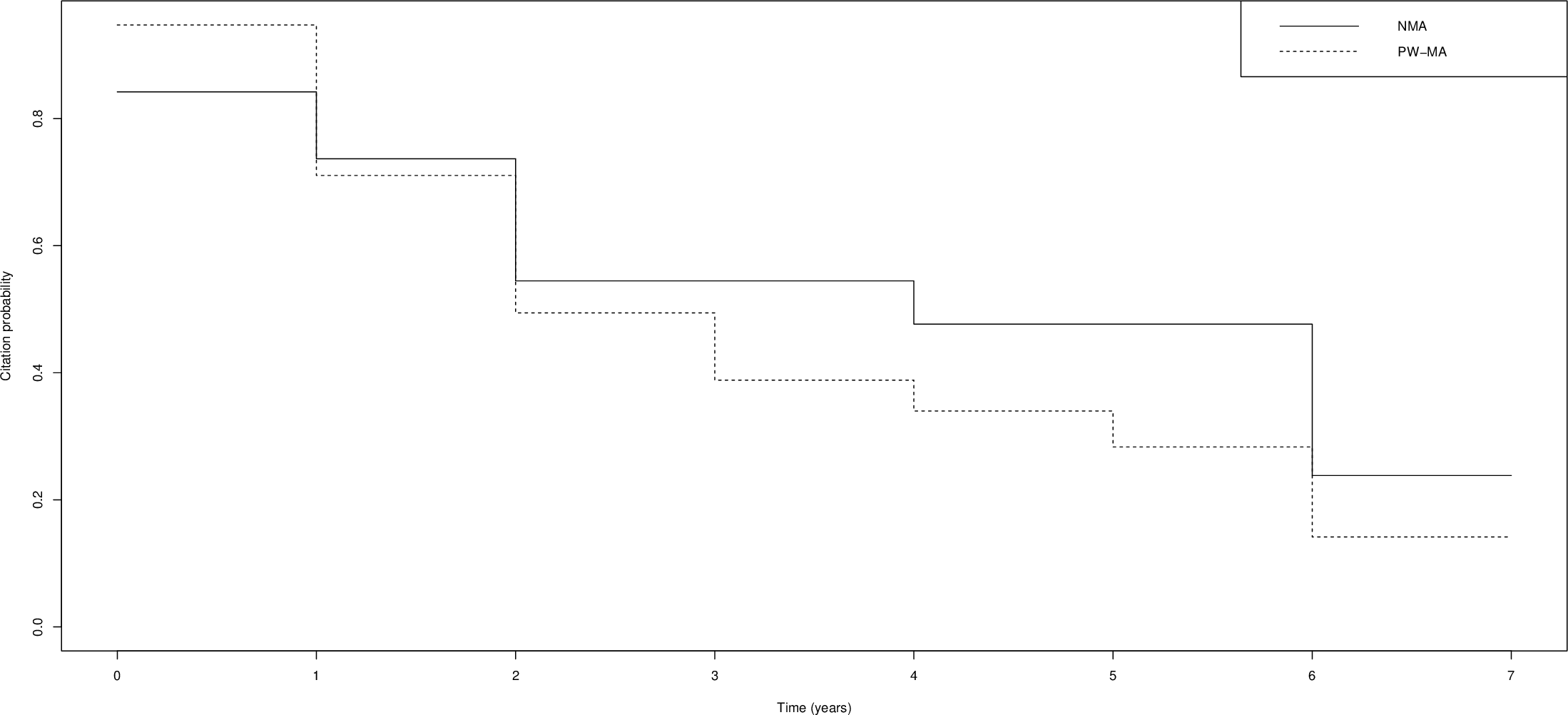

Supplement: S3 Fig — (TIF) [file pone.0315563.s018.tif]

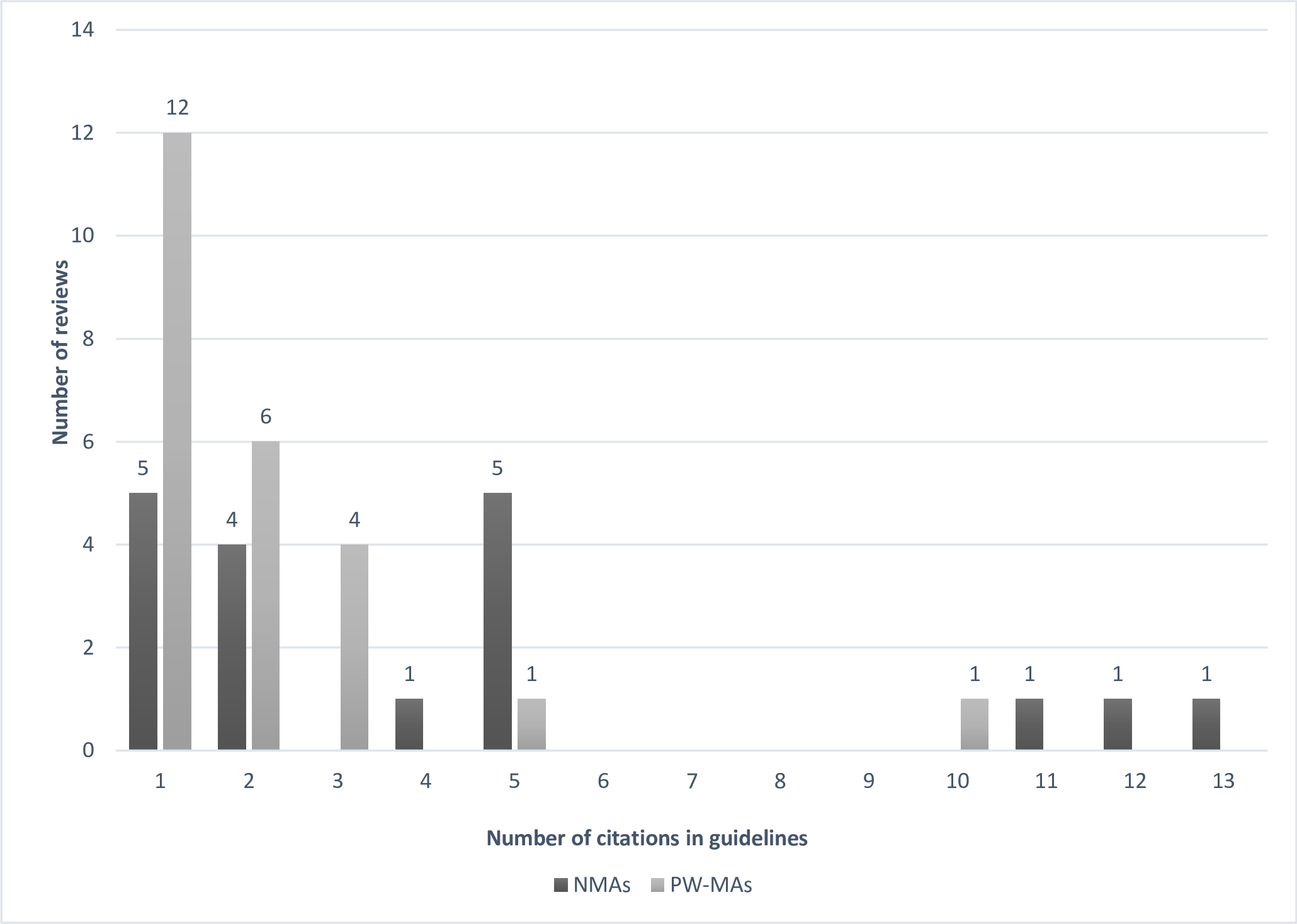

Supplement: S4 Fig — (TIF) [file pone.0315563.s019.tif]

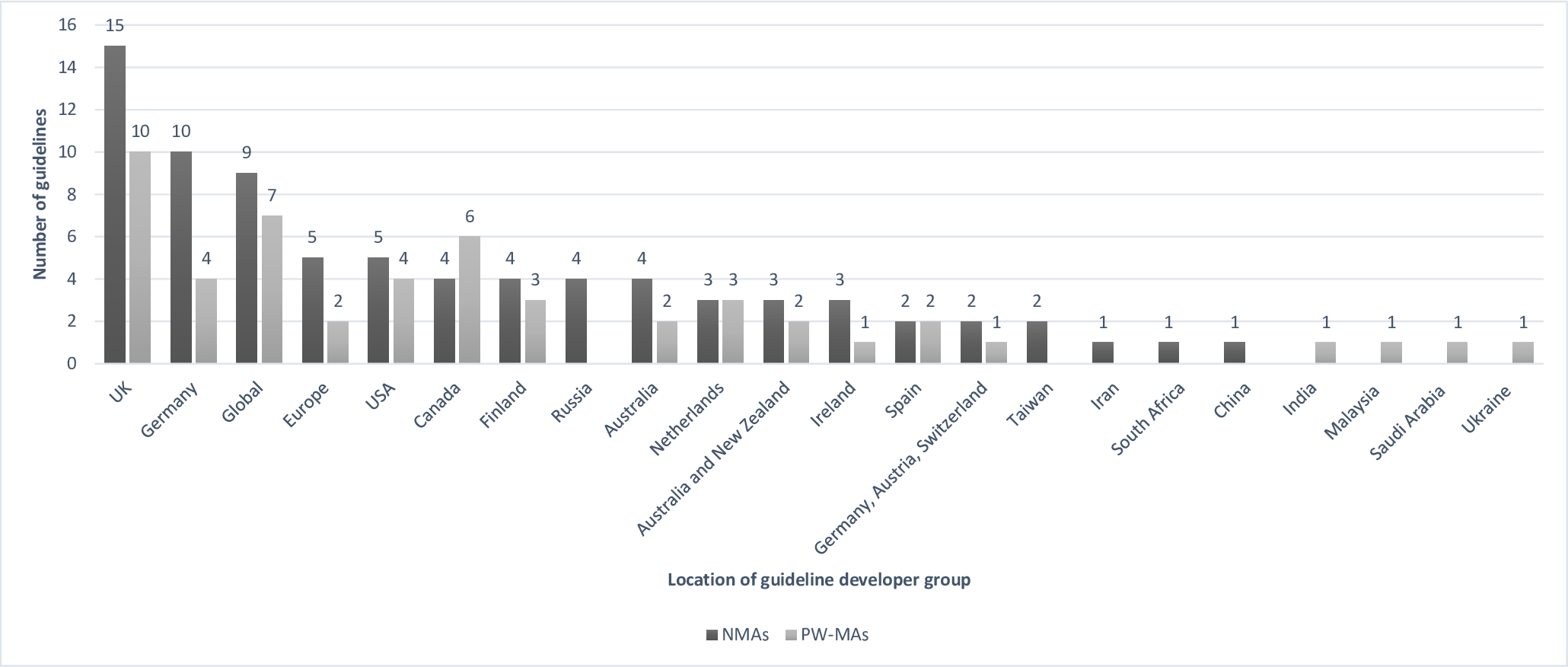

Supplement: S5 Fig — (TIF) [file pone.0315563.s020.tif]

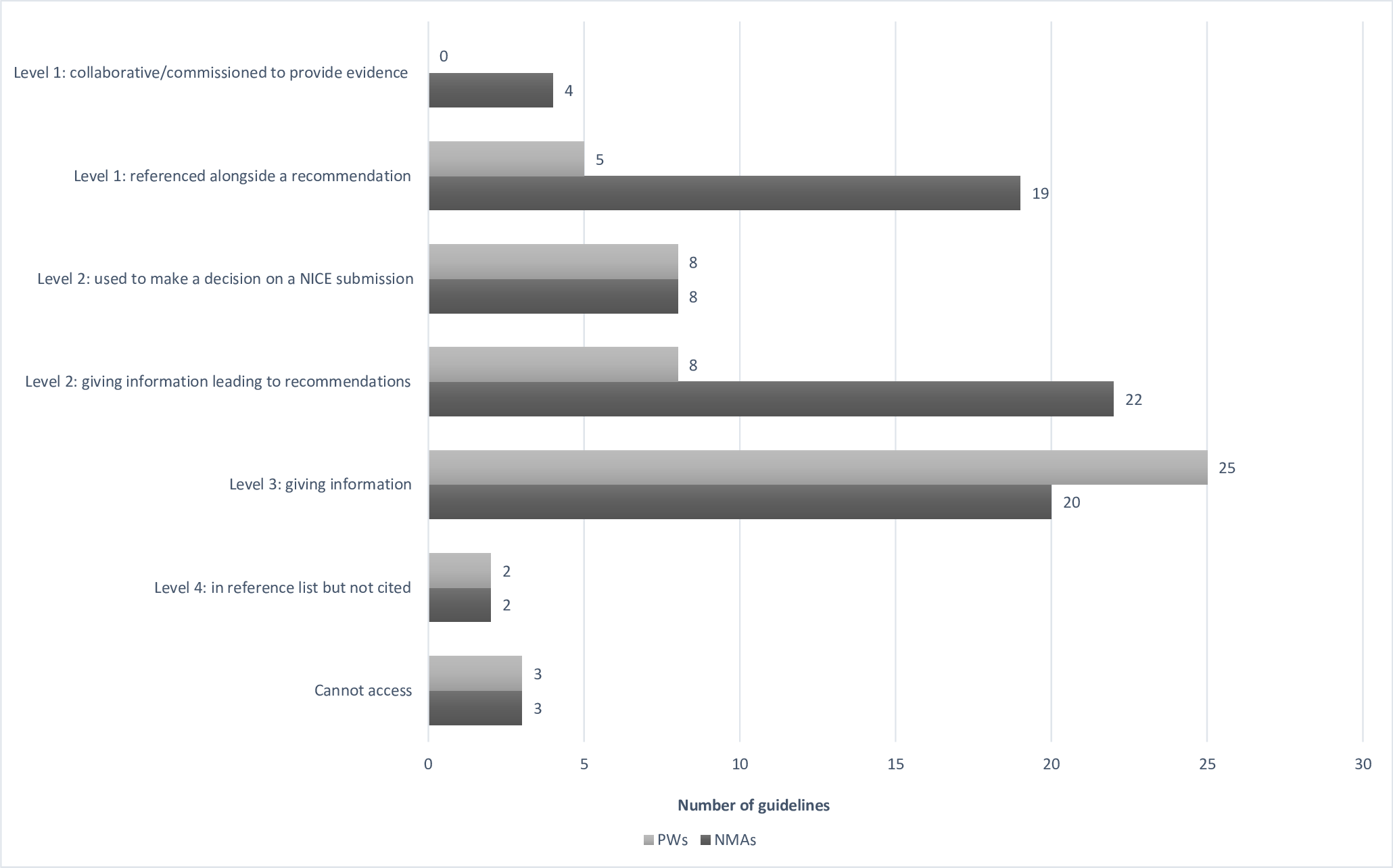

Supplement: S6 Fig — (TIF) [file pone.0315563.s021.tif]
