# Supplementary material for: The impact of Cochrane Reviews that apply network meta-analysis in clinical guidelines: A systematic review
Source: PLoS One. 2024 Dec 26;19(12):e0315563. doi: 10.1371/journal.pone.0315563 (PMC11671017; doi:10.1371/journal.pone.0315563)
Supplement: Attachment — Submitted filename: Response to Reviewers.docx [file pone.0315563.s022.docx]

|  | **Sarah Donegan**  **PhD, MSc, BSc**  Department of Health Data Science Block F Waterhouse Building 1-5 Brownlow Street Liverpool L69 3GL  T 0151 794 9721 Email donegan@liverpool.ac.uk  [www.liverpool.ac.uk](http://www.liverpool.ac.uk) |
| --- | --- |

20^th^ October 2024

Dear Dr Nishant Premnath Jaiswa

**RE: PONE-D-24-27301**

**The impact of Cochrane Reviews that apply network meta-analysis in clinical guidelines: a systematic review.**

Thank you for considering the above article for publication and for the helpful feedback and comments.

We have considered and addressed each comment as detailed below in red text.

Yours sincerely

Dr Sarah Donegan

Lecturer in Evidence Synthesis

**Editor’s comments**

Journal requirements:

When submitting your revision, we need you to address these additional requirements.

1. Please ensure that your manuscript meets PLOS ONE's style requirements, including those for file naming. The PLOS ONE style templates can be found at

https://journals.plos.org/plosone/s/file?id=wjVg/PLOSOne_formatting_sample_main_body.pdf and

[https://journals.plos.org/plosone/s/file?id=ba62/PLOSOne_formatting_sample_title_authors_affiliations.pdf](https://c05y1x9s.r.us-east-2.awstrack.me/L0/https:%2F%2Fjournals.plos.org%2Fplosone%2Fs%2Ffile%3Fid=ba62%2FPLOSOne_formatting_sample_title_authors_affiliations.pdf/1/010f019226727c27-47e94d2f-ba0e-453c-b107-ac8f8b1fc978-000000/C_ll8VcxJwtXs4OriZf9B41fQOw=178)

The submission now meets style requirements.

2. Thank you for stating the following in the Competing Interests section: [I have read the journal's policy and the authors of this manuscript have the following competing interests: CTS and SD were statistical editors for Cochrane Review Groups, ZA was co-ordinating editor for the Cochrane Pregnancy and Childbirth Group, and CTS is a co-convenor for the Cochrane Statistical Methods Group.]. Please confirm that this does not alter your adherence to all PLOS ONE policies on sharing data and materials, by including the following statement: "This does not alter our adherence to PLOS ONE policies on sharing data and materials.” (as detailed online in our guide for authors http://journals.plos.org/plosone/s/competing-interests). If there are restrictions on sharing of data and/or materials, please state these. Please note that we cannot proceed with consideration of your article until this information has been declared. Please include your updated Competing Interests statement in your cover letter; we will change the online submission form on your behalf.

We have included the necessary statement in the competing interests section of the manuscript as follows: **‘Conflict of interest:** CTS and SD were statistical editors for Cochrane Review Groups, ZA was co-ordinating editor for the Cochrane Pregnancy and Child Birth Group, and CTS is a co-convenor for the Cochrane Statistical Methods Group. This does not alter our adherence to PLOS ONE policies on sharing data and materials.’

3. As required by our policy on Data Availability, please ensure your manuscript or supplementary information includes the following:

A numbered table of all studies identified in the literature search, including those that were excluded from the analyses.

For every excluded study, the table should list the reason(s) for exclusion.

For the search for NMAs:

File S2 presents a table with each excluded review with reason for exclusion. Data Tables S1-S2 present each included review. The tables are referred to in the results text (‘*NMA review profile’ section)*.

For the search for PW-MAs:

A search was run for each included NMA and File S3 displays the outcome of each search, and when and why reviews were excluded with references to excluded reviews.

If any of the included studies are unpublished, include a link (URL) to the primary source or detailed information about how the content can be accessed.

All included reviews are published in CDSR.

A table of all data extracted from the primary research sources for the systematic review and/or meta-analysis. The table must include the following information for each study:

Name of data extractors and date of data extraction

Confirmation that the study was eligible to be included in the review.

All data extracted from each study for the reported systematic review and/or meta-analysis that would be needed to replicate your analyses.

Data were extracted over a period of many months with some data extraction repeated for particular characteristics or studies (records of the date of extraction for each review/guideline were not kept). The methods text states who extracted data and states ‘SD extracted data from the included reviews, their associated guidelines, and summary information in CDSR regarding guidelines using a form.’

For NMAs, Data Tables S1-S2 present the data extracted from each review and Data Table 3 presents the data extracted for the guideline. For PW-MAs, Data Tables S4-S5 present the data extracted from each review and Data Table 6 presents the data extracted for the guideline.

If data or supporting information were obtained from another source (e.g. correspondence with the author of the original research article), please provide the source of data and dates on which the data/information were obtained by your research group.

No data were obtained from other sources.

If applicable for your analysis, a table showing the completed risk of bias and quality/certainty assessments for each study or outcome.  Please ensure this is provided for each domain or parameter assessed. For example, if you used the Cochrane risk-of-bias tool for randomized trials, provide answers to each of the signalling questions for each study. If you used GRADE to assess certainty of evidence, provide judgements about each of the quality of evidence factor. This should be provided for each outcome.

The risk of bias of studies was not assessed by the co-authors as part of this methodological review because it was a review of reviews rather than trials. Similarly, we did not assess the credibility of reviews ourselves using grade. The original review authors assessed risk of bias and generally applied grade and we summarised their relevant findings as part of this review.

An explanation of how missing data were handled.

A sentence has been added to the methods section as follows ‘Non-reported data were excluded from analyses.’.

This information can be included in the main text, supplementary information, or relevant data repository. Please note that providing these underlying data is a requirement for publication in this journal, and if these data are not provided your manuscript might be rejected.

This information is presented in supplementary material.

Reviewer #1:

We are very grateful that you have reviewed our article and pointed out the sections which need clarification to help readers understand the manuscript more easily.

1. The eligibility criteria for including reviews in this study need to be spelt out well.

We have provided more detail regarding eligibility criteria for clarification, as follows: ‘Cochrane Reviews of interventions that applied NMA were included. We excluded protocols, withdrawn reviews, overview of reviews, and diagnostic test accuracy reviews. We excluded reviews that used indirect comparison methods (e.g. calculating the difference between results obtained from pairwise meta-analyses) rather than NMA models because indirect comparisons are made in reviews without using searchable terminology. We excluded reviews that applied NMA but for which the main summary of findings table was based on PW-MA or single trials because we primarily aimed to explore the uptake of NMA results in clinical guidelines.’

2. It appears that only 55% of the citations identified were screened independently. This needs explanation.

Originally, rather than searching CDSR, this project was based on a spreadsheet compiled by Cochrane UK as their record of where Cochrane NMA reviews were cited. This spreadsheet is then used by Cochrane UK/Wiley to populate the guideline citation information in CDSR. James initially assessed the eligibility of NMAs in their spreadsheet according to our inclusion criteria. We subsequently decided that searching for NMA reviews in the CDSR using a search strategy was a more systematic approach. Searching CDSR detected additional potentially relevant NMA reviews that were not in the original spreadsheet. This is why James only assessed 55% of the search results located from searching CDSR. James subsequently gained new employment and therefore did not assess the additional reviews located from CDSR. I have not included these details in the manuscript.

3. The rationale for comparing reviews with NMA against those with PW-MA is unclear, unless they are on the same or similar topics.

Thank you for highlighting this. We now provide further information in the introduction (page 7) as follows:

‘Hypothetically, NMA reviews may be less likely to be cited in guidelines than PW-MA reviews because of lack of understanding or cautiousness regarding NMA methods. Previously, in a review of guidelines, Lunny *et al* found that NMA reviews are less cited than PW-MA reviews [15]. However, to our knowledge, no research has reviewed PW-MA reviews and summarised their citation in guidelines or compared samples of NMA reviews and PW-MA reviews in terms of citation. As a post hoc sub-study, a cohort of Cochrane reviews that applied PW-MA is matched to the NMA reviews to compare their citation in guidelines.

We anticipate that our research will highlight the extent of potential research waste, help review authors to maximise the impact of their reviews, aid guidelines developers to improve and increase the use of NMA and PW-MA reviews, and lead to improved collaboration to increase research efficiency.’

4. How were reviews with NMA and those with PW-MA ‘matched’? It appears that matching was done on the basis of publication date. It this is true, it could create an imbalance on account of the fact that the review topic(s)/methods/interventions/etc. may be so dissimilar as to make them incomparable.

We have included additional text in the discussion (page 24) as a limitation of the study to emphasise that the comparison between NMAs and PW-MAs could be subject to confounding and bias, as follows:

‘We were able to match 38 of the 60 included NMA to a PW-MA published by the same Cochrane Review Group and within a month of each other. However, there may be other confounding factors that require matching to ensure the comparison of NMA and PW-MA reviews is unbiased. We did not match additional factors because there is no research exploring which factors are significant, and, if we matched for other factors, we are likely to be able to match even fewer PW-MA reviews to NMA reviews. Ideally, the NMA and PW-MA reviews would be identical in terms of all characteristics (e.g. aims, publication date) except the application of the NMA or PW-MA methodology. However, this would not be feasible because such reviews are not published in CDSR.’

We also inserted ‘appear to’ in the conclusion of the study to say ‘Cochrane NMA reviews appear to have more impact than PW-MA reviews, but many are not cited in guidelines.’

5. Please remove the side-headings in the Results section.

We believe that, in general, the sub-headings are needed to guide readers through the results.

Reviewer #2:

Thank you for reviewing our manuscript and for highlighting these important methodological points which we respond to below in detail.

Thank you for the opportunity to go through the wonderful article, The authors here tried to investigate the extent to which Cochrane Reviews that utilize network meta-analysis (NMA) are cited in guidelines. The study aims to explore various factors related to the citation of these reviews, including review characteristics, citation timing, location, authorship, and the specific contribution of the reviews to the guidelines. Additionally, the study compares the citation rates of NMA reviews to those of pairwise meta-analysis (PW-MA) reviews to assess the potential impact of NMA on guideline development.

The write up is good and easy to understand. I have concerns regarding the methodology –

1. A clear and exact search strategy needs to be given instead just the key terms.
We have edited the manuscript text and provided the search strategy in supplementary material, as follows: ‘The CDSR was searched for NMAs to 15^th^ January 2024 (see File S1 for search strategy). Reviews were filtered to include only intervention reviews.’

**File S1: Search strategy for Cochrane Database of Systematic Reviews**

|  | **Search term** |
| --- | --- |
| #1 | ("network meta-analysis"):ti,ab,kw |
| #2 | ("mixed treatment comparison meta-analysis"):ti,ab,kw |
| #3 | ("multiple treatment comparison meta-analysis"):ti,ab,kw |
| #4 | {OR #1-#3} |
|  | Filter by Review Type Intervention |

2.The identified NMA reviews need to be grouped based on their field/ specific areas addressed. Every other study does not get cited in a guideline if it is not an immediate policy priority. So, an NMA review getting citation or not depends largely on if it is a priority topic at that point of time or not. This factor is a huge predictor in this study and authors have not accounted for this. Grouping NMA reviews would help in that, later a stratified analysis should be presented.

Thank you for this very thought-provoking comment. We agree that reviews are more likely to be cited if they address important unanswered questions that are framed in a way to be relevant to guidelines. We believe that there are also other contributing factors, such as collaboration with guideline developers, quality of review etc. We agree that grouping NMAs according to priority (high vs low priority) and carrying out similar analyses/summaries to those in this article could be highly interesting. However, we do not think that it is feasible to group studies according to whether they are a priority topic because this would require collaboration of clinicians across a range of many areas, and prioritising the review topics across many areas would be highly subjective and potentially controversial. We have added a discussion point as follows:

‘We would expect that reviews are more likely to be cited if they address important unanswered questions that would be considered to be high priority to researchers and guideline developers. However, we did not explore whether high priority reviews were more likely to be published than low priority reviews because, to assess the priority level of each review would require collaboration of clinicians across a range of many areas and would be highly subjective and potentially controversial.’

3.The procedure to match PW-MA reviews to NMAs is also a little sketchy. Was it done completely based on temporal proximity if they come from the same review group? Though they come from same review groups, the topics can be completely different, and thereby how can these be compared? If the authors matched the PW-MA reviews based on objectives, that would have been comparable.

Please see the response to reviewer 1 comment 4.

Please clarify these questions before resubmission of the manuscript.
